# Differential Acute Kidney Injury Profiles of GLP-1RAs and SGLT2is: A Network Meta-Analysis

**DOI:** 10.3390/ijms27094137

**Published:** 2026-05-06

**Authors:** Chih-Sung Liang, Chih-Wei Hsu, Jiann-Jy Chen, Chao-Ming Hung, Bing-Yan Zeng, Wei-Chieh Yang, Mein-Woei Suen, Hung-Yu Wang, Andre F. Carvalho, Brendon Stubbs, Yen-Wen Chen, Tien-Yu Chen, Wei-Te Lei, Shih-Pin Hsu, Yow-Ling Shiue, Cheng-Ta Li, Kuan-Pin Su, Bing-Syuan Zeng, Ping-Tao Tseng

**Affiliations:** 1 Department of Psychiatry, Beitou Branch, Tri-Service General Hospital, School of Medicine, National Defense Medical University, Taipei 112, Taiwan; lcsyfw@gmail.com; 2 Department of Psychiatry, National Defense Medical University, Taipei 114, Taiwan; 3 Department of Psychiatry, Kaohsiung Chang Gung Memorial Hospital and Chang Gung University College of Medicine, Kaohsiung 833, Taiwan; harwicacademia@gmail.com; 4 Prospect Clinic for Otorhinolaryngology & Neurology, Kaohsiung 811, Taiwan; jiannjy@yahoo.com.tw (J.-J.C.); kevinachen0527@gmail.com (Y.-W.C.); 5 Department of Internal Medicine, E-Da Cancer Hospital, I-Shou University, No. 21, Yida Rd., Yanchao Dist., Kaohsiung 824, Taiwan; 6 Division of General Surgery, Department of Surgery, E-Da Cancer Hospital, I-Shou University, Kaohsiung 824, Taiwan; ed100647@edah.org.tw; 7 School of Medicine, College of Medicine, I-Shou University, Kaohsiung 840, Taiwan; a.pin.hsu@gmail.com; 8 Department of Internal Medicine, E-Da Dachang Hospital, I-Shou University, Kaohsiung 807, Taiwan; holdinggreat@yahoo.com.tw; 9 Institute of Biomedical Sciences, National Sun Yat-sen University, Kaohsiung 804, Taiwan; shirley@imst.nsysu.edu.tw; 10 Department of Pediatrics, Ping An Medical Clinic, Tainan 708, Taiwan; medarchies@gmail.com; 11 Department of Psychology, College of Medical and Health Science, Asia University, Taichung 413, Taiwan; blake@asia.edu.tw; 12 Gender Equality Education and Research Center, Asia University, Taichung 413, Taiwan; 13 Department of Medical Research, Asia University Hospital, Asia University, Taichung 413, Taiwan; 14 Department of Medical Research, China Medical University Hospital, China Medical University, Taichung 404, Taiwan; 15 Kaohsiung Municipal Kai-Syuan Psychiatric Hospital, Kaohsiung 802, Taiwan; hywang1975@gmail.com; 16 Innovation in Mental and Physical Health and Clinical Treatment (IMPACT) Strategic Research Centre, School of Medicine, Barwon Health, Deakin University, Geelong, VIC 3220, Australia; canaldasaudemental@gmail.com; 17 Psychological Medicine, Institute of Psychiatry, Psychology and Neuroscience (IoPPN), King’s College London, London WC2R 2LS, UK; brendon.stubbs@kcl.ac.uk; 18 Comprehensive Centre for Clinical Neurosciences and Mental Health, Medical University of Vienna, 1090 Vienna, Austria; 19 Clinical Division of Social Psychiatry, Department of Psychiatry and Psychotherapy, Medical University of Vienna, 1090 Vienna, Austria; 20 Division of Psychology and Mental Health, Manchester Academic Health Science Centre, University of Manchester, Manchester M13 9PL, UK; 21 Department of Psychiatry, Tri-Service General Hospital, Taipei 114, Taiwan; verducciwol@gmail.com; 22 Department of Psychiatry, College of Medicine, National Defense Medical University, Taipei 114, Taiwan; 23 Division of Pediatric Allergy, Immunology, and Rheumatology, Department of Pediatrics, Hsinchu Municipal MacKay Children’s Hospital, Hsinchu 300, Taiwan; lazyleisure@gmail.com; 24 Graduate Institute of Clinical Medical Sciences, College of Medicine, Chang Gung University, Taoyuan 333, Taiwan; 25 Department of Neurology, E-Da Hospital, I-Shou University, Kaohsiung 824, Taiwan; 26 Institute of Precision Medicine, National Sun Yat-sen University, Kaohsiung 804, Taiwan; 27 Department of Psychiatry, Taipei Veterans General Hospital, Taipei 112, Taiwan; on5083@msn.com; 28 Division of Psychiatry, School of Medicine, National Yang Ming Chiao Tung University, Taipei 112, Taiwan; 29 Institute of Brain Science and Brain Research Center, School of Medicine, National Yang Ming Chiao Tung University, Taipei 112, Taiwan; 30 Office of Research and Development, Asia University, Taichung 413, Taiwan; cobolsu@gmail.com; 31 College of Medicine, China Medical University, Taichung 404, Taiwan; 32 An-Nan Hospital, China Medical University, Tainan 709, Taiwan; 33 Mind-Body Interface Research Center (MBI Lab & Care), China Medical University Hospital, Taichung 404, Taiwan; 34 School of Medicine, College of Medicine, National Sun Yat-sen University, 70 Lienhai Road, Gushan District, Kaohsiung 804, Taiwan

**Keywords:** network meta-analysis, GLP-1 receptor agonist, SGLT2 inhibitor, acute kidney injury, tirzepatide, adverse effects

## Abstract

Although glucagon-like peptide-1 receptor agonists (GLP-1RAs) and sodium–glucose co-transporter 2 inhibitors (SGLT2is) have demonstrated protective effects against chronic kidney disease, their impact on acute kidney injury (AKI) remains unclear. AKI and chronic kidney disease share overlapping clinical features but differ in pathogenesis and risk profiles. Previous analyses often grouped diverse agents into single categories, potentially concealing medication-specific renal risks. Given the widespread assumption of renoprotection, particularly among newer agents, there is a need to evaluate the comparative AKI risk of GLP-1RAs and SGLT2is at the individual drug and dose level. We performed a Bayesian network meta-analysis (NMA) following Cochrane-recommended methodology for safety-focused assessments. A systematic literature search across eight databases identified 67 randomized controlled trials (RCTs), including 199,877 participants. Eligible trials reported AKI outcomes or sufficiently explicit acute renal injury-related events associated with GLP-1RA or SGLT2i interventions. The primary outcome was the incidence of AKI; all-cause dropout was analyzed as a general tolerability measure. Odds ratios (ORs) with 95% credible intervals (CrIs) were calculated, and surface under the cumulative ranking curves (SUCRA) were used to estimate relative safety rankings. Only high-dose tirzepatide (10–15 mg/week) was associated with a significantly increased risk of AKI compared to controls (absolute risk difference: 0.28%; number needed to harm: 357). In contrast, lixisenatide, high-dose canagliflozin (300 mg/day), empagliflozin, and dapagliflozin were associated with reduced AKI risk. Risk rankings consistently identified high-dose tirzepatide as the most likely to induce AKI. Subgroup analyses excluding patients with baseline renal impairment yielded consistent results. High-dose tirzepatide may elevate AKI risk despite its metabolic benefits. Clinicians should assess renal vulnerability when prescribing GLP-1RAs or SGLT2is, particularly in patients with preserved kidney function. Further prospective trials are needed to clarify causal mechanisms and inform clinical decision-making.

## 1. Introduction

Glucagon-like peptide-1 receptor agonists (GLP-1RAs) and sodium–glucose co-transporter 2 inhibitors (SGLT2is) represent two classes of novel antihyperglycemic agents with mechanisms of action distinct from traditional glucose-lowering therapies [1]. In addition to their glycemic control efficacy, these agents have shown a spectrum of secondary benefits, including cardiovascular and renal protective effects. Among these, SGLT2is have consistently demonstrated reductions in the progression of chronic kidney disease in patients with type 2 diabetes [2], and GLP-1RAs have similarly been linked to favorable renal outcomes in this population [3].

Despite these overlapping therapeutic applications, the underlying etiology and clinical course of acute kidney injury (AKI) differs substantially from that of chronic kidney disease [4]. AKI—a sudden and often reversible decline in renal function—commonly occurs in individuals with diabetes mellitus [5] or obesity [6], both of which are typical indications for GLP-1RAs or SGLT2is. As a result, the question of whether these drugs mitigate or contribute to AKI risk has become increasingly important, particularly since patients receiving these therapies often present with baseline AKI risk factors. When RCTs are limited by design or scope, meta-analytic methods offer valuable insight by integrating data from larger populations, thereby enhancing generalizability to clinical practice. This is particularly relevant when investigating adverse outcomes, where individual studies may be underpowered [7,8].

Previous pairwise meta-analyses have grouped pharmacologically diverse GLP-1RAs or SGLT2is together, often concluding renoprotective effects against AKI. However, these pooled analyses frequently yield wide confidence intervals and high heterogeneity, which may conceal drug-specific safety concerns [9,10]. Supporting this concern, several case reports have described AKI following tirzepatide initiation. For example, Farhat et al. [11] reported AKI in a 59-year-old man with no prior renal disease one month after starting tirzepatide. Similarly, Aleman Espino et al. [12] described AKI in a 42-year-old woman shortly after initiating the same drug. Moreover, a large-scale trial by Heerspink and colleagues noted a decline in renal function among patients with reduced baseline eGFR (<60 mL/min per 1.73 m^2^), although it did not formally meet AKI criteria [13].

While these prior meta-analyses [9,10] have attempted to examine AKI risk in the context of GLP-1RA or SGLT2i use, their methodological limitations—particularly the pooling of drugs with different pharmacodynamics and dose ranges—may obscure true drug-specific effects. Recent network meta-analyses (NMAs) have sought to overcome some of these issues but often lack inclusion of newer agents such as tirzepatide or lixisenatide, and rarely account for dose stratification. Thus, their clinical utility in guiding renal safety decisions remains limited.

By incorporating multiple treatment comparisons and dosage-specific classifications, NMAs can enable both direct and indirect comparisons between agents, providing a more refined framework for evaluating safety and efficacy [14]. To date, no NMA has specifically addressed the risk of AKI across GLP-1RAs and SGLT2is stratified by individual drug and dosage. Building on our group’s prior work examining neurodegenerative disorder [15,16,17], altered oncology outcomes [18,19,20,21], antiseptic property [22], and gastroenterology safety [23] associated with these medications, the present study aims to systematically evaluate and compare the AKI risk associated with individual GLP-1RAs and SGLT2is at various dosing regimens.

## 2. Results

### 2.1. Study Selection and Characteristics

**Figure 1:**
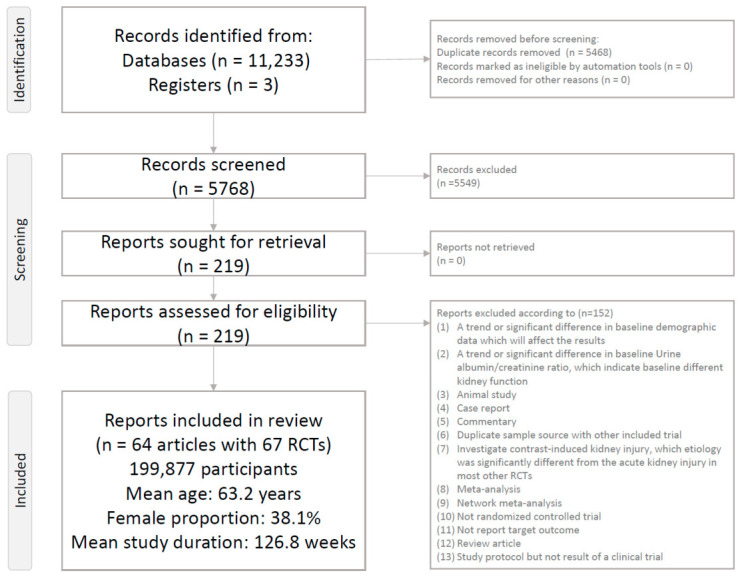
PRISMA2020 Flowchart of current network meta-analysis.

The literature search and screening process are outlined in Figure 1. After excluding 152 articles for not meeting inclusion criteria (see Table S3) [9,10,11,12,24,25,26,27,28,29,30,31,32,33,34,35,36,37,38,39,40,41,42,43,44,45,46,47,48,49,50,51,52,53,54,55,56,57,58,59,60,61,62,63,64,65,66,67,68,69,70,71,72,73,74,75,76,77,78,79,80,81,82,83,84,85,86,87,88,89,90,91,92,93,94,95,96,97,98,99,100,101,102,103,104,105,106,107,108,109,110,111,112,113,114,115,116,117,118,119,120,121,122,123,124,125,126,127,128,129,130,131,132,133,134,135,136,137,138,139,140,141,142,143,144,145,146,147,148,149,150,151,152,153,154,155,156,157,158,159,160,161,162,163,164,165,166,167,168,169,170,171], a total of 64 studies comprising 67 RCTs were included in the analysis (see Table S4) [28,97,107,144,172,173,174,175,176,177,178,179,180,181,182,183,184,185,186,187,188,189,190,191,192,193,194,195,196,197,198,199,200,201,202,203,204,205,206,207,208,209,210,211,212,213,214,215,216,217,218,219,220,221,222,223,224,225,226,227,228,229,230,231,232,233]. These trials enrolled 199,877 participants, with a mean age of 63.2 years (range: 45.1–74.2 years), and a mean female representation of 38.1% (range: 20.8–81.6%). The average follow-up duration across studies was 126.8 weeks (range: 4–281 weeks). Investigated GLP-1RAs included albiglutide, dulaglutide, efpeglenatide, exenatide, liraglutide, lixisenatide, semaglutide, and tirzepatide. SGLT2is evaluated included bexagliflozin, canagliflozin, dapagliflozin, empagliflozin, ertugliflozin, and sotagliflozin.

The distribution of key clinical and design-related effect modifiers across treatment nodes is summarized in Table S9. The network was largely placebo-centered, with the placebo/control node contributing the largest number of trials and participants, whereas several active treatment nodes were informed by relatively few studies. Age and sex distributions were broadly comparable across many nodes; however, visible between-node imbalances were observed in study duration, the proportion of trials including patients with renal impairment, and the proportion of diabetes-enriched populations. These differences should be considered when interpreting indirect comparisons and do not eliminate concern regarding transitivity. In particular, several SGLT2 inhibitor nodes included a higher proportion of trials enrolling patients with renal impairment and had longer follow-up durations, whereas tirzepatide nodes tended to have shorter follow-up and did not include renal impairment-enriched trials. No included trial was restricted to a single sex or to a specific age-defined population. However, because age and sex may still act as study-level effect modifiers across treatment nodes, mean age and female proportion were extracted and summarized in Table S9 to improve transparency regarding potential between-node differences.

### 2.2. Primary Outcome: Acute Kidney Injury Events

**Figure 2:**
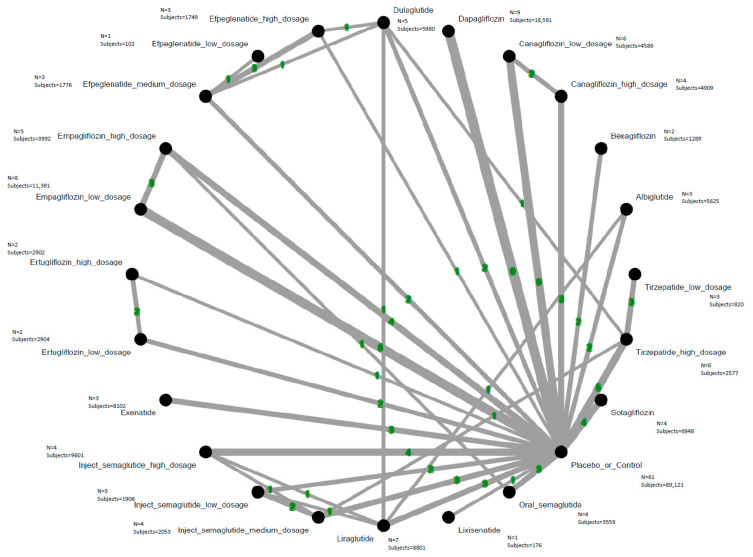
Network structure of the primary outcome: acute kidney injury. The overall structure of the network meta-analysis. The lines between nodes represent direct comparisons from various trials, with the numbers over the lines indicating the number of trials providing these comparisons for each specific treatment. The thickness of the lines corresponds to the number of trials linked to the network.

**Figure 3:**
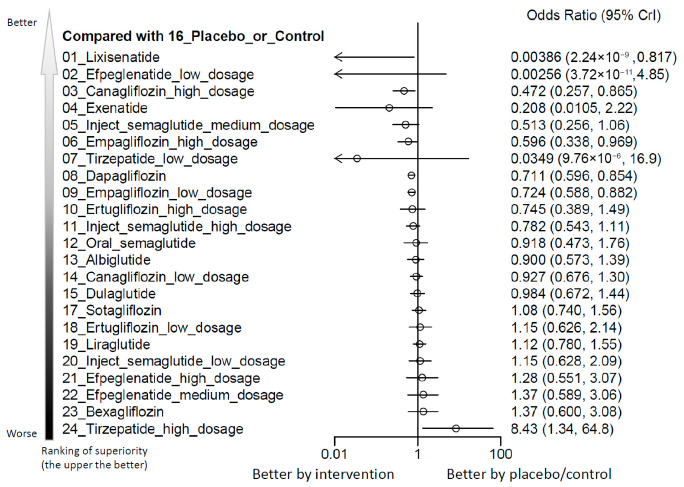
Forest plot of primary outcome: acute kidney injury. When the effect size (expressed as odds ratio) is less than 1, the specified treatment is associated with fewer acute kidney injury events compared to placebo/controls.

Our analysis identified that among all evaluated agents and dosages, only high-dose tirzepatide (10–15 mg/week) was associated with a statistically significant elevation in the risk of AKI when compared with control treatments. The estimated absolute risk difference was 0.28%, corresponding to a number needed to harm (NNH) of 357.34 patients. Although high-dose tirzepatide was the only regimen associated with a statistically significant increase in AKI risk, the estimate was based on sparse-event data with wide credible intervals and should therefore be interpreted cautiously. Conversely, lixisenatide, high-dose canagliflozin (300 mg/day), empagliflozin, and dapagliflozin were associated with a lower incidence of AKI compared to controls. Among all regimens assessed, high-dose tirzepatide had the highest SUCRA-based risk ranking for AKI (Figure 2, Figure 3 and Figure S3 and Table 1). Although high-dose tirzepatide was the only regimen associated with a statistically significant increase in AKI risk, the estimate was based on sparse-event data with wide credible intervals, indicating limited information size and substantial imprecision.

### 2.3. Subgroup Analysis: RCTs Excluding Patients with Pre-Existing Renal Dysfunction

**Figure 4:**
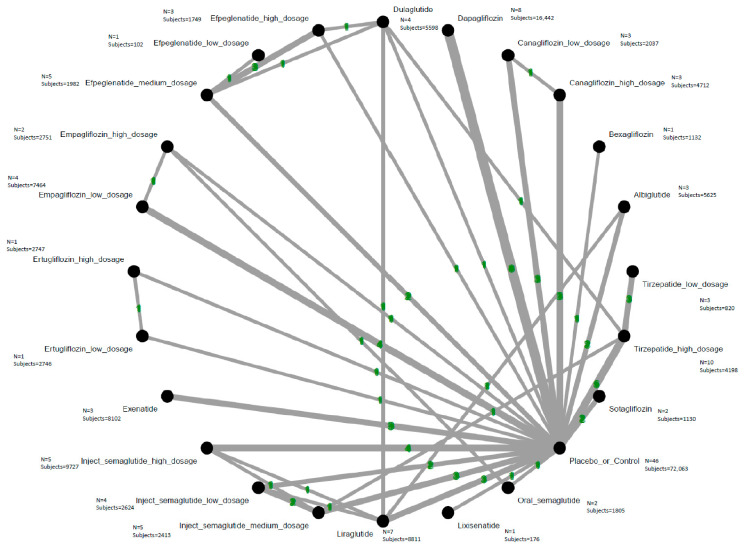
Network structure of the primary outcome: acute kidney injury in the subgroup focusing RCTs without definite underlying kidney failure. The overall structure of the network meta-analysis. The lines between nodes represent direct comparisons from various trials, with the numbers over the lines indicating the number of trials providing these comparisons for each specific treatment. The thickness of the lines corresponds to the number of trials linked to the network.

**Figure 5:**
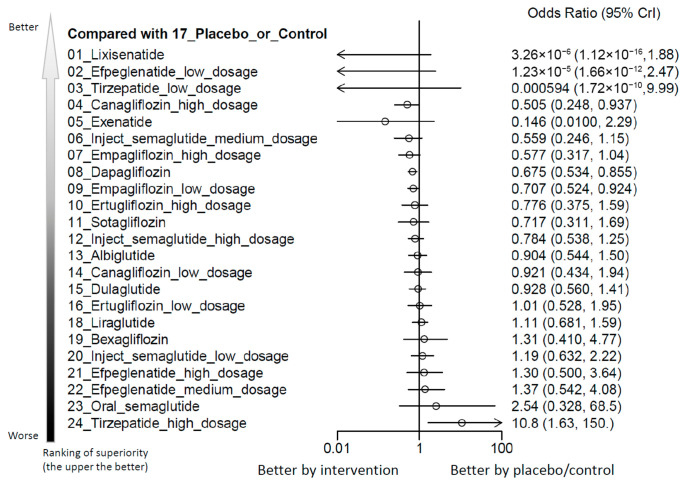
Forest plot of primary outcome: acute kidney injury in the subgroup focusing RCTs without definite underlying kidney failure. When the effect size (expressed as odds ratio) is less than 1, the specified treatment is associated with fewer acute kidney injury events compared to placebo/controls. Abbreviation: 95% CrIs: 95% credible intervals.

In the sensitivity analysis focusing on RCTs that did not enroll participants with baseline kidney disease, findings were consistent with the primary results. Specifically, high-dose tirzepatide (10–15 mg/week) remained significantly associated with increased AKI risk (ARD = 0.28%, NNH = 357.34). In contrast, high-dose canagliflozin (300 mg/day), dapagliflozin, and low-dose empagliflozin (1–10 mg/day) continued to demonstrate protective associations against AKI. Again, high-dose tirzepatide ranked highest for AKI risk (Figure 4, Figure 5 and Figure S3B, and Table 2).

### 2.4. Secondary Outcome: Dropout Rates as Safety Indicator

Among the included agents, tirzepatide and canagliflozin were associated with lower all-cause dropout rates relative to controls. This finding was interpreted descriptively as a general tolerability measure and not as validation of renal safety (Figures S1, S2 and S3C, and Table S5).

### 2.5. Treatment Rankings and Sensitivity Analyses

Comprehensive SUCRA-based treatment hierarchies are detailed in Table S6A–C and Figure S4A–F. Results remained robust in sensitivity analyses based on deviation-model frameworks, with no significant instability observed (Figure S5A–I).

### 2.6. Risk of Bias and Inconsistency Assessment

Risk-of-bias evaluation showed that 78.3% (367 of 469 items) of included studies were deemed low risk, 15.6% (73 items) unclear, and 6.1% (29 items) high risk (Figure S6A,B). Risk-of-bias assessment suggested that most included studies were judged to be at low risk, with a smaller proportion rated as unclear or high risk (Figure S6A,B). In node-splitting analyses, no statistically significant local inconsistency was detected among the estimable comparisons for the overall primary outcome, the subgroup excluding trials enrolling patients with underlying renal dysfunction, or the all-cause dropout network (Table S7A–C). However, many active-comparator contrasts had no direct head-to-head evidence and therefore could not be formally assessed for local inconsistency. Certainty of evidence varied across comparisons: several placebo-linked contrasts, including tirzepatide high-dose versus placebo/control, were rated as high certainty, whereas many indirect active-versus-active comparisons remained low certainty because of limited direct evidence and reliance on indirect estimates (Table S8A–C).

## 3. Discussion

This NMA offers the first comprehensive comparison of AKI risk across individual GLP-1RAs and SGLT2is, stratified by drug and dosage. Our findings suggest a possible dose-specific AKI signal associated with high-dose tirzepatide (10–15 mg/week), whereas lixisenatide, high-dose canagliflozin, empagliflozin, and dapagliflozin were associated with lower AKI risk. Importantly, statistical significance should not be equated with large clinical magnitude. Although high-dose tirzepatide was the only regimen associated with a statistically detectable increase in AKI risk, the absolute excess risk was small (0.28%), corresponding to a number needed to harm of 357. Therefore, this finding should be interpreted cautiously as a possible safety signal requiring confirmation, rather than as evidence of a large or immediately practice-changing renal hazard. However, this estimate was derived from sparse-event data with wide credible intervals and should be interpreted as hypothesis-generating rather than as stable comparative evidence.

The most striking observation from this NMA was the elevated AKI risk linked to high-dose tirzepatide, which contrasts with the broader narrative of renal safety surrounding newer glucose-lowering therapies. Prior meta-analyses predominantly focused on SGLT2is and often aggregated data from pharmacologically diverse agents without dose differentiation. This methodological limitation likely obscured drug-specific effects and introduced considerable statistical heterogeneity [9,10]. Additionally, previous analyses typically lacked inclusion of newer agents such as tirzepatide, and thus may not reflect the full renal safety spectrum of these treatment classes.

Although a few NMAs have attempted to examine renal adverse effects in this drug category [137,160,162], most employed pooled drug classifications similar to those used in conventional pairwise meta-analyses. Moreover, NMAs that included medication-specific analyses generally focused only on select SGLT2is (e.g., canagliflozin, dapagliflozin, empagliflozin) [42,91,98,140], and excluded newer agents such as lixisenatide and tirzepatide. By contrast, our NMA incorporated a broad range of individual agents and explicitly accounted for dose stratification. These methodological refinements enabled detection of differential AKI risks that would otherwise be obscured.

Tirzepatide, in particular, has gained considerable attention due to its metabolic efficacy and dual agonist mechanism (GLP-1 and GIP receptors). However, few studies have addressed its renal safety in detail. Two case reports [11,12] documented AKI onset shortly after initiating tirzepatide in individuals without prior renal dysfunction, establishing a temporal association. Additionally, the SURPASS-4 trial identified a dose-dependent decline in estimated glomerular filtration rate (eGFR) among participants with impaired baseline renal function, although it did not meet the formal definition of AKI [13]. At first glance, the apparent coexistence of chronic renoprotective effects and a possible acute kidney injury signal may appear paradoxical. However, these two observations are not necessarily biologically incompatible. Long-term benefits on chronic kidney disease progression and albuminuria may coexist with short-term vulnerability to AKI under specific clinical conditions, particularly when volume depletion or hemodynamic stress is superimposed. Incretin-based therapies, including GLP-1 receptor agonists, have previously been linked to AKI in case reports and reviews, most commonly through nausea, vomiting, diarrhea, and the resulting reduction in effective circulating volume rather than through established direct nephrotoxicity [234,235]. While causality cannot be established from case reports or secondary observations, these data reinforce the plausibility of our findings.

In this context, the present tirzepatide finding is better interpreted as a hypothesis-generating, dose-specific renal vulnerability signal rather than as evidence that tirzepatide is uniformly nephrotoxic. A plausible explanation is that higher-dose tirzepatide may increase susceptibility to AKI in some patients by amplifying gastrointestinal intolerance, dehydration, reduced oral intake, and hemodynamic instability, especially in the setting of underlying metabolic stress or limited renal reserve. Recent case reports describing AKI after tirzepatide exposure, including one report of acute tubular injury, are compatible with this hypothesis, although they do not establish causality [236,237]. The pathophysiological mechanisms by which tirzepatide might increase AKI risk remain unclear, but several hypotheses merit consideration. AKI etiologies are broadly classified as pre-renal, intrinsic, or post-renal [238]. Tirzepatide has been associated with an elevated risk of thromboembolic events [239], which could contribute to pre-renal ischemia. Moreover, its GIP receptor activity may indirectly influence calcium–phosphate metabolism via parathyroid hormone (PTH) modulation. Although GIP initially suppresses PTH, levels rebound rapidly within 60–90 min after administration [240], mimicking biochemical features seen in hyperparathyroidism—a known risk factor for urinary tract stones [241], which in turn can contribute to post-renal obstruction. Nonetheless, in the case described by Aleman Espino et al. [12], no evidence of nephrolithiasis was detected. It is also conceivable that tirzepatide may exert direct nephrotoxic effects; however, this remains speculative in the absence of supporting in vivo or in vitro evidence [12]. Further mechanistic research is needed to elucidate whether tirzepatide contributes to AKI through direct renal injury or secondary systemic effects. Accordingly, our results should not be interpreted as contradicting the broader cardiorenal literature on incretin-based therapies. Rather, they suggest that chronic kidney benefit and acute kidney vulnerability may operate on different timescales and through different pathophysiologic pathways. Further mechanistic work is needed to clarify whether this apparent dose-related signal reflects gastrointestinal fluid loss, altered renal perfusion, tubular stress, or other endocrine-metabolic pathways relevant to AKI susceptibility [3,242]. Beyond its comparative clinical implications, this study may also have translational relevance within molecular endocrinology and metabolism. The agents examined here are endocrine–metabolic therapies used across diabetes, obesity, and related cardiometabolic disorders, and the observed heterogeneity in AKI risk suggests that renal safety may not be a uniform class property. In particular, the apparent dose-specific signal with tirzepatide raises the possibility that distinct incretin-related, hemodynamic, or volume-regulatory mechanisms may contribute differentially to renal vulnerability. Although the present study does not establish molecular causality, it provides a clinically grounded framework for future mechanistic research into gut–kidney signaling, metabolic stress, and renal susceptibility in metabolically dysregulated populations.

### Strengths and Limitations

Our analysis possesses several methodological strengths. First, the NMA framework enabled head-to-head comparisons across multiple agents and dosages, thereby improving the precision and granularity of risk estimates. By limiting inclusion to RCTs and applying rigorous quality assessments, we minimized the risk of selection and measurement biases. The use of subgroup analysis—specifically excluding studies that enrolled patients with baseline renal impairment—allowed us to test whether our findings were robust across different clinical populations. Additionally, the separation of treatments by both compound and dose offers clinicians actionable data for patient-specific risk stratification.

However, this study is not without limitations. The focus on RCTs, while enhancing internal validity, may exclude real-world signals observed in observational cohorts or long-term registry studies. We standardized the terminology throughout the manuscript as AKI and rechecked the included RCTs to confirm that AKI events were directly reported in all included studies. Remaining limitations therefore relate primarily to sparse-event imprecision, indirect evidence, and between-node differences in study populations rather than to mixed inclusion of inferred renal injury outcomes. Another limitation is that this was a predominantly placebo-centered network, such that many clinically relevant active-comparator contrasts were informed mainly by indirect evidence. Although we summarized key clinical and design-related effect modifiers across treatment nodes (Table S9) and additionally examined baseline renal dysfunction in sensitivity analysis, Table S9 also revealed visible between-node imbalances rather than eliminating concern about transitivity. In particular, several SGLT2 inhibitor nodes were enriched for renal-impairment trials and had longer follow-up durations, whereas tirzepatide nodes tended to have shorter follow-up and no renal-impairment-enriched trials. These differences may have influenced indirect comparisons and therefore argue against overinterpretation of active-versus-active contrasts. Accordingly, comparisons between active agents should not be interpreted as equivalent to direct head-to-head randomized evidence. Although no significant local inconsistency was detected among the estimable comparisons, many clinically relevant active-comparator contrasts lacked direct head-to-head evidence and were therefore informed predominantly by indirect estimates. This explains why certainty remained high for some placebo-linked contrasts but lower for many indirect active-versus-active comparisons. Moreover, the modest absolute risk difference further suggests that statistical detectability should not be overinterpreted as implying a large clinical effect. Although no sex-specific or age-specific trials were included, differences in age and sex distribution across treatment nodes may still have influenced indirect comparisons. We therefore summarized these characteristics at the study level (Table S9); however, the present network meta-analysis could not determine whether age or sex modified AKI risk at the individual-patient level. Accordingly, demographic effect modification by age or sex cannot be excluded and should be explored in future individual-participant-data analyses. Although meta-analyses are sometimes criticized for inconsistencies in endpoint adjudication, they remain an indispensable tool for aggregating safety signals across disparate trials [7,8,243,244]. Our decision to include trials regardless of whether AKI was a primary or secondary endpoint may increase heterogeneity, but also reflects the reality that adverse renal outcomes are often underreported. Importantly, while many of the included RCTs did not explicitly aim to assess AKI risk, renal function changes were frequently monitored as part of safety evaluations. Subgroup analyses were used to minimize the confounding effect of underlying renal disease. We emphasize that all-cause dropout should not be interpreted as corroborative evidence for the AKI findings. This endpoint is influenced by multiple nonrenal factors, including efficacy, gastrointestinal adverse effects, adherence, follow-up duration, and trial design, and therefore serves only as a broad tolerability measure. Accordingly, the dropout analysis was retained only for descriptive tolerability context and not for validation of renal safety signals. Because the primary signal was based on sparse-event data with wide credible intervals, and because many clinically relevant active-comparator contrasts were informed predominantly by indirect evidence, the present findings should be viewed primarily as hypothesis-generating rather than as definitive comparative evidence of drug-specific renal harm. Consistent with this, GRADE certainty varied across comparisons rather than remaining uniformly high across the network. While several placebo-linked contrasts were supported by higher-certainty evidence, many indirect active-versus-active contrasts remained of low certainty because of limited direct evidence and reliance on indirect estimates.

These limitations underscore the need for future large-scale trials specifically designed to assess renal outcomes with emerging glucose-lowering therapies. Prospective trials that incorporate real-time biomarker assessments of tubular stress and injury may further clarify the mechanisms underlying these observations.

## 4. Materials and Methods

In line with Cochrane-recommended methodologies for safety evaluation [245], this NMA was designed to specifically investigate adverse renal outcomes, focusing on the incidence of AKI associated with GLP-1RAs and SGLT2is. The study followed the Preferred Reporting Items for Systematic Reviews and Meta-Analyses guidelines, including the extension for network meta-analyses (PRISMA-NMA) (Table S1) [246]. Our protocol was prospectively registered with PROSPERO (CRD42025648069) and approved by the Institutional Review Board of Tri-Service General Hospital, National Defense Medical Center (approval number: TSGHIRB E202516007).

### 4.1. Literature Search and Study Selection

We conducted comprehensive searches across multiple databases, including PubMed, Embase, Cochrane CENTRAL, ClinicalTrials.gov, ScienceDirect, ProQuest, Web of Science, and ClinicalKey, covering studies published up to 5 February 2025 (see Table S2). Two independent reviewers (PT Tseng and BY Zeng) screened titles, abstracts, and full texts. Any disagreements were resolved through discussion. Additional studies were identified by manually screening the reference lists of existing systematic reviews [33,45,101,125,129,171], meta-analyses [9,10,26,32,36,38,39,41,43,54,55,63,67,70,72,78,79,83,92,95,99,100,103,108,113,114,115,121,124,126,135,139,143,146,147,150,151,152,164,165,166,169,170], and prior NMAs [42,91,98,137,140,160,161,162]. No restrictions were applied based on language or geographic region.

### 4.2. Eligibility Criteria

We applied the PICOS framework (Population, Intervention, Comparison, Outcome, Study Design) to determine study eligibility: Population: Human participants with or without pre-existing renal dysfunction; Intervention: Any GLP-1RA or SGLT2i, regardless of dose; Comparator: Placebo, standard of care, or an active control; Outcomes: Directly reported incidence of AKI; Study design: Randomized controlled trials (RCTs).

Eligible studies were required to directly report AKI outcomes associated with GLP-1RA or SGLT2i interventions. After rechecking all included trials, we confirmed that AKI events were directly reported in the final included dataset.

Exclusion criteria included (1) non-RCTs; (2) RCTs without direct comparison of relevant agents; (3) studies lacking directly reported AKI outcomes; and (4) preclinical or animal trials.

### 4.3. Quality Assessment

Two reviewers independently assessed study quality using the Cochrane Risk of Bias Tool 1.0 [247]. Disagreements were adjudicated by a third reviewer.

### 4.4. Definition of Outcomes

AKI was used as the standardized term throughout this manuscript. Trial-level renal outcome definitions were reviewed individually, and studies were categorized according to whether AKI was explicitly reported or whether acute renal injury-related events were identified through broader renal adverse-event reporting. The diagnosis of AKI was according to KDIGO clinical practice guidelines for AKI [248]. As a secondary outcome, we analyzed all-cause dropout as a broad measure of trial retention and general tolerability, recognizing that it is not a renal-specific safety endpoint.

Dosage categories were defined according to the classification used in the original RCTs [28,97,107,144,172,173,174,175,176,177,178,179,180,181,182,183,184,185,186,187,188,189,190,191,192,193,194,195,196,197,198,199,200,201,202,203,204,205,206,207,208,209,210,211,212,213,214,215,216,217,218,219,220,221,222,223,224,225,226,227,228,229,230,231,232,233]:

1. Canagliflozin (SGLT2i): Low: 100 mg; High: 300 mg.
2. Efpeglenatide (GLP-1RA): Low: 2 mg; Medium: 4 mg; High: 6 mg.
3. Ertugliflozin (SGLT2i): Low: 5 mg; High: 15 mg.
4. Injectable Semaglutide (GLP-1RA): Low: 0.5 mg; Medium: 1.0 mg; High: 2.4 mg.
5. Empagliflozin (SGLT2i): Low: 1–10 mg; High: 25–50 mg.
6. Tirzepatide (Dual GIP/GLP-1RA): Low: 1–5 mg; High: 10–15 mg.

Given that baseline renal insufficiency is a well-established risk factor for AKI [4], we performed a subgroup analysis excluding RCTs that specifically recruited patients with advanced renal impairment. This subgroup analysis aimed to isolate drug effects on AKI risk in populations with preserved renal function.

### 4.5. Data Extraction and Management

Two investigators (PT Tseng and BY Zeng) independently extracted key trial data, including study design, population characteristics, interventions, outcomes, and adverse event data. Any missing or unclear information was requested from corresponding authors. Extraction procedures adhered to the Cochrane Handbook for Systematic Reviews of Interventions and current best practices in evidence synthesis [249].

### 4.6. Statistical Analysis

We implemented a Bayesian random-effects network meta-analysis in MetaInsight (v4.0.2), using its Bayesian analysis modules implemented through the R packages gemtc, BUGSNET, and bnma [250,251]. Binary outcomes were analyzed using arm-level event-count data (events and sample sizes). For dichotomous outcomes, the Bayesian network meta-analysis was implemented in MetaInsight using a binomial-likelihood framework with an appropriate link function for relative treatment effects. Because the primary endpoint was a rare adverse event, the resulting estimates were interpreted with caution in light of sparse-event data and wide credible intervals.

Effect sizes were reported as odds ratios (ORs) with 95% credible intervals (CrIs) and visualized using forest plots [252]. Four Markov chain Monte Carlo (MCMC) chains were run for 25,000 iterations, discarding the initial 5000 as burn-in. Posterior distributions were sampled with a thinning interval of 1, yielding 80,000 samples for inference. Model priors were set as non-informative, with mean = 0 and consistent precision across arms. Model fit was assessed using residual deviance and leverage plots. Convergence was evaluated by visual inspection of Gelman–Rubin diagnostic plots.

Node-splitting analysis was employed to assess inconsistency between direct and indirect comparisons [251,253]. Treatment rankings were calculated using the surface under the cumulative ranking (SUCRA) curve method and presented using Rank-O-Gram and SUCRA radial plots [254].

To assess the plausibility of transitivity in this predominantly placebo-centered network, we summarized major clinical and design-related effect modifiers across treatment nodes, including age, sex distribution, study duration, the proportion of trials including patients with renal impairment, and the proportion of trials including patients with diabetes mellitus (Table S9). These characteristics were examined descriptively to improve transparency regarding the comparability of study populations contributing to indirect comparisons.

### 4.7. Sensitivity Analyses

We conducted deviation-based sensitivity analyses to evaluate model stability [255]. The Grading of Recommendations Assessment, Development and Evaluation (GRADE) framework was used to rate the certainty of evidence for all comparisons [256].

### 4.8. Ethics

This study complied with the principles outlined in the Declaration of Helsinki and was approved by the relevant institutional review board.

## 5. Conclusions

This NMA provides the first comprehensive evaluation of AKI risk across a wide array of GLP-1RAs and SGLT2is, analyzed by individual agent and therapeutic dose. Our findings highlight a novel safety concern: high-dose tirzepatide (10–15 mg/week) may elevate the risk of AKI, particularly among patients with preserved baseline renal function. In contrast, agents such as lixisenatide, high-dose canagliflozin (300 mg/day), empagliflozin, and dapagliflozin appear to confer renal protection.

These results challenge the prevailing perception of class-wide renoprotection among GLP-1RAs and SGLT2is, underscoring the importance of a differentiated, agent-specific approach to clinical decision-making. Given that patients receiving these therapies frequently present with risk factors for renal dysfunction, our findings suggest that personalized medication selection and dose consideration are critical in minimizing adverse renal outcomes.

Until further prospective data are available, clinicians should remain vigilant when prescribing high-dose tirzepatide, particularly in individuals at heightened risk for AKI. Future research should prioritize trials explicitly designed to assess renal endpoints, clarify mechanistic pathways, and identify early biomarkers of renal stress associated with these agents.

## Figures and Tables

**Table 1:** League table of the primary outcome: acute kidney injury. Data presents as OR [95%CIs]. Pairwise (upper-right portion) and network (lower-left portion) meta-analysis results are presented as estimate effect sizes for the outcome of events of acute kidney injury. Interventions are reported in order of mean ranking of beneficially prophylactic effect on events of acute kidney injury, and outcomes are expressed as odds ratio (OR) (95% confidence intervals) (95%CIs). For the pairwise meta-analyses, OR of less than 1 indicates that the treatment specified in the row got more beneficial effect than that specified in the column. For the network meta-analysis (NMA), OR of less than 1 indicates that the treatment specified in the column got more beneficial effect than that specified in the row. Bold results indicate statistical significance. The gray background indicated the targeted medication.

01_ Lixisenatide															0.33 [0.01; 8.10]								
1.66 (0, 12164336259.31)	02_ Efpeglenatide _low_dosage																				0.33 [0.01; 8.28]		
0.01 (0, 1.78)	0.01 (0, 9.55)	03_ Canagliflozin _high_dosage											0.52 [0.22; 1.18]		0.53 [0.28; 0.98]								
0.02 (0, 7.38)	0.02 (0, 36.37)	2.29 (0.21, 42.4)	04_ Exenatide												0.51 [0.09; 2.93]								
0.01 (0, 1.57)	0.01 (0, 10.51)	0.93 (0.36, 2.33)	0.4 (0.02, 4.96)	05_Inject_ semaglutide_ medium_dosage						0.67 [0.11; 4.01]					0.53 [0.26; 1.08]				0.41 [0.19; 0.90]				0.67 [0.03; 16.39]
0.01 (0, 1.33)	0 (0, 8.81)	0.8 (0.38, 1.78)	0.35 (0.02, 3.84)	0.88 (0.38, 2.12)	06_Empagliflozin _high_dosage			0.78 [0.44; 1.37]			1.00 [0.06; 16.08]				0.64 [0.38; 1.07]								
0.22 (0, 8381.19)	0.01 (0, 152847.69)	13.1 (0.03, 53764.14)	5.99 (0.01, 76403.35)	13.37 (0.03, 58351.67)	17.68 (0.04, 54677.42)	07_Tirzepatide _low_dosage																	0.43 [0.07; 2.67]
0.01 (0, 1.15)	0 (0, 6.87)	0.67 (0.35, 1.23)	0.3 (0.01, 3.12)	0.72 (0.35, 1.51)	0.84 (0.46, 1.41)	0.05 (0, 24.18)	08_ Dapagliflozin								0.71 [0.62; 0.81]								
0.01 (0, 1.11)	0 (0, 6.67)	0.66 (0.34, 1.25)	0.29 (0.01, 2.99)	0.7 (0.35, 1.52)	0.82 (0.47, 1.36)	0.05 (0, 23.36)	0.98 (0.76, 1.3)	09_Empagliflozin _low_dosage							0.51 [0.09; 2.93]								
0.01 (0, 1.11)	0 (0, 7.06)	0.63 (0.26, 1.53)	0.27 (0.01, 3.18)	0.69 (0.27, 1.74)	0.79 (0.35, 1.73)	0.05 (0, 21.33)	0.95 (0.47, 1.89)	0.98 (0.47, 1.91)	10_Ertugliflozin _high_dosage						0.75 [0.40; 1.38]								
0 (0, 1.04)	0 (0, 6.26)	0.61 (0.31, 1.18)	0.28 (0.01, 2.86)	0.65 (0.32, 1.47)	0.75 (0.39, 1.38)	0.05 (0, 22.77)	0.91 (0.61, 1.34)	0.93 (0.6, 1.38)	0.96 (0.45, 2.06)	11_Inject_ semaglutide_ high_dosage					0.76 [0.58; 1.00]			3.05 [0.12; 75.53]					
**0 (0, 0.91)**	0 (0, 6.04)	0.51 (0.21, 1.27)	0.25 (0.01, 2.23)	0.55 (0.21, 1.45)	0.65 (0.28, 1.46)	0.04 (0, 20.91)	0.77 (0.4, 1.57)	0.79 (0.4, 1.55)	0.82 (0.32, 2.14)	0.85 (0.42, 1.8)	12_Oral_ semaglutide				0.84 [0.43; 1.64]								
**0 (0, 0.97)**	0 (0, 6.07)	0.52 (0.24, 1.13)	0.23 (0.01, 2.52)	0.57 (0.25, 1.34)	0.66 (0.32, 1.3)	0.04 (0, 20.39)	0.79 (0.5, 1.29)	0.8 (0.5, 1.31)	0.82 (0.38, 1.91)	0.86 (0.5, 1.55)	1.01 (0.46, 2.25)	13_ Albiglutide			0.87 [0.59; 1.29]			3.04 [0.12; 74.78]					
**0 (0, 0.91)**	0 (0, 5.56)	**0.51 (0.26, 0.96)**	0.23 (0.01, 2.44)	0.55 (0.26, 1.19)	0.64 (0.32, 1.13)	0.04 (0, 18.79)	0.77 (0.52, 1.1)	0.78 (0.52, 1.12)	0.81 (0.39, 1.67)	0.84 (0.52, 1.35)	0.98 (0.47, 2.04)	0.97 (0.56, 1.65)	14_Canagliflozin_ low_dosage		0.93 [0.71; 1.21]								
**0 (0, 0.83)**	0 (0, 5.22)	**0.48 (0.24, 0.97)**	0.21 (0.01, 2.3)	0.52 (0.24, 1.19)	0.6 (0.3, 1.15)	0.03 (0, 17.17)	0.72 (0.48, 1.11)	0.74 (0.47, 1.14)	0.76 (0.35, 1.67)	0.79 (0.48, 1.31)	0.93 (0.42, 2.03)	0.92 (0.5, 1.61)	0.93 (0.57, 1.6)	15_ Dulaglutide	1.00 [0.73; 1.38]			0.33 [0.01; 8.22]					0.63 [0.03; 15.80]
**0 (0, 0.82)**	0 (0, 4.85)	**0.47 (0.26, 0.86)**	0.21 (0.01, 2.22)	0.51 (0.26, 1.06)	**0.6 (0.34, 0.97)**	0.03 (0, 16.86)	**0.71 (0.6, 0.85)**	**0.72 (0.59, 0.88)**	0.75 (0.39, 1.49)	0.78 (0.54, 1.11)	0.92 (0.47, 1.76)	0.9 (0.57, 1.39)	0.93 (0.68, 1.3)	0.98 (0.67, 1.44)	16_Placebo_ or_Control	0.93 [0.67; 1.29]	0.95 [0.54; 1.66]	0.86 [0.67; 1.12]	0.88 [0.52; 1.48]	0.82 [0.34; 1.98]	0.86 [0.67; 1.12]	0.72 [0.33; 1.58]	0.44 [0.12; 1.61]
**0 (0, 0.69)**	0 (0, 4.55)	**0.44 (0.22, 0.91)**	0.19 (0.01, 2.11)	0.47 (0.22, 1.12)	0.55 (0.29, 1.03)	0.03 (0, 16.14)	0.66 (0.44, 1.00)	0.67 (0.44, 1.02)	0.69 (0.33, 1.53)	0.72 (0.44, 1.22)	0.86 (0.39, 1.81)	0.84 (0.47, 1.49)	0.86 (0.53, 1.43)	0.92 (0.54, 1.58)	0.93 (0.64, 1.35)	17_Sotagliflozin							
**0 (0, 0.68)**	0 (0, 4.3)	**0.41 (0.17, 0.96)**	0.19 (0.01, 2.18)	0.44 (0.19, 1.14)	0.51 (0.23, 1.17)	0.03 (0, 13.11)	0.62 (0.33, 1.16)	0.64 (0.32, 1.19)	0.65 (0.33, 1.26)	0.68 (0.33, 1.34)	0.79 (0.34, 2.01)	0.79 (0.36, 1.64)	0.81 (0.4, 1.63)	0.86 (0.41, 1.75)	0.87 (0.47, 1.6)	0.93 (0.46, 1.91)	18_Ertugliflozin_ low_dosage						
**0 (0, 0.73)**	0 (0, 4.47)	**0.42 (0.21, 0.85)**	0.19 (0.01, 1.98)	0.46 (0.21, 1.03)	**0.53 (0.28, 0.97)**	0.03 (0, 15.65)	**0.64 (0.44, 0.96)**	**0.65 (0.44, 0.97)**	0.68 (0.33, 1.4)	0.7 (0.44, 1.15)	0.82 (0.39, 1.75)	0.81 (0.47, 1.4)	0.83 (0.54, 1.37)	0.88 (0.53, 1.52)	0.89 (0.64, 1.28)	0.97 (0.59, 1.61)	1.03 (0.51, 2.09)	19_Liraglutide	2.33 [0.09; 50.00]				
**0 (0, 0.64)**	0 (0, 4.04)	**0.41 (0.18, 0.98)**	0.18 (0.01, 1.99)	**0.45 (0.21, 0.98)**	0.51 (0.24, 1.1)	0.03 (0, 15.89)	0.62 (0.33, 1.17)	0.63 (0.33, 1.19)	0.64 (0.27, 1.65)	0.68 (0.34, 1.35)	0.8 (0.33, 1.98)	0.77 (0.37, 1.68)	0.81 (0.41, 1.66)	0.86 (0.42, 1.74)	0.87 (0.48, 1.59)	0.93 (0.47, 1.91)	0.99 (0.43, 2.32)	0.98 (0.49, 1.94)	20_Inject_ semaglutide_ low_dosage				
**0 (0, 0.73)**	0 (0, 4.79)	0.36 (0.12, 1.09)	0.16 (0.01, 1.7)	0.39 (0.13, 1.24)	0.46 (0.17, 1.3)	0.02 (0, 15.83)	0.55 (0.23, 1.32)	0.57 (0.23, 1.34)	0.58 (0.19, 1.83)	0.61 (0.22, 1.54)	0.72 (0.23, 2.03)	0.69 (0.26, 1.81)	0.73 (0.29, 1.83)	0.77 (0.3, 1.93)	0.78 (0.33, 1.82)	0.83 (0.33, 2.12)	0.9 (0.3, 2.61)	0.87 (0.34, 2.18)	0.87 (0.32, 2.5)	21_Efpeglenatide_ high_dosage	1.03 [0.47; 2.24]		
**0 (0, 0.67)**	0 (0, 4.09)	0.34 (0.13, 1.02)	0.15 (0.01, 1.65)	0.38 (0.13, 1.16)	0.44 (0.16, 1.15)	0.02 (0, 12.26)	0.52 (0.23, 1.24)	0.53 (0.23, 1.24)	0.55 (0.19, 1.64)	0.57 (0.24, 1.4)	0.66 (0.25, 2.17)	0.65 (0.26, 1.72)	0.68 (0.29, 1.69)	0.72 (0.3, 1.78)	0.73 (0.33, 1.7)	0.79 (0.32, 1.98)	0.85 (0.3, 2.38)	0.82 (0.35, 2.05)	0.86 (0.3, 2.4)	0.95 (0.43, 2.07)	22_Efpeglenatide_ medium_ dosage		
**0 (0, 0.69)**	0 (0, 3.79)	0.34 (0.12, 1)	0.15 (0.01, 2.03)	0.38 (0.12, 1.1)	0.43 (0.17, 1.11)	0.03 (0, 10.97)	0.52 (0.23, 1.22)	0.53 (0.23, 1.24)	0.54 (0.19, 1.6)	0.57 (0.24, 1.35)	0.69 (0.24, 1.79)	0.66 (0.26, 1.66)	0.68 (0.28, 1.67)	0.72 (0.29, 1.76)	0.73 (0.32, 1.67)	0.78 (0.33, 1.91)	0.85 (0.29, 2.44)	0.82 (0.33, 1.98)	0.84 (0.29, 2.23)	0.96 (0.3, 3.18)	1 (0.3, 2.98)	23_ Bexagliflozin	
**0 (0, 0.13)**	0 (0, 1.17)	**0.06 (0.01, 0.41)**	**0.03 (0, 0.39)**	**0.06 (0.01, 0.4)**	**0.07 (0.01, 0.43)**	**0 (0, 0.98)**	**0.08 (0.01, 0.53)**	**0.09 (0.01, 0.53)**	**0.09 (0.01, 0.59)**	**0.09 (0.01, 0.6)**	**0.11 (0.01, 0.75)**	**0.11 (0.01, 0.66)**	**0.11 (0.01, 0.68)**	**0.12 (0.01, 0.75)**	**0.12 (0.02, 0.75)**	**0.13 (0.02, 0.85)**	**0.14 (0.02, 0.96)**	**0.13 (0.02, 0.83)**	0.14 (0.02, 1.02)	0.15 (0.02, 1.18)	0.16 (0.02, 1.25)	0.16 (0.02, 1.25)	24_Tirzepatide_ high_dosage

**Table 2:** League table of the primary outcome, acute kidney injury, in the subgroup focusing RCTs without definite underlying kidney failure. Data presents as OR [95%CIs]. Pairwise (upper-right portion) and network (lower-left portion) meta-analysis results are presented as estimate effect sizes for the outcome of events of acute kidney injury in the subgroup focusing RCTs without definite underlying kidney failure. Interventions are reported in order of mean ranking of beneficially prophylactic effect on events of acute kidney injury in the subgroup focusing RCTs without definite underlying kidney failure, and outcomes are expressed as odds ratio (OR) (95% confidence intervals) (95% CIs). For the pairwise meta-analyses, OR of less than 1 indicates that the treatment specified in the row got more beneficial effect than that specified in the column. For the network meta-analysis (NMA), OR of less than 1 indicates that the treatment specified in the column got more beneficial effect than that specified in the row. Bold results indicate statistical significance. The gray background indicated the targeted medication. Abbreviation: 95% CIs: 95% confidence intervals; GLP-1 agonist: glucagon-like peptide-1 agonist; NMA: network meta-analysis; OR: odds ratio; RCT: randomized controlled trial; SGLT2 inhibitor: sodium–glucose cotransporter 2 inhibitor.

01_ Lixisenatide																0.33 [0.01; 8.10]							
0.01 (0, 1928395982.52)	02_Efpeglenatide _low_dosage																				0.33 [0.01; 8.28]		
0 (0, 280140903.9)	0.14 (0, 26059247.19)	03_Tirzepatide _low_dosage																					0.43 [0.07; 2.67]
0 (0, 3.96)	0 (0, 4.18)	0 (0, 19.47)	04_Canagliflozin _high_dosage										0.53 [0.22; 1.26]			0.53 [0.28; 0.98]							
0 (0, 15.34)	0 (0, 16.22)	0 (0, 143.29)	3.29 (0.19, 58.06)	05_ Exenatide												0.51 [0.09; 2.93]							
0 (0, 3.63)	0 (0, 5.73)	0 (0, 16.22)	0.91 (0.33, 2.56)	0.26 (0.02, 3.9)	06_Inject_ semaglutide_ medium_dosage						0.67 [0.11; 4.01]					0.53 [0.26; 1.08]			0.41 [0.19; 0.90]				0.67 [0.03; 16.39]
0 (0, 3.5)	0 (0, 4.13)	0 (0, 18.92)	0.89 (0.34, 2.07)	0.26 (0.02, 4.17)	0.96 (0.34, 2.51)	07_Empagliflozin _high_dosage		0.73 [0.40; 1.32]								0.59 [0.33; 1.04]						1.00 [0.06; 16.08]	
0 (0, 2.9)	0 (0, 3.59)	0 (0, 14.68)	0.75 (0.35, 1.46)	0.22 (0.01, 3.38)	0.83 (0.36, 1.78)	0.85 (0.45, 1.62)	08_ Dapagliflozin									0.67 [0.58; 0.78]							
0 (0, 2.83)	0 (0, 3.54)	0 (0, 14.8)	0.72 (0.34, 1.44)	0.21 (0.01, 3.3)	0.79 (0.33, 1.74)	0.82 (0.46, 1.52)	0.96 (0.67, 1.4)	09_Empagliflozin _low_dosage								0.71 [0.57; 0.87]							
0 (0, 2.65)	0 (0, 2.92)	0 (0, 13.96)	0.65 (0.24, 1.67)	0.19 (0.01, 3.5)	0.71 (0.25, 1.98)	0.75 (0.31, 1.87)	0.86 (0.42, 1.88)	0.91 (0.42, 1.97)	10_Ertugliflozin _high_dosage						0.75 [0.41; 1.38]	0.75 [0.40; 1.38]							
0 (0, 2.92)	0 (0, 5.95)	0 (0, 15.31)	0.71 (0.22, 2.1)	0.2 (0.01, 3.62)	0.77 (0.24, 2.4)	0.82 (0.28, 2.16)	0.94 (0.39, 2.29)	0.98 (0.4, 2.35)	1.06 (0.35, 3.27)	11_ Sotagliflozin						0.72 [0.34; 1.53]							
0 (0, 2.41)	0 (0, 3.16)	0 (0, 12.91)	0.64 (0.27, 1.31)	0.19 (0.01, 2.92)	0.7 (0.29, 1.57)	0.74 (0.35, 1.45)	0.86 (0.51, 1.34)	0.9 (0.51, 1.41)	0.99 (0.42, 2.2)	0.9 (0.36, 2.31)	12_Inject_ Semaglutide _high_dosage					0.76 [0.58; 1.00]	3.05 [0.12; 75.53]						
0 (0, 2.1)	0 (0, 2.87)	0 (0, 11.63)	0.56 (0.23, 1.23)	0.16 (0.01, 2.52)	0.61 (0.24, 1.45)	0.65 (0.29, 1.42)	0.75 (0.43, 1.31)	0.78 (0.43, 1.36)	0.85 (0.35, 2.06)	0.79 (0.28, 2.15)	0.87 (0.47, 1.75)	13_ Albiglutide				0.87 [0.59; 1.29]	3.04 [0.12; 74.78]						
0 (0, 2.33)	0 (0, 2.36)	0 (0, 10.38)	0.54 (0.23, 1.31)	0.17 (0.01, 2.7)	0.61 (0.21, 1.69)	0.63 (0.24, 1.63)	0.74 (0.33, 1.6)	0.76 (0.34, 1.69)	0.83 (0.29, 2.37)	0.78 (0.22, 2.45)	0.86 (0.38, 2.08)	0.98 (0.41, 2.48)	14_Canagliflozin _low_dosage			1.02 [0.50; 2.08]							
0 (0, 1.97)	0 (0, 2.81)	0 (0, 10.76)	0.54 (0.24, 1.2)	0.16 (0.01, 2.55)	0.6 (0.24, 1.54)	0.63 (0.3, 1.37)	0.73 (0.45, 1.28)	0.76 (0.46, 1.34)	0.84 (0.36, 2)	0.76 (0.29, 2.18)	0.86 (0.49, 1.74)	0.97 (0.5, 2.01)	0.99 (0.42, 2.47)	15_ Dulaglutide		0.97 [0.70; 1.35]	0.33 [0.01; 8.22]			0.32 [0.01; 7.92]	0.34 [0.01; 8.49]		0.63 [0.03; 15.80]
0 (0, 1.97)	0 (0, 2.27)	0 (0, 10.95)	0.49 (0.19, 1.22)	0.14 (0.01, 2.49)	0.54 (0.21, 1.42)	0.56 (0.24, 1.36)	0.66 (0.33, 1.33)	0.69 (0.34, 1.41)	0.76 (0.37, 1.47)	0.7 (0.22, 2.08)	0.77 (0.36, 1.71)	0.88 (0.39, 2.02)	0.91 (0.33, 2.42)	0.91 (0.4, 1.99)	16_Ertugliflozin _low_dosage	1.00 [0.57; 1.76]							
0 (0, 1.88)	0 (0, 2.47)	0 (0, 9.99)	**0.5 (0.25, 0.94)**	0.15 (0.01, 2.29)	0.56 (0.25, 1.15)	0.58 (0.32, 1.04)	0.67 (0.53, 0.85)	0.71 (0.52, 0.92)	0.78 (0.38, 1.59)	0.72 (0.31, 1.69)	0.78 (0.54, 1.25)	0.9 (0.54, 1.5)	0.92 (0.43, 1.94)	0.93 (0.56, 1.41)	1.01 (0.53, 1.95)	17_Placebo_ or_Control	0.86 [0.67; 1.12]	0.80 [0.25; 2.55]	0.88 [0.52; 1.48]	0.82 [0.34; 1.98]	0.84 [0.35; 2.00]	0.33 [0.02; 6.25]	0.44 [0.12; 1.61]
0 (0, 1.77)	0 (0, 2.33)	0 (0, 9.44)	**0.46 (0.21, 0.99)**	0.13 (0.01, 2.17)	0.49 (0.21, 1.24)	0.52 (0.26, 1.14)	0.6 (0.4, 1.05)	0.63 (0.4, 1.11)	0.69 (0.32, 1.62)	0.64 (0.27, 1.75)	0.7 (0.42, 1.43)	0.81 (0.44, 1.7)	0.83 (0.36, 2)	0.83 (0.47, 1.56)	0.91 (0.44, 2.08)	0.9 (0.63, 1.47)	18_ Liraglutide		2.33 [0.09; 50.00]				
0 (0, 1.63)	0 (0, 2.45)	0 (0, 8.53)	0.39 (0.08, 1.46)	0.11 (0.01, 2.45)	0.41 (0.1, 1.6)	0.43 (0.1, 1.72)	0.51 (0.14, 1.64)	0.53 (0.14, 1.78)	0.6 (0.13, 2.28)	0.52 (0.12, 2.5)	0.59 (0.16, 2.11)	0.68 (0.17, 2.4)	0.71 (0.16, 2.65)	0.7 (0.17, 2.41)	0.77 (0.18, 2.93)	0.76 (0.21, 2.44)	0.84 (0.21, 2.83)	19_ Bexagliflozin					
0 (0, 1.52)	0 (0, 3.6)	0 (0, 8.53)	0.42 (0.17, 1.22)	0.12 (0.01, 1.85)	**0.47 (0.21, 0.97)**	0.49 (0.2, 1.11)	0.57 (0.29, 1.1)	0.59 (0.3, 1.17)	0.65 (0.25, 1.86)	0.59 (0.21, 1.75)	0.67 (0.33, 1.45)	0.76 (0.34, 1.66)	0.76 (0.29, 2.52)	0.78 (0.34, 1.59)	0.85 (0.35, 2.49)	0.84 (0.45, 1.58)	0.94 (0.42, 1.85)	1.11 (0.31, 4.6)	20_Inject_ semaglutide _low_dosage				
0 (0, 1.84)	0 (0, 1.44)	0 (0, 10.71)	0.39 (0.12, 1.14)	0.12 (0.01, 1.94)	0.43 (0.12, 1.39)	0.44 (0.14, 1.34)	0.52 (0.18, 1.37)	0.55 (0.18, 1.45)	0.62 (0.17, 1.79)	0.55 (0.15, 1.89)	0.61 (0.2, 1.69)	0.68 (0.22, 2.04)	0.72 (0.19, 2.24)	0.71 (0.22, 2)	0.8 (0.23, 2.33)	0.77 (0.27, 2)	0.85 (0.27, 2.29)	1 (0.21, 4.76)	0.91 (0.29, 2.81)	21_Efpeglenatide _high_dosage	1.03 [0.47; 2.24]		
0 (0, 1.98)	0 (0, 1.44)	0 (0, 11.84)	0.37 (0.11, 1.08)	0.11 (0.01, 2.02)	0.4 (0.11, 1.35)	0.42 (0.13, 1.23)	0.49 (0.16, 1.28)	0.52 (0.17, 1.32)	0.58 (0.16, 1.8)	0.52 (0.13, 1.83)	0.59 (0.18, 1.57)	0.64 (0.21, 1.93)	0.68 (0.19, 2.18)	0.68 (0.19, 1.94)	0.76 (0.22, 2.25)	0.73 (0.24, 1.84)	0.82 (0.25, 2.1)	0.96 (0.21, 4.43)	0.86 (0.27, 2.64)	0.96 (0.41, 2.19)	22_Efpeglenatide_ medium_dosage		
0 (0, 1.19)	**0 (0, 0.8)**	0 (0, 6.87)	0.19 (0.01, 1.74)	0.05 (0, 1.67)	0.22 (0.01, 1.93)	0.23 (0.01, 1.68)	0.26 (0.01, 2.07)	0.27 (0.01, 2.16)	0.3 (0.01, 2.76)	0.27 (0.01, 2.48)	0.31 (0.01, 2.54)	0.35 (0.01, 3.12)	0.34 (0.01, 3.39)	0.37 (0.01, 3.18)	0.39 (0.01, 3.5)	0.39 (0.01, 3.05)	0.44 (0.02, 3.46)	0.52 (0.02, 5.95)	0.45 (0.02, 3.71)	0.49 (0.02, 5.52)	0.51 (0.02, 5.2)	23_Oral _semaglutide	
**0 (0, 0.22)**	**0 (0, 0.32)**	**0 (0, 0.62)**	**0.05 (0, 0.35)**	**0.01 (0, 0.35)**	**0.05 (0, 0.37)**	**0.06 (0, 0.42)**	**0.06 (0, 0.42)**	**0.07 (0, 0.45)**	**0.07 (0, 0.54)**	**0.06 (0, 0.53)**	**0.07 (0.01, 0.52)**	**0.08 (0.01, 0.58)**	**0.09 (0.01, 0.63)**	**0.09 (0.01, 0.6)**	**0.09 (0.01, 0.72)**	**0.09 (0.01, 0.61)**	**0.1 (0.01, 0.69)**	0.12 (0.01, 1.1)	**0.1 (0.01, 0.76)**	0.12 (0.01, 1.01)	0.12 (0.01, 1.11)	0.25 (0.01, 8.37)	24_Tirzepatide _high_dosage

## Data Availability

Dataset available on request from the authors (The raw data supporting the conclusions of this article will be made available by the authors on request).

## Supplementary Materials

The following supporting information can be downloaded at: https://www.mdpi.com/article/10.3390/ijms27094137/s1.
