# Supplementary material for: Differential Acute Kidney Injury Profiles of GLP-1RAs and SGLT2is: A Network Meta-Analysis
Source: Int J Mol Sci. 2026 May 6;27(9):4137. doi: 10.3390/ijms27094137 (PMC13164542; doi:10.3390/ijms27094137)
Supplement: Supplementary file 1 [file ijms-27-04137-s001.zip › ijms-4232794-supplementary.pdf]

List of content in supplement materials

Differential Acute Kidney Injury Profiles of GLP-1RAs and SGLT2is

A Network Meta-Analysis

Chih-Sung Liang, et al.

Figure S1	Network structure of NMA of safety profile: drop-out rate
Figure S2	Forest plot of NMA of safety profile: drop-out rate
Figure S3	(A) Individual study result of primary outcome: overall acute kidney injury/acute renal failure
	(B) Individual study result of primary outcome: acute kidney injury/acute renal failure in the subgroup focusing RCTs without definite underlying kidney failure
	(C) Individual study result of safety profile: drop-out rate
Figure S4	(A) Bayesian-based Litmus Rank-O-Gram rank plot of primary outcome: overall acute kidney injury/acute renal failure
	(B) Bayesian-based radial surface under the cumulative ranking of primary outcome: overall acute kidney injury/acute renal failure
	(C) Bayesian-based Litmus Rank-O-Gram rank plot of primary outcome: acute kidney injury/acute renal failure in the subgroup focusing RCTs without definite underlying kidney failure
	(D) Bayesian-based radial surface under the cumulative ranking of primary outcome: acute kidney injury/acute renal failure in the subgroup focusing RCTs without definite underlying kidney failure
	(E) Bayesian-based Litmus Rank-O-Gram rank plot of safety profile: drop-out rate
	(F) Bayesian-based radial surface under the cumulative ranking of safety profile: drop-out rate
Figure S5	(A) Bayesian-based residual deviance NMA/UME model of primary outcome: overall acute kidney injury/acute renal failure
	(B) Bayesian-based per-arm residual deviance of primary outcome: overall acute kidney injury/acute renal failure
	(C) Bayesian-based leverage plot of primary outcome: overall acute kidney injury/acute renal failure
	(D) Bayesian-based residual deviance NMA/UME model of primary outcome: acute kidney injury/acute renal failure in the subgroup focusing RCTs without definite underlying kidney failure

	(E) Bayesian-based per-arm residual deviance of primary outcome: acute kidney injury/acute renal failure in the subgroup focusing RCTs without definite underlying kidney failure
	(F) Bayesian-based leverage plot of primary outcome: acute kidney injury/acute renal failure in the subgroup focusing RCTs without definite underlying kidney failure
	(G) Bayesian-based residual deviance NMA/UME model of safety profile: drop-out rate
	(H) Bayesian-based per-arm residual deviance of safety profile: drop-out rate
	(I) Bayesian-based leverage plot of safety profile: drop-out rate
Figure S6	(A) Overview of risk of bias
	(B) Detailed risk of bias in each study
Table S1	PRISMA 2020 checklist of the current network meta-analysis
Table S2	Keyword used in each database and search results
Table S3	Excluded studies and reason
Table S4	Characteristics of the included studies
Table S5	League table of NMA of safety profile: drop-out rate
Table S6	(A) SUCRA (Surface under the cumulative ranking) of primary outcome: overall acute kidney injury/acute renal failure
	(B) SUCRA (Surface under the cumulative ranking) of primary outcome: acute kidney injury/acute renal failure in the subgroup focusing RCTs without definite underlying kidney failure
	(C) SUCRA (Surface under the cumulative ranking) of safety profile: drop-out rate
Table S7	(A) Inconsistency within the network meta-analysis of primary outcome: overall acute kidney injury/acute renal failure
	(B) Inconsistency within the network meta-analysis of primary outcome: acute kidney injury/acute renal failure in the subgroup focusing RCTs without definite underlying kidney failure
	(C) Inconsistency within the network meta-analysis of safety profile: drop-out rate
Table S8	(A) GRADE of primary outcome: overall acute kidney injury/acute renal failure
	(B) GRADE of primary outcome: acute kidney injury/acute renal failure in the subgroup focusing RCTs without definite underlying kidney failure
	(C) GRADE of safety profile: drop-out rate
Table S9	Distribution of key clinical and design-related effect modifiers across treatment nodes to assess the plausibility of transitivity

Figure S1 network structure of NMA of safety profile: drop-out rate

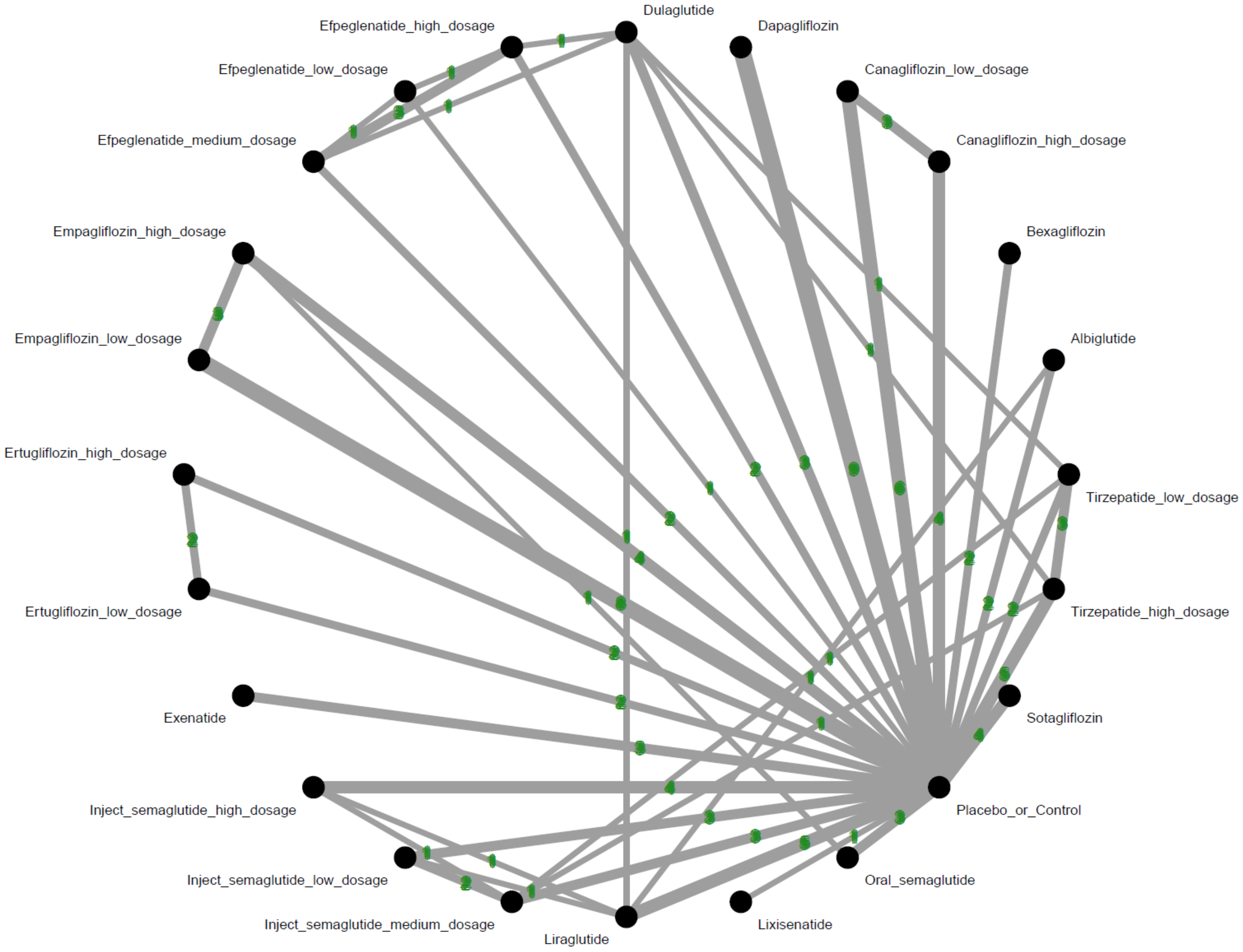

Figure legend of Figure S1

The structure of the network meta-analysis. The lines between nodes represent direct comparisons from various trials, with the numbers over the lines indicating the number of trials providing these comparisons for each specific treatment. The thickness of the lines corresponds to the number of trials linked to the network.

Abbreviation for Figure S1:

95%CIs: 95% confidence intervals; GLP-1 agonist: glucagon-like peptide-1 agonist; NMA: network meta-analysis; OR: odds ratio; RCT: randomized controlled trial; SGLT2 inhibitor: sodium–glucose cotransporter 2 inhibitor

Figure S2 forest plot of NMA of safety profile: drop-out rate

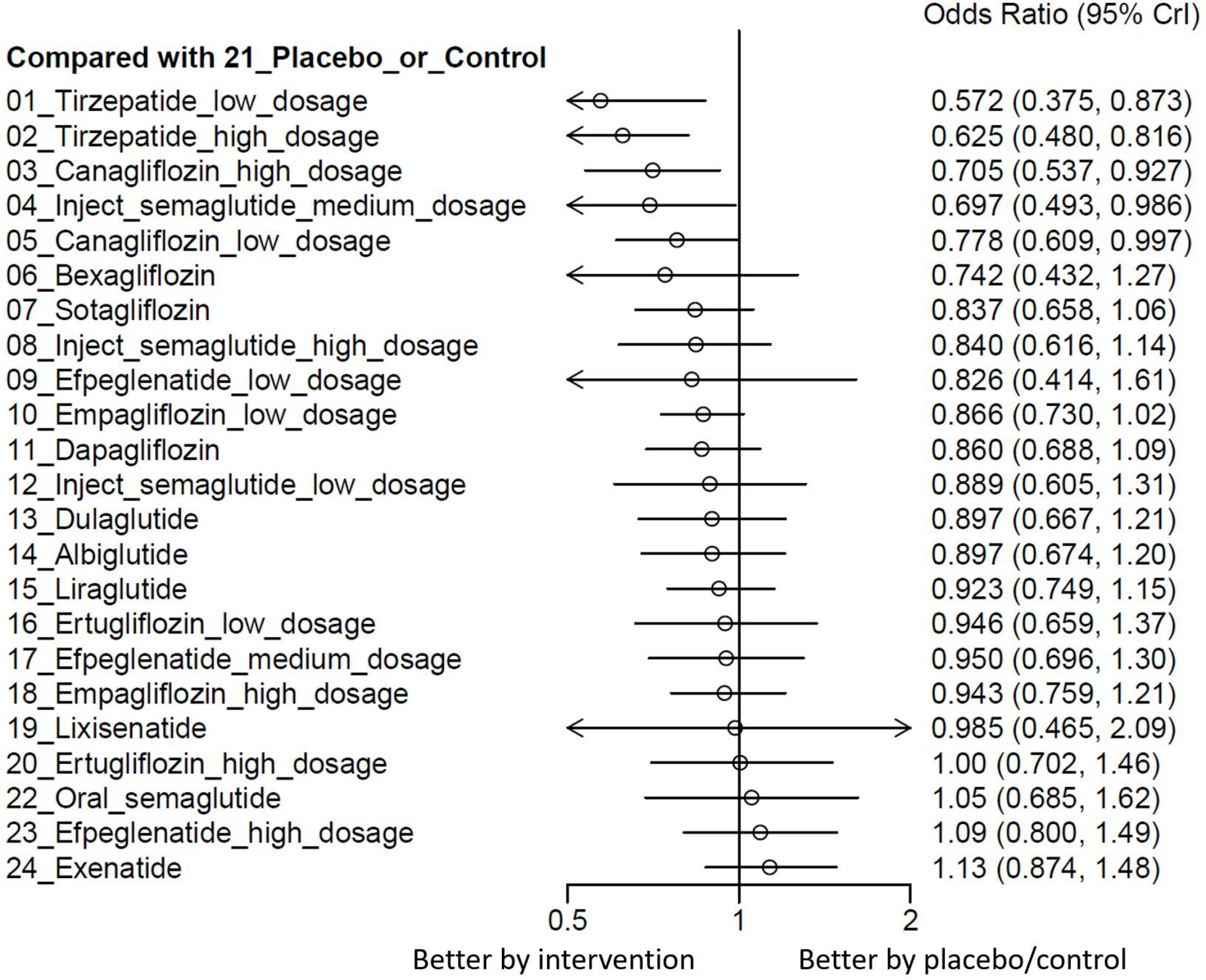

Abbreviation for Figure S2:

95%CIs: 95% confidence intervals; GLP-1 agonist: glucagon-like peptide-1 agonist; NMA: network meta-analysis; OR: odds ratio; RCT: randomized controlled trial; SGLT2 inhibitor: sodium–glucose cotransporter 2 inhibitor

Figure S3A Individual study result of primary outcome: overall acute kidney injury/acute renal failure

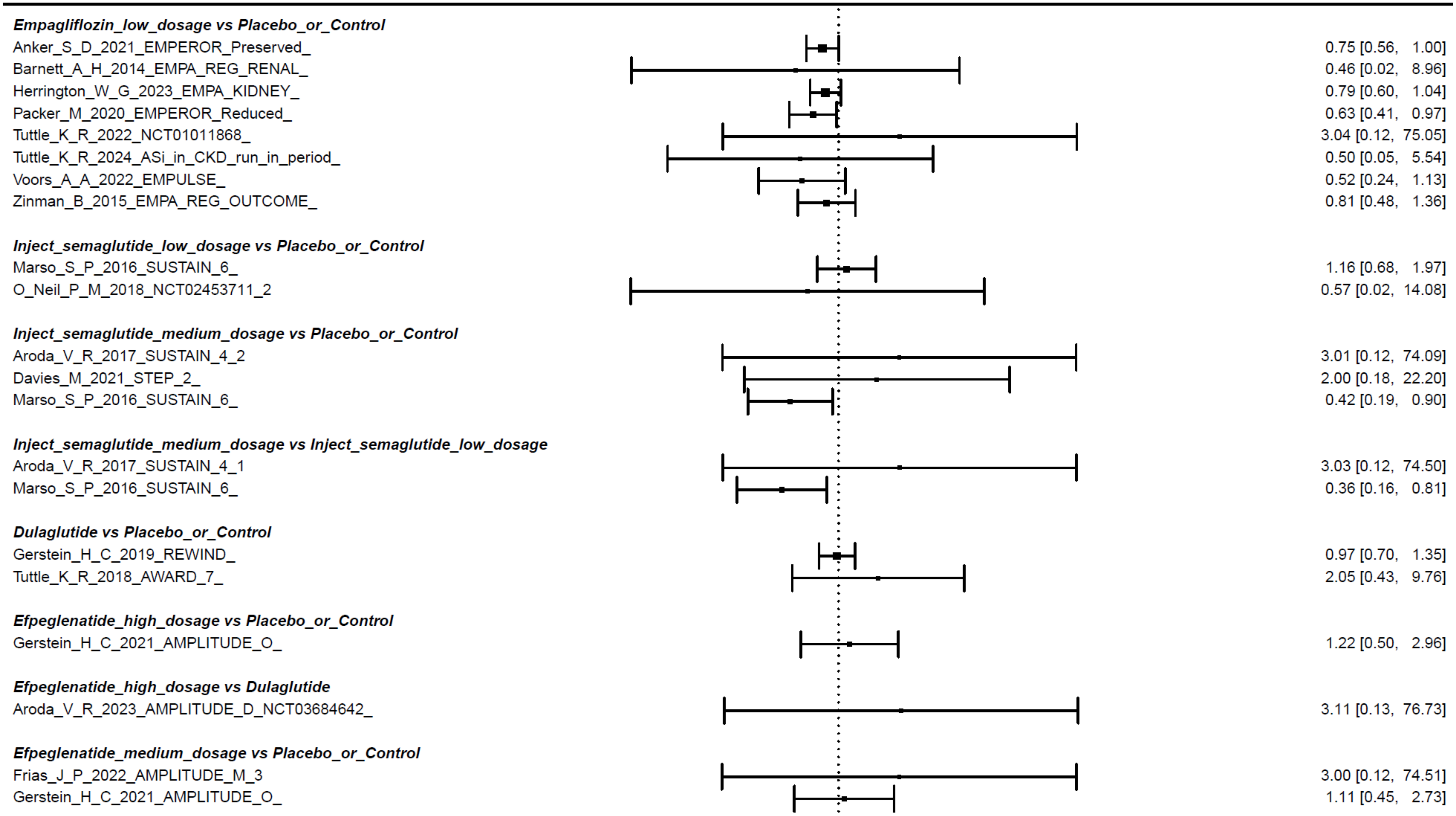

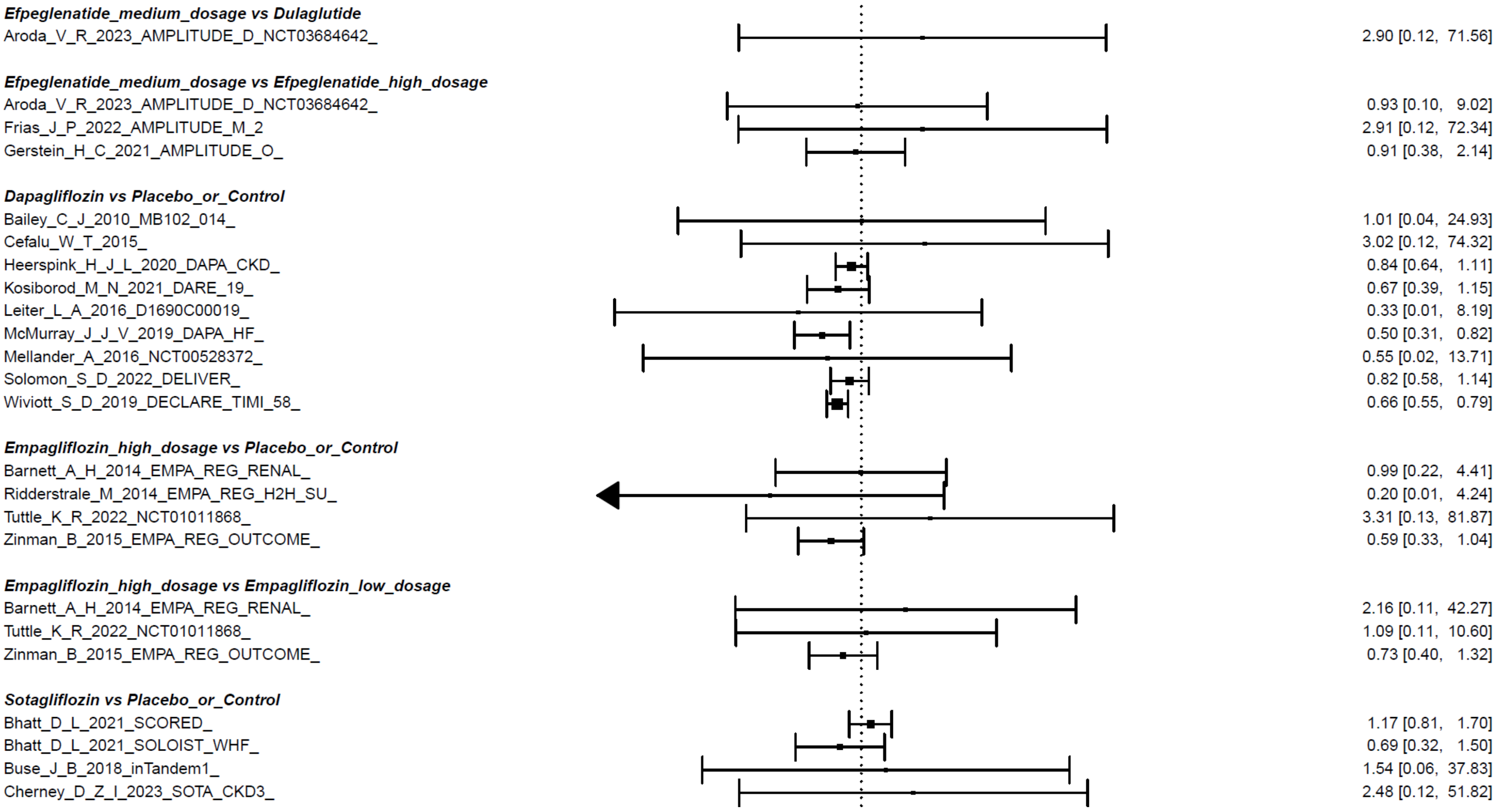
Efpeglenatide_medium_dosage vs Dulaglutide
Aroda_V_R_2023_AMPLITUDE_D_NCT03684642_ 2.90 [0.12, 71.56]
Efpeglenatide_medium_dosage vs Efpeglenatide_high_dosage
Aroda_V_R_2023_AMPLITUDE_D_NCT03684642_ 0.93 [0.10, 9.02]
Frias_J_P_2022_AMPLITUDE_M_2 2.91 [0.12, 72.34]
Gerstein_H_C_2021_AMPLITUDE_O_ 0.91 [0.38, 2.14]
Dapagliflozin vs Placebo_or_Control
Bailey_C_J_2010_MB102_014_ 1.01 [0.04, 24.93]
Cefalu_W_T_2015_ 3.02 [0.12, 74.32]
Heerspink_H_J_L_2020_DAPA_CKD_ 0.84 [0.64, 1.11]
Kosiborod_M_N_2021_DARE_19_ 0.67 [0.39, 1.15]
Leiter_L_A_2016_D1690C00019_ 0.33 [0.01, 8.19]
McMurray_J_J_V_2019_DAPA_HF_ 0.50 [0.31, 0.82]
Mellander_A_2016_NCT00528372_ 0.55 [0.02, 13.71]
Solomon_S_D_2022_DELIVER_ 0.82 [0.58, 1.14]
Wiviott_S_D_2019_DECLARE_TIMI_58_ 0.66 [0.55, 0.79]
Empagliflozin_high_dosage vs Placebo_or_Control
Barnett_A_H_2014_EMPA_REG_RENAL_ 0.99 [0.22, 4.41]
Ridderstrale_M_2014_EMPA_REG_H2H_SU_ 0.20 [0.01, 4.24]
Tuttle_K_R_2022_NCT01011868_ 3.31 [0.13, 81.87]
Zinman_B_2015_EMPA_REG_OUTCOME_ 0.59 [0.33, 1.04]
Empagliflozin_high_dosage vs Empagliflozin_low_dosage
Barnett_A_H_2014_EMPA_REG_RENAL_ 2.16 [0.11, 42.27]
Tuttle_K_R_2022_NCT01011868_ 1.09 [0.11, 10.60]
Zinman_B_2015_EMPA_REG_OUTCOME_ 0.73 [0.40, 1.32]
Sotagliflozin vs Placebo_or_Control
Bhatt_D_L_2021_SCORED_ 1.17 [0.81, 1.70]
Bhatt_D_L_2021_SOLOIST_WHF_ 0.69 [0.32, 1.50]
Buse_J_B_2018_inTandem1_ 1.54 [0.06, 37.83]
Cherney_D_Z_I_2023_SOTA_CKD3_ 2.48 [0.12, 51.82]

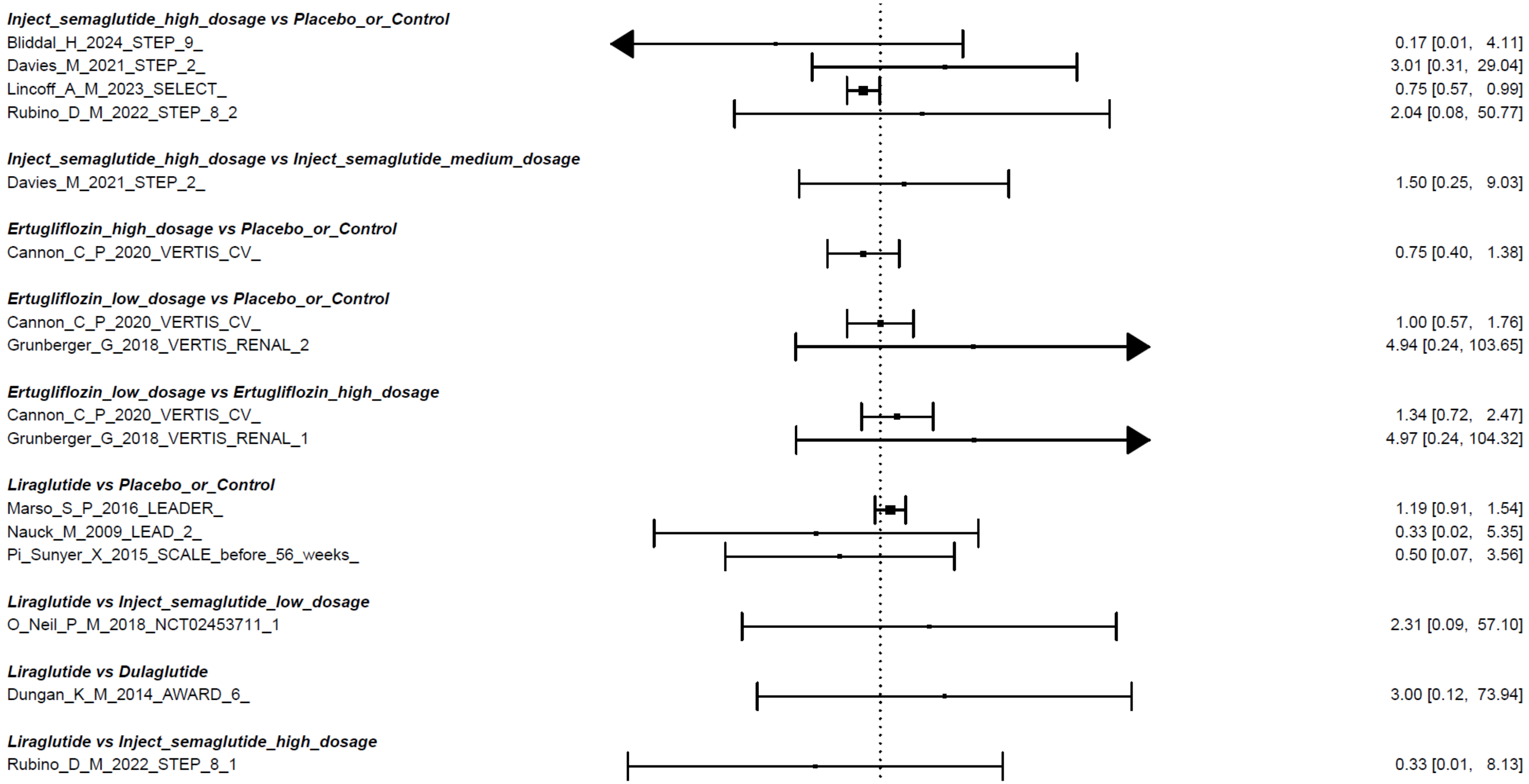
Inject_semaglutide_high_dosage vs Placebo_or_Control
Bliddal_H_2024_STEP_9_ 0.17 [0.01, 4.11]
Davies_M_2021_STEP_2_ 3.01 [0.31, 29.04]
Lincoff_A_M_2023_SELECT_ 0.75 [0.57, 0.99]
Rubino_D_M_2022_STEP_8_2 2.04 [0.08, 50.77]
Inject_semaglutide_high_dosage vs Inject_semaglutide_medium_dosage
Davies_M_2021_STEP_2_ 1.50 [0.25, 9.03]
Ertugliflozin_high_dosage vs Placebo_or_Control
Cannon_C_P_2020_VERTIS_CV_ 0.75 [0.40, 1.38]
Ertugliflozin_low_dosage vs Placebo_or_Control
Cannon_C_P_2020_VERTIS_CV_ 1.00 [0.57, 1.76]
Grunberger_G_2018_VERTIS_RENAL_2 4.94 [0.24, 103.65]
Ertugliflozin_low_dosage vs Ertugliflozin_high_dosage
Cannon_C_P_2020_VERTIS_CV_ 1.34 [0.72, 2.47]
Grunberger_G_2018_VERTIS_RENAL_1 4.97 [0.24, 104.32]
Liraglutide vs Placebo_or_Control
Marso_S_P_2016_LEADER_ 1.19 [0.91, 1.54]
Nauck_M_2009_LEAD_2_ 0.33 [0.02, 5.35]
Pi_Sunyer_X_2015_SCALE_before_56_weeks_ 0.50 [0.07, 3.56]
Liraglutide vs Inject_semaglutide_low_dosage
O_Neil_P_M_2018_NCT02453711_1 2.31 [0.09, 57.10]
Liraglutide vs Dulaglutide
Dungan_K_M_2014_AWARD_6_ 3.00 [0.12, 73.94]
Liraglutide vs Inject_semaglutide_high_dosage
Rubino_D_M_2022_STEP_8_1 0.33 [0.01, 8.13]

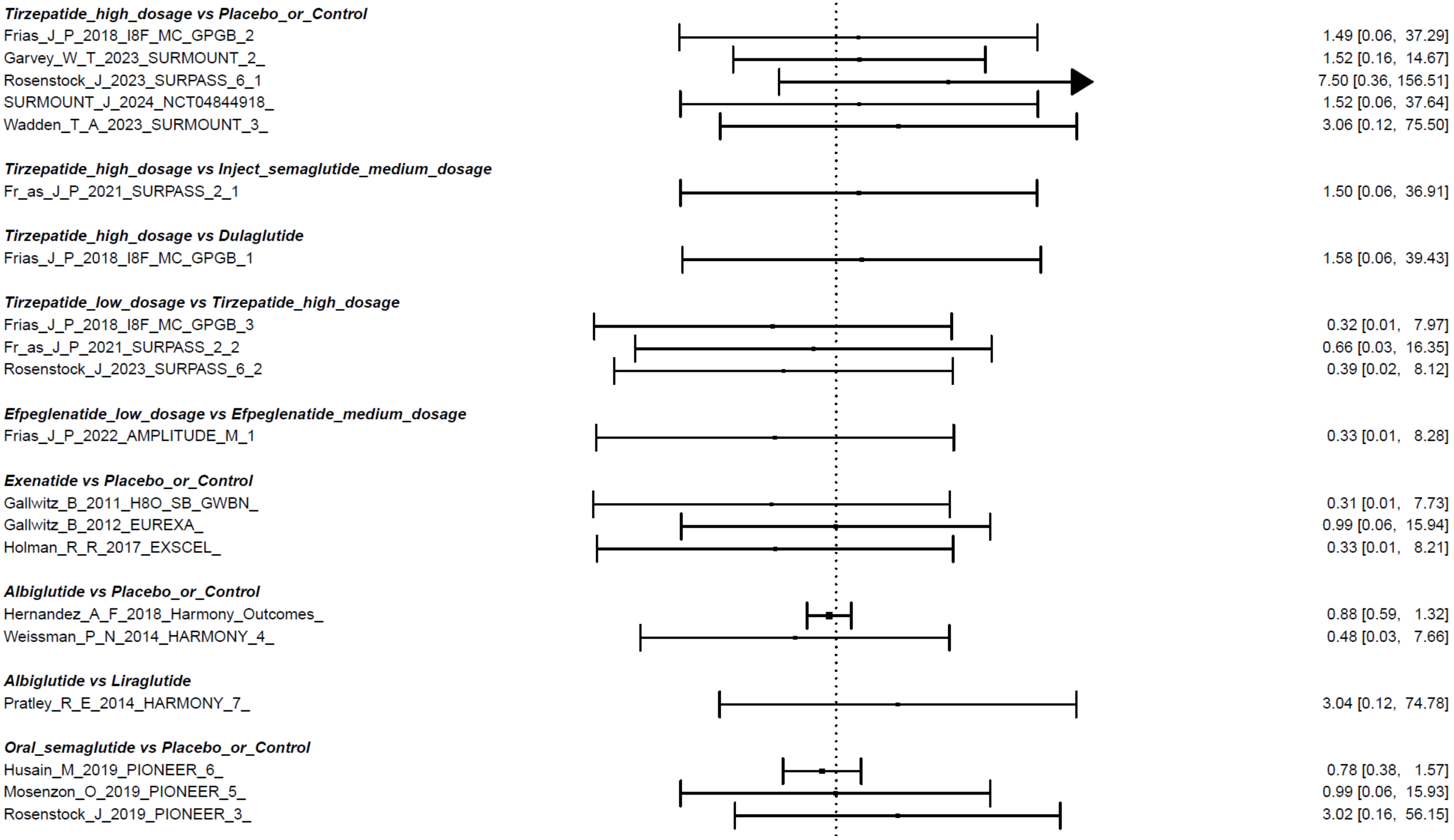
Tirzepatide_high_dosage vs Placebo_or_Control
Frias_J_P_2018_I8F_MC_GPGB_2 1.49 [0.06, 37.29]
Garvey_W_T_2023_SURMOUNT_2_ 1.52 [0.16, 14.67]
Rosenstock_J_2023_SURPASS_6_1 7.50 [0.36, 156.51]
SURMOUNT_J_2024_NCT04844918_ 1.52 [0.06, 37.64]
Wadden_T_A_2023_SURMOUNT_3_ 3.06 [0.12, 75.50]
Tirzepatide_high_dosage vs Inject_semaglutide_medium_dosage
Fr_as_J_P_2021_SURPASS_2_1 1.50 [0.06, 36.91]
Tirzepatide_high_dosage vs Dulaglutide
Frias_J_P_2018_I8F_MC_GPGB_1 1.58 [0.06, 39.43]
Tirzepatide_low_dosage vs Tirzepatide_high_dosage
Frias_J_P_2018_I8F_MC_GPGB_3 0.32 [0.01, 7.97]
Fr_as_J_P_2021_SURPASS_2_2 0.66 [0.03, 16.35]
Rosenstock_J_2023_SURPASS_6_2 0.39 [0.02, 8.12]
Efpeglenatide_low_dosage vs Efpeglenatide_medium_dosage
Frias_J_P_2022_AMPLITUDE_M_1 0.33 [0.01, 8.28]
Exenatide vs Placebo_or_Control
Gallwitz_B_2011_H8O_SB_GWBN_ 0.31 [0.01, 7.73]
Gallwitz_B_2012_EUREXA_ 0.99 [0.06, 15.94]
Holman_R_R_2017_EXSCEL_ 0.33 [0.01, 8.21]
Albiglutide vs Placebo_or_Control
Hernandez_A_F_2018_Harmony_Outcomes_ 0.88 [0.59, 1.32]
Weissman_P_N_2014_HARMONY_4_ 0.48 [0.03, 7.66]
Albiglutide vs Liraglutide
Pratley_R_E_2014_HARMONY_7_ 3.04 [0.12, 74.78]
Oral_semaglutide vs Placebo_or_Control
Husain_M_2019_PIONEER_6_ 0.78 [0.38, 1.57]
Mosenzon_O_2019_PIONEER_5_ 0.99 [0.06, 15.93]
Rosenstock_J_2019_PIONEER_3_ 3.02 [0.16, 56.15]

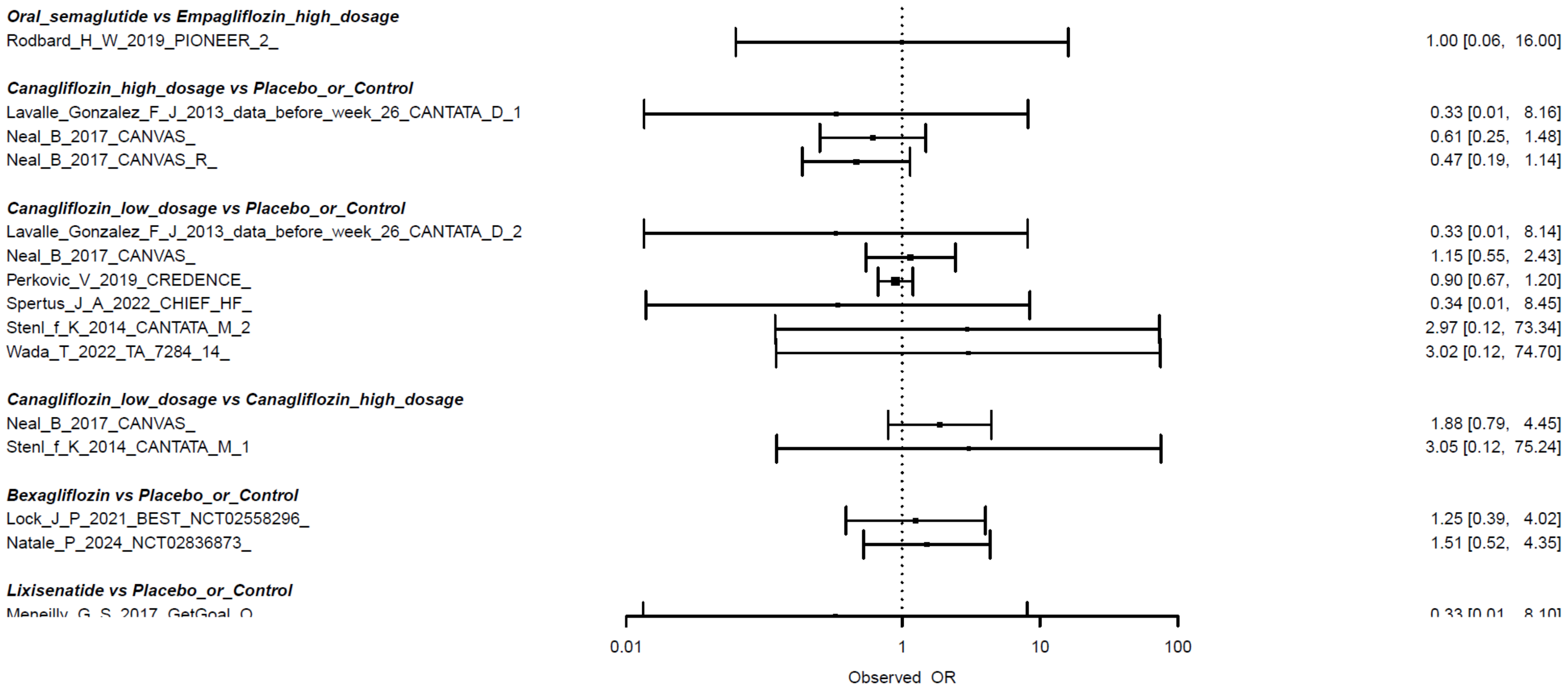

Oral_semaglutide vs Empagliflozin_high_dosage
Rodbard_H_W_2019_PIONEER_2_
1.00 [0.06, 16.00]
Canagliflozin_high_dosage vs Placebo_or_Control
Lavalle_Gonzalez_F_J_2013_data_before_week_26_CANTATA_D_1
0.33 [0.01, 8.16]
Neal_B_2017_CANVAS_
0.61 [0.25, 1.48]
Neal_B_2017_CANVAS_R_
0.47 [0.19, 1.14]
Canagliflozin_low_dosage vs Placebo_or_Control
Lavalle_Gonzalez_F_J_2013_data_before_week_26_CANTATA_D_2
0.33 [0.01, 8.14]
Neal_B_2017_CANVAS_
1.15 [0.55, 2.43]
Perkovic_V_2019_CREDENCE_
0.90 [0.67, 1.20]
Spertus_J_A_2022_CHIEF_HF_
0.34 [0.01, 8.45]
Stenl_f_K_2014_CANTATA_M_2
2.97 [0.12, 73.34]
Wada_T_2022_TA_7284_14_
3.02 [0.12, 74.70]
Canagliflozin_low_dosage vs Canagliflozin_high_dosage
Neal_B_2017_CANVAS_
1.88 [0.79, 4.45]
Stenl_f_K_2014_CANTATA_M_1
3.05 [0.12, 75.24]
Bexagliflozin vs Placebo_or_Control
Lock_J_P_2021_BEST_NCT02558296_
1.25 [0.39, 4.02]
Natale_P_2024_NCT02836873_
1.51 [0.52, 4.35]
Lixisenatide vs Placebo_or_Control
Meneilly_G_S_2017_GetGoal_O
0.33 [0.01, 8.10]
0.01
1
10
100
Observed OR

Figure S3B Individual study result of primary outcome: acute kidney injury/acute renal failure in the subgroup focusing RCTs without definite underlying kidney failure

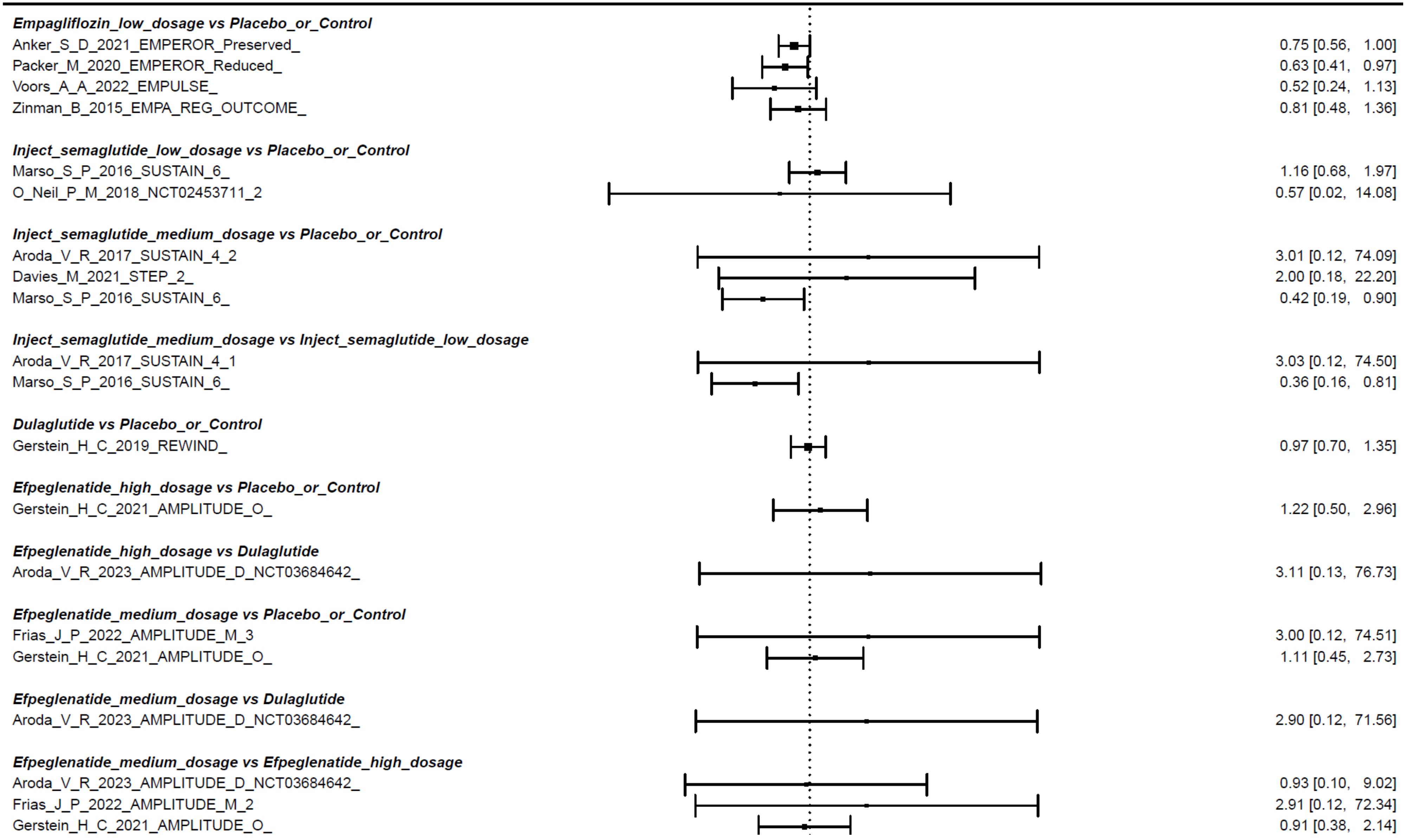

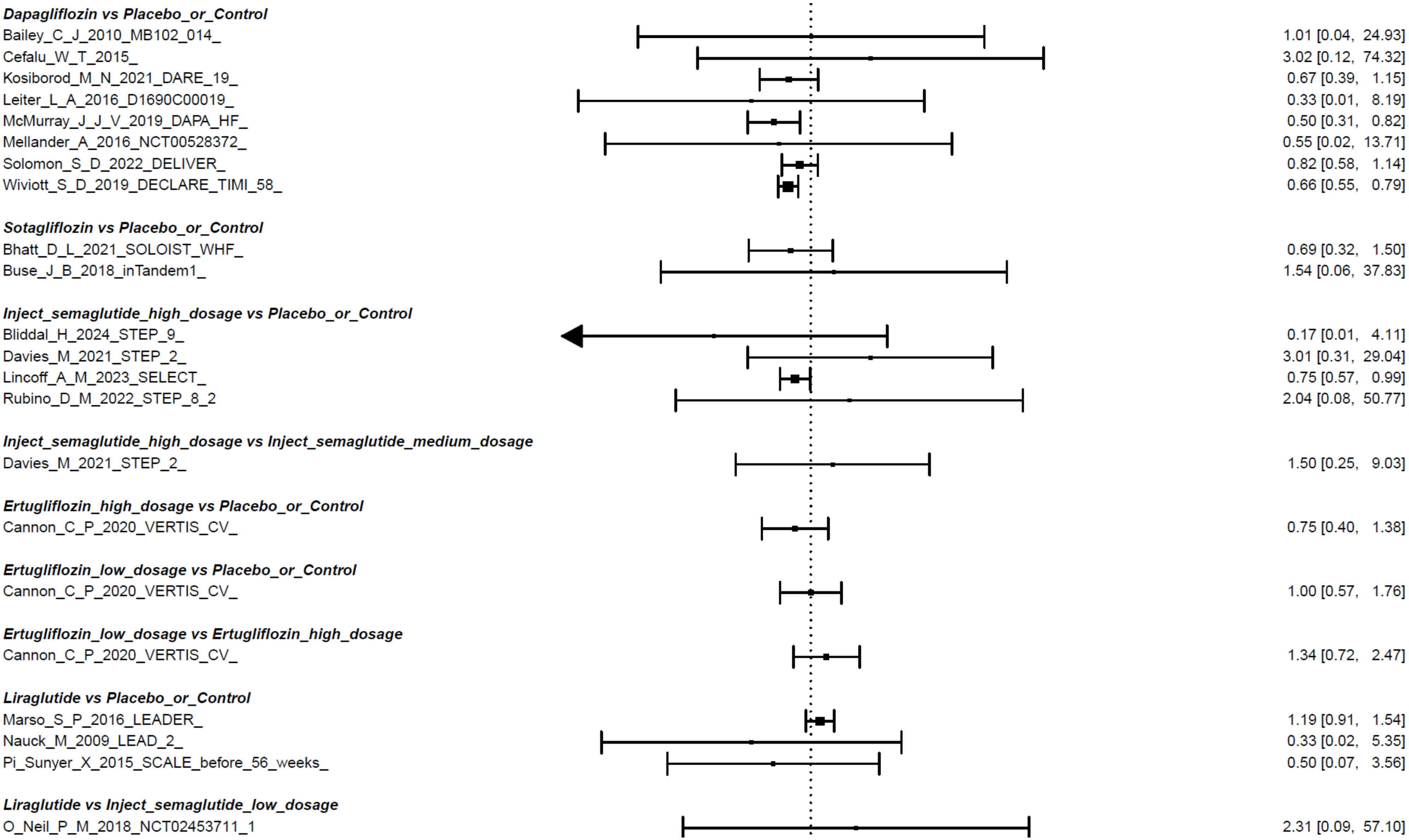

Dapagliflozin vs Placebo_or_Control
Bailey_C_J_2010_MB102_014_ 1.01 [0.04, 24.93]
Cefalu_W_T_2015_ 3.02 [0.12, 74.32]
Kosiborod_M_N_2021_DARE_19_ 0.67 [0.39, 1.15]
Leiter_L_A_2016_D1690C00019_ 0.33 [0.01, 8.19]
McMurray_J_J_V_2019_DAPA_HF_ 0.50 [0.31, 0.82]
Mellander_A_2016_NCT00528372_ 0.55 [0.02, 13.71]
Solomon_S_D_2022_DELIVER_ 0.82 [0.58, 1.14]
Wiviott_S_D_2019_DECLARE_TIMI_58_ 0.66 [0.55, 0.79]
Sotagliflozin vs Placebo_or_Control
Bhatt_D_L_2021_SOLOIST_WHF_ 0.69 [0.32, 1.50]
Buse_J_B_2018_inTandem1_ 1.54 [0.06, 37.83]
Inject_semaglutide_high_dosage vs Placebo_or_Control
Bliddal_H_2024_STEP_9_ 0.17 [0.01, 4.11]
Davies_M_2021_STEP_2_ 3.01 [0.31, 29.04]
Lincoff_A_M_2023_SELECT_ 0.75 [0.57, 0.99]
Rubino_D_M_2022_STEP_8_2 2.04 [0.08, 50.77]
Inject_semaglutide_high_dosage vs Inject_semaglutide_medium_dosage
Davies_M_2021_STEP_2_ 1.50 [0.25, 9.03]
Ertugliflozin_high_dosage vs Placebo_or_Control
Cannon_C_P_2020_VERTIS_CV_ 0.75 [0.40, 1.38]
Ertugliflozin_low_dosage vs Placebo_or_Control
Cannon_C_P_2020_VERTIS_CV_ 1.00 [0.57, 1.76]
Ertugliflozin_low_dosage vs Ertugliflozin_high_dosage
Cannon_C_P_2020_VERTIS_CV_ 1.34 [0.72, 2.47]
Liraglutide vs Placebo_or_Control
Marso_S_P_2016_LEADER_ 1.19 [0.91, 1.54]
Nauck_M_2009_LEAD_2_ 0.33 [0.02, 5.35]
Pi_Sunyer_X_2015_SCALE_before_56_weeks_ 0.50 [0.07, 3.56]
Liraglutide vs Inject_semaglutide_low_dosage
O_Neil_P_M_2018_NCT02453711_1 2.31 [0.09, 57.10]

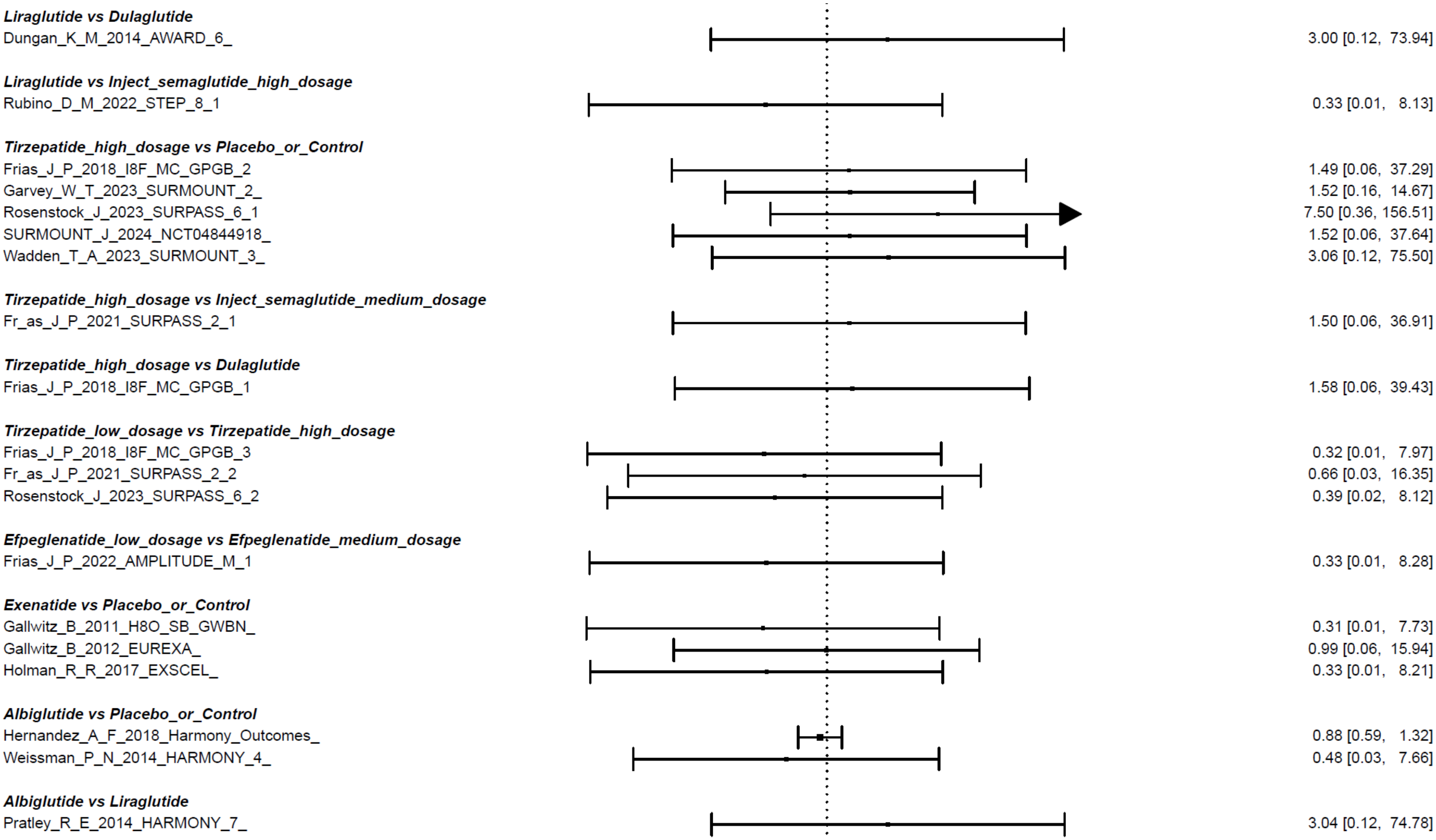

Liraglutide vs Dulaglutide
Dungan_K_M_2014_AWARD_6_
3.00 [0.12, 73.94]
Liraglutide vs Inject_semaglutide_high_dosage
Rubino_D_M_2022_STEP_8_1
0.33 [0.01, 8.13]
Tirzepatide_high_dosage vs Placebo_or_Control
Frias_J_P_2018_I8F_MC_GPGB_2
1.49 [0.06, 37.29]
Garvey_W_T_2023_SURMOUNT_2_
1.52 [0.16, 14.67]
Rosenstock_J_2023_SURPASS_6_1
7.50 [0.36, 156.51]
SURMOUNT_J_2024_NCT04844918_
1.52 [0.06, 37.64]
Wadden_T_A_2023_SURMOUNT_3_
3.06 [0.12, 75.50]
Tirzepatide_high_dosage vs Inject_semaglutide_medium_dosage
Fr_as_J_P_2021_SURPASS_2_1
1.50 [0.06, 36.91]
Tirzepatide_high_dosage vs Dulaglutide
Frias_J_P_2018_I8F_MC_GPGB_1
1.58 [0.06, 39.43]
Tirzepatide_low_dosage vs Tirzepatide_high_dosage
Frias_J_P_2018_I8F_MC_GPGB_3
0.32 [0.01, 7.97]
Fr_as_J_P_2021_SURPASS_2_2
0.66 [0.03, 16.35]
Rosenstock_J_2023_SURPASS_6_2
0.39 [0.02, 8.12]
Efpeglenatide_low_dosage vs Efpeglenatide_medium_dosage
Frias_J_P_2022_AMPLITUDE_M_1
0.33 [0.01, 8.28]
Exenatide vs Placebo_or_Control
Gallwitz_B_2011_H8O_SB_GWBN_
0.31 [0.01, 7.73]
Gallwitz_B_2012_EUREXA_
0.99 [0.06, 15.94]
Holman_R_R_2017_EXSCEL_
0.33 [0.01, 8.21]
Albiglutide vs Placebo_or_Control
Hernandez_A_F_2018_Harmony_Outcomes_
0.88 [0.59, 1.32]
Weissman_P_N_2014_HARMONY_4_
0.48 [0.03, 7.66]
Albiglutide vs Liraglutide
Pratley_R_E_2014_HARMONY_7_
3.04 [0.12, 74.78]

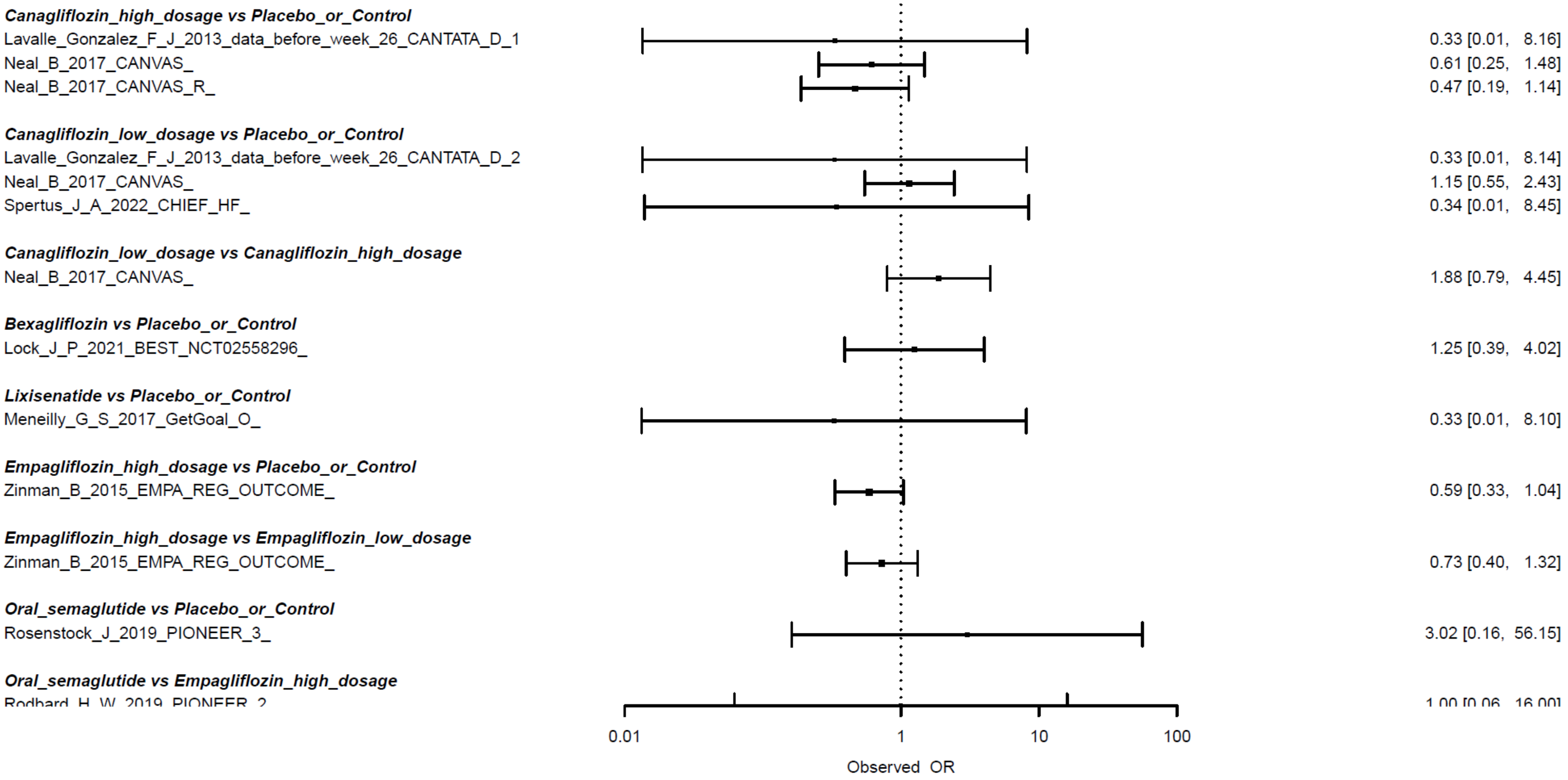
Canagliflozin_high_dosage vs Placebo_or_Control
Lavalle_Gonzalez_F_J_2013_data_before_week_26_CANTATA_D_1
0.33 [0.01, 8.16]
Neal_B_2017_CANVAS_
0.61 [0.25, 1.48]
Neal_B_2017_CANVAS_R_
0.47 [0.19, 1.14]
Canagliflozin_low_dosage vs Placebo_or_Control
Lavalle_Gonzalez_F_J_2013_data_before_week_26_CANTATA_D_2
0.33 [0.01, 8.14]
Neal_B_2017_CANVAS_
1.15 [0.55, 2.43]
Spertus_J_A_2022_CHIEF_HF_
0.34 [0.01, 8.45]
Canagliflozin_low_dosage vs Canagliflozin_high_dosage
Neal_B_2017_CANVAS_
1.88 [0.79, 4.45]
Bexagliflozin vs Placebo_or_Control
Lock_J_P_2021_BEST_NCT02558296_
1.25 [0.39, 4.02]
Lixisenatide vs Placebo_or_Control
Meneilly_G_S_2017_GetGoal_O_
0.33 [0.01, 8.10]
Empagliflozin_high_dosage vs Placebo_or_Control
Zinman_B_2015_EMPA_REG_OUTCOME_
0.59 [0.33, 1.04]
Empagliflozin_high_dosage vs Empagliflozin_low_dosage
Zinman_B_2015_EMPA_REG_OUTCOME_
0.73 [0.40, 1.32]
Oral_semaglutide vs Placebo_or_Control
Rosenstock_J_2019_PIONEER_3_
3.02 [0.16, 56.15]
Oral_semaglutide vs Empagliflozin_high_dosage
Rodbard_H_W_2019_PIONEER_2
1.00 [0.06, 16.00]
0.01
1
10
100
Observed OR

Figure S3C Individual study result of safety profile: drop-out rate

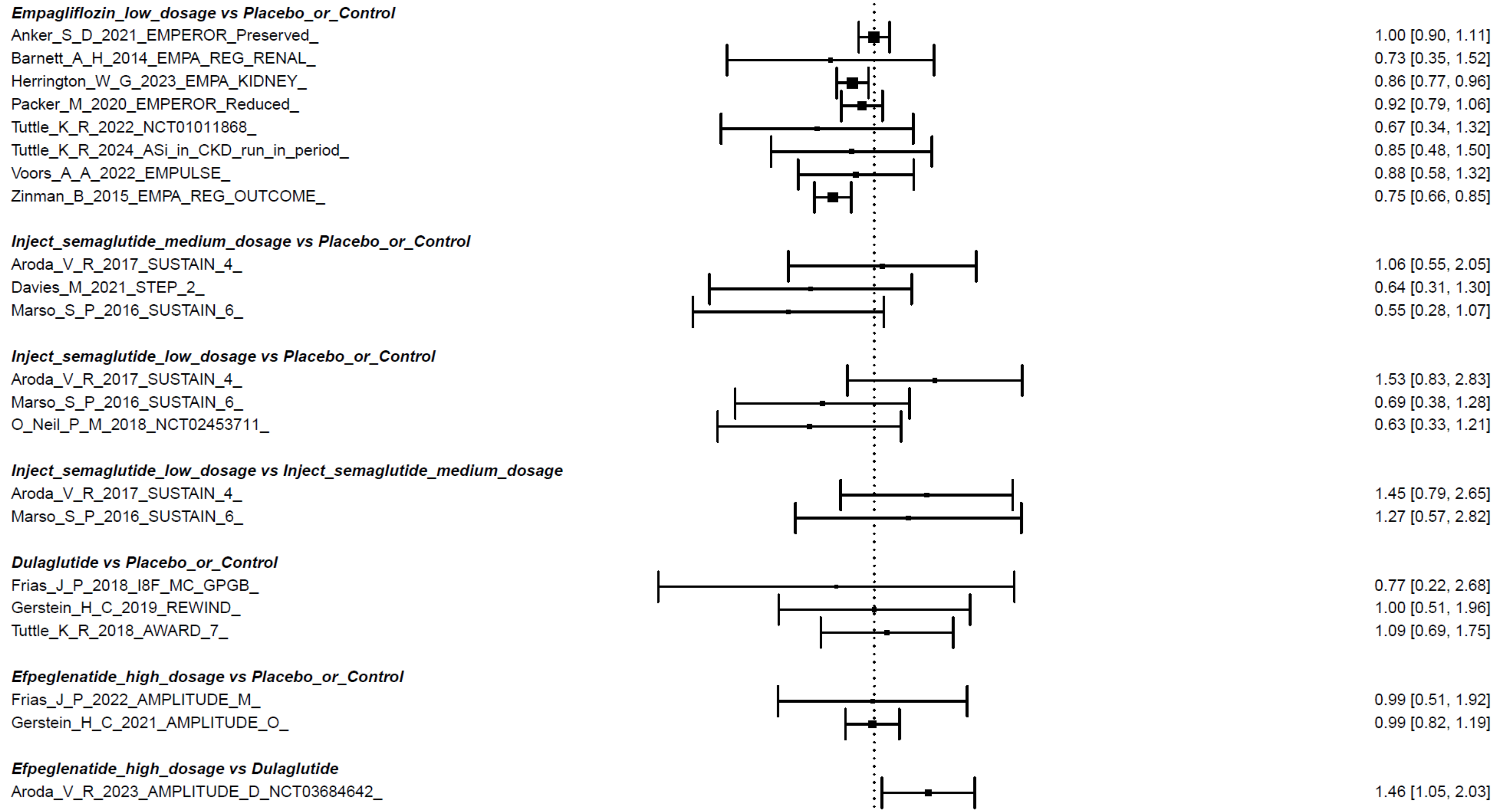

Efpeglenatide_medium_dosage vs Placebo_or_Control

Frias_J_P_2022_AMPLITUDE_M_ 1.13 [0.59, 2.19]
Gerstein_H_C_2021_AMPLITUDE_O_ 0.97 [0.80, 1.17]

Efpeglenatide_medium_dosage vs Dulaglutide

Aroda_V_R_2023_AMPLITUDE_D_NCT03684642_ 0.96 [0.68, 1.34]

Efpeglenatide_medium_dosage vs Efpeglenatide_high_dosage

Aroda_V_R_2023_AMPLITUDE_D_NCT03684642_ 0.65 [0.47, 0.91]
Frias_J_P_2022_AMPLITUDE_M_ 1.15 [0.59, 2.21]
Gerstein_H_C_2021_AMPLITUDE_O_ 0.98 [0.81, 1.19]

Dapagliflozin vs Placebo_or_Control

Bailey_C_J_2010_MB102_014_ 0.70 [0.40, 1.20]
Cefalu_W_T_2015_ 0.99 [0.67, 1.46]
Heerspink_H_J_L_2020_DAPA_CKD_ 2.00 [0.68, 5.87]
Kosiborod_M_N_2021_DARE_19_ 1.61 [0.52, 4.94]
Leiter_L_A_2016_D1690C00019_ 0.72 [0.47, 1.11]
McMurray_J_J_V_2019_DAPA_HF_ 0.83 [0.25, 2.73]
Mellander_A_2016_NCT00528372_ 0.92 [0.47, 1.79]
Solomon_S_D_2022_DELIVER_ 0.91 [0.39, 2.14]
Wiviott_S_D_2019_DECLARE_TIMI_58_ 0.75 [0.58, 0.96]

Empagliflozin_high_dosage vs Placebo_or_Control

Barnett_A_H_2014_EMPA_REG_RENAL_ 0.97 [0.61, 1.53]
Ridderstrale_M_2014_EMPA_REG_H2H_SU_ 0.80 [0.63, 1.02]
Tuttle_K_R_2022_NCT01011868_ 1.29 [0.70, 2.37]
Zinman_B_2015_EMPA_REG_OUTCOME_ 0.73 [0.64, 0.83]

Empagliflozin_high_dosage vs Empagliflozin_low_dosage

Barnett_A_H_2014_EMPA_REG_RENAL_ 1.32 [0.64, 2.74]
Tuttle_K_R_2022_NCT01011868_ 1.93 [0.99, 3.75]
Zinman_B_2015_EMPA_REG_OUTCOME_ 0.97 [0.85, 1.11]

Sotagliflozin vs Placebo_or_Control

Bhatt_D_L_2021_SCORED_ 0.89 [0.79, 1.01]
Bhatt_D_L_2021_SOLOIST_WHF_ 0.90 [0.69, 1.18]
Buse_J_B_2018_inTandem1_ 0.74 [0.50, 1.09]
Cherney_D_Z_I_2023_SOTA_CKD3_ 0.73 [0.46, 1.14]

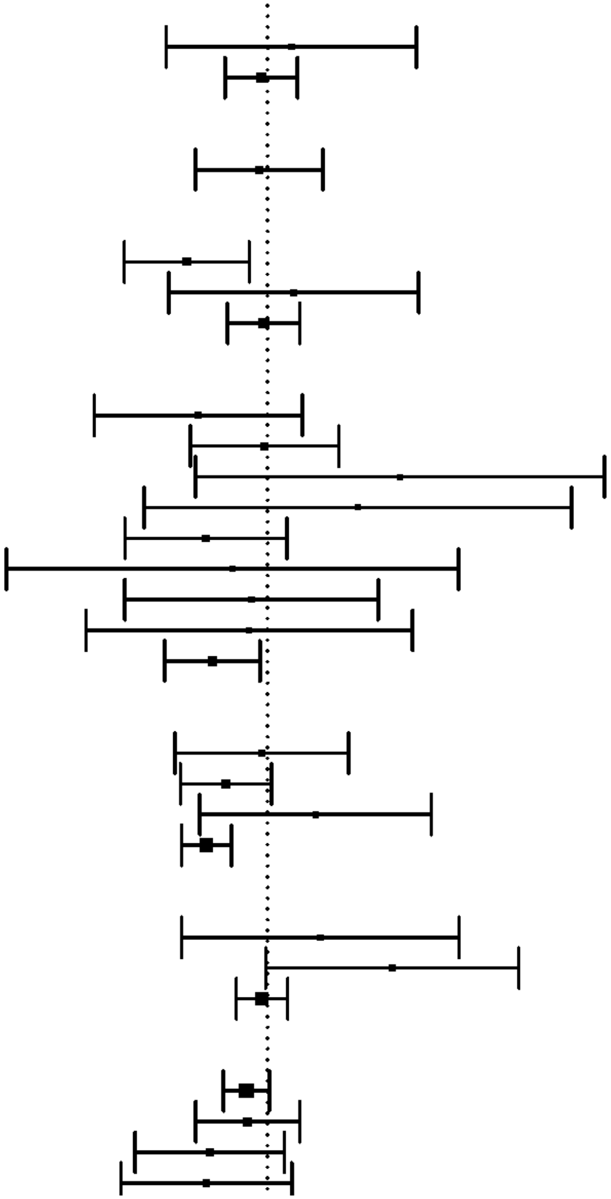

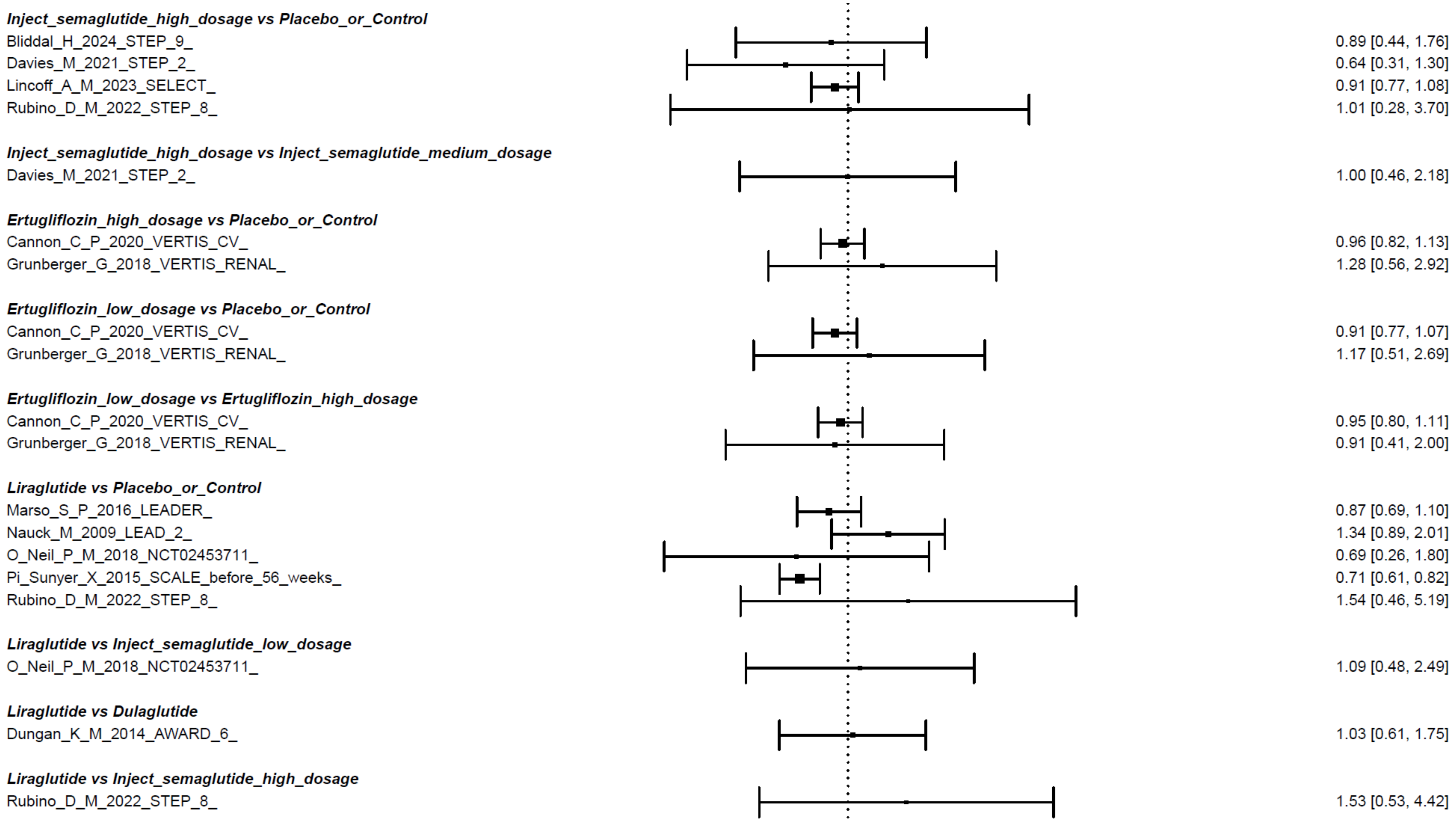

Inject_semaglutide_high_dosage vs Placebo_or_Control
Bliddal_H_2024_STEP_9_ 0.89 [0.44, 1.76]
Davies_M_2021_STEP_2_ 0.64 [0.31, 1.30]
Lincoff_A_M_2023_SELECT_ 0.91 [0.77, 1.08]
Rubino_D_M_2022_STEP_8_ 1.01 [0.28, 3.70]
Inject_semaglutide_high_dosage vs Inject_semaglutide_medium_dosage
Davies_M_2021_STEP_2_ 1.00 [0.46, 2.18]
Ertugliflozin_high_dosage vs Placebo_or_Control
Cannon_C_P_2020_VERTIS_CV_ 0.96 [0.82, 1.13]
Grunberger_G_2018_VERTIS_RENAL_ 1.28 [0.56, 2.92]
Ertugliflozin_low_dosage vs Placebo_or_Control
Cannon_C_P_2020_VERTIS_CV_ 0.91 [0.77, 1.07]
Grunberger_G_2018_VERTIS_RENAL_ 1.17 [0.51, 2.69]
Ertugliflozin_low_dosage vs Ertugliflozin_high_dosage
Cannon_C_P_2020_VERTIS_CV_ 0.95 [0.80, 1.11]
Grunberger_G_2018_VERTIS_RENAL_ 0.91 [0.41, 2.00]
Liraglutide vs Placebo_or_Control
Marso_S_P_2016_LEADER_ 0.87 [0.69, 1.10]
Nauck_M_2009_LEAD_2_ 1.34 [0.89, 2.01]
O_Neil_P_M_2018_NCT02453711_ 0.69 [0.26, 1.80]
Pi_Sunyer_X_2015_SCALE_before_56_weeks_ 0.71 [0.61, 0.82]
Rubino_D_M_2022_STEP_8_ 1.54 [0.46, 5.19]
Liraglutide vs Inject_semaglutide_low_dosage
O_Neil_P_M_2018_NCT02453711_ 1.09 [0.48, 2.49]
Liraglutide vs Dulaglutide
Dungan_K_M_2014_AWARD_6_ 1.03 [0.61, 1.75]
Liraglutide vs Inject_semaglutide_high_dosage
Rubino_D_M_2022_STEP_8_ 1.53 [0.53, 4.42]

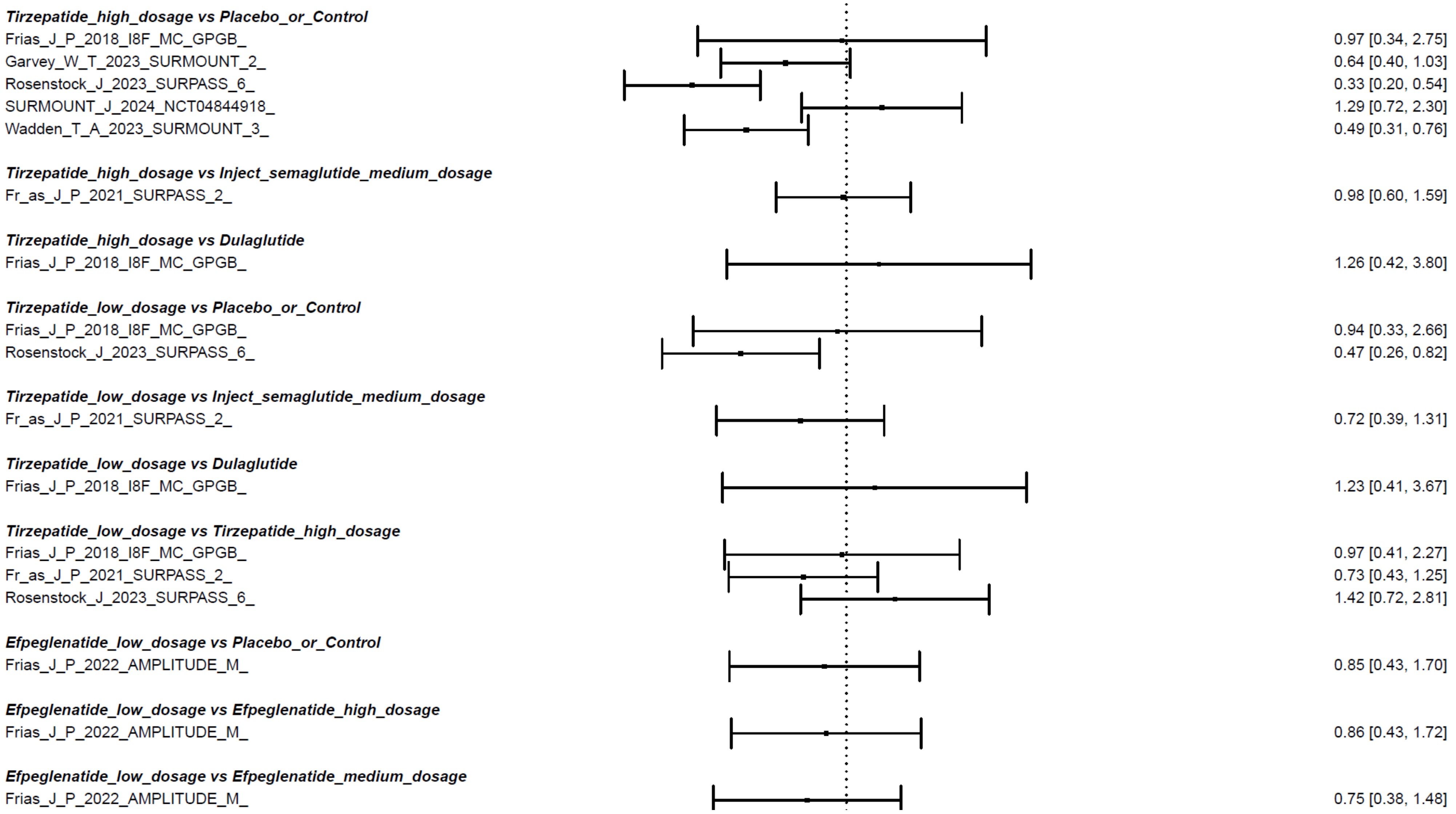
Tirzepatide_high_dosage vs Placebo_or_Control
Frias_J_P_2018_I8F_MC_GPGB_ 0.97 [0.34, 2.75]
Garvey_W_T_2023_SURMOUNT_2_ 0.64 [0.40, 1.03]
Rosenstock_J_2023_SURPASS_6_ 0.33 [0.20, 0.54]
SURMOUNT_J_2024_NCT04844918_ 1.29 [0.72, 2.30]
Wadden_T_A_2023_SURMOUNT_3_ 0.49 [0.31, 0.76]
Tirzepatide_high_dosage vs Inject_semaglutide_medium_dosage
Fr_as_J_P_2021_SURPASS_2_ 0.98 [0.60, 1.59]
Tirzepatide_high_dosage vs Dulaglutide
Frias_J_P_2018_I8F_MC_GPGB_ 1.26 [0.42, 3.80]
Tirzepatide_low_dosage vs Placebo_or_Control
Frias_J_P_2018_I8F_MC_GPGB_ 0.94 [0.33, 2.66]
Rosenstock_J_2023_SURPASS_6_ 0.47 [0.26, 0.82]
Tirzepatide_low_dosage vs Inject_semaglutide_medium_dosage
Fr_as_J_P_2021_SURPASS_2_ 0.72 [0.39, 1.31]
Tirzepatide_low_dosage vs Dulaglutide
Frias_J_P_2018_I8F_MC_GPGB_ 1.23 [0.41, 3.67]
Tirzepatide_low_dosage vs Tirzepatide_high_dosage
Frias_J_P_2018_I8F_MC_GPGB_ 0.97 [0.41, 2.27]
Fr_as_J_P_2021_SURPASS_2_ 0.73 [0.43, 1.25]
Rosenstock_J_2023_SURPASS_6_ 1.42 [0.72, 2.81]
Efpeglenatide_low_dosage vs Placebo_or_Control
Frias_J_P_2022_AMPLITUDE_M_ 0.85 [0.43, 1.70]
Efpeglenatide_low_dosage vs Efpeglenatide_high_dosage
Frias_J_P_2022_AMPLITUDE_M_ 0.86 [0.43, 1.72]
Efpeglenatide_low_dosage vs Efpeglenatide_medium_dosage
Frias_J_P_2022_AMPLITUDE_M_ 0.75 [0.38, 1.48]

Comparison / Study	Estimate [95% CI]
Exenatide vs Placebo_or_Control	
Gallwitz_B_2011_H8O_SB_GWBN_	1.22 [0.83, 1.78]
Gallwitz_B_2012_EUREXA_	1.54 [1.17, 2.02]
Holman_R_R_2017_EXSCEL_	0.86 [0.73, 1.02]
Albiglutide vs Placebo_or_Control	
Hernandez_A_F_2018_Harmony_Outcomes_	0.71 [0.56, 0.91]
Weissman_P_N_2014_HARMONY_4_	1.36 [0.98, 1.88]
Albiglutide vs Liraglutide	
Pratley_R_E_2014_HARMONY_7_	0.84 [0.57, 1.23]
Oral_semaglutide vs Placebo_or_Control	
Husain_M_2019_PIONEER_6_	0.83 [0.25, 2.74]
Mosenzon_O_2019_PIONEER_5_	0.99 [0.28, 3.48]
Rosenstock_J_2019_PIONEER_3_	1.94 [1.13, 3.34]
Oral_semaglutide vs Empagliflozin_high_dosage	
Rodbard_H_W_2019_PIONEER_2_	0.46 [0.22, 0.96]
Canagliflozin_high_dosage vs Placebo_or_Control	
Lavalle_Gonzalez_F_J_2013_data_before_week_26_CANTATA_D_	0.75 [0.45, 1.26]
Neal_B_2017_CANVAS_	0.58 [0.44, 0.77]
Neal_B_2017_CANVAS_R_	0.90 [0.57, 1.42]
Stenl_f_K_2014_CANTATA_M_	0.63 [0.35, 1.13]
Canagliflozin_low_dosage vs Placebo_or_Control	
Lavalle_Gonzalez_F_J_2013_data_before_week_26_CANTATA_D_	0.79 [0.48, 1.31]
Neal_B_2017_CANVAS_	0.67 [0.52, 0.88]
Perkovic_V_2019_CREDENCE_	0.60 [0.31, 1.13]
Spertus_J_A_2022_CHIEF_HF_	0.93 [0.54, 1.59]
Stenl_f_K_2014_CANTATA_M_	0.67 [0.38, 1.19]
Wada_T_2022_TA_7284_14_	1.14 [0.64, 2.02]
Canagliflozin_low_dosage vs Canagliflozin_high_dosage	
Lavalle_Gonzalez_F_J_2013_data_before_week_26_CANTATA_D_	1.05 [0.67, 1.63]
Neal_B_2017_CANVAS_	1.16 [0.86, 1.56]
Stenl_f_K_2014_CANTATA_M_	1.06 [0.57, 1.98]

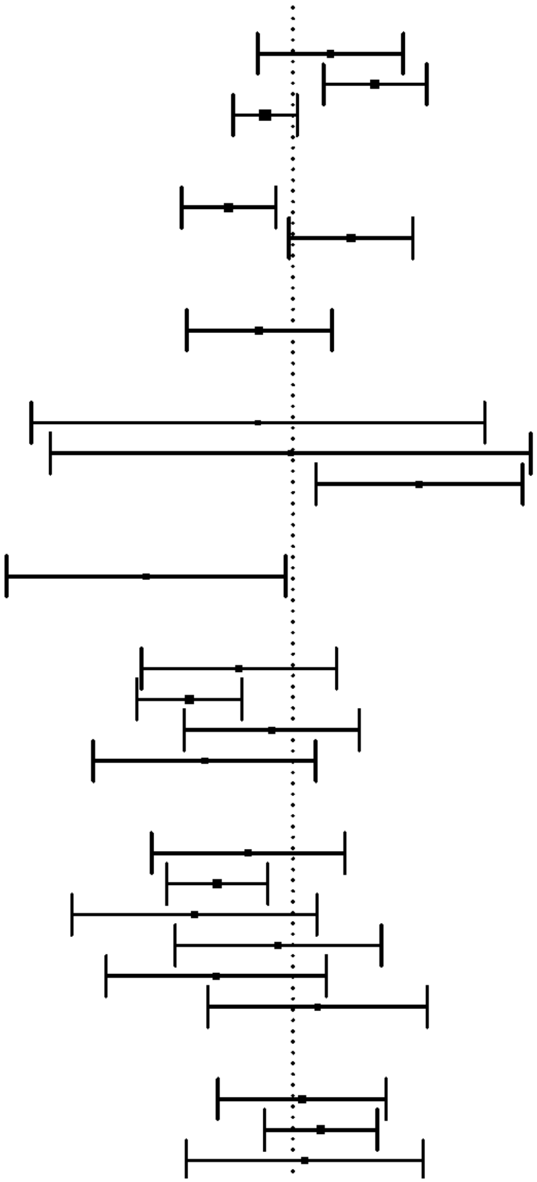

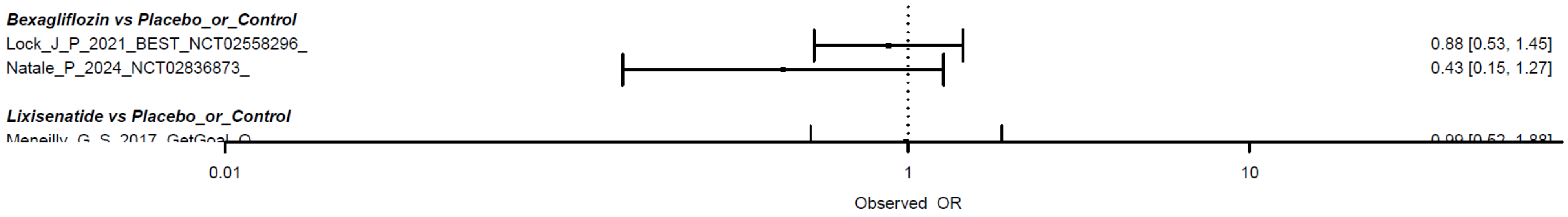
Bexagliflozin vs Placebo_or_Control
Lock_J_P_2021_BEST_NCT02558296_
0.88 [0.53, 1.45]
Natale_P_2024_NCT02836873_
0.43 [0.15, 1.27]
Lixisenatide vs Placebo_or_Control
0.01
1
10
Observed OR

Abbreviation for Figure S3A-3C:

95%CIs: 95% confidence intervals; GLP-1 agonist: glucagon-like peptide-1 agonist; NMA: network meta-analysis; OR: odds ratio; RCT: randomized controlled trial; SGLT2 inhibitor: sodium–glucose cotransporter 2 inhibitor

Figure S4A Bayesian-based Litmus Rank-O-Gram rank plot of primary outcome: overall acute kidney injury/acute renal failure

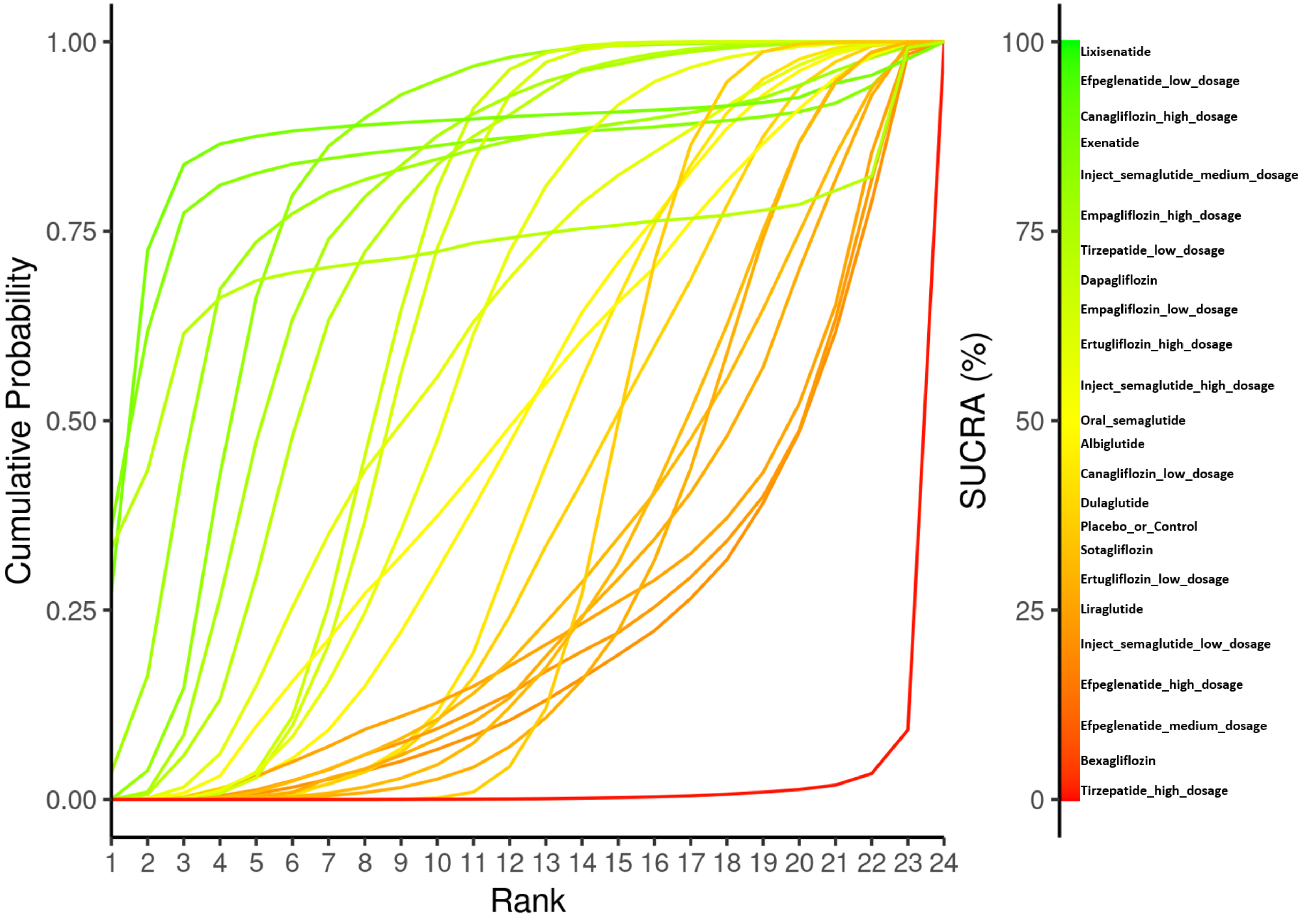

Figure S4B Bayesian-based radial surface under the cumulative ranking of primary outcome: overall acute kidney injury/acute renal failure

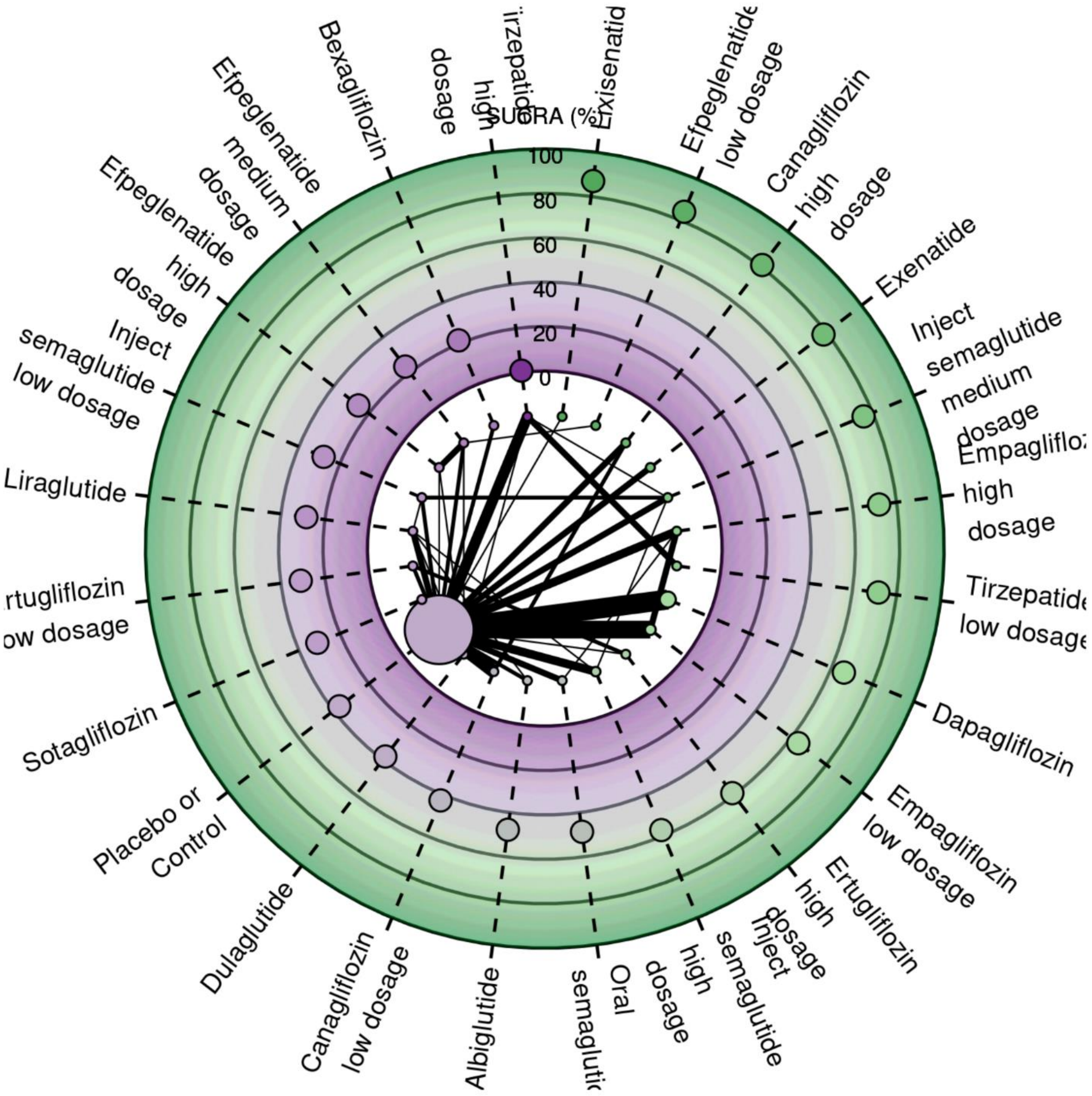

Figure S4C Bayesian-based Litmus Rank-O-Gram rank plot of primary outcome: acute kidney injury/acute renal failure in the subgroup focusing RCTs without definite underlying kidney failure

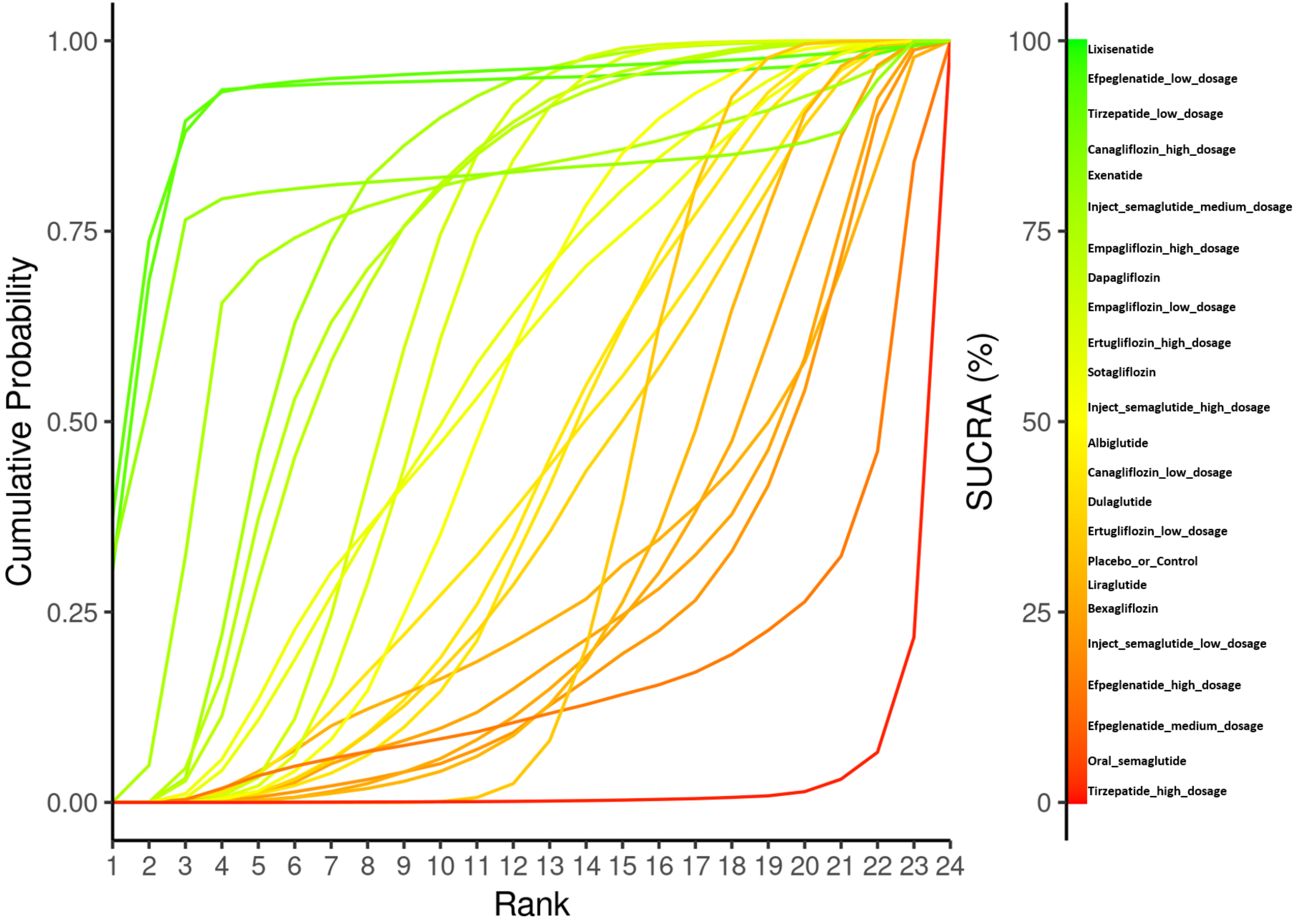

Figure S4D Bayesian-based radial surface under the cumulative ranking of primary outcome: acute kidney injury/acute renal failure in the subgroup focusing RCTs without definite underlying kidney failure

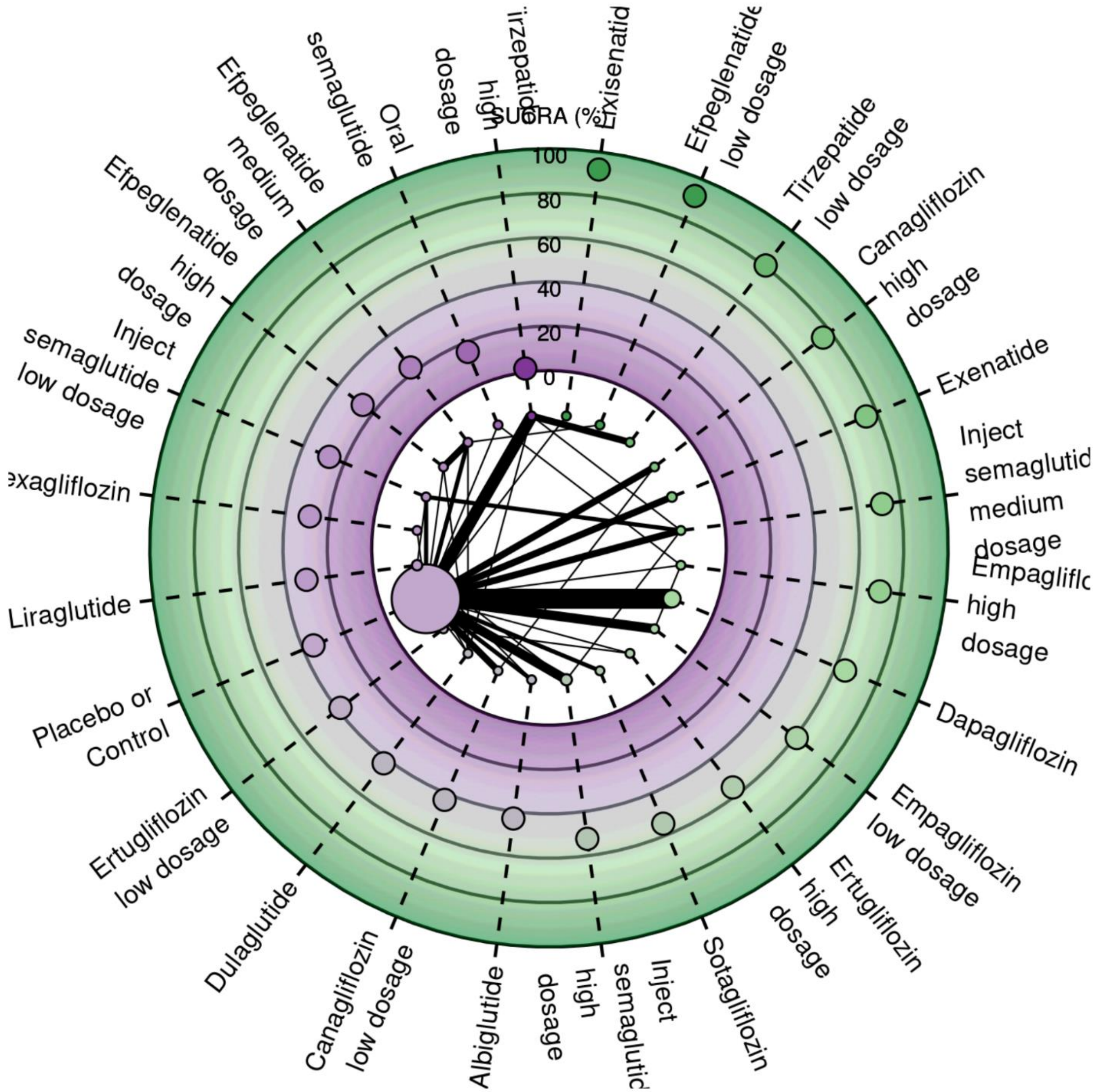

Figure S4E Bayesian-based Litmus Rank-O-Gram rank plot of safety profile: drop-out rate

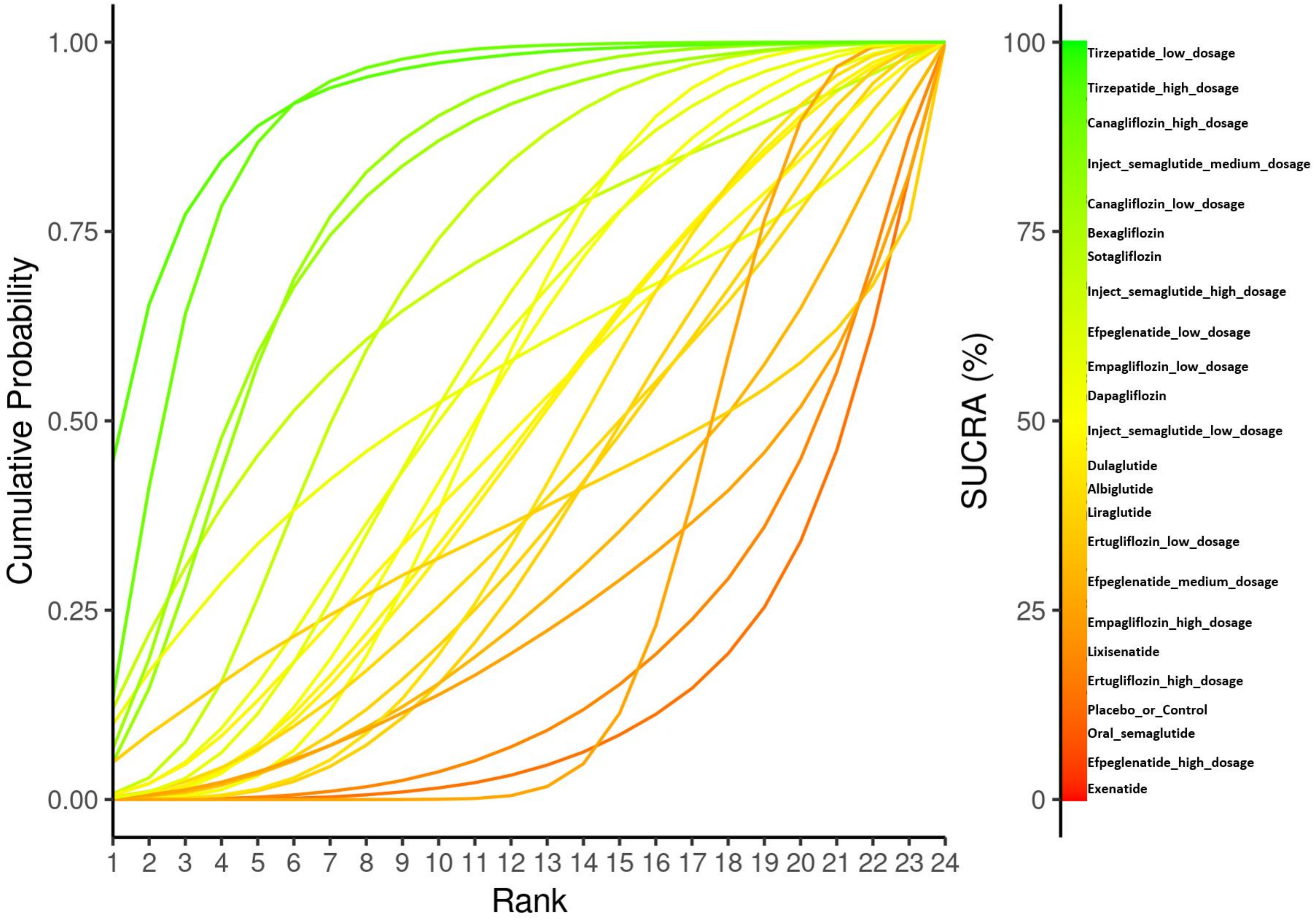

Figure S4F Bayesian-based radial surface under the cumulative ranking of safety profile: drop-out rate

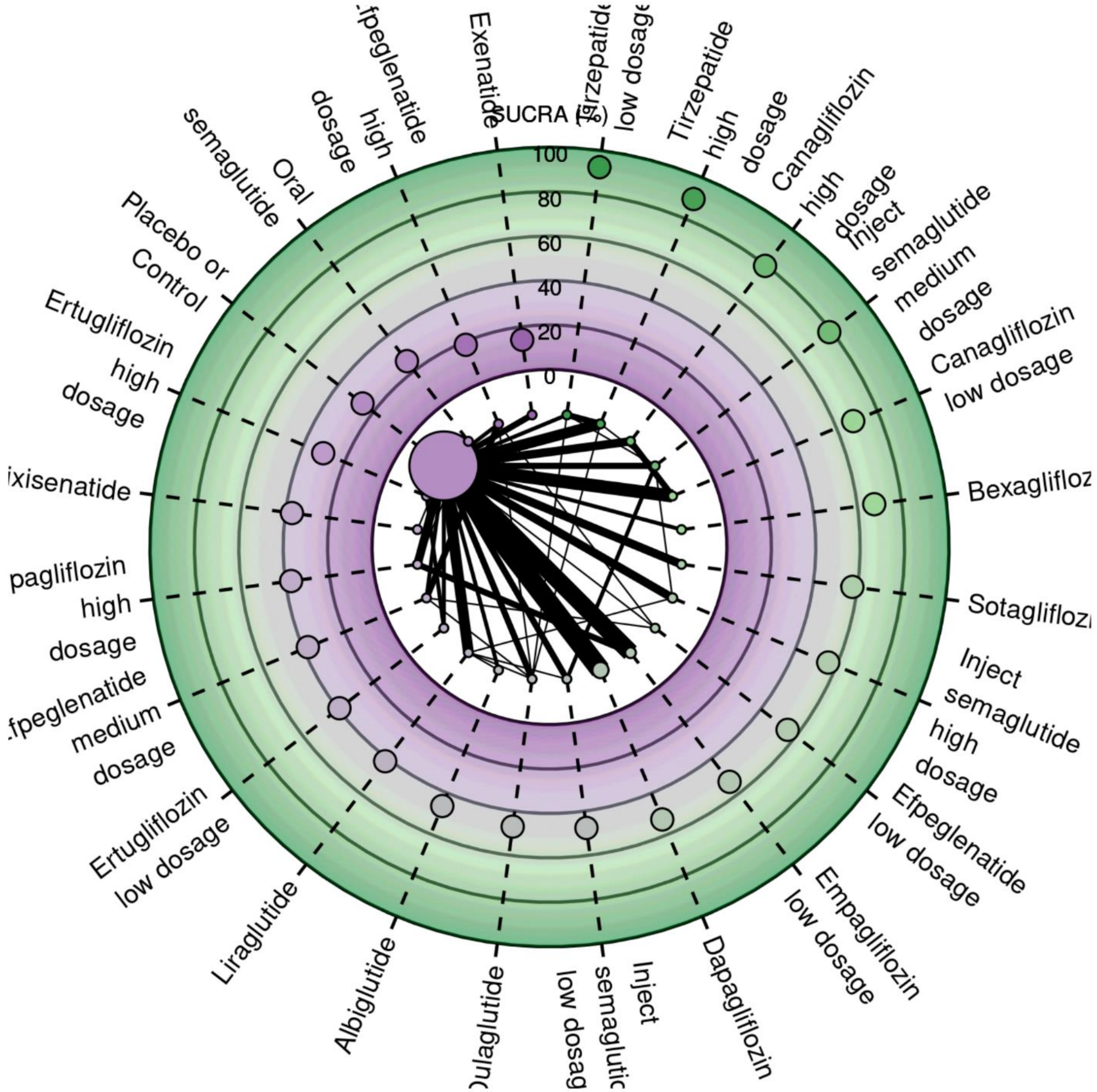

Abbreviation for Figure S4A-4F:

95%CIs: 95% confidence intervals; GLP-1 agonist: glucagon-like peptide-1 agonist; NMA: network meta-analysis; OR: odds ratio; RCT: randomized controlled trial; SGLT2 inhibitor: sodium–glucose cotransporter 2 inhibitor

Figure S5A Bayesian-based residual deviance NMA/UME model of primary outcome: overall acute kidney injury/acute renal failure

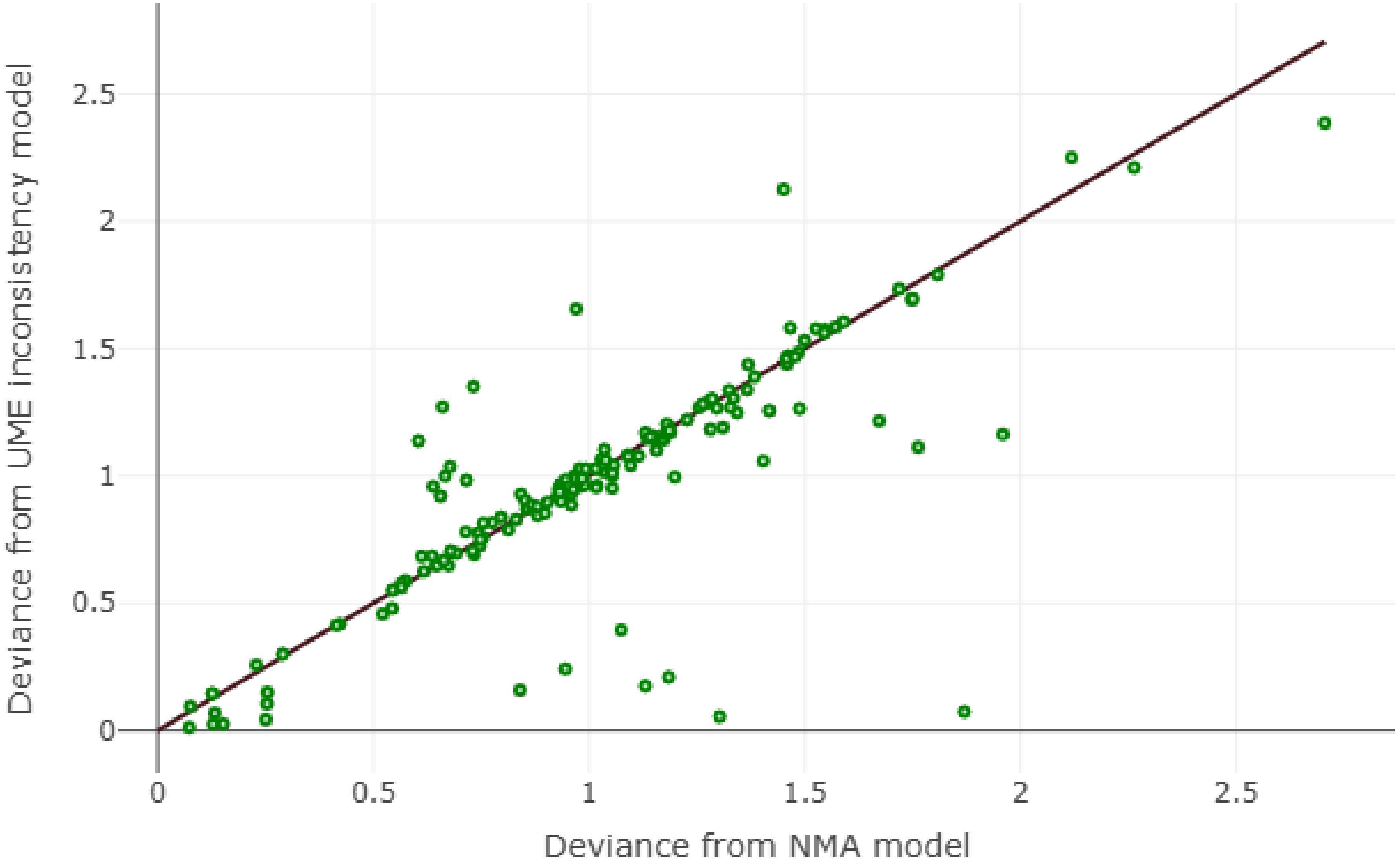

Figure S5B Bayesian-based per-arm residual deviance of primary outcome: overall acute kidney injury/acute renal failure

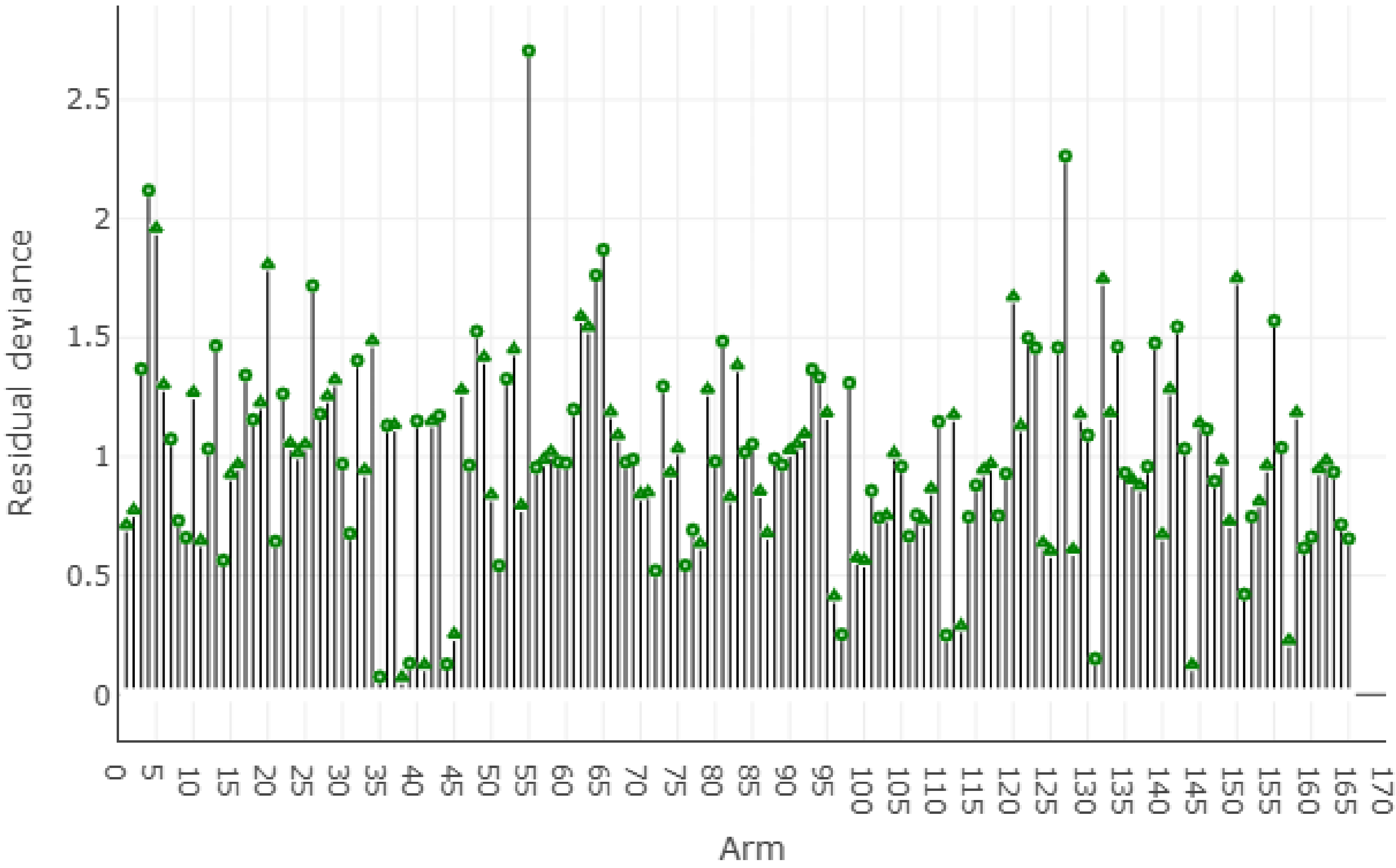

Figure S5C Bayesian-based leverage plot of primary outcome: overall acute kidney injury/acute renal failure

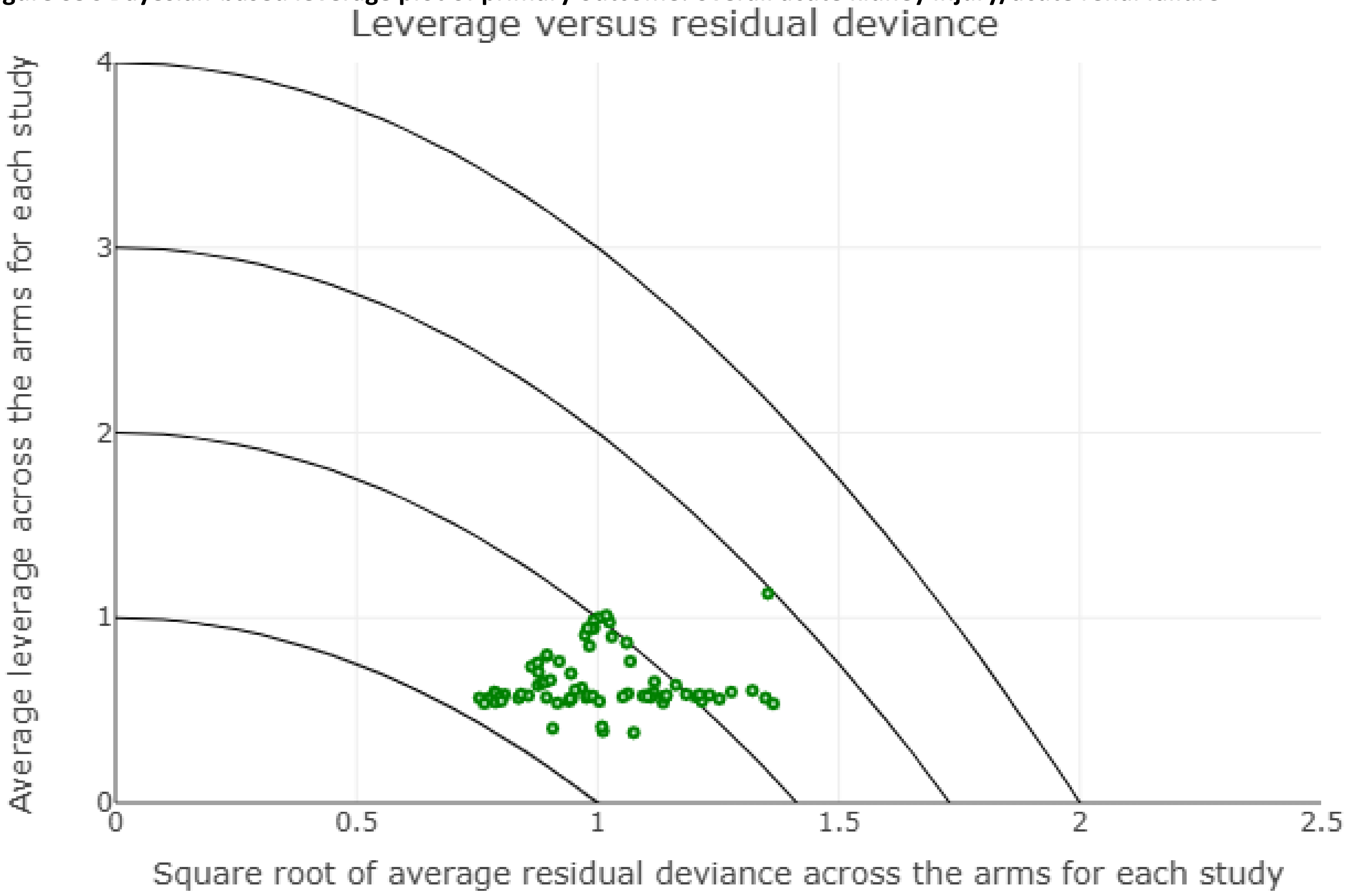

Figure S5D Bayesian-based residual deviance NMA/UME model of primary outcome: acute kidney injury/acute renal failure in the subgroup focusing RCTs without definite underlying kidney failure

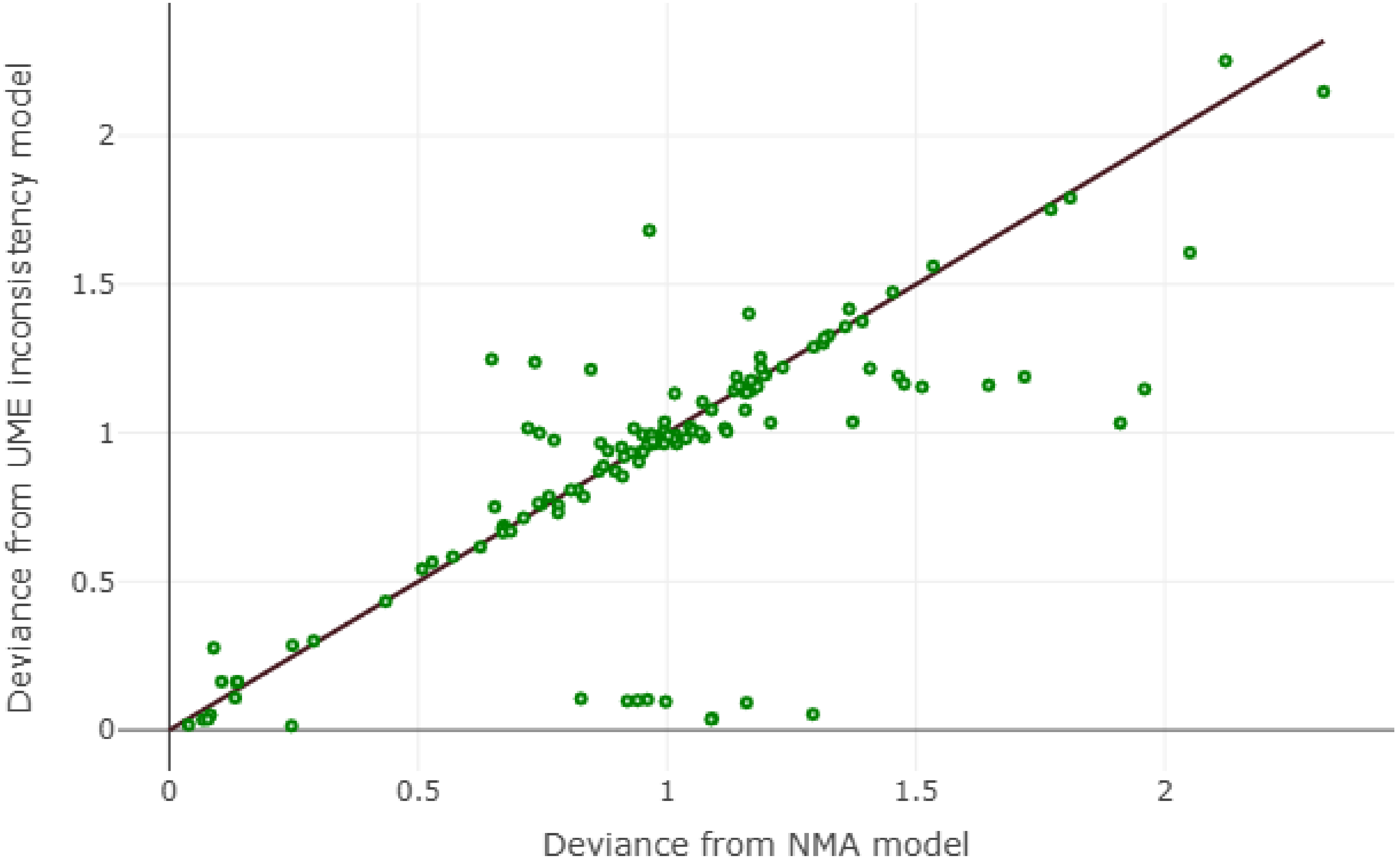

Figure S5E Bayesian-based per-arm residual deviance of primary outcome: acute kidney injury/acute renal failure in the subgroup focusing RCTs without definite underlying kidney failure

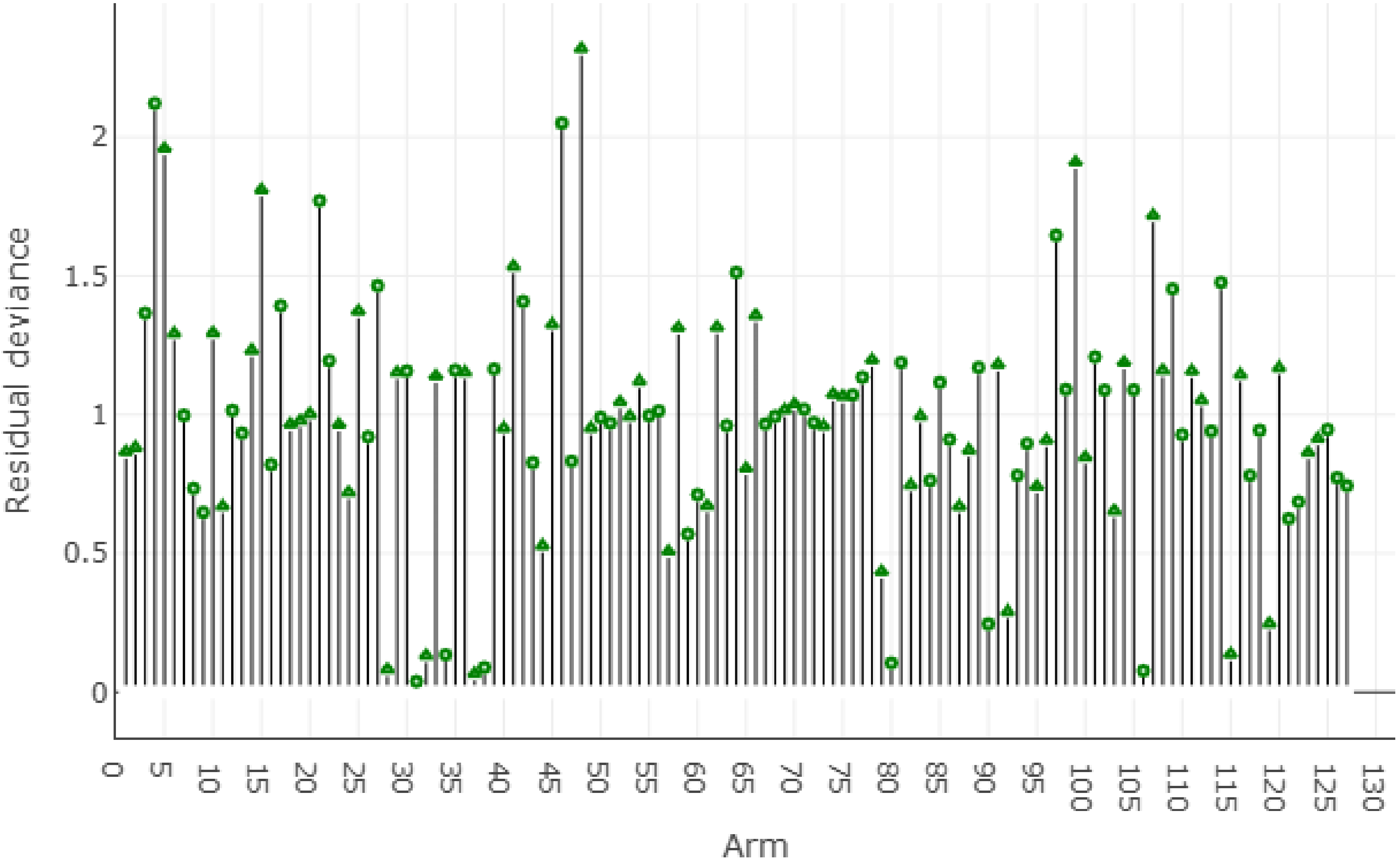

Figure S5F Bayesian-based leverage plot of primary outcome: acute kidney injury/acute renal failure in the subgroup focusing RCTs without definite underlying kidney failure

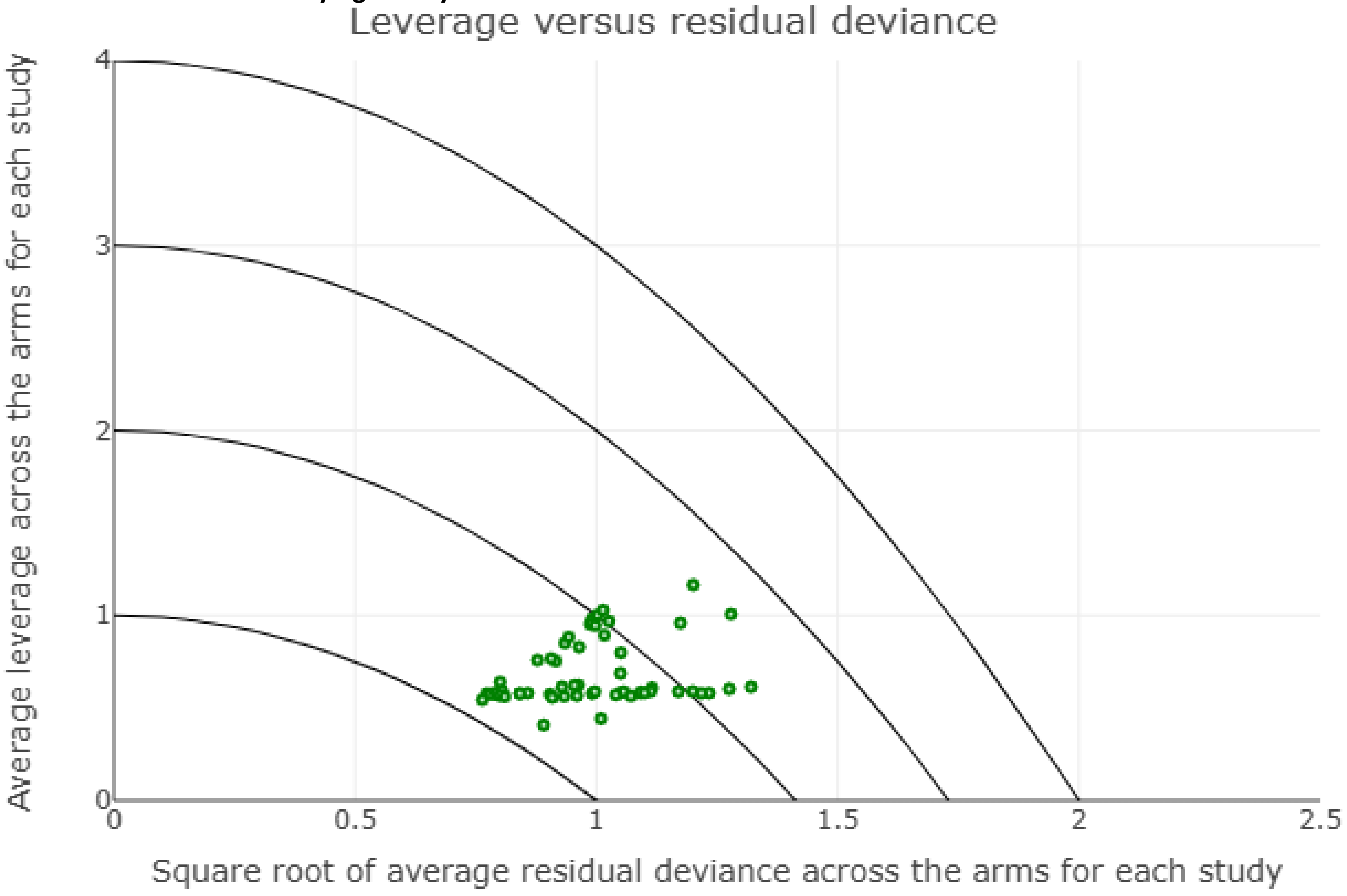

Figure S5G Bayesian-based residual deviance NMA/UME model of safety profile: drop-out rate

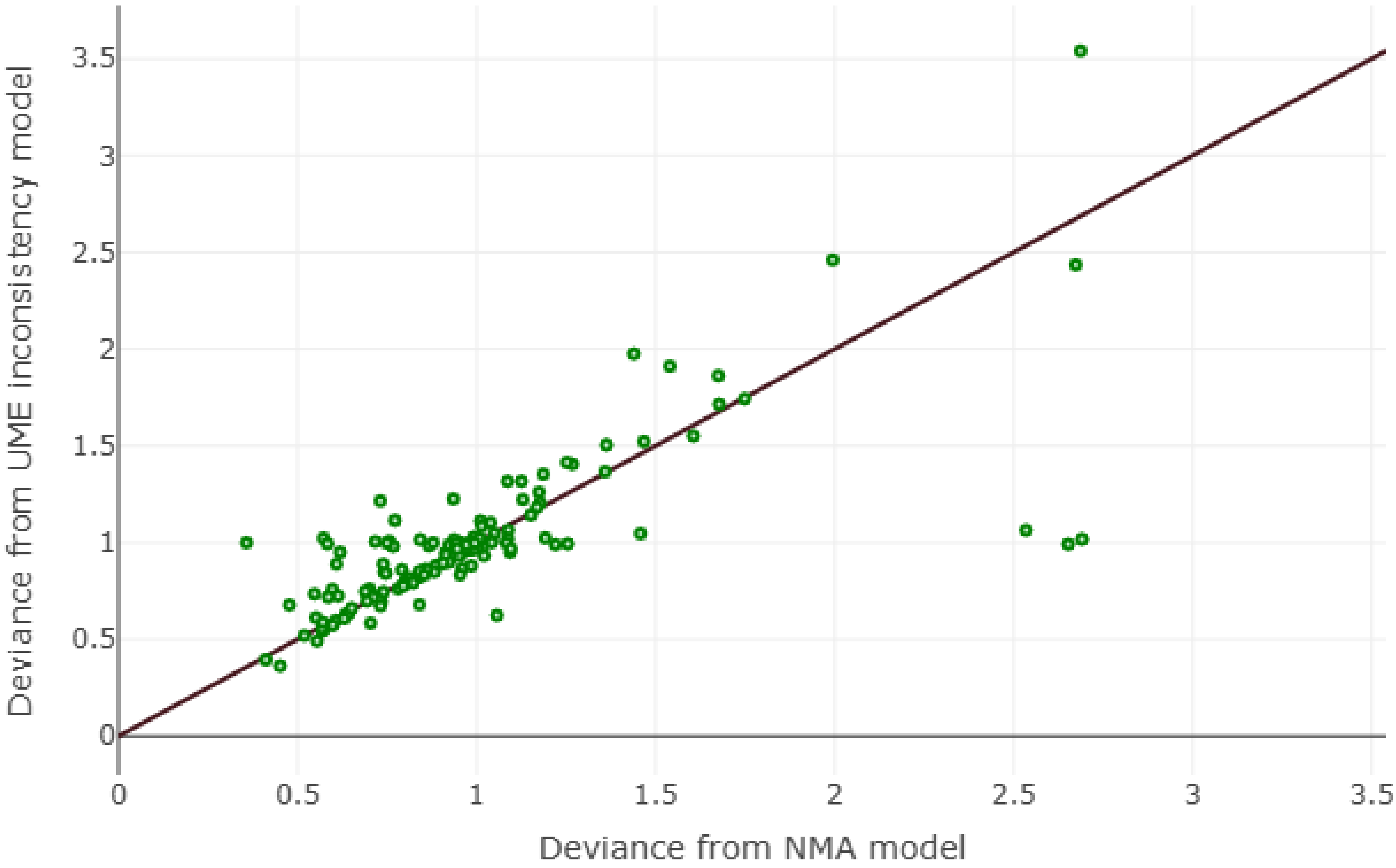

Figure S5H Bayesian-based per-arm residual deviance of safety profile: drop-out rate

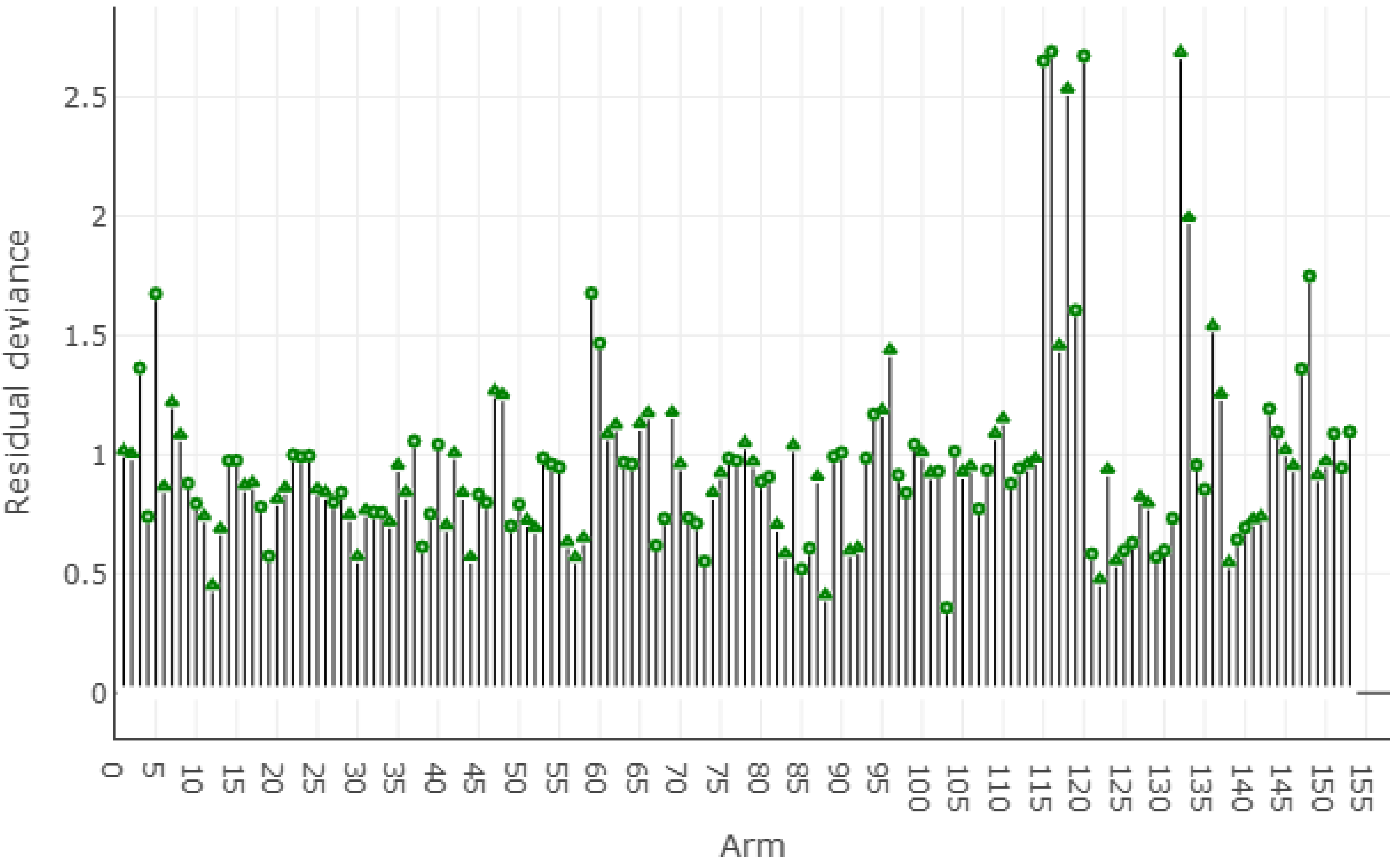

Figure S5I Bayesian-based leverage plot of safety profile: drop-out rate

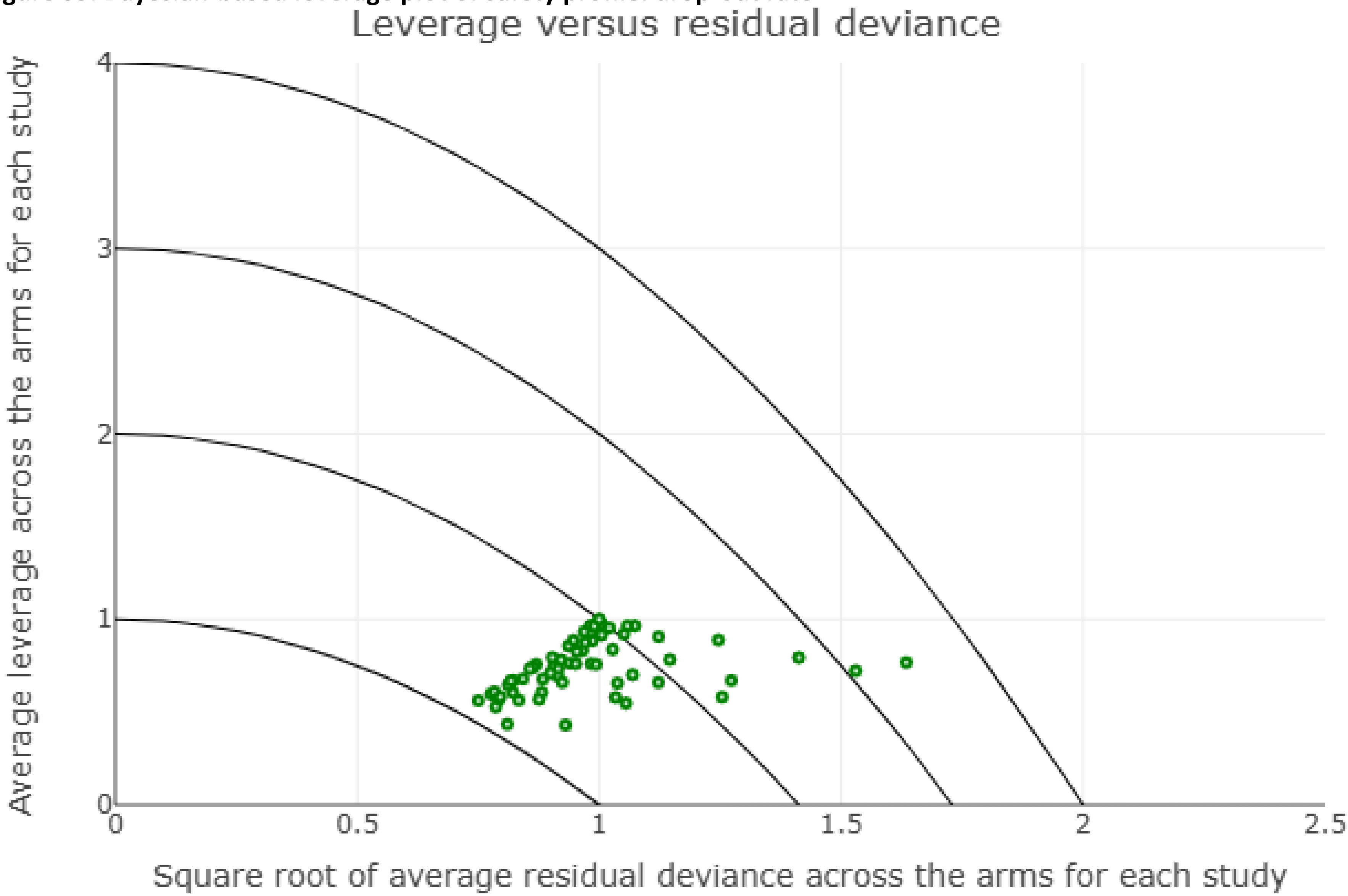

Abbreviation for Figure S5A-5I:

95%CIs: 95% confidence intervals; GLP-1 agonist: glucagon-like peptide-1 agonist; NMA: network meta-analysis; OR: odds ratio; RCT: randomized controlled trial; SGLT2 inhibitor: sodium–glucose cotransporter 2 inhibitor

Figure S6A overview of risk of bias

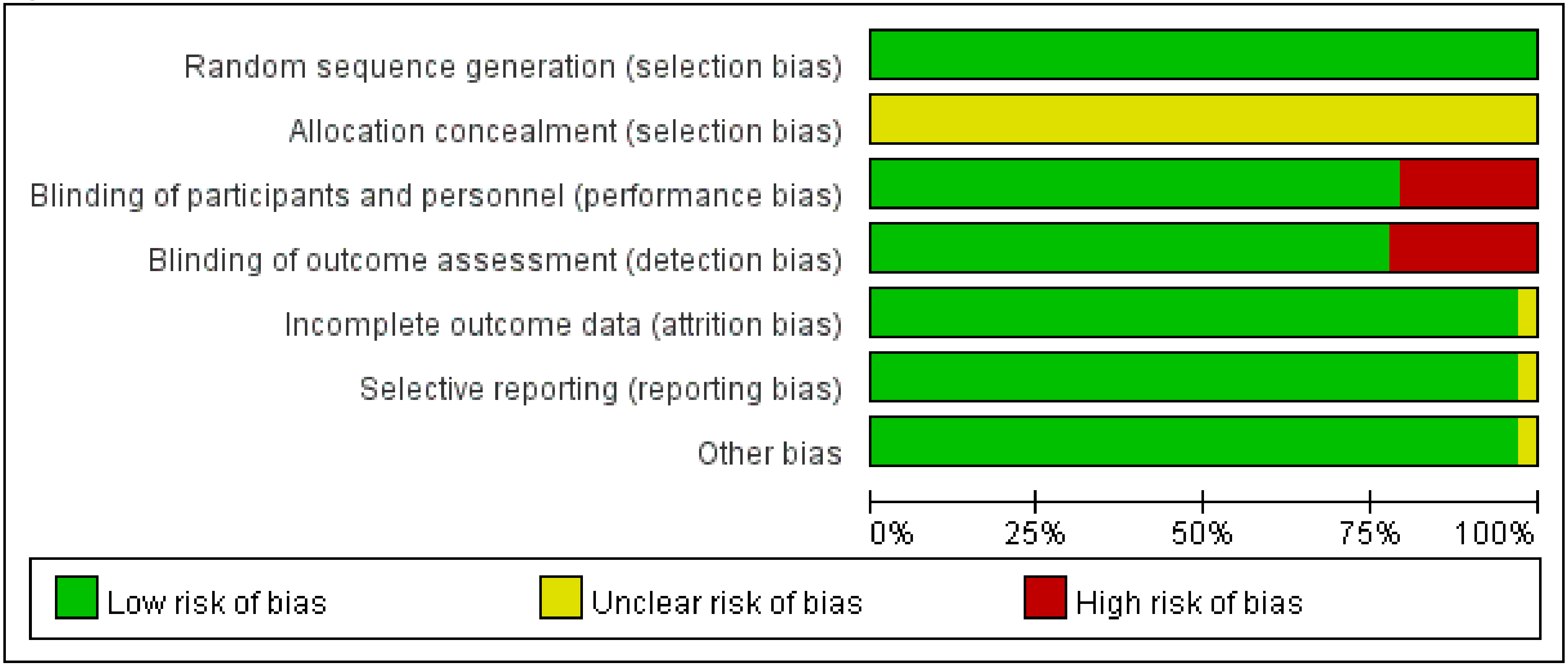

Figure S6B detailed risk of bias in each study

	Random sequence generation (selection bias)	Allocation concealment (selection bias)	Blinding of participants and personnel (performance bias)	Blinding of outcome assessment (detection bias)	Incomplete outcome data (attrition bias)	Selective reporting (reporting bias)	Other bias
Anker, S.D. (2021) (EMPEROR-Preserved)	+	?	+	+	+	+	+
Aroda, V.R. (2017) (SUSTAIN 4)	+	?	−	−	+	+	+
Aroda, V.R. (2023) (AMPLITUDE-D, NCT03684642)	+	?	−	−	+	+	+
Bailey, C.J. (2010) (MB102-014)	+	?	+	+	+	+	+
Barnett, A.H. (2014) (EMPA-REG RENAL)	+	?	+	+	+	+	+
Bhatt, D.L. (2021) (SCORED)	+	?	+	+	+	+	+
Bhatt, D.L. (2021) (SOLOIST-WHF)	+	?	+	+	+	+	+
Bliddal, H. (2024) (STEP 9)	+	?	+	+	+	+	+
Buse, J.B. (2018) (inTandem1)	+	?	+	+	+	+	+
Buse, J.B. (2023) (SEPRA)	+	?	−	−	+	+	+
Cannon, C.P. (2020) (VERTIS CV)	+	?	+	+	+	+	+
Cefalu, W.T. (2015)	+	?	+	+	+	+	+
Cherney, D.Z.I.(2023) (SOTA-CKD3)	+	?	+	+	+	+	+
Davies, M. (2021) (STEP 2)	+	?	+	+	+	+	+
Dungan, K.M. (2014) (AWARD-6)	+	?	−	−	+	+	+
Frias, J.P. (2018) (I8F-MC-GPGB)	+	?	+	+	+	+	+
Frías, J.P. (2021) (SURPASS-2)	+	?	−	−	+	+	+
Frias, J.P. (2022) (AMPLITUDE-M)	+	?	+	+	+	+	+
Gallwitz, B. (2011) (H8O-SB-GWBN)	+	?	−	−	+	+	+
Gallwitz, B. (2012) (EUREXA)	+	?	−	−	+	+	+

Garvey, W.T. (2023) (SURMOUNT-2)	+	?	+	+	+	+	+
Gerstein, H.C. (2019) (REWIND)	+	?	+	+	+	+	+
Gerstein, H.C. (2021) (AMPLITUDE-O)	+	?	+	+	+	+	+
Grunberger, G. (2018) (VERTIS RENAL)	+	?	+	+	+	+	+
Heerspink, H.J.L. (2020) (DAPA-CKD)	+	?	+	+	+	+	+
Hernandez, A.F. (2018) (Harmony Outcomes)	+	?	+	+	+	+	+
Herrington, W.G. (2023) (EMPA-KIDNEY)	+	?	+	+	+	+	+
Holman, R.R. (2017) (EXSCEL)	+	?	+	+	+	+	+
Husain, M. (2019) (PIONEER 6)	+	?	+	+	+	+	+
Kosiborod, M.N. (2021) (DARE-19)	+	?	+	+	+	+	+
Lavalle-Gonzalez, F.J. (2013) (CANTATA-D)	+	?	+	+	+	+	+
Leiter, L.A. (2016) (D1690C00019)	+	?	+	+	+	+	+
Lincoff, A.M. (2023) (SELECT)	+	?	+	+	+	+	+
Lock, J.P. (2021) (BEST) (NCT02558296)	+	?	+	+	?	?	?
Marso, S.P. (2016) (LEADER)	+	?	+	+	+	+	+
Marso, S.P. (2016) (SUSTAIN-6)	+	?	+	+	+	+	+
McMurray, J.J.V. (2019) (DAPA-HF)	+	?	+	+	+	+	+
Mellander, A. (2016) (NCT00528372)	+	?	+	+	+	+	+
Meneilly, G.S. (2017) (GetGoal-O)	+	?	-	-	+	+	+
Mosenzon, O. (2019) (PIONEER 5)	+	?	+	+	+	+	+
Natale, P. (2024) (NCT02836873)	+	?	+	+	+	+	+
Nauck, M. (2009) (LEAD-2)	+	?	+	+	+	+	+
Neal, B. (2017) (CANVAS)	+	?	+	+	+	+	+
Neal, B. (2017) (CANVAS-R)	+	?	+	+	+	+	+
O'Neil, P.M. (2018) (NCT02453711)	+	?	+	+	+	+	+
Packer, M. (2020) (EMPEROR-Reduced)	+	?	+	+	+	+	+

Perkovic, V. (2019) (CREDENCE)	+	?	+	-	+	+	+
Pi-Sunyer, X. (2015) (SCALE) (before 56 weeks)	+	?	+	+	+	+	+
Pratley, R.E. (2014) (HARMONY 7)	+	?	-	-	+	+	+
Ridderstrale, M. (2014) (EMPA-REG H2H-SU)	+	?	+	+	+	+	+
Rodbard, H.W. (2019) (PIONEER 2)	+	?	-	-	+	+	+
Rosenstock, J. (2019) (PIONEER 3)	+	?	+	+	+	+	+
Rosenstock, J. (2023) (SURPASS-6)	+	?	-	-	+	+	+
Rubino, D.M. (2022) (STEP 8)	+	?	-	-	+	+	+
Solomon, S.D. (2022) (DELIVER)	+	?	+	+	+	+	+
Spertus, J.A. (2022) (CHIEF-HF)	+	?	+	+	+	+	+
Stenlöf, K. (2014) (CANTATA-M)	+	?	+	+	+	+	+
SURMOUNT-J (2024) (NCT04844918)	+	?	+	+	?	?	?
Tuttle, K. R. (2018) (AWARD-7)	+	?	-	-	+	+	+
Tuttle, K.R. (2022) (NCT01011868)	+	?	+	+	+	+	+
Tuttle, K.R. (2024) (ASi in CKD) (run-in period)	+	?	+	+	+	+	+
Voors, A.A. (2022) (EMPULSE)	+	?	+	+	+	+	+
Wada, T. (2022) (TA-7284-14)	+	?	+	+	+	+	+
Wadden, T.A. (2023) (SURMOUNT-3)	+	?	+	+	+	+	+
Weissman, P.N. (2014) (HARMONY 4)	+	?	-	-	+	+	+
Wiviott, S.D. (2019) (DECLARE-TIMI 58)	+	?	+	+	+	+	+
Zinman, B. (2015) (EMPA-REG OUTCOME)	+	?	+	+	+	+	+

Table S1: PRISMA 2020 checklist of the current network meta-analysis

Section and Topic	Item #	Checklist item	Page where item is reported
**TITLE**			
Title	1	Identify the report as a systematic review.	1
**ABSTRACT**			
Abstract	2	See the PRISMA 2020 for Abstracts checklist.	7-8
**INTRODUCTION**			
Rationale	3	Describe the rationale for the review in the context of existing knowledge.	9-10
Objectives	4	Provide an explicit statement of the objective(s) or question(s) the review addresses.	9-10
**METHODS**			
Eligibility criteria	5	Specify the inclusion and exclusion criteria for the review and how studies were grouped for the syntheses.	11-12
Information sources	6	Specify all databases, registers, websites, organisations, reference lists and other sources searched or consulted to identify studies. Specify the date when each source was last searched or consulted.	11-12
Search strategy	7	Present the full search strategies for all databases, registers and websites, including any filters and limits used.	11-12
Selection process	8	Specify the methods used to decide whether a study met the inclusion criteria of the review, including how many reviewers screened each record and each report retrieved, whether they worked independently, and if applicable, details of automation tools used in the process.	11-12
Data collection process	9	Specify the methods used to collect data from reports, including how many reviewers collected data from each report, whether they worked independently, any processes for obtaining or confirming data from study investigators, and if applicable, details of automation tools used in the process.	11-12
Data items	10a	List and define all outcomes for which data were sought. Specify whether all results that were compatible with each outcome domain in each study were sought (e.g. for all measures, time points, analyses), and if not, the methods used to decide which results to collect.	12-13
	10b	List and define all other variables for which data were sought (e.g. participant and intervention characteristics, funding sources). Describe any assumptions made about any missing or unclear information.	12-13
Study risk of bias assessment	11	Specify the methods used to assess risk of bias in the included studies, including details of the tool(s) used, how many reviewers assessed each study and whether they worked independently, and if applicable, details of automation tools used in the process.	12-13
Effect measures	12	Specify for each outcome the effect measure(s) (e.g. risk ratio, mean difference) used in the synthesis or presentation of results.	12-13
Synthesis methods	13a	Describe the processes used to decide which studies were eligible for each synthesis (e.g. tabulating the study intervention characteristics and comparing against the planned groups for each synthesis (item #5)).	12-13
	13b	Describe any methods required to prepare the data for presentation or synthesis, such as handling of missing summary statistics, or data conversions.	13-15
	13c	Describe any methods used to tabulate or visually display results of individual studies and syntheses.	13-15
	13d	Describe any methods used to synthesize results and provide a rationale for the choice(s). If meta-analysis was performed, describe the model(s), method(s) to identify the presence and extent of statistical heterogeneity, and software package(s) used.	13-15
	13e	Describe any methods used to explore possible causes of heterogeneity among study results (e.g. subgroup analysis, meta-regression).	13-15
	13f	Describe any sensitivity analyses conducted to assess robustness of the synthesized results.	13-15

Section and Topic	Item #	Checklist item	Page where item is reported
Reporting bias assessment	14	Describe any methods used to assess risk of bias due to missing results in a synthesis (arising from reporting biases).	13-15
Certainty assessment	15	Describe any methods used to assess certainty (or confidence) in the body of evidence for an outcome.	13-15
**RESULTS**			
Study selection	16a	Describe the results of the search and selection process, from the number of records identified in the search to the number of studies included in the review, ideally using a flow diagram.	16-17, Fig 1, eTab 2
	16b	Cite studies that might appear to meet the inclusion criteria, but which were excluded, and explain why they were excluded.	16-17, eTab 3
Study characteristics	17	Cite each included study and present its characteristics.	16-17, eTab 4
Risk of bias in studies	18	Present assessments of risk of bias for each included study.	16-17, eFig 6
Results of individual studies	19	For all outcomes, present, for each study: (a) summary statistics for each group (where appropriate) and (b) an effect estimate and its precision (e.g. confidence/credible interval), ideally using structured tables or plots.	16-17, eFig 3
Results of syntheses	20a	For each synthesis, briefly summarise the characteristics and risk of bias among contributing studies.	17-18, Fig 2
	20b	Present results of all statistical syntheses conducted. If meta-analysis was done, present for each the summary estimate and its precision (e.g. confidence/credible interval) and measures of statistical heterogeneity. If comparing groups, describe the direction of the effect.	17-18, Fig 3
	20c	Present results of all investigations of possible causes of heterogeneity among study results.	17-18, eTab 7
	20d	Present results of all sensitivity analyses conducted to assess the robustness of the synthesized results.	17-18
Reporting biases	21	Present assessments of risk of bias due to missing results (arising from reporting biases) for each synthesis assessed.	17-18, eFig 6
Certainty of evidence	22	Present assessments of certainty (or confidence) in the body of evidence for each outcome assessed.	17-18
**DISCUSSION**			
Discussion	23a	Provide a general interpretation of the results in the context of other evidence.	19-21
	23b	Discuss any limitations of the evidence included in the review.	21-22
	23c	Discuss any limitations of the review processes used.	21-22
	23d	Discuss implications of the results for practice, policy, and future research.	23
**OTHER INFORMATION**			
Registration and protocol	24a	Provide registration information for the review, including register name and registration number, or state that the review was not registered.	8
	24b	Indicate where the review protocol can be accessed, or state that a protocol was not prepared.	8
	24c	Describe and explain any amendments to information provided at registration or in the protocol.	8
Support	25	Describe sources of financial or non-financial support for the review, and the role of the funders or sponsors in the review.	24
Competing interests	26	Declare any competing interests of review authors.	24
Availability of data,	27	Report which of the following are publicly available and where they can be found: template data collection forms; data extracted from included	24

Section and Topic	Item #	Checklist item	Page where item is reported
code and other materials		studies; data used for all analyses; analytic code; any other materials used in the review.	

The current checklist followed the latest PRISMA 2020 guideline [1].

Table S2: Keyword used in each database and search results

Database	Keyword	Filter	Date	Result
PubMed	(acute kidney injury OR acute renal failure) AND (glucagon-like peptide-1 receptor agonist OR Sodium Glucose Cotransporter 2 Inhibitor OR lixisenatide OR orforglipron OR exenatide OR semaglutide OR liraglutide OR albiglutide OR dulaglutide OR tirzepatide OR bexagliflozin OR canagliflozin OR dapagliflozin OR empagliflozin OR ertugliflozin OR ipragliflozin OR luseogliflozin OR remogliflozin OR sergliflozin OR sotagliflozin OR tofogliflozin OR henagliflozin OR janagliflozin OR mizagliflozin OR velagliflozin OR enavogliflozin OR licogliflozin OR rongliflozin) AND (random OR randomized OR randomised)	NA	2025/02/05	175
ClinicalKey	(acute kidney injury OR acute renal failure) AND (glucagon-like peptide-1 receptor agonist OR Sodium Glucose Cotransporter 2 Inhibitor OR lixisenatide OR orforglipron OR exenatide OR semaglutide OR liraglutide OR albiglutide OR dulaglutide OR tirzepatide OR bexagliflozin OR canagliflozin OR dapagliflozin OR empagliflozin OR ertugliflozin OR ipragliflozin OR luseogliflozin OR remogliflozin OR sergliflozin OR sotagliflozin OR tofogliflozin OR henagliflozin OR janagliflozin OR mizagliflozin OR velagliflozin OR enavogliflozin OR licogliflozin OR rongliflozin) AND (random OR randomized OR randomised)	NA	2025/02/05	921
Cochrane CENTRAL	(acute kidney injury OR acute renal failure) AND (glucagon-like peptide-1 receptor agonist OR Sodium Glucose Cotransporter 2 Inhibitor OR lixisenatide OR orforglipron OR exenatide OR semaglutide OR liraglutide OR albiglutide OR dulaglutide OR tirzepatide OR bexagliflozin OR canagliflozin OR dapagliflozin OR empagliflozin OR ertugliflozin OR ipragliflozin OR luseogliflozin OR remogliflozin OR sergliflozin OR sotagliflozin OR tofogliflozin OR	NA	2025/02/05	210

	henagliflozin OR janagliflozin OR mizagliflozin OR velagliflozin OR enavogliflozin OR licogliflozin OR rongliflozin) AND (random OR randomized OR randomised)			
Embase	(acute kidney injury OR acute renal failure) AND (glucagon-like peptide-1 receptor agonist OR Sodium Glucose Cotransporter 2 Inhibitor) AND (random OR randomized OR randomised)	NA	2025/02/05	714
ProQuest	(acute kidney injury OR acute renal failure) AND (glucagon-like peptide-1 receptor agonist OR Sodium Glucose Cotransporter 2 Inhibitor) AND (random OR randomized OR randomised)	NA	2025/02/05	3579
ScienceDirect	(acute kidney injury OR acute renal failure) AND (glucagon-like peptide-1 receptor agonist OR Sodium Glucose Cotransporter 2 Inhibitor) AND (random OR randomized OR randomised)	NA	2025/02/05	5548
Web of Science	(acute kidney injury OR acute renal failure) AND (glucagon-like peptide-1 receptor agonist OR Sodium Glucose Cotransporter 2 Inhibitor) AND (random OR randomized OR randomised)	NA	2025/02/05	86
ClinicalTrials.gov	(acute kidney injury OR acute renal failure) AND (glucagon-like peptide-1 receptor agonist OR Sodium Glucose Cotransporter 2 Inhibitor OR lixisenatide OR orforglipron OR exenatide OR semaglutide OR liraglutide OR albiglutide OR dulaglutide OR tirzepatide OR bexagliflozin OR canagliflozin OR dapagliflozin OR empagliflozin OR ertugliflozin OR ipragliflozin OR luseogliflozin OR remogliflozin OR sergliflozin OR sotagliflozin OR tofogliflozin OR henagliflozin OR janagliflozin OR mizagliflozin OR velagliflozin OR enavogliflozin OR licogliflozin OR rongliflozin) AND (random OR randomized OR randomised)	NA	2025/02/05	3

Abbreviation: N/A: not applied

Table S3: Excluded studies and reason

Reason	Numbers	References
A trend or significant difference in baseline demographic data which will affect the results	2	[2,3]
A trend or significant difference in baseline Urine albumin/creatinine ratio, which indicate baseline different kidney function	1	[4]
Animal study	1	[5]
Case report	3	[6-8]
Commentary	1	[9]
Duplicate sample source with other included trial	5	[10-14]
Investigate contrast-induced kidney injury, which etiology was significantly different from the acute kidney injury in most other RCTs	1	[15]
Meta-analysis	43	[16-58]
Network meta-analysis	8	[59-66]
Not randomized controlled trial	5	[67-71]
Not report target outcome	75	[72-146]
Review article	6	[147-152]
Study protocol but not result of a clinical trial	1	[153]

Table S4: Characteristics of the included studies

Study name	Baseline illness	Comparison	Subjects	Mean age (years)	Female (%)	Treatment duration	ClinicalTrials.gov	Renal outcome directly reported	Category	Country
Bliddal, H. (2024) (STEP 9)[154]	patients with obesity and knee osteoarthritis	Inject semaglutide 2.4 mg<br>Placebo	271<br>136	56.0±10.0<br>56.0±10.0	84.1<br>76.5	68 weeks	NCT05064735	AKI	GLP-1 agonist	Multiple countries
Natale, P. (2024) (NCT02836873)[155]	patients with type 2 diabetes mellitus and chronic kidney disease	Bexagliflozin 20mg<br>Placebo	157<br>155	69.3±8.4<br>69.9±8.3	41.4<br>32.9	24 weeks	NCT02836873	AKI	SGLT2 inhibitor	Multiple countries
SURMOUNT-J (2024) (NCT04844918)[156]	patients with obesity	Tirzepatide 10-15mg<br>Placebo	150<br>75	50.1±10.6<br>52.3±10.9	41.4<br>40.0	72 weeks	NCT04844918	AKI	GLP-1 agonist	Japan
Tuttle, K.R. (2024) (ASi in CKD) (run-in period)[157]	patients chronic kidney disease, with or without type 2 diabetes mellitus	Empagliflozin 10mg<br>Placebo	298<br>288	63.8±11.6<br>63.9±11.0	36.5<br>30.7	14 weeks	NCT05182840	AKI	SGLT2 inhibitor	Multiple countries
Aroda, V.R. (2023) (AMPLITUDE-D, NCT03684642)[139]	patients with type 2 diabetes mellitus	Efpeglenatide 4.0 mg<br>Efpeglenatide 6.0 mg<br>Dulaglutide 1.5 mg	303<br>302<br>303	60.3±9.6<br>60.0±10.1<br>59.4±10.1	46.9<br>52.0<br>50.5	56 weeks	NCT03684642	AKI	GLP-1 agonist	Multiple countries
Buse, J.B. (2023) (SEPRA)[158]	patients with type 2 diabetes mellitus	Inject semaglutide<br>Control with standard care	644<br>634	57.5±11.3<br>57.2±11.0	48.1<br>43.5	104 weeks	NCT03596450	AKI	GLP-1 agonist	Multiple countries
Cherney, D.Z.I.(2023) (SOTA-CKD3)[159]	patients with type 2 diabetes mellitus and chronic kidney disease	Sotagliflozin<br>Placebo	527<br>260	69.5±7.9<br>69.3±8.1	44.0<br>42.7	26 weeks	NCT03242252	AKI	SGLT2 inhibitor	Multiple countries
Garvey, W.T. (2023) (SURMOUNT-2)[160]	patients with obesity	Tirzepatide 10-15mg<br>Placebo	623<br>315	54.0±10.7<br>54.7±10.5	50.8<br>50.5	72 weeks	NCT04657003	AKI	GLP-1 agonist	Multiple countries
Herrington, W.G. (2023) (EMPA-KIDNEY)[161]	patients with renal failure	Empagliflozin 10mg<br>Placebo	3304<br>3305	63.9±13.9<br>63.8±13.9	33.2<br>33.1	104 weeks	NCT03594110	AKI	SGLT2 inhibitor	Multiple countries
Lincoff, A.M. (2023) (SELECT)[162]	patients with obesity	Inject Semaglutide 2.4mg<br>Placebo	8803<br>8801	61.6±8.9<br>61.6±8.8	27.8<br>27.5	104 weeks	NCT03574597	AKI	GLP-1 agonist	Multiple countries
Rosenstock, J. (2023) (SURPASS-6)[163]	patients with type 2 diabetes mellitus	Tirzepatide 5mg<br>Tirzepatide 10-15mg<br>Controls with insulin lispro	243<br>474<br>708	58.0±10.2<br>58.9±9.5<br>59.0±9.7	59.3<br>59.5<br>55.9	52 weeks	NCT04537923	AKI	GLP-1 agonist	Multiple countries
Wadden, T.A. (2023) (SURMOUNT-3)[164]	patients with obesity	Tirzepatide 10-15mg<br>Placebo	287<br>292	45.4±12.6<br>45.7±11.8	63.1<br>62.7	84 weeks	NCT04657016	AKI	GLP-1 agonist	Multiple countries
Frias, J.P. (2022) (AMPLITUDE-M)[165]	patients with type 2 diabetes mellitus	Efpeglenatide 2 mg<br>Efpeglenatide 4 mg<br>Efpeglenatide 6 mg<br>Placebo	100<br>101<br>103<br>102	58.6±10.5<br>56.3±11.5<br>59.6±10.7<br>59.5±11.7	55.0<br>51.5<br>59.2<br>50.0	62 weeks	NCT03353350	AKI	GLP-1 agonist	Multiple countries

Rubino, D.M. (2022) (STEP 8)[166]	patients with obesity	Inject semaglutide 2.4 mg<br>Liraglutide initial 3.0 mg<br>Placebo	126<br>127<br>85	48.0±14.0<br>49.0±13.0<br>51.0±12.0	81.0<br>76.4<br>77.6	68 weeks	NCT04074161	AKI	GLP-1 agonist	Multiple countries
Solomon, S.D. (2022) (DELIVER)[167]	patients with stabilized heart failure	Dapagliflozin 10mg<br>Placebo	3131<br>3132	71.8±9.6<br>71.5±9.5	43.6<br>44.2	120 weeks	NCT03619213	AKI	SGLT2 inhibitor	Multiple countries
Spertus, J.A. (2022) (CHIEF-HF)[168]	patients with stable symptomatic heart failure	Canagliflozin 100 mg<br>Placebo	222<br>226	62.9±13.2<br>64.0±13.5	46.8<br>42.9	12 weeks	NCT04252287	AKI	SGLT2 inhibitor	USA
Tuttle, K.R. (2022) (NCT01011868)[126]	patients with type 2 diabetes mellitus and chronic kidney disease	Empagliflozin 10mg<br>Empagliflozin 25mg<br>Placebo	169<br>155<br>170	58.6±9.8<br>59.9±10.5<br>58.1±9.4	45.0<br>40.0<br>47.1	78 weeks	NCT01011868	AKI	SGLT2 inhibitor	Multiple countries
Voors, A.A. (2022) (EMPULSE)[169]	patients with acute heart failure and dyspnea	Empagliflozin 10mg<br>Placebo	265<br>265	71.0<br>70.0	32.5<br>35.1	13 weeks	NCT04157751	AKI	SGLT2 inhibitor	Multiple countries
Wada, T. (2022) (TA-7284-14)[170]	patients with type 2 diabetes mellitus and chronic kidney disease	Canagliflozin 100 mg<br>Placebo	154<br>154	62.5±10.5<br>62.4±11.1	25.3<br>16.2	104 weeks	NCT03436693	AKI	SGLT2 inhibitor	Japan
Anker, S.D. (2021) (EMPEROR-Preserved)[171]	patients with heart failure with preserved ejection fraction	Empagliflozin 10mg<br>Placebo	2997<br>2991	71.8±9.3<br>71.9±9.6	44.6<br>44.7	156 weeks	NCT03057951	AKI	SGLT2 inhibitor	Multiple countries
Bhatt, D.L. (2021) (SCORED)[172]	patients with type 2 diabetes mellitus and chronic kidney disease	Sotagliflozin 200-400mg<br>Placebo	5292<br>5292	68.4±8.4<br>68.2±8.4	44.3<br>45.5	116 weeks	NCT03315143	AKI	SGLT2 inhibitor	Multiple countries
Bhatt, D.L. (2021) (SOLOIST-WHF)[173]	patients with type 2 diabetes mellitus and heart failure	Sotagliflozin 200mg<br>Placebo	608<br>614	68.6±9.5<br>69.3±8.8	32.6<br>34.9	36 weeks	NCT03521934	AKI	SGLT2 inhibitor	Multiple countries
Davies, M. (2021) (STEP 2)[174]	patients with type 2 diabetes mellitus and obesity	Inject semaglutide 1.0 mg<br>Inject Semaglutide 2.4 mg<br>Placebo	403<br>404<br>403	56.0±10.0<br>55.0±11.0<br>55.0±11.0	50.4<br>55.2<br>47.1	68 weeks	NCT03552757	AKI	GLP-1 agonist	Multiple countries
Frías, J.P. (2021) (SURPASS-2)[175]	patients with type 2 diabetes mellitus	Tirzepatide 5 mg<br>Tirzepatide 10-15 mg<br>Inject semaglutide 1.0 mg	470<br>939<br>469	56.3±10.0<br>56.5±10.5<br>56.9±10.8	56.4<br>51.9<br>52.0	40 weeks	NCT03987919	AKI	GLP-1 agonist	Multiple countries
Gerstein, H.C. (2021) (AMPLITUDE-O)[176]	patients with type 2 diabetes mellitus	Efpeglenatide 4 mg<br>Efpeglenatide 6 mg<br>Placebo	1359<br>1358<br>1359	64.6±8.2<br>64.7±8.2<br>64.4±8.3	32.5<br>35.6<br>30.8	104 weeks	NCT03496298	AKI	GLP-1 agonist	Multiple countries
Kosiborod, M.N. (2021) (DARE-19)[177]	patients with COVID-19	Dapagliflozin 10mg<br>Placebo	625<br>625	61.0±13.4<br>61.8±13.5	41.6<br>43.7	4 weeks	NCT04350593	AKI	SGLT2 inhibitor	Multiple countries
Lock, J.P. (2021) (BEST) (NCT02558296)[178]	patients with type 2 diabetes mellitus	Bexagliflozin 20mg<br>Placebo	1132<br>567	64.4±7.9<br>64.6±8.0	30.1<br>31.2	52 weeks	NCT02558296	AKI	SGLT2 inhibitor	Multiple countries
Cannon, C.P. (2020) (VERTIS CV)[179]	patients with type 2 diabetes mellitus	Ertugliflozin 5 mg<br>Ertugliflozin 15 mg<br>Placebo	2752<br>2747<br>2747	64.3±8.2<br>64.4±8.0<br>64.4±8.0	29.1<br>30.3<br>30.7	182 weeks	NCT01986881	AKI	SGLT2 inhibitor	Multiple countries
Heerspink, H.J.L. (2020) (DAPA-CKD)[180]	patients with renal failure	Dapagliflozin 10mg<br>Placebo	2152<br>2152	61.8±12.1<br>61.9±12.1	32.9<br>33.3	125 weeks	NCT03036150	AKI	SGLT2 inhibitor	Multiple countries

Packer, M. (2020) (EMPEROR-Reduced)[181]	patients with chronic heart failure	Empagliflozin 10mg<br>Placebo	1863<br>1867	67.2±10.8<br>66.5±11.2	23.5<br>24.4	64 weeks	NCT03057977	AKI	SGLT2 inhibitor	Multiple countries
Gerstein, H.C. (2019) (REWIND)[182]	patients with type 2 diabetes mellitus	Dulaglutide 1.5 mg<br>Placebo	4949<br>4952	66.2±6.5<br>66.2±6.5	46.6<br>46.1	281 weeks	NCT01394952	AKI	GLP-1 agonist	Multiple countries
Husain, M. (2019) (PIONEER 6)[183]	patients with cardiovascular disease or chronic kidney disease	Semaglutide 14mg<br>Placebo	1591<br>1592	66.0±7.0<br>66.0±7.0	31.9<br>31.4	64 weeks	NCT02692716	AKI	GLP-1 agonist	Multiple countries
McMurray, J.J.V. (2019) (DAPA-HF)[184]	patients with stabilized heart failure	Dapagliflozin 10mg<br>Placebo	2373<br>2371	66.2±11.0<br>66.5±10.8	23.8<br>23.0	73 weeks	NCT03036124	AKI	SGLT2 inhibitor	Multiple countries
Mosenzon, O. (2019) (PIONEER 5)[185]	patients with type 2 diabetes mellitus and moderate renal impairment	Semaglutide 14mg<br>Placebo	163<br>161	71.0±8.0<br>70.0±8.0	49.1<br>54.7	31 weeks	NCT02827708	AKI	GLP-1 agonist	Multiple countries
Perkovic, V. (2019) (CREDENCE)[186]	patients with type 2 diabetes mellitus and nephropathy	Canagliflozin 100 mg<br>Placebo	2202<br>2199	62.9±9.2<br>63.2±9.2	34.6<br>33.3	130 weeks	NCT02065791	AKI	SGLT2 inhibitor	Multiple countries
Rodbard, H.W. (2019) (PIONEER 2)[187]	patients with type 2 diabetes mellitus	Semaglutide 14mg<br>Empagliflozin 25mg	411<br>410	57.0±10.0<br>58.0±10.0	49.9<br>49.0	52 weeks	NCT02863328	AKI	GLP-1 agonist/SGLT2 inhibitor	Multiple countries
Rosenstock, J. (2019) (PIONEER 3)[188]	patients with type 2 diabetes mellitus	Oral semaglutide 3-14mg<br>Control with sitagliptin	1396<br>466	57.7±10.0<br>58.0±10.0	46.6<br>49.0	78 weeks	NCT02607865	AKI	GLP-1 agonist	Multiple countries
Wiviott, S.D. (2019) (DECLARE-TIMI 58)[189]	patients with atherosclerotic vascular disease	Dapagliflozin 10mg<br>Placebo	8582<br>8578	63.9±6.8<br>64.0±6.8	36.9<br>37.9	206 weeks	NCT01730534	AKI	SGLT2 inhibitor	Multiple countries
Buse, J.B. (2018) (inTandem1)[190]	patients with type 1 diabetes mellitus	Sotagliflozin<br>Placebo	525<br>268	46.5±13.3<br>45.2±12.72	53.1<br>48.9	52 weeks	NCT02384941	AKI	SGLT2 inhibitor	Multiple countries
Frias, J.P. (2018) (I8F-MC-GPGB)[191]	patients with type 2 diabetes mellitus	Tirzepatide 1-5mg<br>Tirzepatide 10-15mg<br>Dulaglutide 1.5mg<br>Placebo	107<br>104<br>54<br>51	57.7±8.5<br>56.2±8.8<br>58.7±7.8<br>56.6±8.9	41.1<br>50.0<br>55.6<br>43.1	26 weeks	NCT03131687	AKI	GLP-1 agonist	Multiple countries
Grunberger, G. (2018) (VERTIS RENAL)[192]	patients with type 2 diabetes mellitus with chronic kidney disease	Ertugliflozin 5 mg<br>Ertugliflozin 15 mg<br>Placebo	158<br>155<br>154	66.7±8.3<br>67.5±8.5<br>67.5±8.9	46.8<br>51.6<br>53.2	54 weeks	NCT01986855	AKI	SGLT2 inhibitor	Multiple countries
Hernandez, A.F. (2018) (Harmony Outcomes)[193]	patients with type 2 diabetes mellitus	Albiglutide 30-50 mg<br>Placebo	4731<br>4732	64.1±8.7<br>64.2±8.7	30.2<br>31.0	86 weeks	NCT02465515	AKI	GLP-1 agonist	Multiple countries
O'Neil, P.M. (2018) (NCT02453711)[194]	patients with obesity	Inject semaglutide 0.05 to 0.4 mg<br>Liraglutide 3.0 mg<br>Placebo	718<br>103<br>136	46.4±12.6<br>48.5±11.2<br>46.4±12.8	64.6<br>65.0<br>64.7	52 weeks	NCT02453711	AKI	GLP-1 agonist	Multiple countries
Tuttle, K. R. (2018) (AWARD-7)[195]	patients with type 2 diabetes mellitus and moderate-to-severe chronic kidney disease	Dulaglutide 0.75-1.5 mg<br>Control with insulin glargine	367<br>194	64.7±8.7<br>64.3±8.4	45.6<br>52.1	52 weeks	NCT01621178	AKI	GLP-1 agonist	Multiple countries

Aroda, V.R. (2017) (SUSTAIN 4)[196]	patients with type 2 diabetes mellitus	Inject semaglutide 0.5 mg<br>Inject semaglutide 1.0 mg<br>Control with insulin glargine	362<br>360<br>360	56.5±10.3<br>56.7±10.4<br>56.2±10.6	45.6<br>49.4<br>45.8	30 weeks	NCT02128932	AKI	GLP-1 agonist	Multiple countries
Holman, R.R. (2017) (EXSCEL)[197]	patients with type 2 diabetes mellitus	Exenatide 2mg<br>Placebo	7356<br>7396	61.8±9.4<br>61.9±9.4	38.0<br>38.0	166 weeks	NCT01144338	AKI	GLP-1 agonist	Multiple countries
Meneilly, G.S. (2017) (GetGoal-O)[198]	patients with type 2 diabetes mellitus	Lixisenatide 20ug<br>Placebo	176<br>174	74.0±4.0<br>74.4±3.8	47.7<br>48.3	26 weeks	NCT01798706	AKI	GLP-1 agonist	Multiple countries
Neal, B. (2017) (CANVAS)[199]	patients with type 2 diabetes mellitus	Canagliflozin 100 mg<br>Canagliflozin 300 mg<br>Placebo	1445<br>1443<br>1442	62.2±8.0<br>62.8±8.1<br>62.3±7.9	33.5<br>34.6<br>33.7	126 weeks	NCT01032629	AKI	SGLT2 inhibitor	Multiple countries
Neal, B. (2017) (CANVAS-R)[199]	patients with type 2 diabetes mellitus	Canagliflozin 300 mg<br>Placebo	2907<br>2905	63.9±8.4<br>64.0±8.3	36.2<br>38.2	126 weeks	NCT01989754	AKI	SGLT2 inhibitor	Multiple countries
Leiter, L.A. (2016) (D1690C00019)[14]	patients with type 2 diabetes mellitus and cardiovascular disease	Dapagliflozin 10mg<br>Placebo	482<br>482	63.9±7.6<br>63.6±7.0	33.1<br>33.0	80 weeks	NCT01042977	AKI	SGLT2 inhibitor	Multiple countries
Marso, S.P. (2016) (LEADER)[200]	patients with type 2 diabetes mellitus	Liraglutide 1.8mg<br>Placebo	4668<br>4672	64.2±7.2<br>64.4±7.2	35.5<br>36.0	198 weeks	NCT01179048	AKI	GLP-1 agonist	Multiple countries
Marso, S.P. (2016) (SUSTAIN-6)[201]	patients with type 2 diabetes mellitus	Inject semaglutide 0.5 mg<br>Inject semaglutide 1.0 mg<br>Placebo	826<br>822<br>1649	NA	40.1<br>37.0<br>40.0	109 weeks	NCT01720446	AKI	GLP-1 agonist	Multiple countries
Mellander, A. (2016) (NCT00528372)[96]	patients with type 2 diabetes mellitus	Dapagliflozin 2.5-10.0 mg<br>Placebo	410<br>75	NA	51.7<br>58.7	102 weeks	NCT00528372	AKI	SGLT2 inhibitor	Multiple countries
Cefalu, W.T. (2015)[202]	patients with type 2 diabetes mellitus	Dapagliflozin 10mg<br>Placebo	455<br>459	62.8±7.0<br>63.0±7.7	32.1<br>31.4	28 weeks	NCT01031680	AKI	SGLT2 inhibitor	Multiple countries
Pi-Sunyer, X. (2015) (SCALE) (before 56 weeks)[203]	patients with obesity	Liraglutide 3.0mg<br>Placebo	2487<br>1244	45.2±12.1<br>45.0±12.0	78.7<br>78.1	56 weeks	NCT01272219	AKI	GLP-1 agonist	Multiple countries
Zinman, B. (2015) (EMPA-REG OUTCOME)[204]	patients with type 2 diabetes mellitus	Empagliflozin 10mg<br>Empagliflozin 25mg<br>Placebo	2345<br>2342<br>2333	63.0±8.6<br>63.2±8.6<br>63.2±8.8	29.5<br>28.1<br>28.0	135 weeks	NCT01131676	AKI	SGLT2 inhibitor	Multiple countries
Barnett, A.H. (2014) (EMPA-REG RENAL)[205]	patients with type 2 diabetes mellitus and kidney disease	Empagliflozin 10mg<br>Empagliflozin 25mg<br>Placebo	98<br>97<br>95	63.2±8.5<br>62.0±8.4<br>62.6±8.1	38.8<br>37.1<br>41.1	24 weeks	NCT01164501	AKI	SGLT2 inhibitor	Multiple countries
Dungan, K.M. (2014) (AWARD-6)[206]	patients with type 2 diabetes mellitus	Dulaglutide 1.5 mg<br>Liraglutide 1.8 mg	299<br>300	56.5±9.3<br>56.8±9.9	53.8<br>50.3	30 weeks	NCT01624259	AKI	GLP-1 agonist	Multiple countries
Pratley, R.E. (2014) (HARMONY 7)[207]	patients with type 2 diabetes mellitus	Albiglutide 50.0 mg<br>Liraglutide 1.8 mg	404<br>408	55.4±10.0<br>55.8±10.0	52.7<br>46.6	32 weeks	NCT01128894	AKI	GLP-1 agonist	Multiple countries
Ridderstrale, M. (2014) (EMPA-REG H2H-SU)[208]	patients with type 2 diabetes mellitus and moderate-to-severe chronic kidney disease	Empagliflozin 25mg<br>Control with glimepiride	765<br>780	56.2±10.3<br>55.7±10.4	43.5<br>46.0	104 weeks	NCT01167881	AKI	SGLT2 inhibitor	Multiple countries

Stenlöf, K. (2014) (CANTATA-M)[209]	patients with type 2 diabetes mellitus and moderate-to-severe chronic kidney disease	Canagliflozin 100 mg<br>Canagliflozin 300 mg<br>Placebo	195<br>197<br>192	55.1±10.8<br>55.3±10.2<br>55.7±10.9	58.5<br>54.8<br>54.2	52 weeks	NCT01081834	AKI	SGLT2 inhibitor	Multiple countries
Weissman, P.N. (2014) (HARMONY 4)[210]	patients with type 2 diabetes mellitus	Albiglutide 30 mg<br>Control with insulin glargine	504<br>241	55.8±9.3<br>54.7±9.8	43.3<br>45.2	52 weeks	NCT00838916	AKI	GLP-1 agonist	Multiple countries
Lavalle-Gonzalez, F.J. (2013) (data before week 26) (CANTATA-D)[211]	patients with type 2 diabetes mellitus	Canagliflozin 100 mg<br>Canagliflozin 300 mg<br>Placebo	368<br>367<br>183	55.5±9.4<br>55.3±9.2<br>55.3±9.8	52.7<br>55.0<br>48.6	26 weeks	NCT01106677	AKI	SGLT2 inhibitor	Multiple countries
Gallwitz, B. (2012) (EUREXA)[212]	patients with type 2 diabetes mellitus	Exenatide<br>Control with glimepiride	490<br>487	56.0±10.0<br>56.0±9.1	44.5<br>48.3	104 weeks	NCT00359762	AKI	GLP-1 agonist	Multiple countries
Gallwitz, B. (2011) (H8O-SB-GWBN)[213]	patients with type 2 diabetes mellitus	Exenatide 5-10 mcg<br>Control with insulin aspart	181<br>173	57.2±10.0<br>56.9±9.9	40.3<br>44.5	26 weeks	NCT00434954	AKI	GLP-1 agonist	Germany
Bailey, C.J. (2010) (MB102-014)[214]	patients with type 2 diabetes mellitus	Dapagliflozin 2.5-10.0 mg<br>Placebo	409<br>137	54.0±9.6<br>53.7±10.3	47.2<br>44.5	24 weeks	NCT00528879	AKI	SGLT2 inhibitor	Multiple countries
Nauck, M. (2009) (LEAD-2)[215]	patients with type 2 diabetes mellitus	Liraglutide<br>Placebo	724<br>121	56.7±9.7<br>56.0±9.0	41.7<br>40.0	26 weeks	NCT00318461	AKI	GLP-1 agonist	Multiple countries

Abbreviations: GLP-1 agonist: glucagon-like peptide-1 agonist; NA: not available; SGLT2 inhibitor: sodium–glucose cotransporter 2 inhibitor

Table S5: League table of NMA of safety profile: drop-out rate

01_Tirzepatide _low_dosage	0.96 [0.63; 1.46]		0.72 [0.37; 1.41]									1.23 [0.39; 3.82]								0.56 [0.32; 0.97]			
0.91 (0.6, 1.38)	02_Tirzepatide _high_dosage		0.98 [0.55; 1.73]									1.26 [0.41; 3.95]								0.59 [0.45; 0.78]			
0.81 (0.49, 1.34)	0.89 (0.61, 1.29)	03_ Canagliflozin _high_dosage		0.91 [0.67; 1.22]																0.68 [0.53; 0.89]			
0.82 (0.5, 1.33)	0.9 (0.61, 1.32)	1.01 (0.65, 1.57)	04_Inject_ semaglutide_ medium_ dosage				1.00 [0.43; 2.31]				0.73 [0.43; 1.23]									0.72 [0.47; 1.11]			
0.73 (0.45, 1.2)	0.8 (0.56, 1.15)	0.91 (0.68, 1.21)	0.9 (0.58, 1.37)	05_ Canagliflozin _low_dosage																0.76 [0.60; 0.96]			
0.77 (0.38, 1.55)	0.84 (0.46, 1.55)	0.95 (0.52, 1.75)	0.94 (0.49, 1.8)	1.05 (0.58, 1.92)	06_ Bexagliflozin															0.75 [0.45; 1.26]			
0.68 (0.42, 1.11)	0.75 (0.53, 1.07)	0.84 (0.59, 1.22)	0.83 (0.55, 1.27)	0.93 (0.66, 1.32)	0.89 (0.49, 1.59)	07_ Sotagliflozin														0.84 [0.68; 1.03]			
0.68 (0.41, 1.15)	0.74 (0.5, 1.12)	0.84 (0.56, 1.27)	0.83 (0.53, 1.31)	0.92 (0.63, 1.38)	0.88 (0.47, 1.64)	1 (0.68, 1.47)	08_Inject_ semaglutide _high_dosage							0.66 [0.22; 1.98]						0.87 [0.65; 1.15]			
0.69 (0.32, 1.55)	0.76 (0.37, 1.59)	0.86 (0.42, 1.79)	0.85 (0.4, 1.83)	0.94 (0.46, 1.97)	0.9 (0.38, 2.15)	1.01 (0.5, 2.11)	1.02 (0.49, 2.16)	09_ Efpeglenatide _low_dosage								0.75 [0.36; 1.58]				0.85 [0.40; 1.80]			
0.66 (0.42, 1.04)	**0.72 (0.53, 0.99)**	0.81 (0.59, 1.13)	0.8 (0.55, 1.19)	0.9 (0.67, 1.22)	0.86 (0.49, 1.51)	0.97 (0.72, 1.29)	0.97 (0.68, 1.37)	0.95 (0.47, 1.9)	10_ Empagliflozin _low_dosage								0.90 [0.68; 1.18]			0.86 [0.75; 0.99]			
0.66 (0.41, 1.07)	0.73 (0.51, 1.03)	0.82 (0.57, 1.16)	0.81 (0.53, 1.22)	0.9 (0.65, 1.26)	0.86 (0.47, 1.54)	0.97 (0.69, 1.34)	0.98 (0.66, 1.42)	0.96 (0.46, 1.94)	1.01 (0.75, 1.32)	11_ Dapagliflozin										0.85 [0.69; 1.05]			
0.64 (0.37, 1.13)	0.7 (0.44, 1.11)	0.79 (0.49, 1.27)	0.78 (0.49, 1.24)	0.88 (0.55, 1.39)	0.83 (0.43, 1.63)	0.94 (0.6, 1.48)	0.95 (0.58, 1.53)	0.93 (0.42, 2.02)	0.97 (0.64, 1.48)	0.97 (0.62, 1.52)	12_ Inject_ semaglutide _low_dosage			0.92 [0.38; 2.20]						0.88 [0.59; 1.32]			
0.64 (0.39, 1.04)	0.7 (0.47, 1.03)	0.79 (0.53, 1.18)	0.78 (0.5, 1.23)	0.87 (0.59, 1.28)	0.83 (0.44, 1.54)	0.93 (0.64, 1.37)	0.94 (0.61, 1.43)	0.92 (0.45, 1.86)	0.97 (0.68, 1.35)	0.96 (0.66, 1.41)	0.99 (0.61, 1.62)	13_ Dulaglutide		0.97 [0.53; 1.77]		1.04 [0.67; 1.63]				1.02 [0.67; 1.56]		0.68 [0.44; 1.06]	
0.64 (0.38, 1.06)	0.7 (0.47, 1.03)	0.79 (0.53, 1.17)	0.78 (0.5, 1.21)	0.87 (0.6, 1.26)	0.83 (0.45, 1.53)	0.93 (0.64, 1.35)	0.94 (0.61, 1.42)	0.92 (0.44, 1.88)	0.96 (0.69, 1.34)	0.96 (0.67, 1.39)	0.99 (0.62, 1.59)	1 (0.66, 1.5)	14_ Albiglutide	0.84 [0.52; 1.36]						0.94 [0.71; 1.26]			
**0.62 (0.39, 0.98)**	**0.68 (0.48, 0.94)**	0.77 (0.54, 1.07)	0.75 (0.5, 1.13)	0.84 (0.61, 1.16)	0.8 (0.45, 1.44)	0.91 (0.65, 1.24)	0.91 (0.62, 1.3)	0.89 (0.43, 1.78)	0.94 (0.7, 1.22)	0.93 (0.68, 1.28)	0.96 (0.63, 1.48)	0.97 (0.69, 1.37)	0.97 (0.7, 1.34)	15_ Liraglutide						0.87 [0.70; 1.07]			
0.6 (0.35, 1.06)	0.66 (0.42, 1.04)	0.75 (0.47, 1.18)	0.74 (0.45, 1.22)	0.82 (0.53, 1.28)	0.79 (0.4, 1.49)	0.89 (0.57, 1.35)	0.89 (0.55, 1.42)	0.87 (0.4, 1.85)	0.92 (0.61, 1.36)	0.91 (0.59, 1.41)	0.94 (0.55, 1.6)	0.95 (0.59, 1.51)	0.95 (0.59, 1.51)	0.98 (0.64, 1.5)	16_Ertugliflozin _low_dosage				0.94 [0.69; 1.29]	0.94 [0.69; 1.28]			
0.6 (0.36, 1.01)	**0.66 (0.44, 0.99)**	0.74 (0.49, 1.13)	0.73 (0.46, 1.17)	0.82 (0.55, 1.22)	0.78 (0.42, 1.45)	0.88 (0.59, 1.3)	0.88 (0.57, 1.36)	0.87 (0.44, 1.69)	0.91 (0.64, 1.3)	0.91 (0.62, 1.34)	0.94 (0.57, 1.54)	0.94 (0.66, 1.34)	0.94 (0.62, 1.44)	0.97 (0.67, 1.42)	1 (0.62, 1.62)	17_ Efpeglenatide_ medium_ dosage				1.00 [0.73; 1.37]			
**0.6 (0.37, 0.97)**	**0.66 (0.46, 0.93)**	0.75 (0.52, 1.05)	0.74 (0.49, 1.11)	0.82 (0.58, 1.14)	0.79 (0.43, 1.4)	0.89 (0.62, 1.22)	0.89 (0.59, 1.28)	0.87 (0.42, 1.76)	0.92 (0.7, 1.17)	0.91 (0.66, 1.25)	0.94 (0.6, 1.46)	0.95 (0.65, 1.37)	0.95 (0.65, 1.36)	0.98 (0.71, 1.33)	1 (0.64, 1.53)	1.01 (0.67, 1.47)	18_ Empagliflozin_ high_dosage			0.83 [0.67; 1.03]	2.16 [0.98; 4.76]		

0.58 (0.24, 1.37)	0.63 (0.29, 1.41)	0.72 (0.32, 1.61)	0.71 (0.31, 1.6)	0.79 (0.36, 1.74)	0.76 (0.3, 1.9)	0.85 (0.39, 1.86)	0.85 (0.38, 1.91)	0.83 (0.3, 2.3)	0.88 (0.41, 1.89)	0.88 (0.4, 1.91)	0.9 (0.39, 2.1)	0.91 (0.41, 2.06)	0.91 (0.41, 2.04)	0.94 (0.43, 2.06)	0.96 (0.42, 2.2)	0.96 (0.43, 2.18)	0.96 (0.44, 2.11)	19_ Lixisenatide		0.99 [0.49; 2.01]			
**0.57 (0.33, 0.99)**	**0.62 (0.4, 0.97)**	0.7 (0.44, 1.11)	0.69 (0.42, 1.15)	0.77 (0.5, 1.2)	0.74 (0.38, 1.4)	0.83 (0.53, 1.28)	0.84 (0.51, 1.34)	0.82 (0.37, 1.76)	0.86 (0.57, 1.28)	0.86 (0.56, 1.32)	0.88 (0.52, 1.5)	0.89 (0.55, 1.42)	0.89 (0.56, 1.42)	0.92 (0.6, 1.41)	0.94 (0.65, 1.36)	0.95 (0.58, 1.52)	0.94 (0.62, 1.46)	0.98 (0.43, 2.25)	20_Ertugliflozin _high_dosage	1.00 [0.73; 1.36]			
**0.57 (0.37, 0.87)**	**0.62 (0.48, 0.82)**	**0.71 (0.54, 0.93)**	**0.7 (0.49, 0.99)**	0.78 (0.61, 1)	0.74 (0.43, 1.27)	0.84 (0.66, 1.06)	0.84 (0.62, 1.14)	0.83 (0.41, 1.61)	0.87 (0.73, 1.02)	0.86 (0.69, 1.09)	0.89 (0.6, 1.31)	0.9 (0.67, 1.21)	0.9 (0.67, 1.2)	0.92 (0.75, 1.15)	0.95 (0.66, 1.37)	0.95 (0.7, 1.3)	0.94 (0.76, 1.21)	0.98 (0.46, 2.09)	1 (0.7, 1.46)	21_Placebo _or_Control	0.66 [0.40; 1.10]	1.01 [0.74; 1.39]	0.89 [0.71; 1.12]
**0.54 (0.3, 0.99)**	**0.59 (0.36, 0.99)**	0.67 (0.4, 1.13)	0.66 (0.38, 1.16)	0.74 (0.45, 1.22)	0.71 (0.36, 1.4)	0.8 (0.49, 1.3)	0.8 (0.47, 1.35)	0.78 (0.35, 1.73)	0.82 (0.52, 1.3)	0.82 (0.51, 1.35)	0.85 (0.48, 1.49)	0.85 (0.5, 1.45)	0.85 (0.51, 1.43)	0.88 (0.55, 1.43)	0.9 (0.51, 1.59)	0.9 (0.53, 1.54)	0.9 (0.57, 1.43)	0.94 (0.4, 2.23)	0.96 (0.55, 1.69)	0.95 (0.62, 1.46)	22_ Oral_ semaglutide		
**0.52 (0.31, 0.88)**	**0.57 (0.39, 0.86)**	**0.65 (0.43, 0.98)**	0.64 (0.4, 1.02)	0.71 (0.48, 1.06)	0.68 (0.36, 1.26)	0.77 (0.52, 1.13)	0.77 (0.5, 1.18)	0.76 (0.38, 1.47)	0.79 (0.56, 1.13)	0.79 (0.54, 1.17)	0.81 (0.5, 1.33)	0.82 (0.58, 1.17)	0.82 (0.54, 1.25)	0.85 (0.59, 1.23)	0.87 (0.54, 1.41)	0.87 (0.65, 1.17)	0.87 (0.6, 1.29)	0.91 (0.4, 2.03)	0.92 (0.57, 1.5)	0.92 (0.67, 1.25)	0.97 (0.57, 1.64)	23_ Efpeglenatide _high_dosage	
**0.5 (0.31, 0.83)**	**0.55 (0.38, 0.8)**	**0.62 (0.43, 0.91)**	**0.62 (0.4, 0.95)**	**0.69 (0.48, 0.99)**	0.65 (0.36, 1.19)	0.74 (0.51, 1.05)	0.74 (0.49, 1.1)	0.73 (0.35, 1.49)	0.76 (0.55, 1.03)	0.76 (0.54, 1.08)	0.78 (0.49, 1.26)	0.79 (0.53, 1.17)	0.79 (0.54, 1.17)	0.81 (0.58, 1.15)	0.84 (0.53, 1.31)	0.84 (0.55, 1.26)	0.83 (0.59, 1.19)	0.87 (0.39, 1.92)	0.89 (0.57, 1.4)	0.88 (0.67, 1.14)	0.93 (0.56, 1.53)	0.96 (0.64, 1.44)	24_ Exenatide

Data presents as OR [95%CIs]. Pairwise (upper-right portion) and network (lower-left portion) meta-analysis results are presented as estimate effect sizes for the outcome of safety profile (drop-out rate). Interventions are reported in order of mean ranking of safety, and outcomes are expressed as odds ratio (OR) (95% confidence intervals) (95%CIs). For the pairwise meta-analyses, OR of less than 1 indicates that the treatment specified in the row got more safety than that specified in the column. For the network meta-analysis (NMA), OR of less than 1 indicates that the treatment specified in the column got more safety than that specified in the row. Bold results indicate statistical significance.

Abbreviation: 95%CIs: 95% confidence intervals; GLP-1 agonist: glucagon-like peptide-1 agonist; NMA: network meta-analysis; OR: odds ratio; RCT: randomized controlled trial; SGLT2 inhibitor: sodium–glucose cotransporter 2 inhibitor

Table S6A: SUCRA (Surface under the cumulative ranking) of primary outcome: overall acute kidney injury/acute renal failure

Treatment	Rank 1	Rank 2	Rank 3	Rank 4	Rank 5	Rank 6	Rank 7	Rank 8	Rank 9	Rank 10	Rank 11	Rank 12	Rank 13	Rank 14	Rank 15	Rank 16	Rank 17	Rank 18	Rank 19	Rank 20	Rank 21	Rank 22	Rank 23	Rank 24	SUCRA
Albiglutide	0	0.0002125	0.0035125	0.011225	0.01395	0.025375	0.0376625	0.0576125	0.0719125	0.0808	0.0825875	0.0858	0.0867875	0.0854625	0.0659375	0.0582375	0.061675	0.0569125	0.0442375	0.0316625	0.0220375	0.0127125	0.0035625	0.000125	47.75951087
Bexagliflozin	0	0	0.0001625	0.00235	0.005025	0.0085875	0.01075	0.0109	0.012725	0.0157125	0.0180375	0.020375	0.02625	0.0294125	0.0308125	0.0318125	0.04245	0.0511125	0.074875	0.093875	0.1317	0.1719	0.194675	0.0165	21.51918478
Canagliflozin_high_dosage	0.0003375	0.0378375	0.1091125	0.281775	0.2338625	0.134575	0.0648875	0.0370625	0.030675	0.019225	0.018675	0.01155	0.007875	0.0047	0.00275	0.001625	0.0011125	0.000825	0.0006375	0.0005625	3.00E-04	3.75E-05	0	0	81.00021739
Canagliflozin_low_dosage	0	0	5.00E-05	0.00025	0.0011875	0.0039	0.0159125	0.0175875	0.02845	0.0479875	0.078825	0.126775	0.1202875	0.1147	0.1057375	0.098175	0.0770625	0.0693375	0.044725	0.0262	0.0145125	0.0063875	0.001925	2.50E-05	42.80282609
Dapagliflozin	0	6.25E-05	0.00095	0.007875	0.027625	0.07305	0.1468625	0.196925	0.1927375	0.1605875	0.10555	0.0513625	0.021925	0.0093375	0.00345	0.001175	4.00E-04	8.75E-05	3.75E-05	0	0	0	0	0	65.96679348
Dulaglutide	0	0	0.0002125	0.000925	0.002875	0.006	0.010175	0.0162	0.02545	0.0417625	0.0579625	0.0800625	0.0929625	0.08505	0.0905125	0.0880875	0.087675	0.098125	0.0907875	0.0654	0.0334	0.020825	0.005475	7.50E-05	38.07038043
Efpeglenatide_high_dosage	0	4.00E-04	0.0022625	0.0109375	0.0172125	0.019	0.020325	0.0223875	0.0171375	0.0182625	0.0216625	0.0266625	0.0282875	0.0278125	0.02905	0.0280375	0.0351625	0.046825	0.0602	0.0910125	0.1296125	0.2013625	0.1391	0.0072875	25.91288043
Efpeglenatide_low_dosage	0.3584625	0.2595	0.1562625	0.03665	0.015675	0.0121125	0.007175	0.006275	0.005325	0.0053	0.0063375	0.0046	0.004175	0.0040875	0.0028875	0.002875	0.0042	0.0042125	0.005075	0.006525	0.0114125	0.022675	0.0486625	0.0095375	84.20967391
Efpeglenatide_medium_dosage	0	0	0.00115	0.003875	0.0076	0.012325	0.0143875	0.01985	0.015625	0.0186625	0.02235	0.023075	0.0303375	0.0263375	0.024675	0.033825	0.039325	0.0478625	0.058875	0.087275	0.1465	0.1838875	0.171725	0.010475	23.33858696
Empagliflozin_high_dosage	0	0.007025	0.0510625	0.073725	0.1615125	0.1861375	0.1530625	0.0899875	0.0626	0.053	0.0371125	0.0285375	0.0315625	0.0284375	0.01165	0.0089375	0.006025	0.0040875	0.0025875	0.001525	0.001025	0.0003375	6.25E-05	0	72.02527174
Empagliflozin_low_dosage	0	1.25E-05	0.0022875	0.0058375	0.0206375	0.068825	0.107075	0.1629875	0.1947125	0.1655125	0.11575	0.0866125	0.042625	0.0166625	0.0061125	0.0027875	0.0009625	0.0003875	0.0001875	1.25E-05	1.25E-05	0	0	0	64.03896739
Ertugliflozin_high_dosage	0	0.001875	0.0145875	0.0439375	0.089625	0.103625	0.09675	0.08425	0.0614625	0.0616125	0.072775	0.05775	0.0526	0.0467375	0.0364375	0.030375	0.030075	0.0307875	0.029025	0.0251	0.0184625	0.0080625	0.0033	0.0007875	58.88391304
Ertugliflozin_low_dosage	0	0.000225	0.00045	0.001775	0.0076375	0.0141125	0.0159625	0.0188125	0.0216	0.0253375	0.034225	0.0418875	0.0518875	0.052975	0.0585	0.0588875	0.07175	0.0783875	0.0927375	0.101275	0.1023	0.0884625	0.0542125	0.0066	30.97918478
Exenatide	0.0358375	0.12765	0.280025	0.22995	0.0624375	0.0374375	0.0275125	0.0172625	0.014025	0.01295	0.0123875	0.0119375	0.0088125	0.0070625	0.00705	0.00675	0.007525	0.0086	0.010875	0.01655	0.0191	0.017275	0.015125	0.0058625	78.38690217
Inject_semaglutide_high_dosage	0	8.75E-05	0.001275	0.011075	0.02315	0.0474125	0.07235	0.09125	0.1089625	0.1185875	0.1404	0.1088625	0.0862625	0.0616	0.04575	0.0303125	0.0189625	0.012575	0.0086375	0.006375	0.0036	0.00175	0.0007375	2.50E-05	57.2638587
Inject_semaglutide_low_dosage	0	0	7.50E-05	0.0004625	0.0026125	0.0062625	0.0187	0.0122625	0.0181625	0.0212875	0.0223	0.0322125	0.0548125	0.0528375	0.04915	0.0525375	0.0612125	0.0745625	0.0911375	0.1292625	0.117975	0.1116625	0.06775	0.0027625	27.92070652
Inject_semaglutide_medium_dosage	8.75E-05	0.0099875	0.075075	0.180075	0.207275	0.1610125	0.1059625	0.055975	0.0413875	0.0388625	0.02945	0.023275	0.0192	0.013825	0.0091875	0.009575	0.0065125	0.0050875	0.0036125	0.0019	0.0017	0.0007375	0.0002375	0	75.55472826
Liraglutide	0	0	5.00E-05	0.0004125	0.0006375	0.0012375	0.002625	0.004175	0.00665	0.0111	0.0157375	0.0271375	0.0381875	0.049725	0.06485	0.093225	0.1208625	0.1515875	0.1531875	0.1253625	0.0792375	0.0398	0.013925	0.0002875	28.44108696
Lixisenatide	0.2738875	0.45095	0.11315	0.02725	0.0101	0.0069125	0.004475	0.0034	0.0030875	0.0027125	0.0023375	0.0029	0.00225	0.0021125	0.0020125	0.0022875	0.002625	0.003075	0.0045375	0.00665	0.0185	0.010775	0.021725	0.0222875	86.97788043

Oral_semaglutide	0	0.0011375	0.0075	0.0224625	0.0656	0.0590875	0.055625	0.0602	0.0496875	0.052675	0.05735	0.0608	0.0571375	0.0572625	0.0500125	0.0458	0.0602	0.053425	0.0497625	0.046025	0.041075	0.0284625	0.017875	0.0008375	48.69141304
Placebo_or_Control	0	0	0	0	0	0	0	3.75E-05	3.00E-04	0.0016875	0.0079375	0.0335375	0.0767125	0.1512875	0.225925	0.212675	0.15365	0.0833125	0.0398	0.010175	0.0027375	0.000225	0	0	36.73733696
Sotagliflozin	0	0	0	3.00E-04	0.0010125	0.0025125	0.004725	0.0079125	0.0116375	0.017775	0.028725	0.047375	0.0520125	0.066225	0.0722	0.095375	0.105725	0.1126625	0.1251125	0.1162125	0.0806375	0.0386125	0.0126375	0.0006125	30.99195652
Tirzepatide_high_dosage	0	0	0	0	0	0	1.25E-05	2.50E-05	1.00E-04	0.0001875	0.00015	0.0001875	0.0004375	0.0006375	0.0007375	0.001	0.001375	0.0020625	0.002875	0.0034375	0.0057875	0.0153	0.0575625	0.908125	0.827880435
Tirzepatide_low_dosage	0.3313875	0.1030375	0.1807875	0.046875	0.02275	0.0105	0.007025	0.0066625	0.0055875	0.0084125	0.011375	0.006725	0.0066125	0.0057125	0.0046125	0.005625	0.003475	0.0041	0.006475	0.007625	0.018375	0.01875	0.169725	0.0077875	71.6988587

Table S6B: SUCRA (Surface under the cumulative ranking) of primary outcome: acute kidney injury/acute renal failure in the subgroup focusing RCTs without definite underlying kidney failure

Treatment	Rank 1	Rank 2	Rank 3	Rank 4	Rank 5	Rank 6	Rank 7	Rank 8	Rank 9	Rank 10	Rank 11	Rank 12	Rank 13	Rank 14	Rank 15	Rank 16	Rank 17	Rank 18	Rank 19	Rank 20	Rank 21	Rank 22	Rank 23	Rank 24	SUCRA
Albiglutide	0	0	0.000475	0.0026375	0.0087	0.0192375	0.0255375	0.034225	0.0438125	0.055275	0.0696625	0.08725	0.104875	0.0962	0.0835125	0.0732625	0.0676125	0.06815	0.0664875	0.0481375	0.0306125	0.0113125	0.0029	0.000125	43.11983696
Bexagliflozin	0	0	0.0031	0.0143375	0.023	0.0279	0.0321375	0.0221	0.0199625	0.0194875	0.022775	0.02575	0.027925	0.028575	0.0445125	0.0336125	0.0429875	0.049425	0.06165	0.0793625	0.121725	0.138375	0.1397125	0.0215875	28.85027174
Canagliflozin_high_dosage	0	0.000175	0.031375	0.1885875	0.237425	0.171625	0.1074625	0.080925	0.04475	0.036825	0.028	0.0220875	0.01545	0.01155	0.0083125	0.0056875	0.0035125	0.002575	0.0014875	9.00E-04	7.00E-04	0.000525	6.25E-05	0	75.79288043
Canagliflozin_low_dosage	0	0	0.00175	0.01145	0.02415	0.0351	0.047725	0.0507	0.0493125	0.0519125	0.051875	0.058925	0.05935	0.0605125	0.056875	0.064425	0.0682875	0.0713375	0.0738	0.073225	0.0505375	0.0268625	0.01185	3.75E-05	43.03146739
Dapagliflozin	0	0	0.0011625	0.00765	0.023925	0.07705	0.1354375	0.1764625	0.1740375	0.1501875	0.1046125	0.065725	0.0403	0.0221625	0.011475	0.004725	0.0024125	0.001375	0.000825	0.000325	0.0001125	3.75E-05	0	0	64.53717391
Dulaglutide	0	0	0.000475	0.003425	0.0070875	0.011225	0.0162125	0.0236375	0.0366	0.0471375	0.066225	0.1033	0.1003	0.1106375	0.099175	0.0960875	0.083225	0.0699	0.0578875	0.038525	0.0188125	0.0084625	0.00165	1.25E-05	42.47793478
Efpeglenatide_high_dosage	0	0	0.0020625	0.0034125	0.0088125	0.0121	0.024425	0.016275	0.0140875	0.0160875	0.0208875	0.0308875	0.033425	0.03165	0.0319375	0.0346	0.0441	0.0534625	0.0848875	0.11995	0.1743375	0.167175	0.0699875	0.00545	25.91576087
Efpeglenatide_low_dosage	0.3741125	0.3632	0.143275	0.054925	0.003775	0.002275	0.002175	0.0012	0.00115	0.0013	0.0012875	0.001775	0.0012875	0.0012125	0.0017625	0.0022875	0.001825	0.0021875	0.00335	0.002475	0.0064375	0.009525	0.0113625	0.0058375	91.77097826
Efpeglenatide_medium_dosage	0	0	3.00E-04	0.0012375	0.00535	0.006975	0.0078875	0.0083	0.0098875	0.010925	0.01865	0.0221625	0.0357375	0.033	0.0348	0.0303375	0.039775	0.0643375	0.0866125	0.124575	0.1745375	0.185675	0.086825	0.0121125	22.5713587
Empagliflozin_high_dosage	0	5.00E-05	0.0281625	0.08515	0.1774	0.1626375	0.1256125	0.096375	0.0813	0.0554375	0.0448625	0.03635	0.0297	0.0212625	0.015325	0.0113	0.0088125	0.0086625	0.0055375	0.0032125	0.002175	0.000525	0.00015	0	70.50266304
Empagliflozin_low_dosage	0	0	0.0004125	0.0052375	0.01615	0.0404375	0.0939125	0.1317875	0.15095	0.169825	0.1361375	0.0984375	0.070275	0.0411125	0.023875	0.0117	0.0048875	0.0026625	0.0014125	0.0005875	0.0001375	5.00E-05	1.25E-05	0	60.86451087
Ertugliflozin_high_dosage	0	1.25E-05	0.005375	0.036475	0.0663	0.0793125	0.0840875	0.0820625	0.07005	0.0714625	0.080775	0.06505	0.062675	0.053225	0.0477125	0.0418375	0.03695	0.033525	0.0308	0.02545	0.0169125	0.0071625	0.00265	0.0001375	56.18896739
Ertugliflozin_low_dosage	0	0	0.0004625	0.003225	0.0094875	0.017	0.0245625	0.0346	0.0370125	0.046275	0.0512125	0.0617375	0.069225	0.0803125	0.0661625	0.070125	0.0744	0.08155	0.0785625	0.0824	0.0605125	0.0381125	0.0122	0.0008625	38.54271739
Exenatide	0.0002875	0.0483875	0.274825	0.3321375	0.0549125	0.0305125	0.0235125	0.0179125	0.0143625	0.0122625	0.0111625	0.01025	0.0091375	0.0089	0.0091125	0.01125	0.0137375	0.0126125	0.0134625	0.01825	0.01655	0.020425	0.0339625	0.002075	74.85847826
Inject_semaglutide_high_dosage	0	0	0.000825	0.0034125	0.0105	0.0262625	0.0414625	0.06405	0.101175	0.1041625	0.124175	0.1184	0.1029	0.0868875	0.0682	0.045575	0.033275	0.0253625	0.02005	0.0123625	0.0064375	0.0033125	0.0011	0.0001125	52.34543478
Inject_semaglutide_low_dosage	0	0	7.50E-05	0.00075	0.0019	0.0040625	0.007525	0.0104375	0.0151	0.0176625	0.0247875	0.029725	0.036975	0.041875	0.050825	0.0598125	0.0801	0.0926375	0.1330125	0.1352625	0.1322375	0.0927875	0.03095	0.0015	27.26396739
Inject_semaglutide_medium_dosage	0	0.000125	0.0446875	0.119925	0.207725	0.1574875	0.1002625	0.070025	0.0555875	0.050625	0.0440625	0.0353875	0.0272375	0.020725	0.0163	0.0138125	0.011825	0.0089625	0.0064	0.004825	0.00265	0.001175	0.0001875	0	71.51532609
Liraglutide	0	0	2.50E-05	0.000525	0.001925	0.0038375	0.005525	0.0064875	0.00935	0.01305	0.01975	0.027225	0.04105	0.056375	0.077675	0.0971875	0.128	0.1588125	0.1333375	0.1243625	0.0618875	0.027775	0.0055375	3.00E-04	30.31494565
Lixisenatide	0.3053625	0.3802375	0.20915	0.0381125	0.00835	0.0052	0.0039375	0.0025	0.002725	0.00225	0.00215	0.00215	0.002	0.0023	0.0018375	0.0019125	0.002225	0.002225	0.0026875	0.0038625	0.0029375	0.0057	0.00355	0.0066375	92.11603261

Oral_semaglutide	0	8.75E-05	0.0036375	0.014725	0.016575	0.012525	0.010075	0.0090375	0.008025	0.0088	0.009125	0.0121625	0.0120625	0.0116875	0.013175	0.0127375	0.01655	0.023375	0.0315875	0.037425	0.0599375	0.1374875	0.3796	0.1596	15.67554348
Placebo_or_Control	0	0	0	0	0	0	0	2.50E-05	0.000125	0.0008125	0.0054625	0.0182375	0.0564375	0.1218125	0.1915875	0.2304375	0.182425	0.1188375	0.0517375	0.0177375	0.003625	0.0006375	6.25E-05	0	34.96559783
Sotagliflozin	0	3.75E-05	0.0113875	0.045175	0.0787125	0.091625	0.0755625	0.057125	0.057125	0.054925	0.0589875	0.0631	0.056625	0.05395	0.0422	0.0424	0.04835	0.041975	0.0456875	0.0319	0.0257625	0.013675	0.0035125	2.00E-04	54.66320652
Tirzepatide_high_dosage	0	0	0	0	2.50E-05	0.0001625	0.000125	0.00015	0.000125	0.0001625	0.000225	0.0003125	0.0004875	0.000525	7.00E-04	0.0009375	0.0010125	0.0016125	0.00205	0.005525	0.0163875	0.0353625	0.151	0.7831125	1.578967391
Tirzepatide_low_dosage	0.3202375	0.2076875	0.237	0.0274875	0.0078125	0.00545	0.0048375	0.0036	0.0033875	0.00315	0.00315	0.0036125	0.0045625	0.00355	0.00295	0.00395	0.0037125	0.0044375	0.0066875	0.0093625	0.0140375	0.0678625	0.051175	3.00E-04	80.53597826

Table S6C: SUCRA (Surface under the cumulative ranking) of safety profile: drop-out rate

Treatment	Rank 1	Rank 2	Rank 3	Rank 4	Rank 5	Rank 6	Rank 7	Rank 8	Rank 9	Rank 10	Rank 11	Rank 12	Rank 13	Rank 14	Rank 15	Rank 16	Rank 17	Rank 18	Rank 19	Rank 20	Rank 21	Rank 22	Rank 23	Rank 24	SUCRA
Albiglutide	0.0016875	0.0048	0.0106375	0.0197125	0.027875	0.0392875	0.0470625	0.0528625	0.0569625	0.06095	0.0632375	0.0643625	0.0661375	0.06385	0.0627125	0.0586	0.053725	0.050625	0.0468875	0.0437	0.03925	0.032775	0.0219125	0.0103875	46.26255435
Bexagliflozin	0.119925	0.099375	0.0872625	0.0795	0.06815	0.0591625	0.0498875	0.0438625	0.038075	0.0324875	0.0304125	0.0270625	0.0283	0.0257125	0.02285	0.0220375	0.0197875	0.0203375	0.0200375	0.02125	0.02115	0.021675	0.022	0.0197	67.59853261
Canagliflozin_high_dosage	0.0490125	0.0978375	0.134375	0.1519875	0.141325	0.1126625	0.08225	0.0586875	0.0426125	0.0321	0.0248125	0.0192375	0.015025	0.0107875	0.0084625	0.0057	0.004375	0.0028375	0.002375	0.001475	0.0011875	0.0006125	0.000225	3.75E-05	79.56103261
Canagliflozin_low_dosage	0.0075125	0.0212125	0.047525	0.07905	0.1107625	0.1178625	0.111475	0.0976625	0.079575	0.06775	0.0559	0.046625	0.0379625	0.03095	0.025225	0.0188375	0.0141625	0.01	0.0076375	0.005425	0.0031875	0.00245	0.0009375	0.0003125	68.12929348
Dapagliflozin	9.00E-04	0.0034625	0.0095625	0.020775	0.0358125	0.0508625	0.0636875	0.0726125	0.0804125	0.0804625	0.0796125	0.07765	0.0728125	0.0675875	0.05945	0.0535375	0.0439875	0.0359625	0.029725	0.02455	0.017575	0.01125	0.0055125	0.0022375	52.80206522
Dulaglutide	0.001575	0.005075	0.0119125	0.0218	0.0316125	0.0415	0.0498	0.05595	0.05785	0.0596	0.0612375	0.0627625	0.0624125	0.0637	0.0616375	0.0566125	0.054175	0.0507	0.0480375	0.0445625	0.0403125	0.0304125	0.0189625	0.0078	46.99402174
Efpeglenatide_high_dosage	0	0.0001625	0.0004375	0.0008625	0.0016625	0.0029625	0.004725	0.0060125	0.0083375	0.0114375	0.014525	0.0184	0.02185	0.027325	0.0332	0.039925	0.0459875	0.053725	0.0684	0.08925	0.1151125	0.14805	0.162725	0.124925	18.54559783
Efpeglenatide_low_dosage	0.099275	0.0696	0.0601	0.056975	0.05155	0.044575	0.0403	0.0370625	0.033325	0.0308375	0.0283375	0.0274875	0.02705	0.02545	0.024875	0.0241625	0.02405	0.0250625	0.0273875	0.0317875	0.037	0.04195	0.0540125	0.0777875	55.24701087
Efpeglenatide_medium_dosage	0.000725	0.0025	0.0052625	0.0099375	0.015725	0.0221625	0.02865	0.033725	0.04	0.0441375	0.0478875	0.0516125	0.056475	0.06095	0.0620625	0.0644875	0.0628875	0.064575	0.067225	0.0721875	0.0733625	0.059125	0.0397125	0.014625	37.8298913
Empagliflozin_high_dosage	7.50E-05	5.00E-04	0.00105	0.002975	0.0070125	0.0122125	0.01985	0.0281375	0.036075	0.044475	0.0549	0.0625375	0.0712875	0.0761875	0.07725	0.0786	0.0744625	0.0742625	0.0681875	0.0668375	0.0596625	0.046025	0.027725	0.0097125	37.4211413
Empagliflozin_low_dosage	0.000225	0.001025	0.0031	0.0086125	0.0184	0.03345	0.0529625	0.0715125	0.0891	0.10355	0.1070875	0.1042875	0.097825	0.08705	0.0702625	0.0534625	0.03755	0.0254	0.015375	0.0105625	0.0055	0.002625	9.00E-04	0.000175	53.28168478
Ertugliflozin_high_dosage	0.00135	0.0032375	0.00605	0.0097375	0.0133125	0.01735	0.0205125	0.0235875	0.0273625	0.0305375	0.0351125	0.03715	0.0402625	0.0430125	0.045725	0.0497125	0.0508875	0.05415	0.0654875	0.073825	0.0864	0.0936875	0.094475	0.077075	30.35994565
Ertugliflozin_low_dosage	0.003425	0.007325	0.012875	0.0184875	0.0240125	0.0305	0.0341125	0.03935	0.0421	0.043025	0.0461	0.0478125	0.0493	0.04945	0.0523125	0.0513375	0.0525625	0.0533	0.057675	0.0637125	0.06535	0.0657625	0.0564375	0.033675	39.28255435
Exenatide	1.25E-05	3.75E-05	0.0001125	0.0002375	0.0004625	9.00E-04	0.0015875	0.0028375	0.0038	0.005325	0.0069	0.0098875	0.0134	0.0171875	0.0227	0.02715	0.0342125	0.0459625	0.0611	0.08675	0.1204625	0.162225	0.2013	0.17545	14.09206522
Inject_semaglutide_high_dosage	0.00625	0.01465	0.028325	0.04485	0.0589375	0.0665625	0.073225	0.071325	0.06925	0.0661	0.063075	0.058475	0.0542625	0.052775	0.0491	0.0412	0.037425	0.0328375	0.028925	0.0261	0.02325	0.0175625	0.011225	0.0043125	55.9475
Inject_semaglutide_low_dosage	0.0060125	0.01535	0.0248875	0.0376875	0.0466375	0.0509625	0.051375	0.05185	0.0513625	0.0493	0.047625	0.05015	0.0489625	0.0481125	0.0470875	0.0441125	0.0432875	0.0429125	0.0429625	0.044625	0.045725	0.04445	0.0381375	0.026425	47.62940217
Inject_semaglutide_medium_dosage	0.0673125	0.11875	0.1505	0.1411125	0.111825	0.0872375	0.0681875	0.0513	0.040375	0.033275	0.0267875	0.021725	0.0170375	0.0143875	0.0125125	0.0091125	0.0071	0.005975	0.004925	0.003925	0.00275	0.00195	0.001575	0.0003625	78.93559783
Liraglutide	3.75E-05	0.0003875	0.0013	0.0041	0.00775	0.0150375	0.02365	0.0349625	0.0462	0.057725	0.06725	0.0771875	0.0819375	0.087125	0.085675	0.0809625	0.0756875	0.0647125	0.0569875	0.04835	0.0391	0.026025	0.013675	0.004175	41.61086957
Lixisenatide	0.048975	0.0372875	0.0334875	0.03425	0.0325375	0.0289	0.02815	0.0262125	0.0262375	0.02255	0.0230125	0.0225125	0.0240625	0.0235875	0.0238125	0.0242375	0.0251125	0.026525	0.0305375	0.0349625	0.043725	0.059025	0.08535	0.23495	37.04673913
Oral_semaglutide	0.00195	0.0042875	0.0065875	0.010175	0.0135625	0.0164375	0.018275	0.0203375	0.022875	0.0238125	0.0257625	0.0287	0.0302375	0.032125	0.0346125	0.0365875	0.0395375	0.0420375	0.0503	0.06025	0.0757625	0.0977875	0.1337625	0.1742375	25.48978261

Placebo_or_Control	0	0	0	0	0	0	0	0	1.25E-05	3.00E-04	0.0010125	0.003775	0.012075	0.029975	0.0666125	0.116	0.1655375	0.190575	0.179125	0.1306625	0.0718875	0.026525	0.0054625	0.0004625	26.16347826
Sotagliflozin	0.002375	0.0077125	0.017775	0.034125	0.0512	0.0679125	0.0806	0.0880875	0.085825	0.0841625	0.078225	0.0724125	0.065425	0.0584125	0.048875	0.040725	0.0316875	0.0259125	0.019675	0.014575	0.01175	0.00765	0.003775	0.001125	57.67038043
Tirzepatide_high_dosage	0.13435	0.2785125	0.228175	0.1422875	0.0841375	0.051775	0.029025	0.0178625	0.0115375	0.00795	0.0052	0.0031875	0.0019625	0.001325	0.0009625	0.000725	0.0004125	0.0003125	2.00E-04	6.25E-05	1.25E-05	2.50E-05	0	0	89.60934783
Tirzepatide_low_dosage	0.4470375	0.2069125	0.1187	0.0707625	0.0457375	0.029725	0.02065	0.0142	0.0107375	0.00815	0.0059875	0.005	0.0039375	0.002975	0.002025	0.002175	0.0014	0.0013	0.000825	0.0006125	0.000525	0.000375	2.00E-04	5.00E-05	92.48951087

Table S7A: inconsistency within the network meta-analysis of primary outcome: overall acute kidney injury/acute renal failure

	Comparison	No.Studies	NMA	Direct	Indirect	Difference	Diff_95CI_lower	Diff_95CI_upper	*p* value
1	Albiglutide:Bexagliflozin	0	-0.444341842	NA	-0.444341842	NA	NA	NA	NA
2	Albiglutide:Canagliflozin_high_dosage	0	0.589767093	NA	0.589767093	NA	NA	NA	NA
3	Albiglutide:Canagliflozin_low_dosage	0	-0.03811025	NA	-0.03811025	NA	NA	NA	NA
4	Albiglutide:Dapagliflozin	0	0.231003525	NA	0.231003525	NA	NA	NA	NA
5	Albiglutide:Dulaglutide	0	-0.103991072	NA	-0.103991072	NA	NA	NA	NA
6	Albiglutide:Efpeglenatide_high_dosage	0	-0.375208035	NA	-0.375208035	NA	NA	NA	NA
7	Albiglutide:Efpeglenatide_low_dosage	0	0.721707245	NA	0.721707245	NA	NA	NA	NA
8	Albiglutide:Efpeglenatide_medium_dosage	0	-0.376905044	NA	-0.376905044	NA	NA	NA	NA
9	Albiglutide:Empagliflozin_high_dosage	0	0.369608812	NA	0.369608812	NA	NA	NA	NA
10	Albiglutide:Empagliflozin_low_dosage	0	0.189395548	NA	0.189395548	NA	NA	NA	NA
11	Albiglutide:Ertugliflozin_high_dosage	0	0.176701322	NA	0.176701322	NA	NA	NA	NA
12	Albiglutide:Ertugliflozin_low_dosage	0	-0.190252293	NA	-0.190252293	NA	NA	NA	NA
13	Albiglutide:Exenatide	0	0.563203594	NA	0.563203594	NA	NA	NA	NA
14	Albiglutide:Inject_semaglutide_high_dosage	0	0.152493797	NA	0.152493797	NA	NA	NA	NA
15	Albiglutide:Inject_semaglutide_low_dosage	0	-0.227331387	NA	-0.227331387	NA	NA	NA	NA
16	Albiglutide:Inject_semaglutide_medium_dosage	0	0.486791573	NA	0.486791573	NA	NA	NA	NA
17	Albiglutide:Liraglutide	1	-0.253778324	1.110927715	-0.283294137	1.394221852	-1.843798859	4.632242564	0.398715116
18	Albiglutide:Lixisenatide	0	0.999711505	NA	0.999711505	NA	NA	NA	NA
19	Albiglutide:Oral_semaglutide	0	0.071425379	NA	0.071425379	NA	NA	NA	NA

20	Albiglutide:Placebo_or_Control	2	-0.116044061	-0.136763693	1.257458159	-1.394221852	-4.632242564	1.843798859	0.398715116
21	Albiglutide:Sotagliflozin	0	-0.190830799	NA	-0.190830799	NA	NA	NA	NA
22	Albiglutide:Tirzepatide_high_dosage	0	-0.765089232	NA	-0.765089232	NA	NA	NA	NA
23	Albiglutide:Tirzepatide_low_dosage	0	0.068822155	NA	0.068822155	NA	NA	NA	NA
24	Bexagliflozin:Canagliflozin_high_dosage	0	1.034108935	NA	1.034108935	NA	NA	NA	NA
25	Bexagliflozin:Canagliflozin_low_dosage	0	0.406231593	NA	0.406231593	NA	NA	NA	NA
26	Bexagliflozin:Dapagliflozin	0	0.675345367	NA	0.675345367	NA	NA	NA	NA
27	Bexagliflozin:Dulaglutide	0	0.340350771	NA	0.340350771	NA	NA	NA	NA
28	Bexagliflozin:Efpeglenatide_high_dosage	0	0.069133808	NA	0.069133808	NA	NA	NA	NA
29	Bexagliflozin:Efpeglenatide_low_dosage	0	1.166049087	NA	1.166049087	NA	NA	NA	NA
30	Bexagliflozin:Efpeglenatide_medium_dosage	0	0.067436798	NA	0.067436798	NA	NA	NA	NA
31	Bexagliflozin:Empagliflozin_high_dosage	0	0.813950654	NA	0.813950654	NA	NA	NA	NA
32	Bexagliflozin:Empagliflozin_low_dosage	0	0.63373739	NA	0.63373739	NA	NA	NA	NA
33	Bexagliflozin:Ertugliflozin_high_dosage	0	0.621043164	NA	0.621043164	NA	NA	NA	NA
34	Bexagliflozin:Ertugliflozin_low_dosage	0	0.254089549	NA	0.254089549	NA	NA	NA	NA
35	Bexagliflozin:Exenatide	0	1.007545437	NA	1.007545437	NA	NA	NA	NA
36	Bexagliflozin:Inject_semaglutide_high_dosage	0	0.59683564	NA	0.59683564	NA	NA	NA	NA
37	Bexagliflozin:Inject_semaglutide_low_dosage	0	0.217010456	NA	0.217010456	NA	NA	NA	NA
38	Bexagliflozin:Inject_semaglutide_medium_dosage	0	0.931133415	NA	0.931133415	NA	NA	NA	NA
39	Bexagliflozin:Liraglutide	0	0.190563519	NA	0.190563519	NA	NA	NA	NA
40	Bexagliflozin:Lixisenatide	0	1.444053347	NA	1.444053347	NA	NA	NA	NA
41	Bexagliflozin:Oral_semaglutide	0	0.515767221	NA	0.515767221	NA	NA	NA	NA

42	Bexagliflozin:Placebo_or_Control	2	0.328297781	0.328297781	NA	NA	NA	NA	NA
43	Bexagliflozin:Sotagliflozin	0	0.253511044	NA	0.253511044	NA	NA	NA	NA
44	Bexagliflozin:Tirzepatide_high_dosage	0	-0.32074739	NA	-0.32074739	NA	NA	NA	NA
45	Bexagliflozin:Tirzepatide_low_dosage	0	0.513163998	NA	0.513163998	NA	NA	NA	NA
46	Canagliflozin_high_dosage:Canagliflozin_low_dosage	2	-0.627877343	-0.663210743	-0.589231223	-0.07397952	-1.277460595	1.129501555	0.904101741
47	Canagliflozin_high_dosage:Dapagliflozin	0	-0.358763568	NA	-0.358763568	NA	NA	NA	NA
48	Canagliflozin_high_dosage:Dulaglutide	0	-0.693758165	NA	-0.693758165	NA	NA	NA	NA
49	Canagliflozin_high_dosage:Efpeglenatide_high_dosage	0	-0.964975128	NA	-0.964975128	NA	NA	NA	NA
50	Canagliflozin_high_dosage:Efpeglenatide_low_dosage	0	0.131940152	NA	0.131940152	NA	NA	NA	NA
51	Canagliflozin_high_dosage:Efpeglenatide_medium_dosage	0	-0.966672137	NA	-0.966672137	NA	NA	NA	NA
52	Canagliflozin_high_dosage:Empagliflozin_high_dosage	0	-0.220158281	NA	-0.220158281	NA	NA	NA	NA
53	Canagliflozin_high_dosage:Empagliflozin_low_dosage	0	-0.400371545	NA	-0.400371545	NA	NA	NA	NA
54	Canagliflozin_high_dosage:Ertugliflozin_high_dosage	0	-0.413065771	NA	-0.413065771	NA	NA	NA	NA
55	Canagliflozin_high_dosage:Ertugliflozin_low_dosage	0	-0.780019386	NA	-0.780019386	NA	NA	NA	NA
56	Canagliflozin_high_dosage:Exenatide	0	-0.026563499	NA	-0.026563499	NA	NA	NA	NA
57	Canagliflozin_high_dosage:Inject_semaglutide_high_dosage	0	-0.437273296	NA	-0.437273296	NA	NA	NA	NA
58	Canagliflozin_high_dosage:Inject_semaglutide_low_dosage	0	-0.81709848	NA	-0.81709848	NA	NA	NA	NA
59	Canagliflozin_high_dosage:Inject_semaglutide_medium_dosage	0	-0.10297552	NA	-0.10297552	NA	NA	NA	NA
60	Canagliflozin_high_dosage:Liraglutide	0	-0.843545417	NA	-0.843545417	NA	NA	NA	NA
61	Canagliflozin_high_dosage:Lixisenatide	0	0.409944412	NA	0.409944412	NA	NA	NA	NA
62	Canagliflozin_high_dosage:Oral_semaglutide	0	-0.518341714	NA	-0.518341714	NA	NA	NA	NA
63	Canagliflozin_high_dosage:Placebo_or_Control	3	-0.705811154	-0.642671132	-1.160268614	0.517597483	-1.252583404	2.287778369	0.566583964

64	Canagliflozin_high_dosage:Sotagliflozin	0	-0.780597892	NA	-0.780597892	NA	NA	NA	NA
65	Canagliflozin_high_dosage:Tirzepatide_high_dosage	0	-1.354856325	NA	-1.354856325	NA	NA	NA	NA
66	Canagliflozin_high_dosage:Tirzepatide_low_dosage	0	-0.520944938	NA	-0.520944938	NA	NA	NA	NA
67	Canagliflozin_low_dosage:Dapagliflozin	0	0.269113774	NA	0.269113774	NA	NA	NA	NA
68	Canagliflozin_low_dosage:Dulaglutide	0	-0.065880822	NA	-0.065880822	NA	NA	NA	NA
69	Canagliflozin_low_dosage:Efpeglenatide_high_dosage	0	-0.337097785	NA	-0.337097785	NA	NA	NA	NA
70	Canagliflozin_low_dosage:Efpeglenatide_low_dosage	0	0.759817495	NA	0.759817495	NA	NA	NA	NA
71	Canagliflozin_low_dosage:Efpeglenatide_medium_dosage	0	-0.338794794	NA	-0.338794794	NA	NA	NA	NA
72	Canagliflozin_low_dosage:Empagliflozin_high_dosage	0	0.407719061	NA	0.407719061	NA	NA	NA	NA
73	Canagliflozin_low_dosage:Empagliflozin_low_dosage	0	0.227505798	NA	0.227505798	NA	NA	NA	NA
74	Canagliflozin_low_dosage:Ertugliflozin_high_dosage	0	0.214811572	NA	0.214811572	NA	NA	NA	NA
75	Canagliflozin_low_dosage:Ertugliflozin_low_dosage	0	-0.152142044	NA	-0.152142044	NA	NA	NA	NA
76	Canagliflozin_low_dosage:Exenatide	0	0.601313844	NA	0.601313844	NA	NA	NA	NA
77	Canagliflozin_low_dosage:Inject_semaglutide_high_dosage	0	0.190604047	NA	0.190604047	NA	NA	NA	NA
78	Canagliflozin_low_dosage:Inject_semaglutide_low_dosage	0	-0.189221137	NA	-0.189221137	NA	NA	NA	NA
79	Canagliflozin_low_dosage:Inject_semaglutide_medium_dosage	0	0.524901823	NA	0.524901823	NA	NA	NA	NA
80	Canagliflozin_low_dosage:Liraglutide	0	-0.215668074	NA	-0.215668074	NA	NA	NA	NA
81	Canagliflozin_low_dosage:Lixisenatide	0	1.037821754	NA	1.037821754	NA	NA	NA	NA
82	Canagliflozin_low_dosage:Oral_semaglutide	0	0.109535628	NA	0.109535628	NA	NA	NA	NA
83	Canagliflozin_low_dosage:Placebo_or_Control	6	-0.077933811	-0.075223935	-0.227033942	0.151810007	-1.84123578	2.144855795	0.881324671
84	Canagliflozin_low_dosage:Sotagliflozin	0	-0.152720549	NA	-0.152720549	NA	NA	NA	NA
85	Canagliflozin_low_dosage:Tirzepatide_high_dosage	0	-0.726978982	NA	-0.726978982	NA	NA	NA	NA

86	Canagliflozin_low_dosage:Tirzepatide_low_dosage	0	0.106932405	NA	0.106932405	NA	NA	NA	NA
87	Dapagliflozin:Dulaglutide	0	-0.334994596	NA	-0.334994596	NA	NA	NA	NA
88	Dapagliflozin:Efpeglenatide_high_dosage	0	-0.606211559	NA	-0.606211559	NA	NA	NA	NA
89	Dapagliflozin:Efpeglenatide_low_dosage	0	0.49070372	NA	0.49070372	NA	NA	NA	NA
90	Dapagliflozin:Efpeglenatide_medium_dosage	0	-0.607908568	NA	-0.607908568	NA	NA	NA	NA
91	Dapagliflozin:Empagliflozin_high_dosage	0	0.138605287	NA	0.138605287	NA	NA	NA	NA
92	Dapagliflozin:Empagliflozin_low_dosage	0	-0.041607977	NA	-0.041607977	NA	NA	NA	NA
93	Dapagliflozin:Ertugliflozin_high_dosage	0	-0.054302203	NA	-0.054302203	NA	NA	NA	NA
94	Dapagliflozin:Ertugliflozin_low_dosage	0	-0.421255818	NA	-0.421255818	NA	NA	NA	NA
95	Dapagliflozin:Exenatide	0	0.33220007	NA	0.33220007	NA	NA	NA	NA
96	Dapagliflozin:Inject_semaglutide_high_dosage	0	-0.078509727	NA	-0.078509727	NA	NA	NA	NA
97	Dapagliflozin:Inject_semaglutide_low_dosage	0	-0.458334911	NA	-0.458334911	NA	NA	NA	NA
98	Dapagliflozin:Inject_semaglutide_medium_dosage	0	0.255788048	NA	0.255788048	NA	NA	NA	NA
99	Dapagliflozin:Liraglutide	0	-0.484781848	NA	-0.484781848	NA	NA	NA	NA
100	Dapagliflozin:Lixisenatide	0	0.76870798	NA	0.76870798	NA	NA	NA	NA
101	Dapagliflozin:Oral_semaglutide	0	-0.159578146	NA	-0.159578146	NA	NA	NA	NA
102	Dapagliflozin:Placebo_or_Control	9	-0.347047585	-0.347047585	NA	NA	NA	NA	NA
103	Dapagliflozin:Sotagliflozin	0	-0.421834323	NA	-0.421834323	NA	NA	NA	NA
104	Dapagliflozin:Tirzepatide_high_dosage	0	-0.996092756	NA	-0.996092756	NA	NA	NA	NA
105	Dapagliflozin:Tirzepatide_low_dosage	0	-0.162181369	NA	-0.162181369	NA	NA	NA	NA
106	Dulaglutide:Efpeglenatide_high_dosage	1	-0.271216963	-1.13565356	-0.199655169	-0.935998391	-4.27065627	2.398659487	0.582224494
107	Dulaglutide:Efpeglenatide_low_dosage	0	0.825698317	NA	0.825698317	NA	NA	NA	NA

108	Dulaglutide:Efpeglenatide_medium_dosage	1	-0.272913972	-1.066089097	-0.207829349	-0.858259747	-4.191651902	2.475132407	0.613812726
109	Dulaglutide:Empagliflozin_high_dosage	0	0.473599883	NA	0.473599883	NA	NA	NA	NA
110	Dulaglutide:Empagliflozin_low_dosage	0	0.29338662	NA	0.29338662	NA	NA	NA	NA
111	Dulaglutide:Ertugliflozin_high_dosage	0	0.280692394	NA	0.280692394	NA	NA	NA	NA
112	Dulaglutide:Ertugliflozin_low_dosage	0	-0.086261222	NA	-0.086261222	NA	NA	NA	NA
113	Dulaglutide:Exenatide	0	0.667194666	NA	0.667194666	NA	NA	NA	NA
114	Dulaglutide:Inject_semaglutide_high_dosage	0	0.256484869	NA	0.256484869	NA	NA	NA	NA
115	Dulaglutide:Inject_semaglutide_low_dosage	0	-0.123340315	NA	-0.123340315	NA	NA	NA	NA
116	Dulaglutide:Inject_semaglutide_medium_dosage	0	0.590782645	NA	0.590782645	NA	NA	NA	NA
117	Dulaglutide:Liraglutide	1	-0.149787252	-1.098612289	-0.134399468	-0.964212821	-4.194706791	2.26628115	0.558551166
118	Dulaglutide:Lixisenatide	0	1.103702577	NA	1.103702577	NA	NA	NA	NA
119	Dulaglutide:Oral_semaglutide	0	0.175416451	NA	0.175416451	NA	NA	NA	NA
120	Dulaglutide:Placebo_or_Control	2	-0.012052989	0.004830449	-0.588807898	0.593638346	-1.303338875	2.490615568	0.539645358
121	Dulaglutide:Sotagliflozin	0	-0.086839727	NA	-0.086839727	NA	NA	NA	NA
122	Dulaglutide:Tirzepatide_high_dosage	1	-0.66109816	-0.457241378	-0.691736625	0.234495247	-3.216194384	3.685184879	0.894041999
123	Dulaglutide:Tirzepatide_low_dosage	0	0.172813227	NA	0.172813227	NA	NA	NA	NA
124	Efpeglenatide_high_dosage:Efpeglenatide_low_dosage	0	1.096915279	NA	1.096915279	NA	NA	NA	NA
125	Efpeglenatide_high_dosage:Efpeglenatide_medium_dosage	3	-0.001697009	0.025488071	-1.783805733	1.809293804	-4.552916749	8.171504357	0.577269784
126	Efpeglenatide_high_dosage:Empagliflozin_high_dosage	0	0.744816846	NA	0.744816846	NA	NA	NA	NA
127	Efpeglenatide_high_dosage:Empagliflozin_low_dosage	0	0.564603583	NA	0.564603583	NA	NA	NA	NA
128	Efpeglenatide_high_dosage:Ertugliflozin_high_dosage	0	0.551909357	NA	0.551909357	NA	NA	NA	NA
129	Efpeglenatide_high_dosage:Ertugliflozin_low_dosage	0	0.184955741	NA	0.184955741	NA	NA	NA	NA

130	Efpeglenatide_high_dosage:Exenatide	0	0.938411629	NA	0.938411629	NA	NA	NA	NA
131	Efpeglenatide_high_dosage:Inject_semaglutide_high_dosage	0	0.527701832	NA	0.527701832	NA	NA	NA	NA
132	Efpeglenatide_high_dosage:Inject_semaglutide_low_dosage	0	0.147876648	NA	0.147876648	NA	NA	NA	NA
133	Efpeglenatide_high_dosage:Inject_semaglutide_medium_dosage	0	0.861999608	NA	0.861999608	NA	NA	NA	NA
134	Efpeglenatide_high_dosage:Liraglutide	0	0.121429711	NA	0.121429711	NA	NA	NA	NA
135	Efpeglenatide_high_dosage:Lixisenatide	0	1.374919539	NA	1.374919539	NA	NA	NA	NA
136	Efpeglenatide_high_dosage:Oral_semaglutide	0	0.446633413	NA	0.446633413	NA	NA	NA	NA
137	Efpeglenatide_high_dosage:Placebo_or_Control	1	0.259163974	0.199928029	0.751695653	-0.551767624	-3.250262833	2.146727585	0.688597996
138	Efpeglenatide_high_dosage:Sotagliflozin	0	0.184377236	NA	0.184377236	NA	NA	NA	NA
139	Efpeglenatide_high_dosage:Tirzepatide_high_dosage	0	-0.389881197	NA	-0.389881197	NA	NA	NA	NA
140	Efpeglenatide_high_dosage:Tirzepatide_low_dosage	0	0.44403019	NA	0.44403019	NA	NA	NA	NA
141	Efpeglenatide_low_dosage:Efpeglenatide_medium_dosage	1	-1.098612289	-1.098612289	NA	NA	NA	NA	NA
142	Efpeglenatide_low_dosage:Empagliflozin_high_dosage	0	-0.352098433	NA	-0.352098433	NA	NA	NA	NA
143	Efpeglenatide_low_dosage:Empagliflozin_low_dosage	0	-0.532311697	NA	-0.532311697	NA	NA	NA	NA
144	Efpeglenatide_low_dosage:Ertugliflozin_high_dosage	0	-0.545005923	NA	-0.545005923	NA	NA	NA	NA
145	Efpeglenatide_low_dosage:Ertugliflozin_low_dosage	0	-0.911959538	NA	-0.911959538	NA	NA	NA	NA
146	Efpeglenatide_low_dosage:Exenatide	0	-0.15850365	NA	-0.15850365	NA	NA	NA	NA
147	Efpeglenatide_low_dosage:Inject_semaglutide_high_dosage	0	-0.569213448	NA	-0.569213448	NA	NA	NA	NA
148	Efpeglenatide_low_dosage:Inject_semaglutide_low_dosage	0	-0.949038631	NA	-0.949038631	NA	NA	NA	NA
149	Efpeglenatide_low_dosage:Inject_semaglutide_medium_dosage	0	-0.234915672	NA	-0.234915672	NA	NA	NA	NA
150	Efpeglenatide_low_dosage:Liraglutide	0	-0.975485569	NA	-0.975485569	NA	NA	NA	NA
151	Efpeglenatide_low_dosage:Lixisenatide	0	0.27800426	NA	0.27800426	NA	NA	NA	NA

152	Efpeglenatide_low_dosage:Oral_semaglutide	0	-0.650281866	NA	-0.650281866	NA	NA	NA	NA
153	Efpeglenatide_low_dosage:Placebo_or_Control	0	-0.837751306	NA	-0.837751306	NA	NA	NA	NA
154	Efpeglenatide_low_dosage:Sotagliflozin	0	-0.912538043	NA	-0.912538043	NA	NA	NA	NA
155	Efpeglenatide_low_dosage:Tirzepatide_high_dosage	0	-1.486796477	NA	-1.486796477	NA	NA	NA	NA
156	Efpeglenatide_low_dosage:Tirzepatide_low_dosage	0	-0.652885089	NA	-0.652885089	NA	NA	NA	NA
157	Efpeglenatide_medium_dosage:Empagliflozin_high_dosage	0	0.746513856	NA	0.746513856	NA	NA	NA	NA
158	Efpeglenatide_medium_dosage:Empagliflozin_low_dosage	0	0.566300592	NA	0.566300592	NA	NA	NA	NA
159	Efpeglenatide_medium_dosage:Ertugliflozin_high_dosage	0	0.553606366	NA	0.553606366	NA	NA	NA	NA
160	Efpeglenatide_medium_dosage:Ertugliflozin_low_dosage	0	0.18665275	NA	0.18665275	NA	NA	NA	NA
161	Efpeglenatide_medium_dosage:Exenatide	0	0.940108638	NA	0.940108638	NA	NA	NA	NA
162	Efpeglenatide_medium_dosage:Inject_semaglutide_high_dosage	0	0.529398841	NA	0.529398841	NA	NA	NA	NA
163	Efpeglenatide_medium_dosage:Inject_semaglutide_low_dosage	0	0.149573657	NA	0.149573657	NA	NA	NA	NA
164	Efpeglenatide_medium_dosage:Inject_semaglutide_medium_dosage	0	0.863696617	NA	0.863696617	NA	NA	NA	NA
165	Efpeglenatide_medium_dosage:Liraglutide	0	0.12312672	NA	0.12312672	NA	NA	NA	NA
166	Efpeglenatide_medium_dosage:Lixisenatide	0	1.376616549	NA	1.376616549	NA	NA	NA	NA
167	Efpeglenatide_medium_dosage:Oral_semaglutide	0	0.448330423	NA	0.448330423	NA	NA	NA	NA
168	Efpeglenatide_medium_dosage:Placebo_or_Control	2	0.260860983	0.175455431	1.162638845	-0.987183414	-3.944791239	1.970424411	0.512987993
169	Efpeglenatide_medium_dosage:Sotagliflozin	0	0.186074245	NA	0.186074245	NA	NA	NA	NA
170	Efpeglenatide_medium_dosage:Tirzepatide_high_dosage	0	-0.388184188	NA	-0.388184188	NA	NA	NA	NA
171	Efpeglenatide_medium_dosage:Tirzepatide_low_dosage	0	0.445727199	NA	0.445727199	NA	NA	NA	NA
172	Empagliflozin_high_dosage:Empagliflozin_low_dosage	3	-0.180213264	-0.251335304	-0.006796508	-0.244538795	-1.291361123	0.802283532	0.647060465
173	Empagliflozin_high_dosage:Ertugliflozin_high_dosage	0	-0.19290749	NA	-0.19290749	NA	NA	NA	NA

174	Empagliflozin_high_dosage:Ertugliflozin_low_dosage	0	-0.559861105	NA	-0.559861105	NA	NA	NA	NA
175	Empagliflozin_high_dosage:Exenatide	0	0.193594783	NA	0.193594783	NA	NA	NA	NA
176	Empagliflozin_high_dosage:Inject_semaglutide_high_dosage	0	-0.217115014	NA	-0.217115014	NA	NA	NA	NA
177	Empagliflozin_high_dosage:Inject_semaglutide_low_dosage	0	-0.596940198	NA	-0.596940198	NA	NA	NA	NA
178	Empagliflozin_high_dosage:Inject_semaglutide_medium_dosage	0	0.117182761	NA	0.117182761	NA	NA	NA	NA
179	Empagliflozin_high_dosage:Liraglutide	0	-0.623387135	NA	-0.623387135	NA	NA	NA	NA
180	Empagliflozin_high_dosage:Lixisenatide	0	0.630102693	NA	0.630102693	NA	NA	NA	NA
181	Empagliflozin_high_dosage:Oral_semaglutide	1	-0.298183433	0.002447982	-0.324194788	0.32664277	-2.56612358	3.21940912	0.824848388
182	Empagliflozin_high_dosage:Placebo_or_Control	4	-0.485652873	-0.453233141	-0.636720148	0.183487007	-1.048681784	1.415655797	0.770389236
183	Empagliflozin_high_dosage:Sotagliflozin	0	-0.56043961	NA	-0.56043961	NA	NA	NA	NA
184	Empagliflozin_high_dosage:Tirzepatide_high_dosage	0	-1.134698044	NA	-1.134698044	NA	NA	NA	NA
185	Empagliflozin_high_dosage:Tirzepatide_low_dosage	0	-0.300786656	NA	-0.300786656	NA	NA	NA	NA
186	Empagliflozin_low_dosage:Ertugliflozin_high_dosage	0	-0.012694226	NA	-0.012694226	NA	NA	NA	NA
187	Empagliflozin_low_dosage:Ertugliflozin_low_dosage	0	-0.379647841	NA	-0.379647841	NA	NA	NA	NA
188	Empagliflozin_low_dosage:Exenatide	0	0.373808046	NA	0.373808046	NA	NA	NA	NA
189	Empagliflozin_low_dosage:Inject_semaglutide_high_dosage	0	-0.036901751	NA	-0.036901751	NA	NA	NA	NA
190	Empagliflozin_low_dosage:Inject_semaglutide_low_dosage	0	-0.416726935	NA	-0.416726935	NA	NA	NA	NA
191	Empagliflozin_low_dosage:Inject_semaglutide_medium_dosage	0	0.297396025	NA	0.297396025	NA	NA	NA	NA
192	Empagliflozin_low_dosage:Liraglutide	0	-0.443173872	NA	-0.443173872	NA	NA	NA	NA
193	Empagliflozin_low_dosage:Lixisenatide	0	0.810315957	NA	0.810315957	NA	NA	NA	NA
194	Empagliflozin_low_dosage:Oral_semaglutide	0	-0.117970169	NA	-0.117970169	NA	NA	NA	NA
195	Empagliflozin_low_dosage:Placebo_or_Control	8	-0.305439609	-0.305610642	-0.249067448	-0.056543194	-3.094233067	2.98114668	0.970897605

196	Empagliflozin_low_dosage:Sotagliflozin	0	-0.380226347	NA	-0.380226347	NA	NA	NA	NA
197	Empagliflozin_low_dosage:Tirzepatide_high_dosage	0	-0.95448478	NA	-0.95448478	NA	NA	NA	NA
198	Empagliflozin_low_dosage:Tirzepatide_low_dosage	0	-0.120573393	NA	-0.120573393	NA	NA	NA	NA
199	Ertugliflozin_high_dosage:Ertugliflozin_low_dosage	2	-0.366953615	-0.34146741	-4.011763556	3.670296145	-3.546096112	10.8866884	0.318838156
200	Ertugliflozin_high_dosage:Exenatide	0	0.386502272	NA	0.386502272	NA	NA	NA	NA
201	Ertugliflozin_high_dosage:Inject_semaglutide_high_dosage	0	-0.024207525	NA	-0.024207525	NA	NA	NA	NA
202	Ertugliflozin_high_dosage:Inject_semaglutide_low_dosage	0	-0.404032709	NA	-0.404032709	NA	NA	NA	NA
203	Ertugliflozin_high_dosage:Inject_semaglutide_medium_dosage	0	0.310090251	NA	0.310090251	NA	NA	NA	NA
204	Ertugliflozin_high_dosage:Liraglutide	0	-0.430479646	NA	-0.430479646	NA	NA	NA	NA
205	Ertugliflozin_high_dosage:Lixisenatide	0	0.823010183	NA	0.823010183	NA	NA	NA	NA
206	Ertugliflozin_high_dosage:Oral_semaglutide	0	-0.105275943	NA	-0.105275943	NA	NA	NA	NA
207	Ertugliflozin_high_dosage:Placebo_or_Control	1	-0.292745383	-0.290617854	-0.395595018	0.104977164	-4.203995342	4.413949669	0.961915851
208	Ertugliflozin_high_dosage:Sotagliflozin	0	-0.367532121	NA	-0.367532121	NA	NA	NA	NA
209	Ertugliflozin_high_dosage:Tirzepatide_high_dosage	0	-0.941790554	NA	-0.941790554	NA	NA	NA	NA
210	Ertugliflozin_high_dosage:Tirzepatide_low_dosage	0	-0.107879167	NA	-0.107879167	NA	NA	NA	NA
211	Ertugliflozin_low_dosage:Exenatide	0	0.753455888	NA	0.753455888	NA	NA	NA	NA
212	Ertugliflozin_low_dosage:Inject_semaglutide_high_dosage	0	0.342746091	NA	0.342746091	NA	NA	NA	NA
213	Ertugliflozin_low_dosage:Inject_semaglutide_low_dosage	0	-0.037079093	NA	-0.037079093	NA	NA	NA	NA
214	Ertugliflozin_low_dosage:Inject_semaglutide_medium_dosage	0	0.677043866	NA	0.677043866	NA	NA	NA	NA
215	Ertugliflozin_low_dosage:Liraglutide	0	-0.06352603	NA	-0.06352603	NA	NA	NA	NA
216	Ertugliflozin_low_dosage:Lixisenatide	0	1.189963798	NA	1.189963798	NA	NA	NA	NA
217	Ertugliflozin_low_dosage:Oral_semaglutide	0	0.261677672	NA	0.261677672	NA	NA	NA	NA

218	Ertugliflozin_low_dosage:Placebo_or_Control	2	0.074208233	0.053399449	2.625191221	-2.571791771	-8.782221039	3.638637496	0.416999486
219	Ertugliflozin_low_dosage:Sotagliflozin	0	-0.000578505	NA	-0.000578505	NA	NA	NA	NA
220	Ertugliflozin_low_dosage:Tirzepatide_high_dosage	0	-0.574836938	NA	-0.574836938	NA	NA	NA	NA
221	Ertugliflozin_low_dosage:Tirzepatide_low_dosage	0	0.259074449	NA	0.259074449	NA	NA	NA	NA
222	Exenatide:Inject_semaglutide_high_dosage	0	-0.410709797	NA	-0.410709797	NA	NA	NA	NA
223	Exenatide:Inject_semaglutide_low_dosage	0	-0.790534981	NA	-0.790534981	NA	NA	NA	NA
224	Exenatide:Inject_semaglutide_medium_dosage	0	-0.076412021	NA	-0.076412021	NA	NA	NA	NA
225	Exenatide:Liraglutide	0	-0.816981918	NA	-0.816981918	NA	NA	NA	NA
226	Exenatide:Lixisenatide	0	0.43650791	NA	0.43650791	NA	NA	NA	NA
227	Exenatide:Oral_semaglutide	0	-0.491778216	NA	-0.491778216	NA	NA	NA	NA
228	Exenatide:Placebo_or_Control	3	-0.679247655	-0.679247655	NA	NA	NA	NA	NA
229	Exenatide:Sotagliflozin	0	-0.754034393	NA	-0.754034393	NA	NA	NA	NA
230	Exenatide:Tirzepatide_high_dosage	0	-1.328292826	NA	-1.328292826	NA	NA	NA	NA
231	Exenatide:Tirzepatide_low_dosage	0	-0.494381439	NA	-0.494381439	NA	NA	NA	NA
232	Inject_semaglutide_high_dosage:Inject_semaglutide_low_dosage	0	-0.379825184	NA	-0.379825184	NA	NA	NA	NA
233	Inject_semaglutide_high_dosage:Inject_semaglutide_medium_dosage	1	0.334297776	0.405465108	0.321418418	0.08404669	-1.866141047	2.034234426	0.932684333
234	Inject_semaglutide_high_dosage:Liraglutide	1	-0.406272121	1.114422895	-0.427086597	1.541509492	-1.690467127	4.773486111	0.349883191
235	Inject_semaglutide_high_dosage:Lixisenatide	0	0.847217708	NA	0.847217708	NA	NA	NA	NA
236	Inject_semaglutide_high_dosage:Oral_semaglutide	0	-0.081068418	NA	-0.081068418	NA	NA	NA	NA
237	Inject_semaglutide_high_dosage:Placebo_or_Control	4	-0.268537858	-0.274385957	0.026846936	-0.301232893	-2.277845777	1.675379991	0.765172295
238	Inject_semaglutide_high_dosage:Sotagliflozin	0	-0.343324596	NA	-0.343324596	NA	NA	NA	NA
239	Inject_semaglutide_high_dosage:Tirzepatide_high_dosage	0	-0.917583029	NA	-0.917583029	NA	NA	NA	NA

240	Inject_semaglutide_high_dosage:Tirzepatide_low_dosage	0	-0.083671642	NA	-0.083671642	NA	NA	NA	NA
241	Inject_semaglutide_low_dosage:Inject_semaglutide_medium_dosage	2	0.71412296	0.894447144	-0.352050854	1.246497998	-0.830240346	3.323236341	0.239431957
242	Inject_semaglutide_low_dosage:Liraglutide	1	-0.026446937	-0.837589046	-0.000119247	-0.837469799	-4.0963392	2.421399602	0.614489866
243	Inject_semaglutide_low_dosage:Lixisenatide	0	1.227042891	NA	1.227042891	NA	NA	NA	NA
244	Inject_semaglutide_low_dosage:Oral_semaglutide	0	0.298756765	NA	0.298756765	NA	NA	NA	NA
245	Inject_semaglutide_low_dosage:Placebo_or_Control	2	0.111287326	0.129468118	-0.220789345	0.350257462	-1.952675529	2.653190453	0.765630922
246	Inject_semaglutide_low_dosage:Sotagliflozin	0	0.036500588	NA	0.036500588	NA	NA	NA	NA
247	Inject_semaglutide_low_dosage:Tirzepatide_high_dosage	0	-0.537757845	NA	-0.537757845	NA	NA	NA	NA
248	Inject_semaglutide_low_dosage:Tirzepatide_low_dosage	0	0.296153542	NA	0.296153542	NA	NA	NA	NA
249	Inject_semaglutide_medium_dosage:Liraglutide	0	-0.740569897	NA	-0.740569897	NA	NA	NA	NA
250	Inject_semaglutide_medium_dosage:Lixisenatide	0	0.512919932	NA	0.512919932	NA	NA	NA	NA
251	Inject_semaglutide_medium_dosage:Oral_semaglutide	0	-0.415366194	NA	-0.415366194	NA	NA	NA	NA
252	Inject_semaglutide_medium_dosage:Placebo_or_Control	3	-0.602835634	-0.639786405	-0.37840586	-0.261380544	-2.156316074	1.633554985	0.786890745
253	Inject_semaglutide_medium_dosage:Sotagliflozin	0	-0.677622372	NA	-0.677622372	NA	NA	NA	NA
254	Inject_semaglutide_medium_dosage:Tirzepatide_high_dosage	1	-1.251880805	-0.405997731	-1.408861007	1.002863275	-2.484186965	4.489913516	0.572972825
255	Inject_semaglutide_medium_dosage:Tirzepatide_low_dosage	0	-0.417969418	NA	-0.417969418	NA	NA	NA	NA
256	Liraglutide:Lixisenatide	0	1.253489828	NA	1.253489828	NA	NA	NA	NA
257	Liraglutide:Oral_semaglutide	0	0.325203702	NA	0.325203702	NA	NA	NA	NA
258	Liraglutide:Placebo_or_Control	3	0.137734263	0.145104586	-0.147294081	0.292398667	-1.343191628	1.927988962	0.726047694
259	Liraglutide:Sotagliflozin	0	0.062947525	NA	0.062947525	NA	NA	NA	NA
260	Liraglutide:Tirzepatide_high_dosage	0	-0.511310908	NA	-0.511310908	NA	NA	NA	NA
261	Liraglutide:Tirzepatide_low_dosage	0	0.322600479	NA	0.322600479	NA	NA	NA	NA

262	Lixisenatide:Oral_semaglutide	0	-0.928286126	NA	-0.928286126	NA	NA	NA	NA
263	Lixisenatide:Placebo_or_Control	1	-1.115755566	-1.115755566	NA	NA	NA	NA	NA
264	Lixisenatide:Sotagliflozin	0	-1.190542303	NA	-1.190542303	NA	NA	NA	NA
265	Lixisenatide:Tirzepatide_high_dosage	0	-1.764800737	NA	-1.764800737	NA	NA	NA	NA
266	Lixisenatide:Tirzepatide_low_dosage	0	-0.930889349	NA	-0.930889349	NA	NA	NA	NA
267	Oral_semaglutide:Placebo_or_Control	3	-0.18746944	-0.170319447	-0.496962216	0.32664277	-2.56612358	3.21940912	0.824848388
268	Oral_semaglutide:Sotagliflozin	0	-0.262256177	NA	-0.262256177	NA	NA	NA	NA
269	Oral_semaglutide:Tirzepatide_high_dosage	0	-0.836514611	NA	-0.836514611	NA	NA	NA	NA
270	Oral_semaglutide:Tirzepatide_low_dosage	0	-0.002603223	NA	-0.002603223	NA	NA	NA	NA
271	Sotagliflozin:Placebo_or_Control	4	0.074786738	0.074786738	NA	NA	NA	NA	NA
272	Tirzepatide_high_dosage:Placebo_or_Control	5	0.649045171	0.821141732	0.108633877	0.712507855	-1.928290097	3.353305807	0.596934799
273	Tirzepatide_low_dosage:Placebo_or_Control	0	-0.184866216	NA	-0.184866216	NA	NA	NA	NA
274	Sotagliflozin:Tirzepatide_high_dosage	0	-0.574258433	NA	-0.574258433	NA	NA	NA	NA
275	Sotagliflozin:Tirzepatide_low_dosage	0	0.259652954	NA	0.259652954	NA	NA	NA	NA
276	Tirzepatide_high_dosage:Tirzepatide_low_dosage	3	0.833911387	0.833911387	NA	NA	NA	NA	NA

Table S7B: inconsistency within the network meta-analysis of primary outcome: acute kidney injury/acute renal failure in the subgroup focusing RCTs without definite underlying kidney failure

	Comparison	No.Studies	NMA	Direct	Indirect	Difference	Diff_95CI_lower	Diff_95CI_upper	*p* value
1	Albiglutide:Bexagliflozin	0	-0.342749324	NA	-0.342749324	NA	NA	NA	NA
2	Albiglutide:Canagliflozin_high_dosage	0	0.556993672	NA	0.556993672	NA	NA	NA	NA
3	Albiglutide:Canagliflozin_low_dosage	0	-0.095269752	NA	-0.095269752	NA	NA	NA	NA
4	Albiglutide:Dapagliflozin	0	0.281598725	NA	0.281598725	NA	NA	NA	NA
5	Albiglutide:Dulaglutide	0	-0.072796897	NA	-0.072796897	NA	NA	NA	NA
6	Albiglutide:Efpeglenatide_high_dosage	0	-0.373314545	NA	-0.373314545	NA	NA	NA	NA
7	Albiglutide:Efpeglenatide_low_dosage	0	0.723589592	NA	0.723589592	NA	NA	NA	NA
8	Albiglutide:Efpeglenatide_medium_dosage	0	-0.375022697	NA	-0.375022697	NA	NA	NA	NA
9	Albiglutide:Empagliflozin_high_dosage	0	0.444907415	NA	0.444907415	NA	NA	NA	NA
10	Albiglutide:Empagliflozin_low_dosage	0	0.228460534	NA	0.228460534	NA	NA	NA	NA
11	Albiglutide:Ertugliflozin_high_dosage	0	0.174570804	NA	0.174570804	NA	NA	NA	NA
12	Albiglutide:Ertugliflozin_low_dosage	0	-0.115679606	NA	-0.115679606	NA	NA	NA	NA
13	Albiglutide:Exenatide	0	0.563200605	NA	0.563200605	NA	NA	NA	NA
14	Albiglutide:Inject_semaglutide_high_dosage	0	0.152495401	NA	0.152495401	NA	NA	NA	NA
15	Albiglutide:Inject_semaglutide_low_dosage	0	-0.227298843	NA	-0.227298843	NA	NA	NA	NA
16	Albiglutide:Inject_semaglutide_medium_dosage	0	0.486952909	NA	0.486952909	NA	NA	NA	NA
17	Albiglutide:Liraglutide	1	-0.25358143	1.110927715	-0.283093008	1.394020724	-1.844000015	4.632041463	0.398783158
18	Albiglutide:Lixisenatide	0	0.999708515	NA	0.999708515	NA	NA	NA	NA

19	Albiglutide:Oral_semaglutide	0	-0.343111651	NA	-0.343111651	NA	NA	NA	NA
20	Albiglutide:Placebo_or_Control	2	-0.11604705	-0.136763693	1.25725703	-1.394020724	-4.632041463	1.844000015	0.398783158
21	Albiglutide:Sotagliflozin	0	0.212519783	NA	0.212519783	NA	NA	NA	NA
22	Albiglutide:Tirzepatide_high_dosage	0	-0.761246213	NA	-0.761246213	NA	NA	NA	NA
23	Albiglutide:Tirzepatide_low_dosage	0	0.072665174	NA	0.072665174	NA	NA	NA	NA
24	Bexagliflozin:Canagliflozin_high_dosage	0	0.899742997	NA	0.899742997	NA	NA	NA	NA
25	Bexagliflozin:Canagliflozin_low_dosage	0	0.247479573	NA	0.247479573	NA	NA	NA	NA
26	Bexagliflozin:Dapagliflozin	0	0.624348049	NA	0.624348049	NA	NA	NA	NA
27	Bexagliflozin:Dulaglutide	0	0.269952428	NA	0.269952428	NA	NA	NA	NA
28	Bexagliflozin:Efpeglenatide_high_dosage	0	-0.030565221	NA	-0.030565221	NA	NA	NA	NA
29	Bexagliflozin:Efpeglenatide_low_dosage	0	1.066338916	NA	1.066338916	NA	NA	NA	NA
30	Bexagliflozin:Efpeglenatide_medium_dosage	0	-0.032273372	NA	-0.032273372	NA	NA	NA	NA
31	Bexagliflozin:Empagliflozin_high_dosage	0	0.787656739	NA	0.787656739	NA	NA	NA	NA
32	Bexagliflozin:Empagliflozin_low_dosage	0	0.571209858	NA	0.571209858	NA	NA	NA	NA
33	Bexagliflozin:Ertugliflozin_high_dosage	0	0.517320128	NA	0.517320128	NA	NA	NA	NA
34	Bexagliflozin:Ertugliflozin_low_dosage	0	0.227069718	NA	0.227069718	NA	NA	NA	NA
35	Bexagliflozin:Exenatide	0	0.905949929	NA	0.905949929	NA	NA	NA	NA
36	Bexagliflozin:Inject_semaglutide_high_dosage	0	0.495244725	NA	0.495244725	NA	NA	NA	NA
37	Bexagliflozin:Inject_semaglutide_low_dosage	0	0.115450482	NA	0.115450482	NA	NA	NA	NA
38	Bexagliflozin:Inject_semaglutide_medium_dosage	0	0.829702233	NA	0.829702233	NA	NA	NA	NA
39	Bexagliflozin:Liraglutide	0	0.089167894	NA	0.089167894	NA	NA	NA	NA
40	Bexagliflozin:Lixisenatide	0	1.34245784	NA	1.34245784	NA	NA	NA	NA

41	Bexagliflozin:Oral_semaglutide	0	-0.000362327	NA	-0.000362327	NA	NA	NA	NA
42	Bexagliflozin:Placebo_or_Control	1	0.226702274	0.226702274	NA	NA	NA	NA	NA
43	Bexagliflozin:Sotagliflozin	0	0.555269107	NA	0.555269107	NA	NA	NA	NA
44	Bexagliflozin:Tirzepatide_high_dosage	0	-0.418496889	NA	-0.418496889	NA	NA	NA	NA
45	Bexagliflozin:Tirzepatide_low_dosage	0	0.415414499	NA	0.415414499	NA	NA	NA	NA
46	Canagliflozin_high_dosage:Canagliflozin_low_dosage	1	-0.652263424	-0.630704364	-0.722319775	0.091615411	-1.68366875	1.866899573	0.919434556
47	Canagliflozin_high_dosage:Dapagliflozin	0	-0.275394948	NA	-0.275394948	NA	NA	NA	NA
48	Canagliflozin_high_dosage:Dulaglutide	0	-0.629790569	NA	-0.629790569	NA	NA	NA	NA
49	Canagliflozin_high_dosage:Efpeglenatide_high_dosage	0	-0.930308217	NA	-0.930308217	NA	NA	NA	NA
50	Canagliflozin_high_dosage:Efpeglenatide_low_dosage	0	0.16659592	NA	0.16659592	NA	NA	NA	NA
51	Canagliflozin_high_dosage:Efpeglenatide_medium_dosage	0	-0.932016369	NA	-0.932016369	NA	NA	NA	NA
52	Canagliflozin_high_dosage:Empagliflozin_high_dosage	0	-0.112086258	NA	-0.112086258	NA	NA	NA	NA
53	Canagliflozin_high_dosage:Empagliflozin_low_dosage	0	-0.328533139	NA	-0.328533139	NA	NA	NA	NA
54	Canagliflozin_high_dosage:Ertugliflozin_high_dosage	0	-0.382422868	NA	-0.382422868	NA	NA	NA	NA
55	Canagliflozin_high_dosage:Ertugliflozin_low_dosage	0	-0.672673278	NA	-0.672673278	NA	NA	NA	NA
56	Canagliflozin_high_dosage:Exenatide	0	0.006206933	NA	0.006206933	NA	NA	NA	NA
57	Canagliflozin_high_dosage:Inject_semaglutide_high_dosage	0	-0.404498271	NA	-0.404498271	NA	NA	NA	NA
58	Canagliflozin_high_dosage:Inject_semaglutide_low_dosage	0	-0.784292515	NA	-0.784292515	NA	NA	NA	NA
59	Canagliflozin_high_dosage:Inject_semaglutide_medium_dosage	0	-0.070040763	NA	-0.070040763	NA	NA	NA	NA
60	Canagliflozin_high_dosage:Liraglutide	0	-0.810575102	NA	-0.810575102	NA	NA	NA	NA
61	Canagliflozin_high_dosage:Lixisenatide	0	0.442714843	NA	0.442714843	NA	NA	NA	NA
62	Canagliflozin_high_dosage:Oral_semaglutide	0	-0.900105323	NA	-0.900105323	NA	NA	NA	NA

63	Canagliflozin_high_dosage:Placebo_or_Control	3	-0.673040723	-0.642671132	-3.713985129	3.071313997	-3.146197152	9.288825146	0.332954893
64	Canagliflozin_high_dosage:Sotagliflozin	0	-0.344473889	NA	-0.344473889	NA	NA	NA	NA
65	Canagliflozin_high_dosage:Tirzepatide_high_dosage	0	-1.318239885	NA	-1.318239885	NA	NA	NA	NA
66	Canagliflozin_high_dosage:Tirzepatide_low_dosage	0	-0.484328498	NA	-0.484328498	NA	NA	NA	NA
67	Canagliflozin_low_dosage:Dapagliflozin	0	0.376868476	NA	0.376868476	NA	NA	NA	NA
68	Canagliflozin_low_dosage:Dulaglutide	0	0.022472855	NA	0.022472855	NA	NA	NA	NA
69	Canagliflozin_low_dosage:Efpeglenatide_high_dosage	0	-0.278044793	NA	-0.278044793	NA	NA	NA	NA
70	Canagliflozin_low_dosage:Efpeglenatide_low_dosage	0	0.818859344	NA	0.818859344	NA	NA	NA	NA
71	Canagliflozin_low_dosage:Efpeglenatide_medium_dosage	0	-0.279752945	NA	-0.279752945	NA	NA	NA	NA
72	Canagliflozin_low_dosage:Empagliflozin_high_dosage	0	0.540177166	NA	0.540177166	NA	NA	NA	NA
73	Canagliflozin_low_dosage:Empagliflozin_low_dosage	0	0.323730285	NA	0.323730285	NA	NA	NA	NA
74	Canagliflozin_low_dosage:Ertugliflozin_high_dosage	0	0.269840556	NA	0.269840556	NA	NA	NA	NA
75	Canagliflozin_low_dosage:Ertugliflozin_low_dosage	0	-0.020409854	NA	-0.020409854	NA	NA	NA	NA
76	Canagliflozin_low_dosage:Exenatide	0	0.658470357	NA	0.658470357	NA	NA	NA	NA
77	Canagliflozin_low_dosage:Inject_semaglutide_high_dosage	0	0.247765153	NA	0.247765153	NA	NA	NA	NA
78	Canagliflozin_low_dosage:Inject_semaglutide_low_dosage	0	-0.132029091	NA	-0.132029091	NA	NA	NA	NA
79	Canagliflozin_low_dosage:Inject_semaglutide_medium_dosage	0	0.582222661	NA	0.582222661	NA	NA	NA	NA
80	Canagliflozin_low_dosage:Liraglutide	0	-0.158311678	NA	-0.158311678	NA	NA	NA	NA
81	Canagliflozin_low_dosage:Lixisenatide	0	1.094978267	NA	1.094978267	NA	NA	NA	NA
82	Canagliflozin_low_dosage:Oral_semaglutide	0	-0.247841899	NA	-0.247841899	NA	NA	NA	NA
83	Canagliflozin_low_dosage:Placebo_or_Control	3	-0.020777299	0.021236715	-0.420447399	0.441684114	-1.856667015	2.740035243	0.706430167
84	Canagliflozin_low_dosage:Sotagliflozin	0	0.307789535	NA	0.307789535	NA	NA	NA	NA

85	Canagliflozin_low_dosage:Tirzepatide_high_dosage	0	-0.665976461	NA	-0.665976461	NA	NA	NA	NA
86	Canagliflozin_low_dosage:Tirzepatide_low_dosage	0	0.167934926	NA	0.167934926	NA	NA	NA	NA
87	Dapagliflozin:Dulaglutide	0	-0.354395621	NA	-0.354395621	NA	NA	NA	NA
88	Dapagliflozin:Efpeglenatide_high_dosage	0	-0.654913269	NA	-0.654913269	NA	NA	NA	NA
89	Dapagliflozin:Efpeglenatide_low_dosage	0	0.441990867	NA	0.441990867	NA	NA	NA	NA
90	Dapagliflozin:Efpeglenatide_medium_dosage	0	-0.656621421	NA	-0.656621421	NA	NA	NA	NA
91	Dapagliflozin:Empagliflozin_high_dosage	0	0.16330869	NA	0.16330869	NA	NA	NA	NA
92	Dapagliflozin:Empagliflozin_low_dosage	0	-0.053138191	NA	-0.053138191	NA	NA	NA	NA
93	Dapagliflozin:Ertugliflozin_high_dosage	0	-0.10702792	NA	-0.10702792	NA	NA	NA	NA
94	Dapagliflozin:Ertugliflozin_low_dosage	0	-0.39727833	NA	-0.39727833	NA	NA	NA	NA
95	Dapagliflozin:Exenatide	0	0.28160188	NA	0.28160188	NA	NA	NA	NA
96	Dapagliflozin:Inject_semaglutide_high_dosage	0	-0.129103324	NA	-0.129103324	NA	NA	NA	NA
97	Dapagliflozin:Inject_semaglutide_low_dosage	0	-0.508897567	NA	-0.508897567	NA	NA	NA	NA
98	Dapagliflozin:Inject_semaglutide_medium_dosage	0	0.205354184	NA	0.205354184	NA	NA	NA	NA
99	Dapagliflozin:Liraglutide	0	-0.535180155	NA	-0.535180155	NA	NA	NA	NA
100	Dapagliflozin:Lixisenatide	0	0.718109791	NA	0.718109791	NA	NA	NA	NA
101	Dapagliflozin:Oral_semaglutide	0	-0.624710375	NA	-0.624710375	NA	NA	NA	NA
102	Dapagliflozin:Placebo_or_Control	8	-0.397645775	-0.397645775	NA	NA	NA	NA	NA
103	Dapagliflozin:Sotagliflozin	0	-0.069078942	NA	-0.069078942	NA	NA	NA	NA
104	Dapagliflozin:Tirzepatide_high_dosage	0	-1.042844937	NA	-1.042844937	NA	NA	NA	NA
105	Dapagliflozin:Tirzepatide_low_dosage	0	-0.20893355	NA	-0.20893355	NA	NA	NA	NA
106	Dulaglutide:Efpeglenatide_high_dosage	1	-0.300517648	-1.13565356	-0.231024746	-0.904628814	-4.239944408	2.430686779	0.595005946

107	Dulaglutide:Efpeglenatide_low_dosage	0	0.796386489	NA	0.796386489	NA	NA	NA	NA
108	Dulaglutide:Efpeglenatide_medium_dosage	1	-0.3022258	-1.066089097	-0.239220246	-0.826868851	-4.160918585	2.507180883	0.626906561
109	Dulaglutide:Empagliflozin_high_dosage	0	0.517704311	NA	0.517704311	NA	NA	NA	NA
110	Dulaglutide:Empagliflozin_low_dosage	0	0.30125743	NA	0.30125743	NA	NA	NA	NA
111	Dulaglutide:Ertugliflozin_high_dosage	0	0.247367701	NA	0.247367701	NA	NA	NA	NA
112	Dulaglutide:Ertugliflozin_low_dosage	0	-0.042882709	NA	-0.042882709	NA	NA	NA	NA
113	Dulaglutide:Exenatide	0	0.635997502	NA	0.635997502	NA	NA	NA	NA
114	Dulaglutide:Inject_semaglutide_high_dosage	0	0.225292298	NA	0.225292298	NA	NA	NA	NA
115	Dulaglutide:Inject_semaglutide_low_dosage	0	-0.154501946	NA	-0.154501946	NA	NA	NA	NA
116	Dulaglutide:Inject_semaglutide_medium_dosage	0	0.559749805	NA	0.559749805	NA	NA	NA	NA
117	Dulaglutide:Liraglutide	1	-0.180784534	-1.098612289	-0.165512944	-0.933099345	-4.164262596	2.298063906	0.57139321
118	Dulaglutide:Lixisenatide	0	1.072505412	NA	1.072505412	NA	NA	NA	NA
119	Dulaglutide:Oral_semaglutide	0	-0.270314754	NA	-0.270314754	NA	NA	NA	NA
120	Dulaglutide:Placebo_or_Control	1	-0.043250154	-0.026578125	-0.588807898	0.562229773	-1.335932556	2.460392102	0.561553735
121	Dulaglutide:Sotagliflozin	0	0.28531668	NA	0.28531668	NA	NA	NA	NA
122	Dulaglutide:Tirzepatide_high_dosage	1	-0.688449316	-0.457241378	-0.723294853	0.266053476	-3.185261188	3.71736814	0.879905613
123	Dulaglutide:Tirzepatide_low_dosage	0	0.145462071	NA	0.145462071	NA	NA	NA	NA
124	Efpeglenatide_high_dosage:Efpeglenatide_low_dosage	0	1.096904137	NA	1.096904137	NA	NA	NA	NA
125	Efpeglenatide_high_dosage:Efpeglenatide_medium_dosage	3	-0.001708152	0.025488071	-1.784547432	1.810035504	-4.552175238	8.172246245	0.577113725
126	Efpeglenatide_high_dosage:Empagliflozin_high_dosage	0	0.818221959	NA	0.818221959	NA	NA	NA	NA
127	Efpeglenatide_high_dosage:Empagliflozin_low_dosage	0	0.601775078	NA	0.601775078	NA	NA	NA	NA
128	Efpeglenatide_high_dosage:Ertugliflozin_high_dosage	0	0.547885349	NA	0.547885349	NA	NA	NA	NA

129	Efpeglenatide_high_dosage:Ertugliflozin_low_dosage	0	0.257634939	NA	0.257634939	NA	NA	NA	NA
130	Efpeglenatide_high_dosage:Exenatide	0	0.93651515	NA	0.93651515	NA	NA	NA	NA
131	Efpeglenatide_high_dosage:Inject_semaglutide_high_dosage	0	0.525809946	NA	0.525809946	NA	NA	NA	NA
132	Efpeglenatide_high_dosage:Inject_semaglutide_low_dosage	0	0.146015702	NA	0.146015702	NA	NA	NA	NA
133	Efpeglenatide_high_dosage:Inject_semaglutide_medium_dosage	0	0.860267454	NA	0.860267454	NA	NA	NA	NA
134	Efpeglenatide_high_dosage:Liraglutide	0	0.119733115	NA	0.119733115	NA	NA	NA	NA
135	Efpeglenatide_high_dosage:Lixisenatide	0	1.37302306	NA	1.37302306	NA	NA	NA	NA
136	Efpeglenatide_high_dosage:Oral_semaglutide	0	0.030202894	NA	0.030202894	NA	NA	NA	NA
137	Efpeglenatide_high_dosage:Placebo_or_Control	1	0.257267495	0.199928029	0.734130172	-0.534202142	-3.232949292	2.164545007	0.698042614
138	Efpeglenatide_high_dosage:Sotagliflozin	0	0.585834328	NA	0.585834328	NA	NA	NA	NA
139	Efpeglenatide_high_dosage:Tirzepatide_high_dosage	0	-0.387931668	NA	-0.387931668	NA	NA	NA	NA
140	Efpeglenatide_high_dosage:Tirzepatide_low_dosage	0	0.445979719	NA	0.445979719	NA	NA	NA	NA
141	Efpeglenatide_low_dosage:Efpeglenatide_medium_dosage	1	-1.098612289	-1.098612289	NA	NA	NA	NA	NA
142	Efpeglenatide_low_dosage:Empagliflozin_high_dosage	0	-0.278682177	NA	-0.278682177	NA	NA	NA	NA
143	Efpeglenatide_low_dosage:Empagliflozin_low_dosage	0	-0.495129058	NA	-0.495129058	NA	NA	NA	NA
144	Efpeglenatide_low_dosage:Ertugliflozin_high_dosage	0	-0.549018788	NA	-0.549018788	NA	NA	NA	NA
145	Efpeglenatide_low_dosage:Ertugliflozin_low_dosage	0	-0.839269198	NA	-0.839269198	NA	NA	NA	NA
146	Efpeglenatide_low_dosage:Exenatide	0	-0.160388987	NA	-0.160388987	NA	NA	NA	NA
147	Efpeglenatide_low_dosage:Inject_semaglutide_high_dosage	0	-0.571094191	NA	-0.571094191	NA	NA	NA	NA
148	Efpeglenatide_low_dosage:Inject_semaglutide_low_dosage	0	-0.950888435	NA	-0.950888435	NA	NA	NA	NA
149	Efpeglenatide_low_dosage:Inject_semaglutide_medium_dosage	0	-0.236636683	NA	-0.236636683	NA	NA	NA	NA
150	Efpeglenatide_low_dosage:Liraglutide	0	-0.977171022	NA	-0.977171022	NA	NA	NA	NA

151	Efpeglenatide_low_dosage:Lixisenatide	0	0.276118923	NA	0.276118923	NA	NA	NA	NA
152	Efpeglenatide_low_dosage:Oral_semaglutide	0	-1.066701243	NA	-1.066701243	NA	NA	NA	NA
153	Efpeglenatide_low_dosage:Placebo_or_Control	0	-0.839636642	NA	-0.839636642	NA	NA	NA	NA
154	Efpeglenatide_low_dosage:Sotagliflozin	0	-0.511069809	NA	-0.511069809	NA	NA	NA	NA
155	Efpeglenatide_low_dosage:Tirzepatide_high_dosage	0	-1.484835805	NA	-1.484835805	NA	NA	NA	NA
156	Efpeglenatide_low_dosage:Tirzepatide_low_dosage	0	-0.650924418	NA	-0.650924418	NA	NA	NA	NA
157	Efpeglenatide_medium_dosage:Empagliflozin_high_dosage	0	0.819930111	NA	0.819930111	NA	NA	NA	NA
158	Efpeglenatide_medium_dosage:Empagliflozin_low_dosage	0	0.60348323	NA	0.60348323	NA	NA	NA	NA
159	Efpeglenatide_medium_dosage:Ertugliflozin_high_dosage	0	0.549593501	NA	0.549593501	NA	NA	NA	NA
160	Efpeglenatide_medium_dosage:Ertugliflozin_low_dosage	0	0.259343091	NA	0.259343091	NA	NA	NA	NA
161	Efpeglenatide_medium_dosage:Exenatide	0	0.938223302	NA	0.938223302	NA	NA	NA	NA
162	Efpeglenatide_medium_dosage:Inject_semaglutide_high_dosage	0	0.527518098	NA	0.527518098	NA	NA	NA	NA
163	Efpeglenatide_medium_dosage:Inject_semaglutide_low_dosage	0	0.147723854	NA	0.147723854	NA	NA	NA	NA
164	Efpeglenatide_medium_dosage:Inject_semaglutide_medium_dosage	0	0.861975606	NA	0.861975606	NA	NA	NA	NA
165	Efpeglenatide_medium_dosage:Liraglutide	0	0.121441267	NA	0.121441267	NA	NA	NA	NA
166	Efpeglenatide_medium_dosage:Lixisenatide	0	1.374731212	NA	1.374731212	NA	NA	NA	NA
167	Efpeglenatide_medium_dosage:Oral_semaglutide	0	0.031911046	NA	0.031911046	NA	NA	NA	NA
168	Efpeglenatide_medium_dosage:Placebo_or_Control	2	0.258975646	0.175455431	1.141075057	-0.965619627	-3.923577283	1.99233803	0.522285403
169	Efpeglenatide_medium_dosage:Sotagliflozin	0	0.58754248	NA	0.58754248	NA	NA	NA	NA
170	Efpeglenatide_medium_dosage:Tirzepatide_high_dosage	0	-0.386223516	NA	-0.386223516	NA	NA	NA	NA
171	Efpeglenatide_medium_dosage:Tirzepatide_low_dosage	0	0.447687871	NA	0.447687871	NA	NA	NA	NA
172	Empagliflozin_high_dosage:Empagliflozin_low_dosage	1	-0.216446881	-0.315380954	0.164354558	-0.479735512	-1.788505474	0.82903445	0.472489971

173	Empagliflozin_high_dosage:Ertugliflozin_high_dosage	0	-0.270336611	NA	-0.270336611	NA	NA	NA	NA
174	Empagliflozin_high_dosage:Ertugliflozin_low_dosage	0	-0.56058702	NA	-0.56058702	NA	NA	NA	NA
175	Empagliflozin_high_dosage:Exenatide	0	0.11829319	NA	0.11829319	NA	NA	NA	NA
176	Empagliflozin_high_dosage:Inject_semaglutide_high_dosage	0	-0.292412014	NA	-0.292412014	NA	NA	NA	NA
177	Empagliflozin_high_dosage:Inject_semaglutide_low_dosage	0	-0.672206257	NA	-0.672206257	NA	NA	NA	NA
178	Empagliflozin_high_dosage:Inject_semaglutide_medium_dosage	0	0.042045494	NA	0.042045494	NA	NA	NA	NA
179	Empagliflozin_high_dosage:Liraglutide	0	-0.698488845	NA	-0.698488845	NA	NA	NA	NA
180	Empagliflozin_high_dosage:Lixisenatide	0	0.554801101	NA	0.554801101	NA	NA	NA	NA
181	Empagliflozin_high_dosage:Oral_semaglutide	1	-0.788019065	0.002447982	-1.694028529	1.696476511	-2.369136643	5.762089664	0.413446897
182	Empagliflozin_high_dosage:Placebo_or_Control	1	-0.560954465	-0.530812566	-0.725600426	0.194787859	-1.255669623	1.645245341	0.792387515
183	Empagliflozin_high_dosage:Sotagliflozin	0	-0.232387632	NA	-0.232387632	NA	NA	NA	NA
184	Empagliflozin_high_dosage:Tirzepatide_high_dosage	0	-1.206153627	NA	-1.206153627	NA	NA	NA	NA
185	Empagliflozin_high_dosage:Tirzepatide_low_dosage	0	-0.37224224	NA	-0.37224224	NA	NA	NA	NA
186	Empagliflozin_low_dosage:Ertugliflozin_high_dosage	0	-0.053889729	NA	-0.053889729	NA	NA	NA	NA
187	Empagliflozin_low_dosage:Ertugliflozin_low_dosage	0	-0.344140139	NA	-0.344140139	NA	NA	NA	NA
188	Empagliflozin_low_dosage:Exenatide	0	0.334740071	NA	0.334740071	NA	NA	NA	NA
189	Empagliflozin_low_dosage:Inject_semaglutide_high_dosage	0	-0.075965133	NA	-0.075965133	NA	NA	NA	NA
190	Empagliflozin_low_dosage:Inject_semaglutide_low_dosage	0	-0.455759376	NA	-0.455759376	NA	NA	NA	NA
191	Empagliflozin_low_dosage:Inject_semaglutide_medium_dosage	0	0.258492375	NA	0.258492375	NA	NA	NA	NA
192	Empagliflozin_low_dosage:Liraglutide	0	-0.482041964	NA	-0.482041964	NA	NA	NA	NA
193	Empagliflozin_low_dosage:Lixisenatide	0	0.771247982	NA	0.771247982	NA	NA	NA	NA
194	Empagliflozin_low_dosage:Oral_semaglutide	0	-0.571572184	NA	-0.571572184	NA	NA	NA	NA

195	Empagliflozin_low_dosage:Placebo_or_Control	4	-0.344507584	-0.346566496	3.432330654	-3.77889715	-12.8350402	5.277245897	0.413446897
196	Empagliflozin_low_dosage:Sotagliflozin	0	-0.01594075	NA	-0.01594075	NA	NA	NA	NA
197	Empagliflozin_low_dosage:Tirzepatide_high_dosage	0	-0.989706746	NA	-0.989706746	NA	NA	NA	NA
198	Empagliflozin_low_dosage:Tirzepatide_low_dosage	0	-0.155795359	NA	-0.155795359	NA	NA	NA	NA
199	Ertugliflozin_high_dosage:Ertugliflozin_low_dosage	1	-0.29025041	-0.29025041	NA	NA	NA	NA	NA
200	Ertugliflozin_high_dosage:Exenatide	0	0.388629801	NA	0.388629801	NA	NA	NA	NA
201	Ertugliflozin_high_dosage:Inject_semaglutide_high_dosage	0	-0.022075403	NA	-0.022075403	NA	NA	NA	NA
202	Ertugliflozin_high_dosage:Inject_semaglutide_low_dosage	0	-0.401869647	NA	-0.401869647	NA	NA	NA	NA
203	Ertugliflozin_high_dosage:Inject_semaglutide_medium_dosage	0	0.312382105	NA	0.312382105	NA	NA	NA	NA
204	Ertugliflozin_high_dosage:Liraglutide	0	-0.428152234	NA	-0.428152234	NA	NA	NA	NA
205	Ertugliflozin_high_dosage:Lixisenatide	0	0.825137711	NA	0.825137711	NA	NA	NA	NA
206	Ertugliflozin_high_dosage:Oral_semaglutide	0	-0.517682455	NA	-0.517682455	NA	NA	NA	NA
207	Ertugliflozin_high_dosage:Placebo_or_Control	1	-0.290617854	-0.290617854	NA	NA	NA	NA	NA
208	Ertugliflozin_high_dosage:Sotagliflozin	0	0.037948979	NA	0.037948979	NA	NA	NA	NA
209	Ertugliflozin_high_dosage:Tirzepatide_high_dosage	0	-0.935817017	NA	-0.935817017	NA	NA	NA	NA
210	Ertugliflozin_high_dosage:Tirzepatide_low_dosage	0	-0.10190563	NA	-0.10190563	NA	NA	NA	NA
211	Ertugliflozin_low_dosage:Exenatide	0	0.678880211	NA	0.678880211	NA	NA	NA	NA
212	Ertugliflozin_low_dosage:Inject_semaglutide_high_dosage	0	0.268175007	NA	0.268175007	NA	NA	NA	NA
213	Ertugliflozin_low_dosage:Inject_semaglutide_low_dosage	0	-0.111619237	NA	-0.111619237	NA	NA	NA	NA
214	Ertugliflozin_low_dosage:Inject_semaglutide_medium_dosage	0	0.602632515	NA	0.602632515	NA	NA	NA	NA
215	Ertugliflozin_low_dosage:Liraglutide	0	-0.137901824	NA	-0.137901824	NA	NA	NA	NA
216	Ertugliflozin_low_dosage:Lixisenatide	0	1.115388121	NA	1.115388121	NA	NA	NA	NA

217	Ertugliflozin_low_dosage:Oral_semaglutide	0	-0.227432045	NA	-0.227432045	NA	NA	NA	NA
218	Ertugliflozin_low_dosage:Placebo_or_Control	1	-0.000367444	-0.000367444	NA	NA	NA	NA	NA
219	Ertugliflozin_low_dosage:Sotagliflozin	0	0.328199389	NA	0.328199389	NA	NA	NA	NA
220	Ertugliflozin_low_dosage:Tirzepatide_high_dosage	0	-0.645566607	NA	-0.645566607	NA	NA	NA	NA
221	Ertugliflozin_low_dosage:Tirzepatide_low_dosage	0	0.18834478	NA	0.18834478	NA	NA	NA	NA
222	Exenatide:Inject_semaglutide_high_dosage	0	-0.410705204	NA	-0.410705204	NA	NA	NA	NA
223	Exenatide:Inject_semaglutide_low_dosage	0	-0.790499448	NA	-0.790499448	NA	NA	NA	NA
224	Exenatide:Inject_semaglutide_medium_dosage	0	-0.076247696	NA	-0.076247696	NA	NA	NA	NA
225	Exenatide:Liraglutide	0	-0.816782035	NA	-0.816782035	NA	NA	NA	NA
226	Exenatide:Lixisenatide	0	0.43650791	NA	0.43650791	NA	NA	NA	NA
227	Exenatide:Oral_semaglutide	0	-0.906312256	NA	-0.906312256	NA	NA	NA	NA
228	Exenatide:Placebo_or_Control	3	-0.679247655	-0.679247655	NA	NA	NA	NA	NA
229	Exenatide:Sotagliflozin	0	-0.350680822	NA	-0.350680822	NA	NA	NA	NA
230	Exenatide:Tirzepatide_high_dosage	0	-1.324446818	NA	-1.324446818	NA	NA	NA	NA
231	Exenatide:Tirzepatide_low_dosage	0	-0.490535431	NA	-0.490535431	NA	NA	NA	NA
232	Inject_semaglutide_high_dosage:Inject_semaglutide_low_dosage	0	-0.379794243	NA	-0.379794243	NA	NA	NA	NA
233	Inject_semaglutide_high_dosage:Inject_semaglutide_medium_dosage	1	0.334457508	0.405465108	0.321607054	0.083858054	-1.866329723	2.03404583	0.932835061
234	Inject_semaglutide_high_dosage:Liraglutide	1	-0.406076831	1.114422895	-0.42688866	1.541311554	-1.690665091	4.7732882	0.34994507
235	Inject_semaglutide_high_dosage:Lixisenatide	0	0.847213114	NA	0.847213114	NA	NA	NA	NA
236	Inject_semaglutide_high_dosage:Oral_semaglutide	0	-0.495607052	NA	-0.495607052	NA	NA	NA	NA
237	Inject_semaglutide_high_dosage:Placebo_or_Control	4	-0.268542451	-0.274385957	0.026610365	-0.300996322	-2.277609268	1.675616624	0.765351309
238	Inject_semaglutide_high_dosage:Sotagliflozin	0	0.060024382	NA	0.060024382	NA	NA	NA	NA

239	Inject_semaglutide_high_dosage:Tirzepatide_high_dosage	0	-0.913741614	NA	-0.913741614	NA	NA	NA	NA
240	Inject_semaglutide_high_dosage:Tirzepatide_low_dosage	0	-0.079830226	NA	-0.079830226	NA	NA	NA	NA
241	Inject_semaglutide_low_dosage:Inject_semaglutide_medium_dosage	2	0.714251751	0.894447144	-0.351161573	1.245608716	-0.831130459	3.322347891	0.239767529
242	Inject_semaglutide_low_dosage:Liraglutide	1	-0.026282588	-0.837589046	5.04E-05	-0.837639493	-4.096508913	2.421229927	0.61441814
243	Inject_semaglutide_low_dosage:Lixisenatide	0	1.227007358	NA	1.227007358	NA	NA	NA	NA
244	Inject_semaglutide_low_dosage:Oral_semaglutide	0	-0.115812808	NA	-0.115812808	NA	NA	NA	NA
245	Inject_semaglutide_low_dosage:Placebo_or_Control	2	0.111251792	0.129468118	-0.221474042	0.350942159	-1.951991275	2.653875593	0.765186261
246	Inject_semaglutide_low_dosage:Sotagliflozin	0	0.439818626	NA	0.439818626	NA	NA	NA	NA
247	Inject_semaglutide_low_dosage:Tirzepatide_high_dosage	0	-0.53394737	NA	-0.53394737	NA	NA	NA	NA
248	Inject_semaglutide_low_dosage:Tirzepatide_low_dosage	0	0.299964017	NA	0.299964017	NA	NA	NA	NA
249	Inject_semaglutide_medium_dosage:Liraglutide	0	-0.740534339	NA	-0.740534339	NA	NA	NA	NA
250	Inject_semaglutide_medium_dosage:Lixisenatide	0	0.512755606	NA	0.512755606	NA	NA	NA	NA
251	Inject_semaglutide_medium_dosage:Oral_semaglutide	0	-0.83006456	NA	-0.83006456	NA	NA	NA	NA
252	Inject_semaglutide_medium_dosage:Placebo_or_Control	3	-0.602999959	-0.639786405	-0.37956783	-0.260218575	-2.155155657	1.634718508	0.787815588
253	Inject_semaglutide_medium_dosage:Sotagliflozin	0	-0.274433126	NA	-0.274433126	NA	NA	NA	NA
254	Inject_semaglutide_medium_dosage:Tirzepatide_high_dosage	1	-1.248199122	-0.405997731	-1.404502888	0.998505156	-2.488556986	4.485567299	0.574642658
255	Inject_semaglutide_medium_dosage:Tirzepatide_low_dosage	0	-0.414287734	NA	-0.414287734	NA	NA	NA	NA
256	Liraglutide:Lixisenatide	0	1.253289945	NA	1.253289945	NA	NA	NA	NA
257	Liraglutide:Oral_semaglutide	0	-0.089530221	NA	-0.089530221	NA	NA	NA	NA
258	Liraglutide:Placebo_or_Control	3	0.13753438	0.145104586	-0.155254686	0.300359272	-1.335314777	1.936033322	0.718915395
259	Liraglutide:Sotagliflozin	0	0.466101213	NA	0.466101213	NA	NA	NA	NA
260	Liraglutide:Tirzepatide_high_dosage	0	-0.507664783	NA	-0.507664783	NA	NA	NA	NA

261	Liraglutide:Tirzepatide_low_dosage	0	0.326246605	NA	0.326246605	NA	NA	NA	NA
262	Lixisenatide:Oral_semaglutide	0	-1.342820166	NA	-1.342820166	NA	NA	NA	NA
263	Lixisenatide:Placebo_or_Control	1	-1.115755566	-1.115755566	NA	NA	NA	NA	NA
264	Lixisenatide:Sotagliflozin	0	-0.787188732	NA	-0.787188732	NA	NA	NA	NA
265	Lixisenatide:Tirzepatide_high_dosage	0	-1.760954728	NA	-1.760954728	NA	NA	NA	NA
266	Lixisenatide:Tirzepatide_low_dosage	0	-0.927043341	NA	-0.927043341	NA	NA	NA	NA
267	Oral_semaglutide:Placebo_or_Control	1	0.227064601	1.104345017	-0.592131494	1.696476511	-2.369136643	5.762089664	0.413446897
268	Oral_semaglutide:Sotagliflozin	0	0.555631434	NA	0.555631434	NA	NA	NA	NA
269	Oral_semaglutide:Tirzepatide_high_dosage	0	-0.418134562	NA	-0.418134562	NA	NA	NA	NA
270	Oral_semaglutide:Tirzepatide_low_dosage	0	0.415776825	NA	0.415776825	NA	NA	NA	NA
271	Sotagliflozin:Placebo_or_Control	2	-0.328566833	-0.328566833	NA	NA	NA	NA	NA
272	Tirzepatide_high_dosage:Placebo_or_Control	5	0.645199163	0.821141732	0.092595377	0.728546354	-1.912460765	3.369553473	0.588732111
273	Tirzepatide_low_dosage:Placebo_or_Control	0	-0.188712225	NA	-0.188712225	NA	NA	NA	NA
274	Sotagliflozin:Tirzepatide_high_dosage	0	-0.973765996	NA	-0.973765996	NA	NA	NA	NA
275	Sotagliflozin:Tirzepatide_low_dosage	0	-0.139854609	NA	-0.139854609	NA	NA	NA	NA
276	Tirzepatide_high_dosage:Tirzepatide_low_dosage	3	0.833911387	0.833911387	NA	NA	NA	NA	NA

Table S7C: inconsistency within the network meta-analysis of safety profile: drop-out rate

	Comparison	No.Studies	NMA	Direct	Indirect	Difference	Diff_95CI_lower	Diff_95CI_upper	*p* value
1	Albiglutide:Bexagliflozin	0	0.168761461	NA	0.168761461	NA	NA	NA	NA
2	Albiglutide:Canagliflozin_high_dosage	0	0.239326586	NA	0.239326586	NA	NA	NA	NA
3	Albiglutide:Canagliflozin_low_dosage	0	0.139943756	NA	0.139943756	NA	NA	NA	NA
4	Albiglutide:Dapagliflozin	0	0.04711762	NA	0.04711762	NA	NA	NA	NA
5	Albiglutide:Dulaglutide	0	-0.003763008	NA	-0.003763008	NA	NA	NA	NA
6	Albiglutide:Efpeglenatide_high_dosage	0	-0.198324494	NA	-0.198324494	NA	NA	NA	NA
7	Albiglutide:Efpeglenatide_low_dosage	0	0.074179343	NA	0.074179343	NA	NA	NA	NA
8	Albiglutide:Efpeglenatide_medium_dosage	0	-0.061699272	NA	-0.061699272	NA	NA	NA	NA
9	Albiglutide:Empagliflozin_high_dosage	0	-0.033394573	NA	-0.033394573	NA	NA	NA	NA
10	Albiglutide:Empagliflozin_low_dosage	0	0.026607176	NA	0.026607176	NA	NA	NA	NA
11	Albiglutide:Ertugliflozin_high_dosage	0	-0.111425201	NA	-0.111425201	NA	NA	NA	NA
12	Albiglutide:Ertugliflozin_low_dosage	0	-0.049552366	NA	-0.049552366	NA	NA	NA	NA
13	Albiglutide:Exenatide	0	-0.232934493	NA	-0.232934493	NA	NA	NA	NA
14	Albiglutide:Inject_semaglutide_high_dosage	0	0.051466782	NA	0.051466782	NA	NA	NA	NA
15	Albiglutide:Inject_semaglutide_low_dosage	0	0.015065088	NA	0.015065088	NA	NA	NA	NA
16	Albiglutide:Inject_semaglutide_medium_dosage	0	0.257714332	NA	0.257714332	NA	NA	NA	NA
17	Albiglutide:Liraglutide	1	-0.019267852	-0.176557852	0.064894459	-0.241452311	-0.838692677	0.355788055	0.428142542
18	Albiglutide:Lixisenatide	0	-0.101478093	NA	-0.101478093	NA	NA	NA	NA
19	Albiglutide:Oral_semaglutide	0	-0.158601236	NA	-0.158601236	NA	NA	NA	NA
20	Albiglutide:Placebo_or_Control	2	-0.114465289	-0.057711774	-0.299164085	0.241452311	-0.355788055	0.838692677	0.428142542

21	Albiglutide:Sotagliflozin	0	0.059597369	NA	0.059597369	NA	NA	NA	NA
22	Albiglutide:Tirzepatide_high_dosage	0	0.362147233	NA	0.362147233	NA	NA	NA	NA
23	Albiglutide:Tirzepatide_low_dosage	0	0.466159785	NA	0.466159785	NA	NA	NA	NA
24	Bexagliflozin:Canagliflozin_high_dosage	0	0.070565125	NA	0.070565125	NA	NA	NA	NA
25	Bexagliflozin:Canagliflozin_low_dosage	0	-0.028817705	NA	-0.028817705	NA	NA	NA	NA
26	Bexagliflozin:Dapagliflozin	0	-0.121643841	NA	-0.121643841	NA	NA	NA	NA
27	Bexagliflozin:Dulaglutide	0	-0.17252447	NA	-0.17252447	NA	NA	NA	NA
28	Bexagliflozin:Efpeglenatide_high_dosage	0	-0.367085955	NA	-0.367085955	NA	NA	NA	NA
29	Bexagliflozin:Efpeglenatide_low_dosage	0	-0.094582118	NA	-0.094582118	NA	NA	NA	NA
30	Bexagliflozin:Efpeglenatide_medium_dosage	0	-0.230460734	NA	-0.230460734	NA	NA	NA	NA
31	Bexagliflozin:Empagliflozin_high_dosage	0	-0.202156034	NA	-0.202156034	NA	NA	NA	NA
32	Bexagliflozin:Empagliflozin_low_dosage	0	-0.142154286	NA	-0.142154286	NA	NA	NA	NA
33	Bexagliflozin:Ertugliflozin_high_dosage	0	-0.280186663	NA	-0.280186663	NA	NA	NA	NA
34	Bexagliflozin:Ertugliflozin_low_dosage	0	-0.218313828	NA	-0.218313828	NA	NA	NA	NA
35	Bexagliflozin:Exenatide	0	-0.401695954	NA	-0.401695954	NA	NA	NA	NA
36	Bexagliflozin:Inject_semaglutide_high_dosage	0	-0.117294679	NA	-0.117294679	NA	NA	NA	NA
37	Bexagliflozin:Inject_semaglutide_low_dosage	0	-0.153696374	NA	-0.153696374	NA	NA	NA	NA
38	Bexagliflozin:Inject_semaglutide_medium_dosage	0	0.08895287	NA	0.08895287	NA	NA	NA	NA
39	Bexagliflozin:Liraglutide	0	-0.188029313	NA	-0.188029313	NA	NA	NA	NA
40	Bexagliflozin:Lixisenatide	0	-0.270239555	NA	-0.270239555	NA	NA	NA	NA
41	Bexagliflozin:Oral_semaglutide	0	-0.327362697	NA	-0.327362697	NA	NA	NA	NA
42	Bexagliflozin:Placebo_or_Control	2	-0.28322675	-0.28322675	NA	NA	NA	NA	NA

43	Bexagliflozin:Sotagliflozin	0	-0.109164092	NA	-0.109164092	NA	NA	NA	NA
44	Bexagliflozin:Tirzepatide_high_dosage	0	0.193385772	NA	0.193385772	NA	NA	NA	NA
45	Bexagliflozin:Tirzepatide_low_dosage	0	0.297398323	NA	0.297398323	NA	NA	NA	NA
46	Canagliflozin_high_dosage:Canagliflozin_low_dosage	3	-0.09938283	-0.099660929	-0.098083287	-0.001577642	-0.707380488	0.704225204	0.99650448
47	Canagliflozin_high_dosage:Dapagliflozin	0	-0.192208965	NA	-0.192208965	NA	NA	NA	NA
48	Canagliflozin_high_dosage:Dulaglutide	0	-0.243089594	NA	-0.243089594	NA	NA	NA	NA
49	Canagliflozin_high_dosage:Efpeglenatide_high_dosage	0	-0.43765108	NA	-0.43765108	NA	NA	NA	NA
50	Canagliflozin_high_dosage:Efpeglenatide_low_dosage	0	-0.165147242	NA	-0.165147242	NA	NA	NA	NA
51	Canagliflozin_high_dosage:Efpeglenatide_medium_dosage	0	-0.301025858	NA	-0.301025858	NA	NA	NA	NA
52	Canagliflozin_high_dosage:Empagliflozin_high_dosage	0	-0.272721159	NA	-0.272721159	NA	NA	NA	NA
53	Canagliflozin_high_dosage:Empagliflozin_low_dosage	0	-0.21271941	NA	-0.21271941	NA	NA	NA	NA
54	Canagliflozin_high_dosage:Ertugliflozin_high_dosage	0	-0.350751787	NA	-0.350751787	NA	NA	NA	NA
55	Canagliflozin_high_dosage:Ertugliflozin_low_dosage	0	-0.288878952	NA	-0.288878952	NA	NA	NA	NA
56	Canagliflozin_high_dosage:Exenatide	0	-0.472261079	NA	-0.472261079	NA	NA	NA	NA
57	Canagliflozin_high_dosage:Inject_semaglutide_high_dosage	0	-0.187859804	NA	-0.187859804	NA	NA	NA	NA
58	Canagliflozin_high_dosage:Inject_semaglutide_low_dosage	0	-0.224261498	NA	-0.224261498	NA	NA	NA	NA
59	Canagliflozin_high_dosage:Inject_semaglutide_medium_dosage	0	0.018387746	NA	0.018387746	NA	NA	NA	NA
60	Canagliflozin_high_dosage:Liraglutide	0	-0.258594438	NA	-0.258594438	NA	NA	NA	NA
61	Canagliflozin_high_dosage:Lixisenatide	0	-0.340804679	NA	-0.340804679	NA	NA	NA	NA
62	Canagliflozin_high_dosage:Oral_semaglutide	0	-0.397927822	NA	-0.397927822	NA	NA	NA	NA
63	Canagliflozin_high_dosage:Placebo_or_Control	4	-0.353791875	-0.380799362	-0.026496228	-0.354303134	-1.299331586	0.590725318	0.462451977
64	Canagliflozin_high_dosage:Sotagliflozin	0	-0.179729217	NA	-0.179729217	NA	NA	NA	NA

65	Canagliflozin_high_dosage:Tirzepatide_high_dosage	0	0.122820647	NA	0.122820647	NA	NA	NA	NA
66	Canagliflozin_high_dosage:Tirzepatide_low_dosage	0	0.226833199	NA	0.226833199	NA	NA	NA	NA
67	Canagliflozin_low_dosage:Dapagliflozin	0	-0.092826136	NA	-0.092826136	NA	NA	NA	NA
68	Canagliflozin_low_dosage:Dulaglutide	0	-0.143706765	NA	-0.143706765	NA	NA	NA	NA
69	Canagliflozin_low_dosage:Efpeglenatide_high_dosage	0	-0.33826825	NA	-0.33826825	NA	NA	NA	NA
70	Canagliflozin_low_dosage:Efpeglenatide_low_dosage	0	-0.065764413	NA	-0.065764413	NA	NA	NA	NA
71	Canagliflozin_low_dosage:Efpeglenatide_medium_dosage	0	-0.201643029	NA	-0.201643029	NA	NA	NA	NA
72	Canagliflozin_low_dosage:Empagliflozin_high_dosage	0	-0.173338329	NA	-0.173338329	NA	NA	NA	NA
73	Canagliflozin_low_dosage:Empagliflozin_low_dosage	0	-0.11333658	NA	-0.11333658	NA	NA	NA	NA
74	Canagliflozin_low_dosage:Ertugliflozin_high_dosage	0	-0.251368957	NA	-0.251368957	NA	NA	NA	NA
75	Canagliflozin_low_dosage:Ertugliflozin_low_dosage	0	-0.189496123	NA	-0.189496123	NA	NA	NA	NA
76	Canagliflozin_low_dosage:Exenatide	0	-0.372878249	NA	-0.372878249	NA	NA	NA	NA
77	Canagliflozin_low_dosage:Inject_semaglutide_high_dosage	0	-0.088476974	NA	-0.088476974	NA	NA	NA	NA
78	Canagliflozin_low_dosage:Inject_semaglutide_low_dosage	0	-0.124878669	NA	-0.124878669	NA	NA	NA	NA
79	Canagliflozin_low_dosage:Inject_semaglutide_medium_dosage	0	0.117770576	NA	0.117770576	NA	NA	NA	NA
80	Canagliflozin_low_dosage:Liraglutide	0	-0.159211608	NA	-0.159211608	NA	NA	NA	NA
81	Canagliflozin_low_dosage:Lixisenatide	0	-0.24142185	NA	-0.24142185	NA	NA	NA	NA
82	Canagliflozin_low_dosage:Oral_semaglutide	0	-0.298544992	NA	-0.298544992	NA	NA	NA	NA
83	Canagliflozin_low_dosage:Placebo_or_Control	6	-0.254409045	-0.272717257	0.204265745	-0.476983002	-1.659287672	0.705321668	0.429108609
84	Canagliflozin_low_dosage:Sotagliflozin	0	-0.080346387	NA	-0.080346387	NA	NA	NA	NA
85	Canagliflozin_low_dosage:Tirzepatide_high_dosage	0	0.222203477	NA	0.222203477	NA	NA	NA	NA
86	Canagliflozin_low_dosage:Tirzepatide_low_dosage	0	0.326216028	NA	0.326216028	NA	NA	NA	NA

87	Dapagliflozin:Dulaglutide	0	-0.050880629	NA	-0.050880629	NA	NA	NA	NA
88	Dapagliflozin:Efpeglenatide_high_dosage	0	-0.245442115	NA	-0.245442115	NA	NA	NA	NA
89	Dapagliflozin:Efpeglenatide_low_dosage	0	0.027061723	NA	0.027061723	NA	NA	NA	NA
90	Dapagliflozin:Efpeglenatide_medium_dosage	0	-0.108816893	NA	-0.108816893	NA	NA	NA	NA
91	Dapagliflozin:Empagliflozin_high_dosage	0	-0.080512194	NA	-0.080512194	NA	NA	NA	NA
92	Dapagliflozin:Empagliflozin_low_dosage	0	-0.020510445	NA	-0.020510445	NA	NA	NA	NA
93	Dapagliflozin:Ertugliflozin_high_dosage	0	-0.158542822	NA	-0.158542822	NA	NA	NA	NA
94	Dapagliflozin:Ertugliflozin_low_dosage	0	-0.096669987	NA	-0.096669987	NA	NA	NA	NA
95	Dapagliflozin:Exenatide	0	-0.280052113	NA	-0.280052113	NA	NA	NA	NA
96	Dapagliflozin:Inject_semaglutide_high_dosage	0	0.004349162	NA	0.004349162	NA	NA	NA	NA
97	Dapagliflozin:Inject_semaglutide_low_dosage	0	-0.032052533	NA	-0.032052533	NA	NA	NA	NA
98	Dapagliflozin:Inject_semaglutide_medium_dosage	0	0.210596711	NA	0.210596711	NA	NA	NA	NA
99	Dapagliflozin:Liraglutide	0	-0.066385473	NA	-0.066385473	NA	NA	NA	NA
100	Dapagliflozin:Lixisenatide	0	-0.148595714	NA	-0.148595714	NA	NA	NA	NA
101	Dapagliflozin:Oral_semaglutide	0	-0.205718857	NA	-0.205718857	NA	NA	NA	NA
102	Dapagliflozin:Placebo_or_Control	9	-0.161582909	-0.161582909	NA	NA	NA	NA	NA
103	Dapagliflozin:Sotagliflozin	0	0.012479749	NA	0.012479749	NA	NA	NA	NA
104	Dapagliflozin:Tirzepatide_high_dosage	0	0.315029612	NA	0.315029612	NA	NA	NA	NA
105	Dapagliflozin:Tirzepatide_low_dosage	0	0.419042164	NA	0.419042164	NA	NA	NA	NA
106	Dulaglutide:Efpeglenatide_high_dosage	1	-0.194561486	-0.380215048	0.001067689	-0.381282737	-1.013453906	0.250888432	0.237159188
107	Dulaglutide:Efpeglenatide_low_dosage	0	0.077942352	NA	0.077942352	NA	NA	NA	NA
108	Dulaglutide:Efpeglenatide_medium_dosage	1	-0.057936264	0.043823744	-0.161649348	0.205473092	-0.429185709	0.840131893	0.525724909

109	Dulaglutide:Empagliflozin_high_dosage	0	-0.029631565	NA	-0.029631565	NA	NA	NA	NA
110	Dulaglutide:Empagliflozin_low_dosage	0	0.030370184	NA	0.030370184	NA	NA	NA	NA
111	Dulaglutide:Ertugliflozin_high_dosage	0	-0.107662193	NA	-0.107662193	NA	NA	NA	NA
112	Dulaglutide:Ertugliflozin_low_dosage	0	-0.045789358	NA	-0.045789358	NA	NA	NA	NA
113	Dulaglutide:Exenatide	0	-0.229171485	NA	-0.229171485	NA	NA	NA	NA
114	Dulaglutide:Inject_semaglutide_high_dosage	0	0.05522979	NA	0.05522979	NA	NA	NA	NA
115	Dulaglutide:Inject_semaglutide_low_dosage	0	0.018828096	NA	0.018828096	NA	NA	NA	NA
116	Dulaglutide:Inject_semaglutide_medium_dosage	0	0.26147734	NA	0.26147734	NA	NA	NA	NA
117	Dulaglutide:Liraglutide	1	-0.015504844	-0.032789823	-0.00921066	-0.023579163	-0.731876675	0.684718349	0.947977315
118	Dulaglutide:Lixisenatide	0	-0.097715085	NA	-0.097715085	NA	NA	NA	NA
119	Dulaglutide:Oral_semaglutide	0	-0.154838228	NA	-0.154838228	NA	NA	NA	NA
120	Dulaglutide:Placebo_or_Control	3	-0.11070228	0.023852281	-0.217070243	0.240922524	-0.318072253	0.799917301	0.398261794
121	Dulaglutide:Sotagliflozin	0	0.063360378	NA	0.063360378	NA	NA	NA	NA
122	Dulaglutide:Tirzepatide_high_dosage	1	0.365910241	-0.234689542	0.435820857	-0.6705104	-1.873064395	0.532043596	0.274472409
123	Dulaglutide:Tirzepatide_low_dosage	1	0.469922793	-0.202940844	0.610521473	-0.813462317	-2.064248597	0.437323963	0.20242123
124	Efpeglenatide_high_dosage:Efpeglenatide_low_dosage	1	0.272503838	0.146603474	0.600123534	-0.45352006	-1.872398866	0.965358745	0.531007642
125	Efpeglenatide_high_dosage:Efpeglenatide_medium_dosage	3	0.136625222	0.136479916	NA	NA	NA	NA	NA
126	Efpeglenatide_high_dosage:Empagliflozin_high_dosage	0	0.164929921	NA	0.164929921	NA	NA	NA	NA
127	Efpeglenatide_high_dosage:Empagliflozin_low_dosage	0	0.22493167	NA	0.22493167	NA	NA	NA	NA
128	Efpeglenatide_high_dosage:Ertugliflozin_high_dosage	0	0.086899293	NA	0.086899293	NA	NA	NA	NA
129	Efpeglenatide_high_dosage:Ertugliflozin_low_dosage	0	0.148772128	NA	0.148772128	NA	NA	NA	NA
130	Efpeglenatide_high_dosage:Exenatide	0	-0.034609999	NA	-0.034609999	NA	NA	NA	NA

131	Efpeglenatide_high_dosage:Inject_semaglutide_high_dosage	0	0.249791276	NA	0.249791276	NA	NA	NA	NA
132	Efpeglenatide_high_dosage:Inject_semaglutide_low_dosage	0	0.213389582	NA	0.213389582	NA	NA	NA	NA
133	Efpeglenatide_high_dosage:Inject_semaglutide_medium_dosage	0	0.456038826	NA	0.456038826	NA	NA	NA	NA
134	Efpeglenatide_high_dosage:Liraglutide	0	0.179056642	NA	0.179056642	NA	NA	NA	NA
135	Efpeglenatide_high_dosage:Lixisenatide	0	0.096846401	NA	0.096846401	NA	NA	NA	NA
136	Efpeglenatide_high_dosage:Oral_semaglutide	0	0.039723258	NA	0.039723258	NA	NA	NA	NA
137	Efpeglenatide_high_dosage:Placebo_or_Control	2	0.083859205	-0.012996443	0.371963363	-0.384959806	-1.015512205	0.245592594	0.231469703
138	Efpeglenatide_high_dosage:Sotagliflozin	0	0.257921863	NA	0.257921863	NA	NA	NA	NA
139	Efpeglenatide_high_dosage:Tirzepatide_high_dosage	0	0.560471727	NA	0.560471727	NA	NA	NA	NA
140	Efpeglenatide_high_dosage:Tirzepatide_low_dosage	0	0.664484279	NA	0.664484279	NA	NA	NA	NA
141	Efpeglenatide_low_dosage:Efpeglenatide_medium_dosage	1	-0.135878616	-0.284258584	0.279056171	-0.563314755	-2.004104332	0.877474822	0.443497992
142	Efpeglenatide_low_dosage:Empagliflozin_high_dosage	0	-0.107573917	NA	-0.107573917	NA	NA	NA	NA
143	Efpeglenatide_low_dosage:Empagliflozin_low_dosage	0	-0.047572168	NA	-0.047572168	NA	NA	NA	NA
144	Efpeglenatide_low_dosage:Ertugliflozin_high_dosage	0	-0.185604545	NA	-0.185604545	NA	NA	NA	NA
145	Efpeglenatide_low_dosage:Ertugliflozin_low_dosage	0	-0.12373171	NA	-0.12373171	NA	NA	NA	NA
146	Efpeglenatide_low_dosage:Exenatide	0	-0.307113836	NA	-0.307113836	NA	NA	NA	NA
147	Efpeglenatide_low_dosage:Inject_semaglutide_high_dosage	0	-0.022712561	NA	-0.022712561	NA	NA	NA	NA
148	Efpeglenatide_low_dosage:Inject_semaglutide_low_dosage	0	-0.059114256	NA	-0.059114256	NA	NA	NA	NA
149	Efpeglenatide_low_dosage:Inject_semaglutide_medium_dosage	0	0.183534988	NA	0.183534988	NA	NA	NA	NA
150	Efpeglenatide_low_dosage:Liraglutide	0	-0.093447196	NA	-0.093447196	NA	NA	NA	NA
151	Efpeglenatide_low_dosage:Lixisenatide	0	-0.175657437	NA	-0.175657437	NA	NA	NA	NA
152	Efpeglenatide_low_dosage:Oral_semaglutide	0	-0.23278058	NA	-0.23278058	NA	NA	NA	NA

153	Efpeglenatide_low_dosage:Placebo_or_Control	1	-0.188644632	-0.159025994	-0.267626007	0.108600013	-1.323664223	1.540864249	0.881859549
154	Efpeglenatide_low_dosage:Sotagliflozin	0	-0.014581974	NA	-0.014581974	NA	NA	NA	NA
155	Efpeglenatide_low_dosage:Tirzepatide_high_dosage	0	0.287967889	NA	0.287967889	NA	NA	NA	NA
156	Efpeglenatide_low_dosage:Tirzepatide_low_dosage	0	0.391980441	NA	0.391980441	NA	NA	NA	NA
157	Efpeglenatide_medium_dosage:Empagliflozin_high_dosage	0	0.028304699	NA	0.028304699	NA	NA	NA	NA
158	Efpeglenatide_medium_dosage:Empagliflozin_low_dosage	0	0.088306448	NA	0.088306448	NA	NA	NA	NA
159	Efpeglenatide_medium_dosage:Ertugliflozin_high_dosage	0	-0.049725929	NA	-0.049725929	NA	NA	NA	NA
160	Efpeglenatide_medium_dosage:Ertugliflozin_low_dosage	0	0.012146906	NA	0.012146906	NA	NA	NA	NA
161	Efpeglenatide_medium_dosage:Exenatide	0	-0.17123522	NA	-0.17123522	NA	NA	NA	NA
162	Efpeglenatide_medium_dosage:Inject_semaglutide_high_dosage	0	0.113166054	NA	0.113166054	NA	NA	NA	NA
163	Efpeglenatide_medium_dosage:Inject_semaglutide_low_dosage	0	0.07676436	NA	0.07676436	NA	NA	NA	NA
164	Efpeglenatide_medium_dosage:Inject_semaglutide_medium_dosage	0	0.319413604	NA	0.319413604	NA	NA	NA	NA
165	Efpeglenatide_medium_dosage:Liraglutide	0	0.04243142	NA	0.04243142	NA	NA	NA	NA
166	Efpeglenatide_medium_dosage:Lixisenatide	0	-0.039778821	NA	-0.039778821	NA	NA	NA	NA
167	Efpeglenatide_medium_dosage:Oral_semaglutide	0	-0.096901964	NA	-0.096901964	NA	NA	NA	NA
168	Efpeglenatide_medium_dosage:Placebo_or_Control	2	-0.052766016	-0.002605732	-0.205456605	0.202850873	-0.43223544	0.837937187	0.531298031
169	Efpeglenatide_medium_dosage:Sotagliflozin	0	0.121296642	NA	0.121296642	NA	NA	NA	NA
170	Efpeglenatide_medium_dosage:Tirzepatide_high_dosage	0	0.423846505	NA	0.423846505	NA	NA	NA	NA
171	Efpeglenatide_medium_dosage:Tirzepatide_low_dosage	0	0.527859057	NA	0.527859057	NA	NA	NA	NA
172	Empagliflozin_high_dosage:Empagliflozin_low_dosage	3	0.060001749	0.108550922	-0.012343759	0.120894681	-0.317106741	0.558896104	0.588522608
173	Empagliflozin_high_dosage:Ertugliflozin_high_dosage	0	-0.078030628	NA	-0.078030628	NA	NA	NA	NA
174	Empagliflozin_high_dosage:Ertugliflozin_low_dosage	0	-0.016157793	NA	-0.016157793	NA	NA	NA	NA

175	Empagliflozin_high_dosage:Exenatide	0	-0.19953992	NA	-0.19953992	NA	NA	NA	NA
176	Empagliflozin_high_dosage:Inject_semaglutide_high_dosage	0	0.084861355	NA	0.084861355	NA	NA	NA	NA
177	Empagliflozin_high_dosage:Inject_semaglutide_low_dosage	0	0.048459661	NA	0.048459661	NA	NA	NA	NA
178	Empagliflozin_high_dosage:Inject_semaglutide_medium_dosage	0	0.291108905	NA	0.291108905	NA	NA	NA	NA
179	Empagliflozin_high_dosage:Liraglutide	0	0.014126721	NA	0.014126721	NA	NA	NA	NA
180	Empagliflozin_high_dosage:Lixisenatide	0	-0.06808352	NA	-0.06808352	NA	NA	NA	NA
181	Empagliflozin_high_dosage:Oral_semaglutide	1	-0.125206663	0.770638797	-0.552974263	1.323613061	0.364185546	2.283040575	0.006852277
182	Empagliflozin_high_dosage:Placebo_or_Control	4	-0.081070716	-0.18725899	0.508671605	-0.695930595	-1.240761347	-0.151099842	0.012296204
183	Empagliflozin_high_dosage:Sotagliflozin	0	0.092991943	NA	0.092991943	NA	NA	NA	NA
184	Empagliflozin_high_dosage:Tirzepatide_high_dosage	0	0.395541806	NA	0.395541806	NA	NA	NA	NA
185	Empagliflozin_high_dosage:Tirzepatide_low_dosage	0	0.499554358	NA	0.499554358	NA	NA	NA	NA
186	Empagliflozin_low_dosage:Ertugliflozin_high_dosage	0	-0.138032377	NA	-0.138032377	NA	NA	NA	NA
187	Empagliflozin_low_dosage:Ertugliflozin_low_dosage	0	-0.076159542	NA	-0.076159542	NA	NA	NA	NA
188	Empagliflozin_low_dosage:Exenatide	0	-0.259541669	NA	-0.259541669	NA	NA	NA	NA
189	Empagliflozin_low_dosage:Inject_semaglutide_high_dosage	0	0.024859606	NA	0.024859606	NA	NA	NA	NA
190	Empagliflozin_low_dosage:Inject_semaglutide_low_dosage	0	-0.011542088	NA	-0.011542088	NA	NA	NA	NA
191	Empagliflozin_low_dosage:Inject_semaglutide_medium_dosage	0	0.231107156	NA	0.231107156	NA	NA	NA	NA
192	Empagliflozin_low_dosage:Liraglutide	0	-0.045875028	NA	-0.045875028	NA	NA	NA	NA
193	Empagliflozin_low_dosage:Lixisenatide	0	-0.128085269	NA	-0.128085269	NA	NA	NA	NA
194	Empagliflozin_low_dosage:Oral_semaglutide	0	-0.185208412	NA	-0.185208412	NA	NA	NA	NA
195	Empagliflozin_low_dosage:Placebo_or_Control	8	-0.141072465	-0.14939361	0.178465083	-0.327858693	-1.226572019	0.570854633	0.474601341
196	Empagliflozin_low_dosage:Sotagliflozin	0	0.032990194	NA	0.032990194	NA	NA	NA	NA

197	Empagliflozin_low_dosage:Tirzepatide_high_dosage	0	0.335540057	NA	0.335540057	NA	NA	NA	NA
198	Empagliflozin_low_dosage:Tirzepatide_low_dosage	0	0.439552609	NA	0.439552609	NA	NA	NA	NA
199	Ertugliflozin_high_dosage:Ertugliflozin_low_dosage	2	0.061872835	0.061406278	NA	NA	NA	NA	NA
200	Ertugliflozin_high_dosage:Exenatide	0	-0.121509292	NA	-0.121509292	NA	NA	NA	NA
201	Ertugliflozin_high_dosage:Inject_semaglutide_high_dosage	0	0.162891983	NA	0.162891983	NA	NA	NA	NA
202	Ertugliflozin_high_dosage:Inject_semaglutide_low_dosage	0	0.126490289	NA	0.126490289	NA	NA	NA	NA
203	Ertugliflozin_high_dosage:Inject_semaglutide_medium_dosage	0	0.369139533	NA	0.369139533	NA	NA	NA	NA
204	Ertugliflozin_high_dosage:Liraglutide	0	0.092157349	NA	0.092157349	NA	NA	NA	NA
205	Ertugliflozin_high_dosage:Lixisenatide	0	0.009947108	NA	0.009947108	NA	NA	NA	NA
206	Ertugliflozin_high_dosage:Oral_semaglutide	0	-0.047176035	NA	-0.047176035	NA	NA	NA	NA
207	Ertugliflozin_high_dosage:Placebo_or_Control	2	-0.003040088	-0.00232661	NA	NA	NA	NA	NA
208	Ertugliflozin_high_dosage:Sotagliflozin	0	0.171022571	NA	0.171022571	NA	NA	NA	NA
209	Ertugliflozin_high_dosage:Tirzepatide_high_dosage	0	0.473572434	NA	0.473572434	NA	NA	NA	NA
210	Ertugliflozin_high_dosage:Tirzepatide_low_dosage	0	0.577584986	NA	0.577584986	NA	NA	NA	NA
211	Ertugliflozin_low_dosage:Exenatide	0	-0.183382127	NA	-0.183382127	NA	NA	NA	NA
212	Ertugliflozin_low_dosage:Inject_semaglutide_high_dosage	0	0.101019148	NA	0.101019148	NA	NA	NA	NA
213	Ertugliflozin_low_dosage:Inject_semaglutide_low_dosage	0	0.064617454	NA	0.064617454	NA	NA	NA	NA
214	Ertugliflozin_low_dosage:Inject_semaglutide_medium_dosage	0	0.307266698	NA	0.307266698	NA	NA	NA	NA
215	Ertugliflozin_low_dosage:Liraglutide	0	0.030284514	NA	0.030284514	NA	NA	NA	NA
216	Ertugliflozin_low_dosage:Lixisenatide	0	-0.051925727	NA	-0.051925727	NA	NA	NA	NA
217	Ertugliflozin_low_dosage:Oral_semaglutide	0	-0.10904887	NA	-0.10904887	NA	NA	NA	NA
218	Ertugliflozin_low_dosage:Placebo_or_Control	2	-0.064912923	-0.063969597	NA	NA	NA	NA	NA

219	Ertugliflozin_low_dosage:Sotagliflozin	0	0.109149736	NA	0.109149736	NA	NA	NA	NA
220	Ertugliflozin_low_dosage:Tirzepatide_high_dosage	0	0.411699599	NA	0.411699599	NA	NA	NA	NA
221	Ertugliflozin_low_dosage:Tirzepatide_low_dosage	0	0.515712151	NA	0.515712151	NA	NA	NA	NA
222	Exenatide:Inject_semaglutide_high_dosage	0	0.284401275	NA	0.284401275	NA	NA	NA	NA
223	Exenatide:Inject_semaglutide_low_dosage	0	0.247999581	NA	0.247999581	NA	NA	NA	NA
224	Exenatide:Inject_semaglutide_medium_dosage	0	0.490648825	NA	0.490648825	NA	NA	NA	NA
225	Exenatide:Liraglutide	0	0.213666641	NA	0.213666641	NA	NA	NA	NA
226	Exenatide:Lixisenatide	0	0.1314564	NA	0.1314564	NA	NA	NA	NA
227	Exenatide:Oral_semaglutide	0	0.074333257	NA	0.074333257	NA	NA	NA	NA
228	Exenatide:Placebo_or_Control	3	0.118469204	0.118469204	NA	NA	NA	NA	NA
229	Exenatide:Sotagliflozin	0	0.292531862	NA	0.292531862	NA	NA	NA	NA
230	Exenatide:Tirzepatide_high_dosage	0	0.595081726	NA	0.595081726	NA	NA	NA	NA
231	Exenatide:Tirzepatide_low_dosage	0	0.699094277	NA	0.699094277	NA	NA	NA	NA
232	Inject_semaglutide_high_dosage:Inject_semaglutide_low_dosage	0	-0.036401694	NA	-0.036401694	NA	NA	NA	NA
233	Inject_semaglutide_high_dosage:Inject_semaglutide_medium_dosage	1	0.20624755	-0.002560821	0.275191286	-0.277752107	-1.241255974	0.68575176	0.572070628
234	Inject_semaglutide_high_dosage:Liraglutide	1	-0.070734634	-0.422272226	-0.036631438	-0.385640788	-1.541980932	0.770699356	0.513337145
235	Inject_semaglutide_high_dosage:Lixisenatide	0	-0.152944875	NA	-0.152944875	NA	NA	NA	NA
236	Inject_semaglutide_high_dosage:Oral_semaglutide	0	-0.210068018	NA	-0.210068018	NA	NA	NA	NA
237	Inject_semaglutide_high_dosage:Placebo_or_Control	4	-0.165932071	-0.141664803	-0.649214378	0.507549576	-0.780344976	1.795444127	0.439873353
238	Inject_semaglutide_high_dosage:Sotagliflozin	0	0.008130587	NA	0.008130587	NA	NA	NA	NA
239	Inject_semaglutide_high_dosage:Tirzepatide_high_dosage	0	0.310680451	NA	0.310680451	NA	NA	NA	NA
240	Inject_semaglutide_high_dosage:Tirzepatide_low_dosage	0	0.414693003	NA	0.414693003	NA	NA	NA	NA

241	Inject_semaglutide_low_dosage:Inject_semaglutide_medium_dosage	2	0.242649244	0.319163512	0.094449978	0.224713534	-0.679907392	1.12933446	0.626352306
242	Inject_semaglutide_low_dosage:Liraglutide	1	-0.03433294	-0.086644797	-0.02071681	-0.065927987	-1.049175611	0.917319637	0.895444614
243	Inject_semaglutide_low_dosage:Lixisenatide	0	-0.116543181	NA	-0.116543181	NA	NA	NA	NA
244	Inject_semaglutide_low_dosage:Oral_semaglutide	0	-0.173666324	NA	-0.173666324	NA	NA	NA	NA
245	Inject_semaglutide_low_dosage:Placebo_or_Control	3	-0.129530377	-0.124015169	-0.156432672	0.032417503	-0.935094187	0.999929193	0.947640026
246	Inject_semaglutide_low_dosage:Sotagliflozin	0	0.044532282	NA	0.044532282	NA	NA	NA	NA
247	Inject_semaglutide_low_dosage:Tirzepatide_high_dosage	0	0.347082145	NA	0.347082145	NA	NA	NA	NA
248	Inject_semaglutide_low_dosage:Tirzepatide_low_dosage	0	0.451094697	NA	0.451094697	NA	NA	NA	NA
249	Inject_semaglutide_medium_dosage:Liraglutide	0	-0.276982184	NA	-0.276982184	NA	NA	NA	NA
250	Inject_semaglutide_medium_dosage:Lixisenatide	0	-0.359192425	NA	-0.359192425	NA	NA	NA	NA
251	Inject_semaglutide_medium_dosage:Oral_semaglutide	0	-0.416315568	NA	-0.416315568	NA	NA	NA	NA
252	Inject_semaglutide_medium_dosage:Placebo_or_Control	3	-0.372179621	-0.325313541	-0.445060217	0.119746676	-0.565698312	0.805191663	0.732046806
253	Inject_semaglutide_medium_dosage:Sotagliflozin	0	-0.198116962	NA	-0.198116962	NA	NA	NA	NA
254	Inject_semaglutide_medium_dosage:Tirzepatide_high_dosage	1	0.104432901	0.021672878	0.164634375	-0.142961497	-0.890282016	0.604359023	0.707706056
255	Inject_semaglutide_medium_dosage:Tirzepatide_low_dosage	1	0.208445453	0.333769969	0.095455873	0.238314096	-0.691180803	1.167808996	0.615303854
256	Liraglutide:Lixisenatide	0	-0.082210241	NA	-0.082210241	NA	NA	NA	NA
257	Liraglutide:Oral_semaglutide	0	-0.139333384	NA	-0.139333384	NA	NA	NA	NA
258	Liraglutide:Placebo_or_Control	5	-0.095197437	-0.14388472	0.083740232	-0.227624952	-0.689660099	0.234410195	0.334249174
259	Liraglutide:Sotagliflozin	0	0.078865221	NA	0.078865221	NA	NA	NA	NA
260	Liraglutide:Tirzepatide_high_dosage	0	0.381415085	NA	0.381415085	NA	NA	NA	NA
261	Liraglutide:Tirzepatide_low_dosage	0	0.485427637	NA	0.485427637	NA	NA	NA	NA
262	Lixisenatide:Oral_semaglutide	0	-0.057123143	NA	-0.057123143	NA	NA	NA	NA

263	Lixisenatide:Placebo_or_Control	1	-0.012987196	-0.012987196	NA	NA	NA	NA	NA
264	Lixisenatide:Sotagliflozin	0	0.161075463	NA	0.161075463	NA	NA	NA	NA
265	Lixisenatide:Tirzepatide_high_dosage	0	0.463625326	NA	0.463625326	NA	NA	NA	NA
266	Lixisenatide:Tirzepatide_low_dosage	0	0.567637878	NA	0.567637878	NA	NA	NA	NA
267	Oral_semaglutide:Placebo_or_Control	3	0.044135947	0.414196826	-0.909416234	1.323613061	0.364185546	2.283040575	0.006852277
268	Oral_semaglutide:Sotagliflozin	0	0.218198605	NA	0.218198605	NA	NA	NA	NA
269	Oral_semaglutide:Tirzepatide_high_dosage	0	0.520748469	NA	0.520748469	NA	NA	NA	NA
270	Oral_semaglutide:Tirzepatide_low_dosage	0	0.624761021	NA	0.624761021	NA	NA	NA	NA
271	Sotagliflozin:Placebo_or_Control	4	-0.174062658	-0.174062658	NA	NA	NA	NA	NA
272	Tirzepatide_high_dosage:Placebo_or_Control	5	-0.476612522	-0.520403781	-0.265086067	-0.255317714	-0.924969271	0.414333843	0.45489789
273	Tirzepatide_low_dosage:Placebo_or_Control	2	-0.580625073	-0.582898769	-0.578167011	-0.004731758	-0.799876421	0.790412905	0.990694179
274	Sotagliflozin:Tirzepatide_high_dosage	0	0.302549863	NA	0.302549863	NA	NA	NA	NA
275	Sotagliflozin:Tirzepatide_low_dosage	0	0.406562415	NA	0.406562415	NA	NA	NA	NA
276	Tirzepatide_high_dosage:Tirzepatide_low_dosage	3	0.104012552	0.040574746	0.575436073	-0.534861327	-1.751301038	0.681578383	0.388806093

Abbreviation: 95%CIs: 95% confidence intervals; GLP-1 agonist: glucagon-like peptide-1 agonist; NA: not applicable; NMA: network meta-analysis; OR: odds ratio; RCT: randomized controlled trial; SGLT2 inhibitor: sodium–glucose cotransporter 2 inhibitor

Table S8A: GRADE of primary outcome: overall acute kidney injury/acute renal failure

	Comparison	No.Studies	Direct		Indirect		NMA	
			Estimate	Rate	Estimate	Rate	Estimate	Rate
1	Albiglutide:Bexagliflozin	0			-0.444341842	⊕⊕○○ Low	-0.444341842	⊕⊕○○ Low
2	Albiglutide:Canagliflozin_high_dosage	0			0.589767093	⊕⊕○○ Low	0.589767093	⊕⊕○○ Low
3	Albiglutide:Canagliflozin_low_dosage	0			-0.03811025	⊕⊕○○ Low	-0.03811025	⊕⊕○○ Low
4	Albiglutide:Dapagliflozin	0			0.231003525	⊕⊕○○ Low	0.231003525	⊕⊕○○ Low
5	Albiglutide:Dulaglutide	0			-0.103991072	⊕⊕○○ Low	-0.103991072	⊕⊕○○ Low
6	Albiglutide:Efpeglenatide_high_dosage	0			-0.375208035	⊕⊕○○ Low	-0.375208035	⊕⊕○○ Low
7	Albiglutide:Efpeglenatide_low_dosage	0			0.721707245	⊕⊕○○ Low	0.721707245	⊕⊕○○ Low
8	Albiglutide:Efpeglenatide_medium_dosage	0			-0.376905044	⊕⊕○○ Low	-0.376905044	⊕⊕○○ Low
9	Albiglutide:Empagliflozin_high_dosage	0			0.369608812	⊕⊕○○ Low	0.369608812	⊕⊕○○ Low
10	Albiglutide:Empagliflozin_low_dosage	0			0.189395548	⊕⊕○○ Low	0.189395548	⊕⊕○○ Low
11	Albiglutide:Ertugliflozin_high_dosage	0			0.176701322	⊕⊕○○ Low	0.176701322	⊕⊕○○ Low
12	Albiglutide:Ertugliflozin_low_dosage	0			-0.190252293	⊕⊕○○ Low	-0.190252293	⊕⊕○○ Low
13	Albiglutide:Exenatide	0			0.563203594	⊕⊕○○ Low	0.563203594	⊕⊕○○ Low
14	Albiglutide:Inject_semaglutide_high_dosage	0			0.152493797	⊕⊕○○ Low	0.152493797	⊕⊕○○ Low
15	Albiglutide:Inject_semaglutide_low_dosage	0			-0.227331387	⊕⊕○○ Low	-0.227331387	⊕⊕○○ Low
16	Albiglutide:Inject_semaglutide_medium_dosage	0			0.486791573	⊕⊕○○ Low	0.486791573	⊕⊕○○ Low
17	Albiglutide:Liraglutide	1	1.110927715	⊕⊕⊕⊕ High	-0.283294137	⊕⊕⊕○ Medium	-0.253778324	⊕⊕⊕⊕ High
18	Albiglutide:Lixisenatide	0			0.999711505	⊕⊕○○ Low	0.999711505	⊕⊕○○ Low
19	Albiglutide:Oral_semaglutide	0			0.071425379	⊕⊕○○ Low	0.071425379	⊕⊕○○ Low

20	Albiglutide:Placebo_or_Control	2	-0.136763693	⊕⊕⊕⊕ High	1.257458159	⊕⊕⊕○ Medium	-0.116044061	⊕⊕⊕⊕ High
21	Albiglutide:Sotagliflozin	0			-0.190830799	⊕⊕○○ Low	-0.190830799	⊕⊕○○ Low
22	Albiglutide:Tirzepatide_high_dosage	0			-0.765089232	⊕⊕○○ Low	-0.765089232	⊕⊕○○ Low
23	Albiglutide:Tirzepatide_low_dosage	0			0.068822155	⊕⊕○○ Low	0.068822155	⊕⊕○○ Low
24	Bexagliflozin:Canagliflozin_high_dosage	0			1.034108935	⊕⊕○○ Low	1.034108935	⊕⊕○○ Low
25	Bexagliflozin:Canagliflozin_low_dosage	0			0.406231593	⊕⊕○○ Low	0.406231593	⊕⊕○○ Low
26	Bexagliflozin:Dapagliflozin	0			0.675345367	⊕⊕○○ Low	0.675345367	⊕⊕○○ Low
27	Bexagliflozin:Dulaglutide	0			0.340350771	⊕⊕○○ Low	0.340350771	⊕⊕○○ Low
28	Bexagliflozin:Efpeglenatide_high_dosage	0			0.069133808	⊕⊕○○ Low	0.069133808	⊕⊕○○ Low
29	Bexagliflozin:Efpeglenatide_low_dosage	0			1.166049087	⊕⊕○○ Low	1.166049087	⊕⊕○○ Low
30	Bexagliflozin:Efpeglenatide_medium_dosage	0			0.067436798	⊕⊕○○ Low	0.067436798	⊕⊕○○ Low
31	Bexagliflozin:Empagliflozin_high_dosage	0			0.813950654	⊕⊕○○ Low	0.813950654	⊕⊕○○ Low
32	Bexagliflozin:Empagliflozin_low_dosage	0			0.63373739	⊕⊕○○ Low	0.63373739	⊕⊕○○ Low
33	Bexagliflozin:Ertugliflozin_high_dosage	0			0.621043164	⊕⊕○○ Low	0.621043164	⊕⊕○○ Low
34	Bexagliflozin:Ertugliflozin_low_dosage	0			0.254089549	⊕⊕○○ Low	0.254089549	⊕⊕○○ Low
35	Bexagliflozin:Exenatide	0			1.007545437	⊕⊕○○ Low	1.007545437	⊕⊕○○ Low
36	Bexagliflozin:Inject_semaglutide_high_dosage	0			0.59683564	⊕⊕○○ Low	0.59683564	⊕⊕○○ Low
37	Bexagliflozin:Inject_semaglutide_low_dosage	0			0.217010456	⊕⊕○○ Low	0.217010456	⊕⊕○○ Low
38	Bexagliflozin:Inject_semaglutide_medium_dosage	0			0.931133415	⊕⊕○○ Low	0.931133415	⊕⊕○○ Low
39	Bexagliflozin:Liraglutide	0			0.190563519	⊕⊕○○ Low	0.190563519	⊕⊕○○ Low
40	Bexagliflozin:Lixisenatide	0			1.444053347	⊕⊕○○ Low	1.444053347	⊕⊕○○ Low
41	Bexagliflozin:Oral_semaglutide	0			0.515767221	⊕⊕○○ Low	0.515767221	⊕⊕○○ Low

42	Bexagliflozin:Placebo_or_Control	2	0.328297781	⊕⊕⊕⊕ High			0.328297781	⊕⊕⊕○ Medium
43	Bexagliflozin:Sotagliflozin	0			0.253511044	⊕⊕○○ Low	0.253511044	⊕⊕○○ Low
44	Bexagliflozin:Tirzepatide_high_dosage	0			-0.32074739	⊕⊕○○ Low	-0.32074739	⊕⊕○○ Low
45	Bexagliflozin:Tirzepatide_low_dosage	0			0.513163998	⊕⊕○○ Low	0.513163998	⊕⊕○○ Low
46	Canagliflozin_high_dosage:Canagliflozin_low_dosage	2	-0.663210743	⊕⊕⊕⊕ High	-0.589231223	⊕⊕⊕⊕ High	-0.627877343	⊕⊕⊕⊕ High
47	Canagliflozin_high_dosage:Dapagliflozin	0			-0.358763568	⊕⊕○○ Low	-0.358763568	⊕⊕○○ Low
48	Canagliflozin_high_dosage:Dulaglutide	0			-0.693758165	⊕⊕○○ Low	-0.693758165	⊕⊕○○ Low
49	Canagliflozin_high_dosage:Efpeglenatide_high_dosage	0			-0.964975128	⊕⊕○○ Low	-0.964975128	⊕⊕○○ Low
50	Canagliflozin_high_dosage:Efpeglenatide_low_dosage	0			0.131940152	⊕⊕○○ Low	0.131940152	⊕⊕○○ Low
51	Canagliflozin_high_dosage:Efpeglenatide_medium_dosage	0			-0.966672137	⊕⊕○○ Low	-0.966672137	⊕⊕○○ Low
52	Canagliflozin_high_dosage:Empagliflozin_high_dosage	0			-0.220158281	⊕⊕○○ Low	-0.220158281	⊕⊕○○ Low
53	Canagliflozin_high_dosage:Empagliflozin_low_dosage	0			-0.400371545	⊕⊕○○ Low	-0.400371545	⊕⊕○○ Low
54	Canagliflozin_high_dosage:Ertugliflozin_high_dosage	0			-0.413065771	⊕⊕○○ Low	-0.413065771	⊕⊕○○ Low
55	Canagliflozin_high_dosage:Ertugliflozin_low_dosage	0			-0.780019386	⊕⊕○○ Low	-0.780019386	⊕⊕○○ Low
56	Canagliflozin_high_dosage:Exenatide	0			-0.026563499	⊕⊕○○ Low	-0.026563499	⊕⊕○○ Low
57	Canagliflozin_high_dosage:Inject_semaglutide_high_dosage	0			-0.437273296	⊕⊕○○ Low	-0.437273296	⊕⊕○○ Low
58	Canagliflozin_high_dosage:Inject_semaglutide_low_dosage	0			-0.81709848	⊕⊕○○ Low	-0.81709848	⊕⊕○○ Low
59	Canagliflozin_high_dosage:Inject_semaglutide_medium_dosage	0			-0.10297552	⊕⊕○○ Low	-0.10297552	⊕⊕○○ Low
60	Canagliflozin_high_dosage:Liraglutide	0			-0.843545417	⊕⊕○○ Low	-0.843545417	⊕⊕○○ Low
61	Canagliflozin_high_dosage:Lixisenatide	0			0.409944412	⊕⊕○○ Low	0.409944412	⊕⊕○○ Low
62	Canagliflozin_high_dosage:Oral_semaglutide	0			-0.518341714	⊕⊕○○ Low	-0.518341714	⊕⊕○○ Low
63	Canagliflozin_high_dosage:Placebo_or_Control	3	-0.642671132	⊕⊕⊕⊕ High	-1.160268614	⊕⊕⊕⊕ High	-0.705811154	⊕⊕⊕⊕ High

64	Canagliflozin_high_dosage:Sotagliflozin	0			-0.780597892	⊕⊕○○ Low	-0.780597892	⊕⊕○○ Low
65	Canagliflozin_high_dosage:Tirzepatide_high_dosage	0			-1.354856325	⊕⊕○○ Low	-1.354856325	⊕⊕○○ Low
66	Canagliflozin_high_dosage:Tirzepatide_low_dosage	0			-0.520944938	⊕⊕○○ Low	-0.520944938	⊕⊕○○ Low
67	Canagliflozin_low_dosage:Dapagliflozin	0			0.269113774	⊕⊕○○ Low	0.269113774	⊕⊕○○ Low
68	Canagliflozin_low_dosage:Dulaglutide	0			-0.065880822	⊕⊕○○ Low	-0.065880822	⊕⊕○○ Low
69	Canagliflozin_low_dosage:Efpeglenatide_high_dosage	0			-0.337097785	⊕⊕○○ Low	-0.337097785	⊕⊕○○ Low
70	Canagliflozin_low_dosage:Efpeglenatide_low_dosage	0			0.759817495	⊕⊕○○ Low	0.759817495	⊕⊕○○ Low
71	Canagliflozin_low_dosage:Efpeglenatide_medium_dosage	0			-0.338794794	⊕⊕○○ Low	-0.338794794	⊕⊕○○ Low
72	Canagliflozin_low_dosage:Empagliflozin_high_dosage	0			0.407719061	⊕⊕○○ Low	0.407719061	⊕⊕○○ Low
73	Canagliflozin_low_dosage:Empagliflozin_low_dosage	0			0.227505798	⊕⊕○○ Low	0.227505798	⊕⊕○○ Low
74	Canagliflozin_low_dosage:Ertugliflozin_high_dosage	0			0.214811572	⊕⊕○○ Low	0.214811572	⊕⊕○○ Low
75	Canagliflozin_low_dosage:Ertugliflozin_low_dosage	0			-0.152142044	⊕⊕○○ Low	-0.152142044	⊕⊕○○ Low
76	Canagliflozin_low_dosage:Exenatide	0			0.601313844	⊕⊕○○ Low	0.601313844	⊕⊕○○ Low
77	Canagliflozin_low_dosage:Inject_semaglutide_high_dosage	0			0.190604047	⊕⊕○○ Low	0.190604047	⊕⊕○○ Low
78	Canagliflozin_low_dosage:Inject_semaglutide_low_dosage	0			-0.189221137	⊕⊕○○ Low	-0.189221137	⊕⊕○○ Low
79	Canagliflozin_low_dosage:Inject_semaglutide_medium_dosage	0			0.524901823	⊕⊕○○ Low	0.524901823	⊕⊕○○ Low
80	Canagliflozin_low_dosage:Liraglutide	0			-0.215668074	⊕⊕○○ Low	-0.215668074	⊕⊕○○ Low
81	Canagliflozin_low_dosage:Lixisenatide	0			1.037821754	⊕⊕○○ Low	1.037821754	⊕⊕○○ Low
82	Canagliflozin_low_dosage:Oral_semaglutide	0			0.109535628	⊕⊕○○ Low	0.109535628	⊕⊕○○ Low
83	Canagliflozin_low_dosage:Placebo_or_Control	6	-0.075223935	⊕⊕⊕⊕ High	-0.227033942	⊕⊕⊕⊕ High	-0.077933811	⊕⊕⊕⊕ High
84	Canagliflozin_low_dosage:Sotagliflozin	0			-0.152720549	⊕⊕○○ Low	-0.152720549	⊕⊕○○ Low
85	Canagliflozin_low_dosage:Tirzepatide_high_dosage	0			-0.726978982	⊕⊕○○ Low	-0.726978982	⊕⊕○○ Low

86	Canagliflozin_low_dosage:Tirzepatide_low_dosage	0			0.106932405	⊕⊕○○ Low	0.106932405	⊕⊕○○ Low
87	Dapagliflozin:Dulaglutide	0			-0.334994596	⊕⊕○○ Low	-0.334994596	⊕⊕○○ Low
88	Dapagliflozin:Efpeglenatide_high_dosage	0			-0.606211559	⊕⊕○○ Low	-0.606211559	⊕⊕○○ Low
89	Dapagliflozin:Efpeglenatide_low_dosage	0			0.49070372	⊕⊕○○ Low	0.49070372	⊕⊕○○ Low
90	Dapagliflozin:Efpeglenatide_medium_dosage	0			-0.607908568	⊕⊕○○ Low	-0.607908568	⊕⊕○○ Low
91	Dapagliflozin:Empagliflozin_high_dosage	0			0.138605287	⊕⊕○○ Low	0.138605287	⊕⊕○○ Low
92	Dapagliflozin:Empagliflozin_low_dosage	0			-0.041607977	⊕⊕○○ Low	-0.041607977	⊕⊕○○ Low
93	Dapagliflozin:Ertugliflozin_high_dosage	0			-0.054302203	⊕⊕○○ Low	-0.054302203	⊕⊕○○ Low
94	Dapagliflozin:Ertugliflozin_low_dosage	0			-0.421255818	⊕⊕○○ Low	-0.421255818	⊕⊕○○ Low
95	Dapagliflozin:Exenatide	0			0.33220007	⊕⊕○○ Low	0.33220007	⊕⊕○○ Low
96	Dapagliflozin:Inject_semaglutide_high_dosage	0			-0.078509727	⊕⊕○○ Low	-0.078509727	⊕⊕○○ Low
97	Dapagliflozin:Inject_semaglutide_low_dosage	0			-0.458334911	⊕⊕○○ Low	-0.458334911	⊕⊕○○ Low
98	Dapagliflozin:Inject_semaglutide_medium_dosage	0			0.255788048	⊕⊕○○ Low	0.255788048	⊕⊕○○ Low
99	Dapagliflozin:Liraglutide	0			-0.484781848	⊕⊕○○ Low	-0.484781848	⊕⊕○○ Low
100	Dapagliflozin:Lixisenatide	0			0.76870798	⊕⊕○○ Low	0.76870798	⊕⊕○○ Low
101	Dapagliflozin:Oral_semaglutide	0			-0.159578146	⊕⊕○○ Low	-0.159578146	⊕⊕○○ Low
102	Dapagliflozin:Placebo_or_Control	9	-0.347047585	⊕⊕⊕⊕ High			-0.347047585	⊕⊕⊕○ Medium
103	Dapagliflozin:Sotagliflozin	0			-0.421834323	⊕⊕○○ Low	-0.421834323	⊕⊕○○ Low
104	Dapagliflozin:Tirzepatide_high_dosage	0			-0.996092756	⊕⊕○○ Low	-0.996092756	⊕⊕○○ Low
105	Dapagliflozin:Tirzepatide_low_dosage	0			-0.162181369	⊕⊕○○ Low	-0.162181369	⊕⊕○○ Low
106	Dulaglutide:Efpeglenatide_high_dosage	1	-1.13565356	⊕⊕⊕⊕ High	-0.199655169	⊕⊕⊕⊕ High	-0.271216963	⊕⊕⊕⊕ High
107	Dulaglutide:Efpeglenatide_low_dosage	0			0.825698317	⊕⊕○○ Low	0.825698317	⊕⊕○○ Low

108	Dulaglutide:Efpeglenatide_medium_dosage	1	-1.066089097	⊕⊕⊕⊕ High	-0.207829349	⊕⊕⊕⊕ High	-0.272913972	⊕⊕⊕⊕ High
109	Dulaglutide:Empagliflozin_high_dosage	0			0.473599883	⊕⊕○○ Low	0.473599883	⊕⊕○○ Low
110	Dulaglutide:Empagliflozin_low_dosage	0			0.29338662	⊕⊕○○ Low	0.29338662	⊕⊕○○ Low
111	Dulaglutide:Ertugliflozin_high_dosage	0			0.280692394	⊕⊕○○ Low	0.280692394	⊕⊕○○ Low
112	Dulaglutide:Ertugliflozin_low_dosage	0			-0.086261222	⊕⊕○○ Low	-0.086261222	⊕⊕○○ Low
113	Dulaglutide:Exenatide	0			0.667194666	⊕⊕○○ Low	0.667194666	⊕⊕○○ Low
114	Dulaglutide:Inject_semaglutide_high_dosage	0			0.256484869	⊕⊕○○ Low	0.256484869	⊕⊕○○ Low
115	Dulaglutide:Inject_semaglutide_low_dosage	0			-0.123340315	⊕⊕○○ Low	-0.123340315	⊕⊕○○ Low
116	Dulaglutide:Inject_semaglutide_medium_dosage	0			0.590782645	⊕⊕○○ Low	0.590782645	⊕⊕○○ Low
117	Dulaglutide:Liraglutide	1	-1.098612289	⊕⊕⊕⊕ High	-0.134399468	⊕⊕⊕⊕ High	-0.149787252	⊕⊕⊕⊕ High
118	Dulaglutide:Lixisenatide	0			1.103702577	⊕⊕○○ Low	1.103702577	⊕⊕○○ Low
119	Dulaglutide:Oral_semaglutide	0			0.175416451	⊕⊕○○ Low	0.175416451	⊕⊕○○ Low
120	Dulaglutide:Placebo_or_Control	2	0.004830449	⊕⊕⊕⊕ High	-0.588807898	⊕⊕⊕○ Medium	-0.012052989	⊕⊕⊕⊕ High
121	Dulaglutide:Sotagliflozin	0			-0.086839727	⊕⊕○○ Low	-0.086839727	⊕⊕○○ Low
122	Dulaglutide:Tirzepatide_high_dosage	1	-0.457241378	⊕⊕⊕⊕ High	-0.691736625	⊕⊕⊕⊕ High	-0.66109816	⊕⊕⊕⊕ High
123	Dulaglutide:Tirzepatide_low_dosage	0			0.172813227	⊕⊕○○ Low	0.172813227	⊕⊕○○ Low
124	Efpeglenatide_high_dosage:Efpeglenatide_low_dosage	0			1.096915279	⊕⊕○○ Low	1.096915279	⊕⊕○○ Low
125	Efpeglenatide_high_dosage:Efpeglenatide_medium_dosage	3	0.025488071	⊕⊕⊕⊕ High	-1.783805733	⊕⊕⊕○ Medium	-0.001697009	⊕⊕⊕⊕ High
126	Efpeglenatide_high_dosage:Empagliflozin_high_dosage	0			0.744816846	⊕⊕○○ Low	0.744816846	⊕⊕○○ Low
127	Efpeglenatide_high_dosage:Empagliflozin_low_dosage	0			0.564603583	⊕⊕○○ Low	0.564603583	⊕⊕○○ Low
128	Efpeglenatide_high_dosage:Ertugliflozin_high_dosage	0			0.551909357	⊕⊕○○ Low	0.551909357	⊕⊕○○ Low
129	Efpeglenatide_high_dosage:Ertugliflozin_low_dosage	0			0.184955741	⊕⊕○○ Low	0.184955741	⊕⊕○○ Low

130	Efpeglenatide_high_dosage:Exenatide	0			0.938411629	⊕⊕○○ Low	0.938411629	⊕⊕○○ Low
131	Efpeglenatide_high_dosage:Inject_semaglutide_high_dosage	0			0.527701832	⊕⊕○○ Low	0.527701832	⊕⊕○○ Low
132	Efpeglenatide_high_dosage:Inject_semaglutide_low_dosage	0			0.147876648	⊕⊕○○ Low	0.147876648	⊕⊕○○ Low
133	Efpeglenatide_high_dosage:Inject_semaglutide_medium_dosage	0			0.861999608	⊕⊕○○ Low	0.861999608	⊕⊕○○ Low
134	Efpeglenatide_high_dosage:Liraglutide	0			0.121429711	⊕⊕○○ Low	0.121429711	⊕⊕○○ Low
135	Efpeglenatide_high_dosage:Lixisenatide	0			1.374919539	⊕⊕○○ Low	1.374919539	⊕⊕○○ Low
136	Efpeglenatide_high_dosage:Oral_semaglutide	0			0.446633413	⊕⊕○○ Low	0.446633413	⊕⊕○○ Low
137	Efpeglenatide_high_dosage:Placebo_or_Control	1	0.199928029	⊕⊕⊕⊕ High	0.751695653	⊕⊕⊕⊕ High	0.259163974	⊕⊕⊕⊕ High
138	Efpeglenatide_high_dosage:Sotagliflozin	0			0.184377236	⊕⊕○○ Low	0.184377236	⊕⊕○○ Low
139	Efpeglenatide_high_dosage:Tirzepatide_high_dosage	0			-0.389881197	⊕⊕○○ Low	-0.389881197	⊕⊕○○ Low
140	Efpeglenatide_high_dosage:Tirzepatide_low_dosage	0			0.44403019	⊕⊕○○ Low	0.44403019	⊕⊕○○ Low
141	Efpeglenatide_low_dosage:Efpeglenatide_medium_dosage	1	-1.098612289	⊕⊕⊕⊕ High			-1.098612289	⊕⊕⊕○ Medium
142	Efpeglenatide_low_dosage:Empagliflozin_high_dosage	0			-0.352098433	⊕⊕○○ Low	-0.352098433	⊕⊕○○ Low
143	Efpeglenatide_low_dosage:Empagliflozin_low_dosage	0			-0.532311697	⊕⊕○○ Low	-0.532311697	⊕⊕○○ Low
144	Efpeglenatide_low_dosage:Ertugliflozin_high_dosage	0			-0.545005923	⊕⊕○○ Low	-0.545005923	⊕⊕○○ Low
145	Efpeglenatide_low_dosage:Ertugliflozin_low_dosage	0			-0.911959538	⊕⊕○○ Low	-0.911959538	⊕⊕○○ Low
146	Efpeglenatide_low_dosage:Exenatide	0			-0.15850365	⊕⊕○○ Low	-0.15850365	⊕⊕○○ Low
147	Efpeglenatide_low_dosage:Inject_semaglutide_high_dosage	0			-0.569213448	⊕⊕○○ Low	-0.569213448	⊕⊕○○ Low
148	Efpeglenatide_low_dosage:Inject_semaglutide_low_dosage	0			-0.949038631	⊕⊕○○ Low	-0.949038631	⊕⊕○○ Low
149	Efpeglenatide_low_dosage:Inject_semaglutide_medium_dosage	0			-0.234915672	⊕⊕○○ Low	-0.234915672	⊕⊕○○ Low
150	Efpeglenatide_low_dosage:Liraglutide	0			-0.975485569	⊕⊕○○ Low	-0.975485569	⊕⊕○○ Low
151	Efpeglenatide_low_dosage:Lixisenatide	0			0.27800426	⊕⊕○○ Low	0.27800426	⊕⊕○○ Low

152	Efpeglenatide_low_dosage:Oral_semaglutide	0			-0.650281866	⊕⊕○○ Low	-0.650281866	⊕⊕○○ Low
153	Efpeglenatide_low_dosage:Placebo_or_Control	0			-0.837751306	⊕⊕○○ Low	-0.837751306	⊕⊕○○ Low
154	Efpeglenatide_low_dosage:Sotagliflozin	0			-0.912538043	⊕⊕○○ Low	-0.912538043	⊕⊕○○ Low
155	Efpeglenatide_low_dosage:Tirzepatide_high_dosage	0			-1.486796477	⊕⊕○○ Low	-1.486796477	⊕⊕○○ Low
156	Efpeglenatide_low_dosage:Tirzepatide_low_dosage	0			-0.652885089	⊕⊕○○ Low	-0.652885089	⊕⊕○○ Low
157	Efpeglenatide_medium_dosage:Empagliflozin_high_dosage	0			0.746513856	⊕⊕○○ Low	0.746513856	⊕⊕○○ Low
158	Efpeglenatide_medium_dosage:Empagliflozin_low_dosage	0			0.566300592	⊕⊕○○ Low	0.566300592	⊕⊕○○ Low
159	Efpeglenatide_medium_dosage:Ertugliflozin_high_dosage	0			0.553606366	⊕⊕○○ Low	0.553606366	⊕⊕○○ Low
160	Efpeglenatide_medium_dosage:Ertugliflozin_low_dosage	0			0.18665275	⊕⊕○○ Low	0.18665275	⊕⊕○○ Low
161	Efpeglenatide_medium_dosage:Exenatide	0			0.940108638	⊕⊕○○ Low	0.940108638	⊕⊕○○ Low
162	Efpeglenatide_medium_dosage:Inject_semaglutide_high_dosage	0			0.529398841	⊕⊕○○ Low	0.529398841	⊕⊕○○ Low
163	Efpeglenatide_medium_dosage:Inject_semaglutide_low_dosage	0			0.149573657	⊕⊕○○ Low	0.149573657	⊕⊕○○ Low
164	Efpeglenatide_medium_dosage:Inject_semaglutide_medium_dosage	0			0.863696617	⊕⊕○○ Low	0.863696617	⊕⊕○○ Low
165	Efpeglenatide_medium_dosage:Liraglutide	0			0.12312672	⊕⊕○○ Low	0.12312672	⊕⊕○○ Low
166	Efpeglenatide_medium_dosage:Lixisenatide	0			1.376616549	⊕⊕○○ Low	1.376616549	⊕⊕○○ Low
167	Efpeglenatide_medium_dosage:Oral_semaglutide	0			0.448330423	⊕⊕○○ Low	0.448330423	⊕⊕○○ Low
168	Efpeglenatide_medium_dosage:Placebo_or_Control	2	0.175455431	⊕⊕⊕⊕ High	1.162638845	⊕⊕⊕⊕ High	0.260860983	⊕⊕⊕⊕ High
169	Efpeglenatide_medium_dosage:Sotagliflozin	0			0.186074245	⊕⊕○○ Low	0.186074245	⊕⊕○○ Low
170	Efpeglenatide_medium_dosage:Tirzepatide_high_dosage	0			-0.388184188	⊕⊕○○ Low	-0.388184188	⊕⊕○○ Low
171	Efpeglenatide_medium_dosage:Tirzepatide_low_dosage	0			0.445727199	⊕⊕○○ Low	0.445727199	⊕⊕○○ Low
172	Empagliflozin_high_dosage:Empagliflozin_low_dosage	3	-0.251335304	⊕⊕⊕⊕ High	-0.006796508	⊕⊕⊕⊕ High	-0.180213264	⊕⊕⊕⊕ High
173	Empagliflozin_high_dosage:Ertugliflozin_high_dosage	0			-0.19290749	⊕⊕○○ Low	-0.19290749	⊕⊕○○ Low

174	Empagliflozin_high_dosage:Ertugliflozin_low_dosage	0			-0.559861105	⊕⊕○○ Low	-0.559861105	⊕⊕○○ Low
175	Empagliflozin_high_dosage:Exenatide	0			0.193594783	⊕⊕○○ Low	0.193594783	⊕⊕○○ Low
176	Empagliflozin_high_dosage:Inject_semaglutide_high_dosage	0			-0.217115014	⊕⊕○○ Low	-0.217115014	⊕⊕○○ Low
177	Empagliflozin_high_dosage:Inject_semaglutide_low_dosage	0			-0.596940198	⊕⊕○○ Low	-0.596940198	⊕⊕○○ Low
178	Empagliflozin_high_dosage:Inject_semaglutide_medium_dosage	0			0.117182761	⊕⊕○○ Low	0.117182761	⊕⊕○○ Low
179	Empagliflozin_high_dosage:Liraglutide	0			-0.623387135	⊕⊕○○ Low	-0.623387135	⊕⊕○○ Low
180	Empagliflozin_high_dosage:Lixisenatide	0			0.630102693	⊕⊕○○ Low	0.630102693	⊕⊕○○ Low
181	Empagliflozin_high_dosage:Oral_semaglutide	1	0.002447982	⊕⊕⊕⊕ High	-0.324194788	⊕⊕⊕○ Medium	-0.298183433	⊕⊕⊕⊕ High
182	Empagliflozin_high_dosage:Placebo_or_Control	4	-0.453233141	⊕⊕⊕⊕ High	-0.636720148	⊕⊕⊕⊕ High	-0.485652873	⊕⊕⊕⊕ High
183	Empagliflozin_high_dosage:Sotagliflozin	0			-0.56043961	⊕⊕○○ Low	-0.56043961	⊕⊕○○ Low
184	Empagliflozin_high_dosage:Tirzepatide_high_dosage	0			-1.134698044	⊕⊕○○ Low	-1.134698044	⊕⊕○○ Low
185	Empagliflozin_high_dosage:Tirzepatide_low_dosage	0			-0.300786656	⊕⊕○○ Low	-0.300786656	⊕⊕○○ Low
186	Empagliflozin_low_dosage:Ertugliflozin_high_dosage	0			-0.012694226	⊕⊕○○ Low	-0.012694226	⊕⊕○○ Low
187	Empagliflozin_low_dosage:Ertugliflozin_low_dosage	0			-0.379647841	⊕⊕○○ Low	-0.379647841	⊕⊕○○ Low
188	Empagliflozin_low_dosage:Exenatide	0			0.373808046	⊕⊕○○ Low	0.373808046	⊕⊕○○ Low
189	Empagliflozin_low_dosage:Inject_semaglutide_high_dosage	0			-0.036901751	⊕⊕○○ Low	-0.036901751	⊕⊕○○ Low
190	Empagliflozin_low_dosage:Inject_semaglutide_low_dosage	0			-0.416726935	⊕⊕○○ Low	-0.416726935	⊕⊕○○ Low
191	Empagliflozin_low_dosage:Inject_semaglutide_medium_dosage	0			0.297396025	⊕⊕○○ Low	0.297396025	⊕⊕○○ Low
192	Empagliflozin_low_dosage:Liraglutide	0			-0.443173872	⊕⊕○○ Low	-0.443173872	⊕⊕○○ Low
193	Empagliflozin_low_dosage:Lixisenatide	0			0.810315957	⊕⊕○○ Low	0.810315957	⊕⊕○○ Low
194	Empagliflozin_low_dosage:Oral_semaglutide	0			-0.117970169	⊕⊕○○ Low	-0.117970169	⊕⊕○○ Low
195	Empagliflozin_low_dosage:Placebo_or_Control	8	-0.305610642	⊕⊕⊕⊕ High	-0.249067448	⊕⊕⊕⊕ High	-0.305439609	⊕⊕⊕⊕ High

196	Empagliflozin_low_dosage:Sotagliflozin	0			-0.380226347	⊕⊕○○ Low	-0.380226347	⊕⊕○○ Low
197	Empagliflozin_low_dosage:Tirzepatide_high_dosage	0			-0.95448478	⊕⊕○○ Low	-0.95448478	⊕⊕○○ Low
198	Empagliflozin_low_dosage:Tirzepatide_low_dosage	0			-0.120573393	⊕⊕○○ Low	-0.120573393	⊕⊕○○ Low
199	Ertugliflozin_high_dosage:Ertugliflozin_low_dosage	2	-0.34146741	⊕⊕⊕⊕ High	-4.011763556	⊕⊕⊕⊕ High	-0.366953615	⊕⊕⊕⊕ High
200	Ertugliflozin_high_dosage:Exenatide	0			0.386502272	⊕⊕○○ Low	0.386502272	⊕⊕○○ Low
201	Ertugliflozin_high_dosage:Inject_semaglutide_high_dosage	0			-0.024207525	⊕⊕○○ Low	-0.024207525	⊕⊕○○ Low
202	Ertugliflozin_high_dosage:Inject_semaglutide_low_dosage	0			-0.404032709	⊕⊕○○ Low	-0.404032709	⊕⊕○○ Low
203	Ertugliflozin_high_dosage:Inject_semaglutide_medium_dosage	0			0.310090251	⊕⊕○○ Low	0.310090251	⊕⊕○○ Low
204	Ertugliflozin_high_dosage:Liraglutide	0			-0.430479646	⊕⊕○○ Low	-0.430479646	⊕⊕○○ Low
205	Ertugliflozin_high_dosage:Lixisenatide	0			0.823010183	⊕⊕○○ Low	0.823010183	⊕⊕○○ Low
206	Ertugliflozin_high_dosage:Oral_semaglutide	0			-0.105275943	⊕⊕○○ Low	-0.105275943	⊕⊕○○ Low
207	Ertugliflozin_high_dosage:Placebo_or_Control	1	-0.290617854	⊕⊕⊕⊕ High	-0.395595018	⊕⊕⊕⊕ High	-0.292745383	⊕⊕⊕⊕ High
208	Ertugliflozin_high_dosage:Sotagliflozin	0			-0.367532121	⊕⊕○○ Low	-0.367532121	⊕⊕○○ Low
209	Ertugliflozin_high_dosage:Tirzepatide_high_dosage	0			-0.941790554	⊕⊕○○ Low	-0.941790554	⊕⊕○○ Low
210	Ertugliflozin_high_dosage:Tirzepatide_low_dosage	0			-0.107879167	⊕⊕○○ Low	-0.107879167	⊕⊕○○ Low
211	Ertugliflozin_low_dosage:Exenatide	0			0.753455888	⊕⊕○○ Low	0.753455888	⊕⊕○○ Low
212	Ertugliflozin_low_dosage:Inject_semaglutide_high_dosage	0			0.342746091	⊕⊕○○ Low	0.342746091	⊕⊕○○ Low
213	Ertugliflozin_low_dosage:Inject_semaglutide_low_dosage	0			-0.037079093	⊕⊕○○ Low	-0.037079093	⊕⊕○○ Low
214	Ertugliflozin_low_dosage:Inject_semaglutide_medium_dosage	0			0.677043866	⊕⊕○○ Low	0.677043866	⊕⊕○○ Low
215	Ertugliflozin_low_dosage:Liraglutide	0			-0.06352603	⊕⊕○○ Low	-0.06352603	⊕⊕○○ Low
216	Ertugliflozin_low_dosage:Lixisenatide	0			1.189963798	⊕⊕○○ Low	1.189963798	⊕⊕○○ Low
217	Ertugliflozin_low_dosage:Oral_semaglutide	0			0.261677672	⊕⊕○○ Low	0.261677672	⊕⊕○○ Low

218	Ertugliflozin_low_dosage:Placebo_or_Control	2	0.053399449	⊕⊕⊕⊕ High	2.625191221	⊕⊕⊕⊕ High	0.074208233	⊕⊕⊕⊕ High
219	Ertugliflozin_low_dosage:Sotagliflozin	0			-0.000578505	⊕⊕○○ Low	-0.000578505	⊕⊕○○ Low
220	Ertugliflozin_low_dosage:Tirzepatide_high_dosage	0			-0.574836938	⊕⊕○○ Low	-0.574836938	⊕⊕○○ Low
221	Ertugliflozin_low_dosage:Tirzepatide_low_dosage	0			0.259074449	⊕⊕○○ Low	0.259074449	⊕⊕○○ Low
222	Exenatide:Inject_semaglutide_high_dosage	0			-0.410709797	⊕⊕○○ Low	-0.410709797	⊕⊕○○ Low
223	Exenatide:Inject_semaglutide_low_dosage	0			-0.790534981	⊕⊕○○ Low	-0.790534981	⊕⊕○○ Low
224	Exenatide:Inject_semaglutide_medium_dosage	0			-0.076412021	⊕⊕○○ Low	-0.076412021	⊕⊕○○ Low
225	Exenatide:Liraglutide	0			-0.816981918	⊕⊕○○ Low	-0.816981918	⊕⊕○○ Low
226	Exenatide:Lixisenatide	0			0.43650791	⊕⊕○○ Low	0.43650791	⊕⊕○○ Low
227	Exenatide:Oral_semaglutide	0			-0.491778216	⊕⊕○○ Low	-0.491778216	⊕⊕○○ Low
228	Exenatide:Placebo_or_Control	3	-0.679247655	⊕⊕⊕⊕ High			-0.679247655	⊕⊕⊕○ Medium
229	Exenatide:Sotagliflozin	0			-0.754034393	⊕⊕○○ Low	-0.754034393	⊕⊕○○ Low
230	Exenatide:Tirzepatide_high_dosage	0			-1.328292826	⊕⊕○○ Low	-1.328292826	⊕⊕○○ Low
231	Exenatide:Tirzepatide_low_dosage	0			-0.494381439	⊕⊕○○ Low	-0.494381439	⊕⊕○○ Low
232	Inject_semaglutide_high_dosage:Inject_semaglutide_low_dosage	0			-0.379825184	⊕⊕○○ Low	-0.379825184	⊕⊕○○ Low
233	Inject_semaglutide_high_dosage:Inject_semaglutide_medium_dosage	1	0.405465108	⊕⊕⊕⊕ High	0.321418418	⊕⊕⊕⊕ High	0.334297776	⊕⊕⊕⊕ High
234	Inject_semaglutide_high_dosage:Liraglutide	1	1.114422895	⊕⊕⊕⊕ High	-0.427086597	⊕⊕⊕○ Medium	-0.406272121	⊕⊕⊕⊕ High
235	Inject_semaglutide_high_dosage:Lixisenatide	0			0.847217708	⊕⊕○○ Low	0.847217708	⊕⊕○○ Low
236	Inject_semaglutide_high_dosage:Oral_semaglutide	0			-0.081068418	⊕⊕○○ Low	-0.081068418	⊕⊕○○ Low
237	Inject_semaglutide_high_dosage:Placebo_or_Control	4	-0.274385957	⊕⊕⊕⊕ High	0.026846936	⊕⊕⊕○ Medium	-0.268537858	⊕⊕⊕⊕ High
238	Inject_semaglutide_high_dosage:Sotagliflozin	0			-0.343324596	⊕⊕○○ Low	-0.343324596	⊕⊕○○ Low
239	Inject_semaglutide_high_dosage:Tirzepatide_high_dosage	0			-0.917583029	⊕⊕○○ Low	-0.917583029	⊕⊕○○ Low

240	Inject_semaglutide_high_dosage:Tirzepatide_low_dosage	0			-0.083671642	⊕⊕○○ Low	-0.083671642	⊕⊕○○ Low
241	Inject_semaglutide_low_dosage:Inject_semaglutide_medium_dosage	2	0.894447144	⊕⊕⊕⊕ High	-0.352050854	⊕⊕⊕○ Medium	0.71412296	⊕⊕⊕⊕ High
242	Inject_semaglutide_low_dosage:Liraglutide	1	-0.837589046	⊕⊕⊕⊕ High	-0.000119247	⊕⊕⊕⊕ High	-0.026446937	⊕⊕⊕⊕ High
243	Inject_semaglutide_low_dosage:Lixisenatide	0			1.227042891	⊕⊕○○ Low	1.227042891	⊕⊕○○ Low
244	Inject_semaglutide_low_dosage:Oral_semaglutide	0			0.298756765	⊕⊕○○ Low	0.298756765	⊕⊕○○ Low
245	Inject_semaglutide_low_dosage:Placebo_or_Control	2	0.129468118	⊕⊕⊕⊕ High	-0.220789345	⊕⊕⊕○ Medium	0.111287326	⊕⊕⊕⊕ High
246	Inject_semaglutide_low_dosage:Sotagliflozin	0			0.036500588	⊕⊕○○ Low	0.036500588	⊕⊕○○ Low
247	Inject_semaglutide_low_dosage:Tirzepatide_high_dosage	0			-0.537757845	⊕⊕○○ Low	-0.537757845	⊕⊕○○ Low
248	Inject_semaglutide_low_dosage:Tirzepatide_low_dosage	0			0.296153542	⊕⊕○○ Low	0.296153542	⊕⊕○○ Low
249	Inject_semaglutide_medium_dosage:Liraglutide	0			-0.740569897	⊕⊕○○ Low	-0.740569897	⊕⊕○○ Low
250	Inject_semaglutide_medium_dosage:Lixisenatide	0			0.512919932	⊕⊕○○ Low	0.512919932	⊕⊕○○ Low
251	Inject_semaglutide_medium_dosage:Oral_semaglutide	0			-0.415366194	⊕⊕○○ Low	-0.415366194	⊕⊕○○ Low
252	Inject_semaglutide_medium_dosage:Placebo_or_Control	3	-0.639786405	⊕⊕⊕⊕ High	-0.37840586	⊕⊕⊕⊕ High	-0.602835634	⊕⊕⊕⊕ High
253	Inject_semaglutide_medium_dosage:Sotagliflozin	0			-0.677622372	⊕⊕○○ Low	-0.677622372	⊕⊕○○ Low
254	Inject_semaglutide_medium_dosage:Tirzepatide_high_dosage	1	-0.405997731	⊕⊕⊕⊕ High	-1.408861007	⊕⊕⊕⊕ High	-1.251880805	⊕⊕⊕⊕ High
255	Inject_semaglutide_medium_dosage:Tirzepatide_low_dosage	0			-0.417969418	⊕⊕○○ Low	-0.417969418	⊕⊕○○ Low
256	Liraglutide:Lixisenatide	0			1.253489828	⊕⊕○○ Low	1.253489828	⊕⊕○○ Low
257	Liraglutide:Oral_semaglutide	0			0.325203702	⊕⊕○○ Low	0.325203702	⊕⊕○○ Low
258	Liraglutide:Placebo_or_Control	3	0.145104586	⊕⊕⊕⊕ High	-0.147294081	⊕⊕⊕○ Medium	0.137734263	⊕⊕⊕⊕ High
259	Liraglutide:Sotagliflozin	0			0.062947525	⊕⊕○○ Low	0.062947525	⊕⊕○○ Low
260	Liraglutide:Tirzepatide_high_dosage	0			-0.511310908	⊕⊕○○ Low	-0.511310908	⊕⊕○○ Low
261	Liraglutide:Tirzepatide_low_dosage	0			0.322600479	⊕⊕○○ Low	0.322600479	⊕⊕○○ Low

262	Lixisenatide:Oral_semaglutide	0			-0.928286126	⊕⊕○○ Low	-0.928286126	⊕⊕○○ Low
263	Lixisenatide:Placebo_or_Control	1	-1.115755566	⊕⊕⊕⊕ High			-1.115755566	⊕⊕⊕○ Medium
264	Lixisenatide:Sotagliflozin	0			-1.190542303	⊕⊕○○ Low	-1.190542303	⊕⊕○○ Low
265	Lixisenatide:Tirzepatide_high_dosage	0			-1.764800737	⊕⊕○○ Low	-1.764800737	⊕⊕○○ Low
266	Lixisenatide:Tirzepatide_low_dosage	0			-0.930889349	⊕⊕○○ Low	-0.930889349	⊕⊕○○ Low
267	Oral_semaglutide:Placebo_or_Control	3	-0.170319447	⊕⊕⊕⊕ High	-0.496962216	⊕⊕⊕⊕ High	-0.18746944	⊕⊕⊕⊕ High
268	Oral_semaglutide:Sotagliflozin	0			-0.262256177	⊕⊕○○ Low	-0.262256177	⊕⊕○○ Low
269	Oral_semaglutide:Tirzepatide_high_dosage	0			-0.836514611	⊕⊕○○ Low	-0.836514611	⊕⊕○○ Low
270	Oral_semaglutide:Tirzepatide_low_dosage	0			-0.002603223	⊕⊕○○ Low	-0.002603223	⊕⊕○○ Low
271	Sotagliflozin:Placebo_or_Control	4	0.074786738	⊕⊕⊕⊕ High			0.074786738	⊕⊕⊕○ Medium
272	Tirzepatide_high_dosage:Placebo_or_Control	5	0.821141732	⊕⊕⊕⊕ High	0.108633877	⊕⊕⊕⊕ High	0.649045171	⊕⊕⊕⊕ High
273	Tirzepatide_low_dosage:Placebo_or_Control	0			-0.184866216	⊕⊕○○ Low	-0.184866216	⊕⊕○○ Low
274	Sotagliflozin:Tirzepatide_high_dosage	0			-0.574258433	⊕⊕○○ Low	-0.574258433	⊕⊕○○ Low
275	Sotagliflozin:Tirzepatide_low_dosage	0			0.259652954	⊕⊕○○ Low	0.259652954	⊕⊕○○ Low
276	Tirzepatide_high_dosage:Tirzepatide_low_dosage	3	0.833911387	⊕⊕⊕⊕ High			0.833911387	⊕⊕⊕○ Medium

Table S8B: GRADE of primary outcome: acute kidney injury/acute renal failure in the subgroup focusing RCTs without definite underlying kidney failure

	Comparison	No.Studies	Direct		Indirect		NMA	
			Estimate	Rate	Estimate	Rate	Estimate	Rate
1	Albiglutide:Bexagliflozin	0			-0.342749324	⊕⊕○○ Low	-0.342749324	⊕⊕○○ Low
2	Albiglutide:Canagliflozin_high_dosage	0			0.556993672	⊕⊕○○ Low	0.556993672	⊕⊕○○ Low
3	Albiglutide:Canagliflozin_low_dosage	0			-0.095269752	⊕⊕○○ Low	-0.095269752	⊕⊕○○ Low
4	Albiglutide:Dapagliflozin	0			0.281598725	⊕⊕○○ Low	0.281598725	⊕⊕○○ Low
5	Albiglutide:Dulaglutide	0			-0.072796897	⊕⊕○○ Low	-0.072796897	⊕⊕○○ Low
6	Albiglutide:Efpeglenatide_high_dosage	0			-0.373314545	⊕⊕○○ Low	-0.373314545	⊕⊕○○ Low
7	Albiglutide:Efpeglenatide_low_dosage	0			0.723589592	⊕⊕○○ Low	0.723589592	⊕⊕○○ Low
8	Albiglutide:Efpeglenatide_medium_dosage	0			-0.375022697	⊕⊕○○ Low	-0.375022697	⊕⊕○○ Low
9	Albiglutide:Empagliflozin_high_dosage	0			0.444907415	⊕⊕○○ Low	0.444907415	⊕⊕○○ Low
10	Albiglutide:Empagliflozin_low_dosage	0			0.228460534	⊕⊕○○ Low	0.228460534	⊕⊕○○ Low
11	Albiglutide:Ertugliflozin_high_dosage	0			0.174570804	⊕⊕○○ Low	0.174570804	⊕⊕○○ Low
12	Albiglutide:Ertugliflozin_low_dosage	0			-0.115679606	⊕⊕○○ Low	-0.115679606	⊕⊕○○ Low
13	Albiglutide:Exenatide	0			0.563200605	⊕⊕○○ Low	0.563200605	⊕⊕○○ Low
14	Albiglutide:Inject_semaglutide_high_dosage	0			0.152495401	⊕⊕○○ Low	0.152495401	⊕⊕○○ Low
15	Albiglutide:Inject_semaglutide_low_dosage	0			-0.227298843	⊕⊕○○ Low	-0.227298843	⊕⊕○○ Low
16	Albiglutide:Inject_semaglutide_medium_dosage	0			0.486952909	⊕⊕○○ Low	0.486952909	⊕⊕○○ Low
17	Albiglutide:Liraglutide	1	1.110927715	⊕⊕⊕⊕ High	-0.283093008	⊕⊕⊕○ Medium	-0.25358143	⊕⊕⊕⊕ High
18	Albiglutide:Lixisenatide	0			0.999708515	⊕⊕○○ Low	0.999708515	⊕⊕○○ Low

19	Albiglutide:Oral_semaglutide	0			-0.343111651	⊕⊕○○ Low	-0.343111651	⊕⊕○○ Low
20	Albiglutide:Placebo_or_Control	2	-0.136763693	⊕⊕⊕⊕ High	1.25725703	⊕⊕⊕○ Medium	-0.11604705	⊕⊕⊕⊕ High
21	Albiglutide:Sotagliflozin	0			0.212519783	⊕⊕○○ Low	0.212519783	⊕⊕○○ Low
22	Albiglutide:Tirzepatide_high_dosage	0			-0.761246213	⊕⊕○○ Low	-0.761246213	⊕⊕○○ Low
23	Albiglutide:Tirzepatide_low_dosage	0			0.072665174	⊕⊕○○ Low	0.072665174	⊕⊕○○ Low
24	Bexagliflozin:Canagliflozin_high_dosage	0			0.899742997	⊕⊕○○ Low	0.899742997	⊕⊕○○ Low
25	Bexagliflozin:Canagliflozin_low_dosage	0			0.247479573	⊕⊕○○ Low	0.247479573	⊕⊕○○ Low
26	Bexagliflozin:Dapagliflozin	0			0.624348049	⊕⊕○○ Low	0.624348049	⊕⊕○○ Low
27	Bexagliflozin:Dulaglutide	0			0.269952428	⊕⊕○○ Low	0.269952428	⊕⊕○○ Low
28	Bexagliflozin:Efpeglenatide_high_dosage	0			-0.030565221	⊕⊕○○ Low	-0.030565221	⊕⊕○○ Low
29	Bexagliflozin:Efpeglenatide_low_dosage	0			1.066338916	⊕⊕○○ Low	1.066338916	⊕⊕○○ Low
30	Bexagliflozin:Efpeglenatide_medium_dosage	0			-0.032273372	⊕⊕○○ Low	-0.032273372	⊕⊕○○ Low
31	Bexagliflozin:Empagliflozin_high_dosage	0			0.787656739	⊕⊕○○ Low	0.787656739	⊕⊕○○ Low
32	Bexagliflozin:Empagliflozin_low_dosage	0			0.571209858	⊕⊕○○ Low	0.571209858	⊕⊕○○ Low
33	Bexagliflozin:Ertugliflozin_high_dosage	0			0.517320128	⊕⊕○○ Low	0.517320128	⊕⊕○○ Low
34	Bexagliflozin:Ertugliflozin_low_dosage	0			0.227069718	⊕⊕○○ Low	0.227069718	⊕⊕○○ Low
35	Bexagliflozin:Exenatide	0			0.905949929	⊕⊕○○ Low	0.905949929	⊕⊕○○ Low
36	Bexagliflozin:Inject_semaglutide_high_dosage	0			0.495244725	⊕⊕○○ Low	0.495244725	⊕⊕○○ Low
37	Bexagliflozin:Inject_semaglutide_low_dosage	0			0.115450482	⊕⊕○○ Low	0.115450482	⊕⊕○○ Low
38	Bexagliflozin:Inject_semaglutide_medium_dosage	0			0.829702233	⊕⊕○○ Low	0.829702233	⊕⊕○○ Low
39	Bexagliflozin:Liraglutide	0			0.089167894	⊕⊕○○ Low	0.089167894	⊕⊕○○ Low
40	Bexagliflozin:Lixisenatide	0			1.34245784	⊕⊕○○ Low	1.34245784	⊕⊕○○ Low

41	Bexagliflozin:Oral_semaglutide	0			-0.000362327	⊕⊕○○ Low	-0.000362327	⊕⊕○○ Low
42	Bexagliflozin:Placebo_or_Control	1	0.226702274	⊕⊕⊕⊕ High			0.226702274	⊕⊕⊕○ Medium
43	Bexagliflozin:Sotagliflozin	0			0.555269107	⊕⊕○○ Low	0.555269107	⊕⊕○○ Low
44	Bexagliflozin:Tirzepatide_high_dosage	0			-0.418496889	⊕⊕○○ Low	-0.418496889	⊕⊕○○ Low
45	Bexagliflozin:Tirzepatide_low_dosage	0			0.415414499	⊕⊕○○ Low	0.415414499	⊕⊕○○ Low
46	Canagliflozin_high_dosage:Canagliflozin_low_dosage	1	-0.630704364	⊕⊕⊕⊕ High	-0.722319775	⊕⊕⊕⊕ High	-0.652263424	⊕⊕⊕⊕ High
47	Canagliflozin_high_dosage:Dapagliflozin	0			-0.275394948	⊕⊕○○ Low	-0.275394948	⊕⊕○○ Low
48	Canagliflozin_high_dosage:Dulaglutide	0			-0.629790569	⊕⊕○○ Low	-0.629790569	⊕⊕○○ Low
49	Canagliflozin_high_dosage:Efpeglenatide_high_dosage	0			-0.930308217	⊕⊕○○ Low	-0.930308217	⊕⊕○○ Low
50	Canagliflozin_high_dosage:Efpeglenatide_low_dosage	0			0.16659592	⊕⊕○○ Low	0.16659592	⊕⊕○○ Low
51	Canagliflozin_high_dosage:Efpeglenatide_medium_dosage	0			-0.932016369	⊕⊕○○ Low	-0.932016369	⊕⊕○○ Low
52	Canagliflozin_high_dosage:Empagliflozin_high_dosage	0			-0.112086258	⊕⊕○○ Low	-0.112086258	⊕⊕○○ Low
53	Canagliflozin_high_dosage:Empagliflozin_low_dosage	0			-0.328533139	⊕⊕○○ Low	-0.328533139	⊕⊕○○ Low
54	Canagliflozin_high_dosage:Ertugliflozin_high_dosage	0			-0.382422868	⊕⊕○○ Low	-0.382422868	⊕⊕○○ Low
55	Canagliflozin_high_dosage:Ertugliflozin_low_dosage	0			-0.672673278	⊕⊕○○ Low	-0.672673278	⊕⊕○○ Low
56	Canagliflozin_high_dosage:Exenatide	0			0.006206933	⊕⊕○○ Low	0.006206933	⊕⊕○○ Low
57	Canagliflozin_high_dosage:Inject_semaglutide_high_dosage	0			-0.404498271	⊕⊕○○ Low	-0.404498271	⊕⊕○○ Low
58	Canagliflozin_high_dosage:Inject_semaglutide_low_dosage	0			-0.784292515	⊕⊕○○ Low	-0.784292515	⊕⊕○○ Low
59	Canagliflozin_high_dosage:Inject_semaglutide_medium_dosage	0			-0.070040763	⊕⊕○○ Low	-0.070040763	⊕⊕○○ Low
60	Canagliflozin_high_dosage:Liraglutide	0			-0.810575102	⊕⊕○○ Low	-0.810575102	⊕⊕○○ Low
61	Canagliflozin_high_dosage:Lixisenatide	0			0.442714843	⊕⊕○○ Low	0.442714843	⊕⊕○○ Low
62	Canagliflozin_high_dosage:Oral_semaglutide	0			-0.900105323	⊕⊕○○ Low	-0.900105323	⊕⊕○○ Low

63	Canagliflozin_high_dosage:Placebo_or_Control	3	-0.642671132	⊕⊕⊕⊕ High	-3.713985129	⊕⊕⊕⊕ High	-0.673040723	⊕⊕⊕⊕ High
64	Canagliflozin_high_dosage:Sotagliflozin	0			-0.344473889	⊕⊕○○ Low	-0.344473889	⊕⊕○○ Low
65	Canagliflozin_high_dosage:Tirzepatide_high_dosage	0			-1.318239885	⊕⊕○○ Low	-1.318239885	⊕⊕○○ Low
66	Canagliflozin_high_dosage:Tirzepatide_low_dosage	0			-0.484328498	⊕⊕○○ Low	-0.484328498	⊕⊕○○ Low
67	Canagliflozin_low_dosage:Dapagliflozin	0			0.376868476	⊕⊕○○ Low	0.376868476	⊕⊕○○ Low
68	Canagliflozin_low_dosage:Dulaglutide	0			0.022472855	⊕⊕○○ Low	0.022472855	⊕⊕○○ Low
69	Canagliflozin_low_dosage:Efpeglenatide_high_dosage	0			-0.278044793	⊕⊕○○ Low	-0.278044793	⊕⊕○○ Low
70	Canagliflozin_low_dosage:Efpeglenatide_low_dosage	0			0.818859344	⊕⊕○○ Low	0.818859344	⊕⊕○○ Low
71	Canagliflozin_low_dosage:Efpeglenatide_medium_dosage	0			-0.279752945	⊕⊕○○ Low	-0.279752945	⊕⊕○○ Low
72	Canagliflozin_low_dosage:Empagliflozin_high_dosage	0			0.540177166	⊕⊕○○ Low	0.540177166	⊕⊕○○ Low
73	Canagliflozin_low_dosage:Empagliflozin_low_dosage	0			0.323730285	⊕⊕○○ Low	0.323730285	⊕⊕○○ Low
74	Canagliflozin_low_dosage:Ertugliflozin_high_dosage	0			0.269840556	⊕⊕○○ Low	0.269840556	⊕⊕○○ Low
75	Canagliflozin_low_dosage:Ertugliflozin_low_dosage	0			-0.020409854	⊕⊕○○ Low	-0.020409854	⊕⊕○○ Low
76	Canagliflozin_low_dosage:Exenatide	0			0.658470357	⊕⊕○○ Low	0.658470357	⊕⊕○○ Low
77	Canagliflozin_low_dosage:Inject_semaglutide_high_dosage	0			0.247765153	⊕⊕○○ Low	0.247765153	⊕⊕○○ Low
78	Canagliflozin_low_dosage:Inject_semaglutide_low_dosage	0			-0.132029091	⊕⊕○○ Low	-0.132029091	⊕⊕○○ Low
79	Canagliflozin_low_dosage:Inject_semaglutide_medium_dosage	0			0.582222661	⊕⊕○○ Low	0.582222661	⊕⊕○○ Low
80	Canagliflozin_low_dosage:Liraglutide	0			-0.158311678	⊕⊕○○ Low	-0.158311678	⊕⊕○○ Low
81	Canagliflozin_low_dosage:Lixisenatide	0			1.094978267	⊕⊕○○ Low	1.094978267	⊕⊕○○ Low
82	Canagliflozin_low_dosage:Oral_semaglutide	0			-0.247841899	⊕⊕○○ Low	-0.247841899	⊕⊕○○ Low
83	Canagliflozin_low_dosage:Placebo_or_Control	3	0.021236715	⊕⊕⊕⊕ High	-0.420447399	⊕⊕⊕○ Medium	-0.020777299	⊕⊕⊕⊕ High
84	Canagliflozin_low_dosage:Sotagliflozin	0			0.307789535	⊕⊕○○ Low	0.307789535	⊕⊕○○ Low

85	Canagliflozin_low_dosage:Tirzepatide_high_dosage	0			-0.665976461	⊕⊕○○ Low	-0.665976461	⊕⊕○○ Low
86	Canagliflozin_low_dosage:Tirzepatide_low_dosage	0			0.167934926	⊕⊕○○ Low	0.167934926	⊕⊕○○ Low
87	Dapagliflozin:Dulaglutide	0			-0.354395621	⊕⊕○○ Low	-0.354395621	⊕⊕○○ Low
88	Dapagliflozin:Efpeglenatide_high_dosage	0			-0.654913269	⊕⊕○○ Low	-0.654913269	⊕⊕○○ Low
89	Dapagliflozin:Efpeglenatide_low_dosage	0			0.441990867	⊕⊕○○ Low	0.441990867	⊕⊕○○ Low
90	Dapagliflozin:Efpeglenatide_medium_dosage	0			-0.656621421	⊕⊕○○ Low	-0.656621421	⊕⊕○○ Low
91	Dapagliflozin:Empagliflozin_high_dosage	0			0.16330869	⊕⊕○○ Low	0.16330869	⊕⊕○○ Low
92	Dapagliflozin:Empagliflozin_low_dosage	0			-0.053138191	⊕⊕○○ Low	-0.053138191	⊕⊕○○ Low
93	Dapagliflozin:Ertugliflozin_high_dosage	0			-0.10702792	⊕⊕○○ Low	-0.10702792	⊕⊕○○ Low
94	Dapagliflozin:Ertugliflozin_low_dosage	0			-0.39727833	⊕⊕○○ Low	-0.39727833	⊕⊕○○ Low
95	Dapagliflozin:Exenatide	0			0.28160188	⊕⊕○○ Low	0.28160188	⊕⊕○○ Low
96	Dapagliflozin:Inject_semaglutide_high_dosage	0			-0.129103324	⊕⊕○○ Low	-0.129103324	⊕⊕○○ Low
97	Dapagliflozin:Inject_semaglutide_low_dosage	0			-0.508897567	⊕⊕○○ Low	-0.508897567	⊕⊕○○ Low
98	Dapagliflozin:Inject_semaglutide_medium_dosage	0			0.205354184	⊕⊕○○ Low	0.205354184	⊕⊕○○ Low
99	Dapagliflozin:Liraglutide	0			-0.535180155	⊕⊕○○ Low	-0.535180155	⊕⊕○○ Low
100	Dapagliflozin:Lixisenatide	0			0.718109791	⊕⊕○○ Low	0.718109791	⊕⊕○○ Low
101	Dapagliflozin:Oral_semaglutide	0			-0.624710375	⊕⊕○○ Low	-0.624710375	⊕⊕○○ Low
102	Dapagliflozin:Placebo_or_Control	8	-0.397645775	⊕⊕⊕⊕ High			-0.397645775	⊕⊕⊕○ Medium
103	Dapagliflozin:Sotagliflozin	0			-0.069078942	⊕⊕○○ Low	-0.069078942	⊕⊕○○ Low
104	Dapagliflozin:Tirzepatide_high_dosage	0			-1.042844937	⊕⊕○○ Low	-1.042844937	⊕⊕○○ Low
105	Dapagliflozin:Tirzepatide_low_dosage	0			-0.20893355	⊕⊕○○ Low	-0.20893355	⊕⊕○○ Low
106	Dulaglutide:Efpeglenatide_high_dosage	1	-1.13565356	⊕⊕⊕⊕ High	-0.231024746	⊕⊕⊕⊕ High	-0.300517648	⊕⊕⊕⊕ High

107	Dulaglutide:Efpeglenatide_low_dosage	0			0.796386489	⊕⊕○○ Low	0.796386489	⊕⊕○○ Low
108	Dulaglutide:Efpeglenatide_medium_dosage	1	-1.066089097	⊕⊕⊕⊕ High	-0.239220246	⊕⊕⊕⊕ High	-0.3022258	⊕⊕⊕⊕ High
109	Dulaglutide:Empagliflozin_high_dosage	0			0.517704311	⊕⊕○○ Low	0.517704311	⊕⊕○○ Low
110	Dulaglutide:Empagliflozin_low_dosage	0			0.30125743	⊕⊕○○ Low	0.30125743	⊕⊕○○ Low
111	Dulaglutide:Ertugliflozin_high_dosage	0			0.247367701	⊕⊕○○ Low	0.247367701	⊕⊕○○ Low
112	Dulaglutide:Ertugliflozin_low_dosage	0			-0.042882709	⊕⊕○○ Low	-0.042882709	⊕⊕○○ Low
113	Dulaglutide:Exenatide	0			0.635997502	⊕⊕○○ Low	0.635997502	⊕⊕○○ Low
114	Dulaglutide:Inject_semaglutide_high_dosage	0			0.225292298	⊕⊕○○ Low	0.225292298	⊕⊕○○ Low
115	Dulaglutide:Inject_semaglutide_low_dosage	0			-0.154501946	⊕⊕○○ Low	-0.154501946	⊕⊕○○ Low
116	Dulaglutide:Inject_semaglutide_medium_dosage	0			0.559749805	⊕⊕○○ Low	0.559749805	⊕⊕○○ Low
117	Dulaglutide:Liraglutide	1	-1.098612289	⊕⊕⊕⊕ High	-0.165512944	⊕⊕⊕⊕ High	-0.180784534	⊕⊕⊕⊕ High
118	Dulaglutide:Lixisenatide	0			1.072505412	⊕⊕○○ Low	1.072505412	⊕⊕○○ Low
119	Dulaglutide:Oral_semaglutide	0			-0.270314754	⊕⊕○○ Low	-0.270314754	⊕⊕○○ Low
120	Dulaglutide:Placebo_or_Control	1	-0.026578125	⊕⊕⊕⊕ High	-0.588807898	⊕⊕⊕⊕ High	-0.043250154	⊕⊕⊕⊕ High
121	Dulaglutide:Sotagliflozin	0			0.28531668	⊕⊕○○ Low	0.28531668	⊕⊕○○ Low
122	Dulaglutide:Tirzepatide_high_dosage	1	-0.457241378	⊕⊕⊕⊕ High	-0.723294853	⊕⊕⊕⊕ High	-0.688449316	⊕⊕⊕⊕ High
123	Dulaglutide:Tirzepatide_low_dosage	0			0.145462071	⊕⊕○○ Low	0.145462071	⊕⊕○○ Low
124	Efpeglenatide_high_dosage:Efpeglenatide_low_dosage	0			1.096904137	⊕⊕○○ Low	1.096904137	⊕⊕○○ Low
125	Efpeglenatide_high_dosage:Efpeglenatide_medium_dosage	3	0.025488071	⊕⊕⊕⊕ High	-1.784547432	⊕⊕⊕○ Medium	-0.001708152	⊕⊕⊕⊕ High
126	Efpeglenatide_high_dosage:Empagliflozin_high_dosage	0			0.818221959	⊕⊕○○ Low	0.818221959	⊕⊕○○ Low
127	Efpeglenatide_high_dosage:Empagliflozin_low_dosage	0			0.601775078	⊕⊕○○ Low	0.601775078	⊕⊕○○ Low
128	Efpeglenatide_high_dosage:Ertugliflozin_high_dosage	0			0.547885349	⊕⊕○○ Low	0.547885349	⊕⊕○○ Low

129	Efpeglenatide_high_dosage:Ertugliflozin_low_dosage	0			0.257634939	⊕⊕○○ Low	0.257634939	⊕⊕○○ Low
130	Efpeglenatide_high_dosage:Exenatide	0			0.93651515	⊕⊕○○ Low	0.93651515	⊕⊕○○ Low
131	Efpeglenatide_high_dosage:Inject_semaglutide_high_dosage	0			0.525809946	⊕⊕○○ Low	0.525809946	⊕⊕○○ Low
132	Efpeglenatide_high_dosage:Inject_semaglutide_low_dosage	0			0.146015702	⊕⊕○○ Low	0.146015702	⊕⊕○○ Low
133	Efpeglenatide_high_dosage:Inject_semaglutide_medium_dosage	0			0.860267454	⊕⊕○○ Low	0.860267454	⊕⊕○○ Low
134	Efpeglenatide_high_dosage:Liraglutide	0			0.119733115	⊕⊕○○ Low	0.119733115	⊕⊕○○ Low
135	Efpeglenatide_high_dosage:Lixisenatide	0			1.37302306	⊕⊕○○ Low	1.37302306	⊕⊕○○ Low
136	Efpeglenatide_high_dosage:Oral_semaglutide	0			0.030202894	⊕⊕○○ Low	0.030202894	⊕⊕○○ Low
137	Efpeglenatide_high_dosage:Placebo_or_Control	1	0.199928029	⊕⊕⊕⊕ High	0.734130172	⊕⊕⊕⊕ High	0.257267495	⊕⊕⊕⊕ High
138	Efpeglenatide_high_dosage:Sotagliflozin	0			0.585834328	⊕⊕○○ Low	0.585834328	⊕⊕○○ Low
139	Efpeglenatide_high_dosage:Tirzepatide_high_dosage	0			-0.387931668	⊕⊕○○ Low	-0.387931668	⊕⊕○○ Low
140	Efpeglenatide_high_dosage:Tirzepatide_low_dosage	0			0.445979719	⊕⊕○○ Low	0.445979719	⊕⊕○○ Low
141	Efpeglenatide_low_dosage:Efpeglenatide_medium_dosage	1	-1.098612289	⊕⊕⊕⊕ High			-1.098612289	⊕⊕⊕○ Medium
142	Efpeglenatide_low_dosage:Empagliflozin_high_dosage	0			-0.278682177	⊕⊕○○ Low	-0.278682177	⊕⊕○○ Low
143	Efpeglenatide_low_dosage:Empagliflozin_low_dosage	0			-0.495129058	⊕⊕○○ Low	-0.495129058	⊕⊕○○ Low
144	Efpeglenatide_low_dosage:Ertugliflozin_high_dosage	0			-0.549018788	⊕⊕○○ Low	-0.549018788	⊕⊕○○ Low
145	Efpeglenatide_low_dosage:Ertugliflozin_low_dosage	0			-0.839269198	⊕⊕○○ Low	-0.839269198	⊕⊕○○ Low
146	Efpeglenatide_low_dosage:Exenatide	0			-0.160388987	⊕⊕○○ Low	-0.160388987	⊕⊕○○ Low
147	Efpeglenatide_low_dosage:Inject_semaglutide_high_dosage	0			-0.571094191	⊕⊕○○ Low	-0.571094191	⊕⊕○○ Low
148	Efpeglenatide_low_dosage:Inject_semaglutide_low_dosage	0			-0.950888435	⊕⊕○○ Low	-0.950888435	⊕⊕○○ Low
149	Efpeglenatide_low_dosage:Inject_semaglutide_medium_dosage	0			-0.236636683	⊕⊕○○ Low	-0.236636683	⊕⊕○○ Low
150	Efpeglenatide_low_dosage:Liraglutide	0			-0.977171022	⊕⊕○○ Low	-0.977171022	⊕⊕○○ Low

151	Efpeglenatide_low_dosage:Lixisenatide	0			0.276118923	⊕⊕○○ Low	0.276118923	⊕⊕○○ Low
152	Efpeglenatide_low_dosage:Oral_semaglutide	0			-1.066701243	⊕⊕○○ Low	-1.066701243	⊕⊕○○ Low
153	Efpeglenatide_low_dosage:Placebo_or_Control	0			-0.839636642	⊕⊕○○ Low	-0.839636642	⊕⊕○○ Low
154	Efpeglenatide_low_dosage:Sotagliflozin	0			-0.511069809	⊕⊕○○ Low	-0.511069809	⊕⊕○○ Low
155	Efpeglenatide_low_dosage:Tirzepatide_high_dosage	0			-1.484835805	⊕⊕○○ Low	-1.484835805	⊕⊕○○ Low
156	Efpeglenatide_low_dosage:Tirzepatide_low_dosage	0			-0.650924418	⊕⊕○○ Low	-0.650924418	⊕⊕○○ Low
157	Efpeglenatide_medium_dosage:Empagliflozin_high_dosage	0			0.819930111	⊕⊕○○ Low	0.819930111	⊕⊕○○ Low
158	Efpeglenatide_medium_dosage:Empagliflozin_low_dosage	0			0.60348323	⊕⊕○○ Low	0.60348323	⊕⊕○○ Low
159	Efpeglenatide_medium_dosage:Ertugliflozin_high_dosage	0			0.549593501	⊕⊕○○ Low	0.549593501	⊕⊕○○ Low
160	Efpeglenatide_medium_dosage:Ertugliflozin_low_dosage	0			0.259343091	⊕⊕○○ Low	0.259343091	⊕⊕○○ Low
161	Efpeglenatide_medium_dosage:Exenatide	0			0.938223302	⊕⊕○○ Low	0.938223302	⊕⊕○○ Low
162	Efpeglenatide_medium_dosage:Inject_semaglutide_high_dosage	0			0.527518098	⊕⊕○○ Low	0.527518098	⊕⊕○○ Low
163	Efpeglenatide_medium_dosage:Inject_semaglutide_low_dosage	0			0.147723854	⊕⊕○○ Low	0.147723854	⊕⊕○○ Low
164	Efpeglenatide_medium_dosage:Inject_semaglutide_medium_dosage	0			0.861975606	⊕⊕○○ Low	0.861975606	⊕⊕○○ Low
165	Efpeglenatide_medium_dosage:Liraglutide	0			0.121441267	⊕⊕○○ Low	0.121441267	⊕⊕○○ Low
166	Efpeglenatide_medium_dosage:Lixisenatide	0			1.374731212	⊕⊕○○ Low	1.374731212	⊕⊕○○ Low
167	Efpeglenatide_medium_dosage:Oral_semaglutide	0			0.031911046	⊕⊕○○ Low	0.031911046	⊕⊕○○ Low
168	Efpeglenatide_medium_dosage:Placebo_or_Control	2	0.175455431	⊕⊕⊕⊕ High	1.141075057	⊕⊕⊕⊕ High	0.258975646	⊕⊕⊕⊕ High
169	Efpeglenatide_medium_dosage:Sotagliflozin	0			0.58754248	⊕⊕○○ Low	0.58754248	⊕⊕○○ Low
170	Efpeglenatide_medium_dosage:Tirzepatide_high_dosage	0			-0.386223516	⊕⊕○○ Low	-0.386223516	⊕⊕○○ Low
171	Efpeglenatide_medium_dosage:Tirzepatide_low_dosage	0			0.447687871	⊕⊕○○ Low	0.447687871	⊕⊕○○ Low
172	Empagliflozin_high_dosage:Empagliflozin_low_dosage	1	-0.315380954	⊕⊕⊕⊕ High	0.164354558	⊕⊕⊕○ Medium	-0.216446881	⊕⊕⊕⊕ High

173	Empagliflozin_high_dosage:Ertugliflozin_high_dosage	0			-0.270336611	⊕⊕○○ Low	-0.270336611	⊕⊕○○ Low
174	Empagliflozin_high_dosage:Ertugliflozin_low_dosage	0			-0.56058702	⊕⊕○○ Low	-0.56058702	⊕⊕○○ Low
175	Empagliflozin_high_dosage:Exenatide	0			0.11829319	⊕⊕○○ Low	0.11829319	⊕⊕○○ Low
176	Empagliflozin_high_dosage:Inject_semaglutide_high_dosage	0			-0.292412014	⊕⊕○○ Low	-0.292412014	⊕⊕○○ Low
177	Empagliflozin_high_dosage:Inject_semaglutide_low_dosage	0			-0.672206257	⊕⊕○○ Low	-0.672206257	⊕⊕○○ Low
178	Empagliflozin_high_dosage:Inject_semaglutide_medium_dosage	0			0.042045494	⊕⊕○○ Low	0.042045494	⊕⊕○○ Low
179	Empagliflozin_high_dosage:Liraglutide	0			-0.698488845	⊕⊕○○ Low	-0.698488845	⊕⊕○○ Low
180	Empagliflozin_high_dosage:Lixisenatide	0			0.554801101	⊕⊕○○ Low	0.554801101	⊕⊕○○ Low
181	Empagliflozin_high_dosage:Oral_semaglutide	1	0.002447982	⊕⊕⊕⊕ High	-1.694028529	⊕⊕⊕○ Medium	-0.788019065	⊕⊕⊕⊕ High
182	Empagliflozin_high_dosage:Placebo_or_Control	1	-0.530812566	⊕⊕⊕⊕ High	-0.725600426	⊕⊕⊕⊕ High	-0.560954465	⊕⊕⊕⊕ High
183	Empagliflozin_high_dosage:Sotagliflozin	0			-0.232387632	⊕⊕○○ Low	-0.232387632	⊕⊕○○ Low
184	Empagliflozin_high_dosage:Tirzepatide_high_dosage	0			-1.206153627	⊕⊕○○ Low	-1.206153627	⊕⊕○○ Low
185	Empagliflozin_high_dosage:Tirzepatide_low_dosage	0			-0.37224224	⊕⊕○○ Low	-0.37224224	⊕⊕○○ Low
186	Empagliflozin_low_dosage:Ertugliflozin_high_dosage	0			-0.053889729	⊕⊕○○ Low	-0.053889729	⊕⊕○○ Low
187	Empagliflozin_low_dosage:Ertugliflozin_low_dosage	0			-0.344140139	⊕⊕○○ Low	-0.344140139	⊕⊕○○ Low
188	Empagliflozin_low_dosage:Exenatide	0			0.334740071	⊕⊕○○ Low	0.334740071	⊕⊕○○ Low
189	Empagliflozin_low_dosage:Inject_semaglutide_high_dosage	0			-0.075965133	⊕⊕○○ Low	-0.075965133	⊕⊕○○ Low
190	Empagliflozin_low_dosage:Inject_semaglutide_low_dosage	0			-0.455759376	⊕⊕○○ Low	-0.455759376	⊕⊕○○ Low
191	Empagliflozin_low_dosage:Inject_semaglutide_medium_dosage	0			0.258492375	⊕⊕○○ Low	0.258492375	⊕⊕○○ Low
192	Empagliflozin_low_dosage:Liraglutide	0			-0.482041964	⊕⊕○○ Low	-0.482041964	⊕⊕○○ Low
193	Empagliflozin_low_dosage:Lixisenatide	0			0.771247982	⊕⊕○○ Low	0.771247982	⊕⊕○○ Low
194	Empagliflozin_low_dosage:Oral_semaglutide	0			-0.571572184	⊕⊕○○ Low	-0.571572184	⊕⊕○○ Low

195	Empagliflozin_low_dosage:Placebo_or_Control	4	-0.346566496	⊕⊕⊕⊕ High	3.432330654	⊕⊕⊕○ Medium	-0.344507584	⊕⊕⊕⊕ High
196	Empagliflozin_low_dosage:Sotagliflozin	0			-0.01594075	⊕⊕○○ Low	-0.01594075	⊕⊕○○ Low
197	Empagliflozin_low_dosage:Tirzepatide_high_dosage	0			-0.989706746	⊕⊕○○ Low	-0.989706746	⊕⊕○○ Low
198	Empagliflozin_low_dosage:Tirzepatide_low_dosage	0			-0.155795359	⊕⊕○○ Low	-0.155795359	⊕⊕○○ Low
199	Ertugliflozin_high_dosage:Ertugliflozin_low_dosage	1	-0.29025041	⊕⊕⊕⊕ High			-0.29025041	⊕⊕⊕○ Medium
200	Ertugliflozin_high_dosage:Exenatide	0			0.388629801	⊕⊕○○ Low	0.388629801	⊕⊕○○ Low
201	Ertugliflozin_high_dosage:Inject_semaglutide_high_dosage	0			-0.022075403	⊕⊕○○ Low	-0.022075403	⊕⊕○○ Low
202	Ertugliflozin_high_dosage:Inject_semaglutide_low_dosage	0			-0.401869647	⊕⊕○○ Low	-0.401869647	⊕⊕○○ Low
203	Ertugliflozin_high_dosage:Inject_semaglutide_medium_dosage	0			0.312382105	⊕⊕○○ Low	0.312382105	⊕⊕○○ Low
204	Ertugliflozin_high_dosage:Liraglutide	0			-0.428152234	⊕⊕○○ Low	-0.428152234	⊕⊕○○ Low
205	Ertugliflozin_high_dosage:Lixisenatide	0			0.825137711	⊕⊕○○ Low	0.825137711	⊕⊕○○ Low
206	Ertugliflozin_high_dosage:Oral_semaglutide	0			-0.517682455	⊕⊕○○ Low	-0.517682455	⊕⊕○○ Low
207	Ertugliflozin_high_dosage:Placebo_or_Control	1	-0.290617854	⊕⊕⊕⊕ High			-0.290617854	⊕⊕⊕○ Medium
208	Ertugliflozin_high_dosage:Sotagliflozin	0			0.037948979	⊕⊕○○ Low	0.037948979	⊕⊕○○ Low
209	Ertugliflozin_high_dosage:Tirzepatide_high_dosage	0			-0.935817017	⊕⊕○○ Low	-0.935817017	⊕⊕○○ Low
210	Ertugliflozin_high_dosage:Tirzepatide_low_dosage	0			-0.10190563	⊕⊕○○ Low	-0.10190563	⊕⊕○○ Low
211	Ertugliflozin_low_dosage:Exenatide	0			0.678880211	⊕⊕○○ Low	0.678880211	⊕⊕○○ Low
212	Ertugliflozin_low_dosage:Inject_semaglutide_high_dosage	0			0.268175007	⊕⊕○○ Low	0.268175007	⊕⊕○○ Low
213	Ertugliflozin_low_dosage:Inject_semaglutide_low_dosage	0			-0.111619237	⊕⊕○○ Low	-0.111619237	⊕⊕○○ Low
214	Ertugliflozin_low_dosage:Inject_semaglutide_medium_dosage	0			0.602632515	⊕⊕○○ Low	0.602632515	⊕⊕○○ Low
215	Ertugliflozin_low_dosage:Liraglutide	0			-0.137901824	⊕⊕○○ Low	-0.137901824	⊕⊕○○ Low
216	Ertugliflozin_low_dosage:Lixisenatide	0			1.115388121	⊕⊕○○ Low	1.115388121	⊕⊕○○ Low

217	Ertugliflozin_low_dosage:Oral_semaglutide	0			-0.227432045	⊕⊕○○ Low	-0.227432045	⊕⊕○○ Low
218	Ertugliflozin_low_dosage:Placebo_or_Control	1	-0.000367444	⊕⊕⊕⊕ High			-0.000367444	⊕⊕⊕○ Medium
219	Ertugliflozin_low_dosage:Sotagliflozin	0			0.328199389	⊕⊕○○ Low	0.328199389	⊕⊕○○ Low
220	Ertugliflozin_low_dosage:Tirzepatide_high_dosage	0			-0.645566607	⊕⊕○○ Low	-0.645566607	⊕⊕○○ Low
221	Ertugliflozin_low_dosage:Tirzepatide_low_dosage	0			0.18834478	⊕⊕○○ Low	0.18834478	⊕⊕○○ Low
222	Exenatide:Inject_semaglutide_high_dosage	0			-0.410705204	⊕⊕○○ Low	-0.410705204	⊕⊕○○ Low
223	Exenatide:Inject_semaglutide_low_dosage	0			-0.790499448	⊕⊕○○ Low	-0.790499448	⊕⊕○○ Low
224	Exenatide:Inject_semaglutide_medium_dosage	0			-0.076247696	⊕⊕○○ Low	-0.076247696	⊕⊕○○ Low
225	Exenatide:Liraglutide	0			-0.816782035	⊕⊕○○ Low	-0.816782035	⊕⊕○○ Low
226	Exenatide:Lixisenatide	0			0.43650791	⊕⊕○○ Low	0.43650791	⊕⊕○○ Low
227	Exenatide:Oral_semaglutide	0			-0.906312256	⊕⊕○○ Low	-0.906312256	⊕⊕○○ Low
228	Exenatide:Placebo_or_Control	3	-0.679247655	⊕⊕⊕⊕ High			-0.679247655	⊕⊕⊕○ Medium
229	Exenatide:Sotagliflozin	0			-0.350680822	⊕⊕○○ Low	-0.350680822	⊕⊕○○ Low
230	Exenatide:Tirzepatide_high_dosage	0			-1.324446818	⊕⊕○○ Low	-1.324446818	⊕⊕○○ Low
231	Exenatide:Tirzepatide_low_dosage	0			-0.490535431	⊕⊕○○ Low	-0.490535431	⊕⊕○○ Low
232	Inject_semaglutide_high_dosage:Inject_semaglutide_low_dosage	0			-0.379794243	⊕⊕○○ Low	-0.379794243	⊕⊕○○ Low
233	Inject_semaglutide_high_dosage:Inject_semaglutide_medium_dosage	1	0.405465108	⊕⊕⊕⊕ High	0.321607054	⊕⊕⊕⊕ High	0.334457508	⊕⊕⊕⊕ High
234	Inject_semaglutide_high_dosage:Liraglutide	1	1.114422895	⊕⊕⊕⊕ High	-0.42688866	⊕⊕⊕○ Medium	-0.406076831	⊕⊕⊕⊕ High
235	Inject_semaglutide_high_dosage:Lixisenatide	0			0.847213114	⊕⊕○○ Low	0.847213114	⊕⊕○○ Low
236	Inject_semaglutide_high_dosage:Oral_semaglutide	0			-0.495607052	⊕⊕○○ Low	-0.495607052	⊕⊕○○ Low
237	Inject_semaglutide_high_dosage:Placebo_or_Control	4	-0.274385957	⊕⊕⊕⊕ High	0.026610365	⊕⊕⊕○ Medium	-0.268542451	⊕⊕⊕⊕ High
238	Inject_semaglutide_high_dosage:Sotagliflozin	0			0.060024382	⊕⊕○○ Low	0.060024382	⊕⊕○○ Low

239	Inject_semaglutide_high_dosage:Tirzepatide_high_dosage	0			-0.913741614	⊕⊕○○ Low	-0.913741614	⊕⊕○○ Low
240	Inject_semaglutide_high_dosage:Tirzepatide_low_dosage	0			-0.079830226	⊕⊕○○ Low	-0.079830226	⊕⊕○○ Low
241	Inject_semaglutide_low_dosage:Inject_semaglutide_medium_dosage	2	0.894447144	⊕⊕⊕⊕ High	-0.351161573	⊕⊕⊕○ Medium	0.714251751	⊕⊕⊕⊕ High
242	Inject_semaglutide_low_dosage:Liraglutide	1	-0.837589046	⊕⊕⊕⊕ High	5.04E-05	⊕⊕⊕○ Medium	-0.026282588	⊕⊕⊕⊕ High
243	Inject_semaglutide_low_dosage:Lixisenatide	0			1.227007358	⊕⊕○○ Low	1.227007358	⊕⊕○○ Low
244	Inject_semaglutide_low_dosage:Oral_semaglutide	0			-0.115812808	⊕⊕○○ Low	-0.115812808	⊕⊕○○ Low
245	Inject_semaglutide_low_dosage:Placebo_or_Control	2	0.129468118	⊕⊕⊕⊕ High	-0.221474042	⊕⊕⊕○ Medium	0.111251792	⊕⊕⊕⊕ High
246	Inject_semaglutide_low_dosage:Sotagliflozin	0			0.439818626	⊕⊕○○ Low	0.439818626	⊕⊕○○ Low
247	Inject_semaglutide_low_dosage:Tirzepatide_high_dosage	0			-0.53394737	⊕⊕○○ Low	-0.53394737	⊕⊕○○ Low
248	Inject_semaglutide_low_dosage:Tirzepatide_low_dosage	0			0.299964017	⊕⊕○○ Low	0.299964017	⊕⊕○○ Low
249	Inject_semaglutide_medium_dosage:Liraglutide	0			-0.740534339	⊕⊕○○ Low	-0.740534339	⊕⊕○○ Low
250	Inject_semaglutide_medium_dosage:Lixisenatide	0			0.512755606	⊕⊕○○ Low	0.512755606	⊕⊕○○ Low
251	Inject_semaglutide_medium_dosage:Oral_semaglutide	0			-0.83006456	⊕⊕○○ Low	-0.83006456	⊕⊕○○ Low
252	Inject_semaglutide_medium_dosage:Placebo_or_Control	3	-0.639786405	⊕⊕⊕⊕ High	-0.37956783	⊕⊕⊕⊕ High	-0.602999959	⊕⊕⊕⊕ High
253	Inject_semaglutide_medium_dosage:Sotagliflozin	0			-0.274433126	⊕⊕○○ Low	-0.274433126	⊕⊕○○ Low
254	Inject_semaglutide_medium_dosage:Tirzepatide_high_dosage	1	-0.405997731	⊕⊕⊕⊕ High	-1.404502888	⊕⊕⊕⊕ High	-1.248199122	⊕⊕⊕⊕ High
255	Inject_semaglutide_medium_dosage:Tirzepatide_low_dosage	0			-0.414287734	⊕⊕○○ Low	-0.414287734	⊕⊕○○ Low
256	Liraglutide:Lixisenatide	0			1.253289945	⊕⊕○○ Low	1.253289945	⊕⊕○○ Low
257	Liraglutide:Oral_semaglutide	0			-0.089530221	⊕⊕○○ Low	-0.089530221	⊕⊕○○ Low
258	Liraglutide:Placebo_or_Control	3	0.145104586	⊕⊕⊕⊕ High	-0.155254686	⊕⊕⊕○ Medium	0.13753438	⊕⊕⊕⊕ High
259	Liraglutide:Sotagliflozin	0			0.466101213	⊕⊕○○ Low	0.466101213	⊕⊕○○ Low
260	Liraglutide:Tirzepatide_high_dosage	0			-0.507664783	⊕⊕○○ Low	-0.507664783	⊕⊕○○ Low

261	Liraglutide:Tirzepatide_low_dosage	0			0.326246605	⊕⊕○○ Low	0.326246605	⊕⊕○○ Low
262	Lixisenatide:Oral_semaglutide	0			-1.342820166	⊕⊕○○ Low	-1.342820166	⊕⊕○○ Low
263	Lixisenatide:Placebo_or_Control	1	-1.115755566	⊕⊕⊕⊕ High			-1.115755566	⊕⊕⊕○ Medium
264	Lixisenatide:Sotagliflozin	0			-0.787188732	⊕⊕○○ Low	-0.787188732	⊕⊕○○ Low
265	Lixisenatide:Tirzepatide_high_dosage	0			-1.760954728	⊕⊕○○ Low	-1.760954728	⊕⊕○○ Low
266	Lixisenatide:Tirzepatide_low_dosage	0			-0.927043341	⊕⊕○○ Low	-0.927043341	⊕⊕○○ Low
267	Oral_semaglutide:Placebo_or_Control	1	1.104345017	⊕⊕⊕⊕ High	-0.592131494	⊕⊕⊕○ Medium	0.227064601	⊕⊕⊕⊕ High
268	Oral_semaglutide:Sotagliflozin	0			0.555631434	⊕⊕○○ Low	0.555631434	⊕⊕○○ Low
269	Oral_semaglutide:Tirzepatide_high_dosage	0			-0.418134562	⊕⊕○○ Low	-0.418134562	⊕⊕○○ Low
270	Oral_semaglutide:Tirzepatide_low_dosage	0			0.415776825	⊕⊕○○ Low	0.415776825	⊕⊕○○ Low
271	Sotagliflozin:Placebo_or_Control	2	-0.328566833	⊕⊕⊕⊕ High			-0.328566833	⊕⊕⊕○ Medium
272	Tirzepatide_high_dosage:Placebo_or_Control	5	0.821141732	⊕⊕⊕⊕ High	0.092595377	⊕⊕⊕⊕ High	0.645199163	⊕⊕⊕⊕ High
273	Tirzepatide_low_dosage:Placebo_or_Control	0			-0.188712225	⊕⊕○○ Low	-0.188712225	⊕⊕○○ Low
274	Sotagliflozin:Tirzepatide_high_dosage	0			-0.973765996	⊕⊕○○ Low	-0.973765996	⊕⊕○○ Low
275	Sotagliflozin:Tirzepatide_low_dosage	0			-0.139854609	⊕⊕○○ Low	-0.139854609	⊕⊕○○ Low
276	Tirzepatide_high_dosage:Tirzepatide_low_dosage	3	0.833911387	⊕⊕⊕⊕ High			0.833911387	⊕⊕⊕○ Medium

Table S8C: GRADE of safety profile: drop-out rate

	Comparison	No.Studies	Direct		Indirect		NMA	
			Estimate	Rate	Estimate	Rate	Estimate	Rate
1	Albiglutide:Bexagliflozin	0			0.168761461	⊕⊕○○ Low	0.168761461	⊕⊕○○ Low
2	Albiglutide:Canagliflozin_high_dosage	0			0.239326586	⊕⊕○○ Low	0.239326586	⊕⊕○○ Low
3	Albiglutide:Canagliflozin_low_dosage	0			0.139943756	⊕⊕○○ Low	0.139943756	⊕⊕○○ Low
4	Albiglutide:Dapagliflozin	0			0.04711762	⊕⊕○○ Low	0.04711762	⊕⊕○○ Low
5	Albiglutide:Dulaglutide	0			-0.003763008	⊕⊕○○ Low	-0.003763008	⊕⊕○○ Low
6	Albiglutide:Efpeglenatide_high_dosage	0			-0.198324494	⊕⊕○○ Low	-0.198324494	⊕⊕○○ Low
7	Albiglutide:Efpeglenatide_low_dosage	0			0.074179343	⊕⊕○○ Low	0.074179343	⊕⊕○○ Low
8	Albiglutide:Efpeglenatide_medium_dosage	0			-0.061699272	⊕⊕○○ Low	-0.061699272	⊕⊕○○ Low
9	Albiglutide:Empagliflozin_high_dosage	0			-0.033394573	⊕⊕○○ Low	-0.033394573	⊕⊕○○ Low
10	Albiglutide:Empagliflozin_low_dosage	0			0.026607176	⊕⊕○○ Low	0.026607176	⊕⊕○○ Low
11	Albiglutide:Ertugliflozin_high_dosage	0			-0.111425201	⊕⊕○○ Low	-0.111425201	⊕⊕○○ Low
12	Albiglutide:Ertugliflozin_low_dosage	0			-0.049552366	⊕⊕○○ Low	-0.049552366	⊕⊕○○ Low
13	Albiglutide:Exenatide	0			-0.232934493	⊕⊕○○ Low	-0.232934493	⊕⊕○○ Low
14	Albiglutide:Inject_semaglutide_high_dosage	0			0.051466782	⊕⊕○○ Low	0.051466782	⊕⊕○○ Low
15	Albiglutide:Inject_semaglutide_low_dosage	0			0.015065088	⊕⊕○○ Low	0.015065088	⊕⊕○○ Low
16	Albiglutide:Inject_semaglutide_medium_dosage	0			0.257714332	⊕⊕○○ Low	0.257714332	⊕⊕○○ Low
17	Albiglutide:Liraglutide	1	-0.176557852	⊕⊕⊕⊕ High	0.064894459	⊕⊕⊕○ Medium	-0.019267852	⊕⊕⊕⊕ High
18	Albiglutide:Lixisenatide	0			-0.101478093	⊕⊕○○ Low	-0.101478093	⊕⊕○○ Low
19	Albiglutide:Oral_semaglutide	0			-0.158601236	⊕⊕○○ Low	-0.158601236	⊕⊕○○ Low

20	Albiglutide:Placebo_or_Control	2	-0.057711774	⊕⊕⊕⊕ High	-0.299164085	⊕⊕⊕⊕ High	-0.114465289	⊕⊕⊕⊕ High
21	Albiglutide:Sotagliflozin	0			0.059597369	⊕⊕○○ Low	0.059597369	⊕⊕○○ Low
22	Albiglutide:Tirzepatide_high_dosage	0			0.362147233	⊕⊕○○ Low	0.362147233	⊕⊕○○ Low
23	Albiglutide:Tirzepatide_low_dosage	0			0.466159785	⊕⊕○○ Low	0.466159785	⊕⊕○○ Low
24	Bexagliflozin:Canagliflozin_high_dosage	0			0.070565125	⊕⊕○○ Low	0.070565125	⊕⊕○○ Low
25	Bexagliflozin:Canagliflozin_low_dosage	0			-0.028817705	⊕⊕○○ Low	-0.028817705	⊕⊕○○ Low
26	Bexagliflozin:Dapagliflozin	0			-0.121643841	⊕⊕○○ Low	-0.121643841	⊕⊕○○ Low
27	Bexagliflozin:Dulaglutide	0			-0.17252447	⊕⊕○○ Low	-0.17252447	⊕⊕○○ Low
28	Bexagliflozin:Efpeglenatide_high_dosage	0			-0.367085955	⊕⊕○○ Low	-0.367085955	⊕⊕○○ Low
29	Bexagliflozin:Efpeglenatide_low_dosage	0			-0.094582118	⊕⊕○○ Low	-0.094582118	⊕⊕○○ Low
30	Bexagliflozin:Efpeglenatide_medium_dosage	0			-0.230460734	⊕⊕○○ Low	-0.230460734	⊕⊕○○ Low
31	Bexagliflozin:Empagliflozin_high_dosage	0			-0.202156034	⊕⊕○○ Low	-0.202156034	⊕⊕○○ Low
32	Bexagliflozin:Empagliflozin_low_dosage	0			-0.142154286	⊕⊕○○ Low	-0.142154286	⊕⊕○○ Low
33	Bexagliflozin:Ertugliflozin_high_dosage	0			-0.280186663	⊕⊕○○ Low	-0.280186663	⊕⊕○○ Low
34	Bexagliflozin:Ertugliflozin_low_dosage	0			-0.218313828	⊕⊕○○ Low	-0.218313828	⊕⊕○○ Low
35	Bexagliflozin:Exenatide	0			-0.401695954	⊕⊕○○ Low	-0.401695954	⊕⊕○○ Low
36	Bexagliflozin:Inject_semaglutide_high_dosage	0			-0.117294679	⊕⊕○○ Low	-0.117294679	⊕⊕○○ Low
37	Bexagliflozin:Inject_semaglutide_low_dosage	0			-0.153696374	⊕⊕○○ Low	-0.153696374	⊕⊕○○ Low
38	Bexagliflozin:Inject_semaglutide_medium_dosage	0			0.08895287	⊕⊕○○ Low	0.08895287	⊕⊕○○ Low
39	Bexagliflozin:Liraglutide	0			-0.188029313	⊕⊕○○ Low	-0.188029313	⊕⊕○○ Low
40	Bexagliflozin:Lixisenatide	0			-0.270239555	⊕⊕○○ Low	-0.270239555	⊕⊕○○ Low
41	Bexagliflozin:Oral_semaglutide	0			-0.327362697	⊕⊕○○ Low	-0.327362697	⊕⊕○○ Low

42	Bexagliflozin:Placebo_or_Control	2	-0.28322675	⊕⊕⊕⊕ High			-0.28322675	⊕⊕⊕○ Medium
43	Bexagliflozin:Sotagliflozin	0			-0.109164092	⊕⊕○○ Low	-0.109164092	⊕⊕○○ Low
44	Bexagliflozin:Tirzepatide_high_dosage	0			0.193385772	⊕⊕○○ Low	0.193385772	⊕⊕○○ Low
45	Bexagliflozin:Tirzepatide_low_dosage	0			0.297398323	⊕⊕○○ Low	0.297398323	⊕⊕○○ Low
46	Canagliflozin_high_dosage:Canagliflozin_low_dosage	3	-0.099660929	⊕⊕⊕⊕ High	-0.098083287	⊕⊕⊕⊕ High	-0.09938283	⊕⊕⊕⊕ High
47	Canagliflozin_high_dosage:Dapagliflozin	0			-0.192208965	⊕⊕○○ Low	-0.192208965	⊕⊕○○ Low
48	Canagliflozin_high_dosage:Dulaglutide	0			-0.243089594	⊕⊕○○ Low	-0.243089594	⊕⊕○○ Low
49	Canagliflozin_high_dosage:Efpeglenatide_high_dosage	0			-0.43765108	⊕⊕○○ Low	-0.43765108	⊕⊕○○ Low
50	Canagliflozin_high_dosage:Efpeglenatide_low_dosage	0			-0.165147242	⊕⊕○○ Low	-0.165147242	⊕⊕○○ Low
51	Canagliflozin_high_dosage:Efpeglenatide_medium_dosage	0			-0.301025858	⊕⊕○○ Low	-0.301025858	⊕⊕○○ Low
52	Canagliflozin_high_dosage:Empagliflozin_high_dosage	0			-0.272721159	⊕⊕○○ Low	-0.272721159	⊕⊕○○ Low
53	Canagliflozin_high_dosage:Empagliflozin_low_dosage	0			-0.21271941	⊕⊕○○ Low	-0.21271941	⊕⊕○○ Low
54	Canagliflozin_high_dosage:Ertugliflozin_high_dosage	0			-0.350751787	⊕⊕○○ Low	-0.350751787	⊕⊕○○ Low
55	Canagliflozin_high_dosage:Ertugliflozin_low_dosage	0			-0.288878952	⊕⊕○○ Low	-0.288878952	⊕⊕○○ Low
56	Canagliflozin_high_dosage:Exenatide	0			-0.472261079	⊕⊕○○ Low	-0.472261079	⊕⊕○○ Low
57	Canagliflozin_high_dosage:Inject_semaglutide_high_dosage	0			-0.187859804	⊕⊕○○ Low	-0.187859804	⊕⊕○○ Low
58	Canagliflozin_high_dosage:Inject_semaglutide_low_dosage	0			-0.224261498	⊕⊕○○ Low	-0.224261498	⊕⊕○○ Low
59	Canagliflozin_high_dosage:Inject_semaglutide_medium_dosage	0			0.018387746	⊕⊕○○ Low	0.018387746	⊕⊕○○ Low
60	Canagliflozin_high_dosage:Liraglutide	0			-0.258594438	⊕⊕○○ Low	-0.258594438	⊕⊕○○ Low
61	Canagliflozin_high_dosage:Lixisenatide	0			-0.340804679	⊕⊕○○ Low	-0.340804679	⊕⊕○○ Low
62	Canagliflozin_high_dosage:Oral_semaglutide	0			-0.397927822	⊕⊕○○ Low	-0.397927822	⊕⊕○○ Low
63	Canagliflozin_high_dosage:Placebo_or_Control	4	-0.380799362	⊕⊕⊕⊕ High	-0.026496228	⊕⊕⊕⊕ High	-0.353791875	⊕⊕⊕⊕ High

64	Canagliflozin_high_dosage:Sotagliflozin	0			-0.179729217	⊕⊕○○ Low	-0.179729217	⊕⊕○○ Low
65	Canagliflozin_high_dosage:Tirzepatide_high_dosage	0			0.122820647	⊕⊕○○ Low	0.122820647	⊕⊕○○ Low
66	Canagliflozin_high_dosage:Tirzepatide_low_dosage	0			0.226833199	⊕⊕○○ Low	0.226833199	⊕⊕○○ Low
67	Canagliflozin_low_dosage:Dapagliflozin	0			-0.092826136	⊕⊕○○ Low	-0.092826136	⊕⊕○○ Low
68	Canagliflozin_low_dosage:Dulaglutide	0			-0.143706765	⊕⊕○○ Low	-0.143706765	⊕⊕○○ Low
69	Canagliflozin_low_dosage:Efpeglenatide_high_dosage	0			-0.33826825	⊕⊕○○ Low	-0.33826825	⊕⊕○○ Low
70	Canagliflozin_low_dosage:Efpeglenatide_low_dosage	0			-0.065764413	⊕⊕○○ Low	-0.065764413	⊕⊕○○ Low
71	Canagliflozin_low_dosage:Efpeglenatide_medium_dosage	0			-0.201643029	⊕⊕○○ Low	-0.201643029	⊕⊕○○ Low
72	Canagliflozin_low_dosage:Empagliflozin_high_dosage	0			-0.173338329	⊕⊕○○ Low	-0.173338329	⊕⊕○○ Low
73	Canagliflozin_low_dosage:Empagliflozin_low_dosage	0			-0.11333658	⊕⊕○○ Low	-0.11333658	⊕⊕○○ Low
74	Canagliflozin_low_dosage:Ertugliflozin_high_dosage	0			-0.251368957	⊕⊕○○ Low	-0.251368957	⊕⊕○○ Low
75	Canagliflozin_low_dosage:Ertugliflozin_low_dosage	0			-0.189496123	⊕⊕○○ Low	-0.189496123	⊕⊕○○ Low
76	Canagliflozin_low_dosage:Exenatide	0			-0.372878249	⊕⊕○○ Low	-0.372878249	⊕⊕○○ Low
77	Canagliflozin_low_dosage:Inject_semaglutide_high_dosage	0			-0.088476974	⊕⊕○○ Low	-0.088476974	⊕⊕○○ Low
78	Canagliflozin_low_dosage:Inject_semaglutide_low_dosage	0			-0.124878669	⊕⊕○○ Low	-0.124878669	⊕⊕○○ Low
79	Canagliflozin_low_dosage:Inject_semaglutide_medium_dosage	0			0.117770576	⊕⊕○○ Low	0.117770576	⊕⊕○○ Low
80	Canagliflozin_low_dosage:Liraglutide	0			-0.159211608	⊕⊕○○ Low	-0.159211608	⊕⊕○○ Low
81	Canagliflozin_low_dosage:Lixisenatide	0			-0.24142185	⊕⊕○○ Low	-0.24142185	⊕⊕○○ Low
82	Canagliflozin_low_dosage:Oral_semaglutide	0			-0.298544992	⊕⊕○○ Low	-0.298544992	⊕⊕○○ Low
83	Canagliflozin_low_dosage:Placebo_or_Control	6	-0.272717257	⊕⊕⊕⊕ High	0.204265745	⊕⊕⊕○ Medium	-0.254409045	⊕⊕⊕⊕ High
84	Canagliflozin_low_dosage:Sotagliflozin	0			-0.080346387	⊕⊕○○ Low	-0.080346387	⊕⊕○○ Low
85	Canagliflozin_low_dosage:Tirzepatide_high_dosage	0			0.222203477	⊕⊕○○ Low	0.222203477	⊕⊕○○ Low

86	Canagliflozin_low_dosage:Tirzepatide_low_dosage	0			0.326216028	⊕⊕○○ Low	0.326216028	⊕⊕○○ Low
87	Dapagliflozin:Dulaglutide	0			-0.050880629	⊕⊕○○ Low	-0.050880629	⊕⊕○○ Low
88	Dapagliflozin:Efpeglenatide_high_dosage	0			-0.245442115	⊕⊕○○ Low	-0.245442115	⊕⊕○○ Low
89	Dapagliflozin:Efpeglenatide_low_dosage	0			0.027061723	⊕⊕○○ Low	0.027061723	⊕⊕○○ Low
90	Dapagliflozin:Efpeglenatide_medium_dosage	0			-0.108816893	⊕⊕○○ Low	-0.108816893	⊕⊕○○ Low
91	Dapagliflozin:Empagliflozin_high_dosage	0			-0.080512194	⊕⊕○○ Low	-0.080512194	⊕⊕○○ Low
92	Dapagliflozin:Empagliflozin_low_dosage	0			-0.020510445	⊕⊕○○ Low	-0.020510445	⊕⊕○○ Low
93	Dapagliflozin:Ertugliflozin_high_dosage	0			-0.158542822	⊕⊕○○ Low	-0.158542822	⊕⊕○○ Low
94	Dapagliflozin:Ertugliflozin_low_dosage	0			-0.096669987	⊕⊕○○ Low	-0.096669987	⊕⊕○○ Low
95	Dapagliflozin:Exenatide	0			-0.280052113	⊕⊕○○ Low	-0.280052113	⊕⊕○○ Low
96	Dapagliflozin:Inject_semaglutide_high_dosage	0			0.004349162	⊕⊕○○ Low	0.004349162	⊕⊕○○ Low
97	Dapagliflozin:Inject_semaglutide_low_dosage	0			-0.032052533	⊕⊕○○ Low	-0.032052533	⊕⊕○○ Low
98	Dapagliflozin:Inject_semaglutide_medium_dosage	0			0.210596711	⊕⊕○○ Low	0.210596711	⊕⊕○○ Low
99	Dapagliflozin:Liraglutide	0			-0.066385473	⊕⊕○○ Low	-0.066385473	⊕⊕○○ Low
100	Dapagliflozin:Lixisenatide	0			-0.148595714	⊕⊕○○ Low	-0.148595714	⊕⊕○○ Low
101	Dapagliflozin:Oral_semaglutide	0			-0.205718857	⊕⊕○○ Low	-0.205718857	⊕⊕○○ Low
102	Dapagliflozin:Placebo_or_Control	9	-0.161582909	⊕⊕⊕⊕ High			-0.161582909	⊕⊕⊕○ Medium
103	Dapagliflozin:Sotagliflozin	0			0.012479749	⊕⊕○○ Low	0.012479749	⊕⊕○○ Low
104	Dapagliflozin:Tirzepatide_high_dosage	0			0.315029612	⊕⊕○○ Low	0.315029612	⊕⊕○○ Low
105	Dapagliflozin:Tirzepatide_low_dosage	0			0.419042164	⊕⊕○○ Low	0.419042164	⊕⊕○○ Low
106	Dulaglutide:Efpeglenatide_high_dosage	1	-0.380215048	⊕⊕⊕⊕ High	0.001067689	⊕⊕⊕○ Medium	-0.194561486	⊕⊕⊕⊕ High
107	Dulaglutide:Efpeglenatide_low_dosage	0			0.077942352	⊕⊕○○ Low	0.077942352	⊕⊕○○ Low

108	Dulaglutide:Efpeglenatide_medium_dosage	1	0.043823744	⊕⊕⊕⊕ High	-0.161649348	⊕⊕⊕○ Medium	-0.057936264	⊕⊕⊕⊕ High
109	Dulaglutide:Empagliflozin_high_dosage	0			-0.029631565	⊕⊕○○ Low	-0.029631565	⊕⊕○○ Low
110	Dulaglutide:Empagliflozin_low_dosage	0			0.030370184	⊕⊕○○ Low	0.030370184	⊕⊕○○ Low
111	Dulaglutide:Ertugliflozin_high_dosage	0			-0.107662193	⊕⊕○○ Low	-0.107662193	⊕⊕○○ Low
112	Dulaglutide:Ertugliflozin_low_dosage	0			-0.045789358	⊕⊕○○ Low	-0.045789358	⊕⊕○○ Low
113	Dulaglutide:Exenatide	0			-0.229171485	⊕⊕○○ Low	-0.229171485	⊕⊕○○ Low
114	Dulaglutide:Inject_semaglutide_high_dosage	0			0.05522979	⊕⊕○○ Low	0.05522979	⊕⊕○○ Low
115	Dulaglutide:Inject_semaglutide_low_dosage	0			0.018828096	⊕⊕○○ Low	0.018828096	⊕⊕○○ Low
116	Dulaglutide:Inject_semaglutide_medium_dosage	0			0.26147734	⊕⊕○○ Low	0.26147734	⊕⊕○○ Low
117	Dulaglutide:Liraglutide	1	-0.032789823	⊕⊕⊕⊕ High	-0.00921066	⊕⊕⊕⊕ High	-0.015504844	⊕⊕⊕⊕ High
118	Dulaglutide:Lixisenatide	0			-0.097715085	⊕⊕○○ Low	-0.097715085	⊕⊕○○ Low
119	Dulaglutide:Oral_semaglutide	0			-0.154838228	⊕⊕○○ Low	-0.154838228	⊕⊕○○ Low
120	Dulaglutide:Placebo_or_Control	3	0.023852281	⊕⊕⊕⊕ High	-0.217070243	⊕⊕⊕○ Medium	-0.11070228	⊕⊕⊕⊕ High
121	Dulaglutide:Sotagliflozin	0			0.063360378	⊕⊕○○ Low	0.063360378	⊕⊕○○ Low
122	Dulaglutide:Tirzepatide_high_dosage	1	-0.234689542	⊕⊕⊕⊕ High	0.435820857	⊕⊕⊕○ Medium	0.365910241	⊕⊕⊕⊕ High
123	Dulaglutide:Tirzepatide_low_dosage	1	-0.202940844	⊕⊕⊕⊕ High	0.610521473	⊕⊕⊕○ Medium	0.469922793	⊕⊕⊕⊕ High
124	Efpeglenatide_high_dosage:Efpeglenatide_low_dosage	1	0.146603474	⊕⊕⊕⊕ High	0.600123534	⊕⊕⊕⊕ High	0.272503838	⊕⊕⊕⊕ High
125	Efpeglenatide_high_dosage:Efpeglenatide_medium_dosage	3	0.136479916	⊕⊕⊕⊕ High			0.136625222	⊕⊕⊕○ Medium
126	Efpeglenatide_high_dosage:Empagliflozin_high_dosage	0			0.164929921	⊕⊕○○ Low	0.164929921	⊕⊕○○ Low
127	Efpeglenatide_high_dosage:Empagliflozin_low_dosage	0			0.22493167	⊕⊕○○ Low	0.22493167	⊕⊕○○ Low
128	Efpeglenatide_high_dosage:Ertugliflozin_high_dosage	0			0.086899293	⊕⊕○○ Low	0.086899293	⊕⊕○○ Low
129	Efpeglenatide_high_dosage:Ertugliflozin_low_dosage	0			0.148772128	⊕⊕○○ Low	0.148772128	⊕⊕○○ Low

130	Efpeglenatide_high_dosage:Exenatide	0			-0.034609999	⊕⊕○○ Low	-0.034609999	⊕⊕○○ Low
131	Efpeglenatide_high_dosage:Inject_semaglutide_high_dosage	0			0.249791276	⊕⊕○○ Low	0.249791276	⊕⊕○○ Low
132	Efpeglenatide_high_dosage:Inject_semaglutide_low_dosage	0			0.213389582	⊕⊕○○ Low	0.213389582	⊕⊕○○ Low
133	Efpeglenatide_high_dosage:Inject_semaglutide_medium_dosage	0			0.456038826	⊕⊕○○ Low	0.456038826	⊕⊕○○ Low
134	Efpeglenatide_high_dosage:Liraglutide	0			0.179056642	⊕⊕○○ Low	0.179056642	⊕⊕○○ Low
135	Efpeglenatide_high_dosage:Lixisenatide	0			0.096846401	⊕⊕○○ Low	0.096846401	⊕⊕○○ Low
136	Efpeglenatide_high_dosage:Oral_semaglutide	0			0.039723258	⊕⊕○○ Low	0.039723258	⊕⊕○○ Low
137	Efpeglenatide_high_dosage:Placebo_or_Control	2	-0.012996443	⊕⊕⊕⊕ High	0.371963363	⊕⊕⊕○ Medium	0.083859205	⊕⊕⊕⊕ High
138	Efpeglenatide_high_dosage:Sotagliflozin	0			0.257921863	⊕⊕○○ Low	0.257921863	⊕⊕○○ Low
139	Efpeglenatide_high_dosage:Tirzepatide_high_dosage	0			0.560471727	⊕⊕○○ Low	0.560471727	⊕⊕○○ Low
140	Efpeglenatide_high_dosage:Tirzepatide_low_dosage	0			0.664484279	⊕⊕○○ Low	0.664484279	⊕⊕○○ Low
141	Efpeglenatide_low_dosage:Efpeglenatide_medium_dosage	1	-0.284258584	⊕⊕⊕⊕ High	0.279056171	⊕⊕⊕○ Medium	-0.135878616	⊕⊕⊕⊕ High
142	Efpeglenatide_low_dosage:Empagliflozin_high_dosage	0			-0.107573917	⊕⊕○○ Low	-0.107573917	⊕⊕○○ Low
143	Efpeglenatide_low_dosage:Empagliflozin_low_dosage	0			-0.047572168	⊕⊕○○ Low	-0.047572168	⊕⊕○○ Low
144	Efpeglenatide_low_dosage:Ertugliflozin_high_dosage	0			-0.185604545	⊕⊕○○ Low	-0.185604545	⊕⊕○○ Low
145	Efpeglenatide_low_dosage:Ertugliflozin_low_dosage	0			-0.12373171	⊕⊕○○ Low	-0.12373171	⊕⊕○○ Low
146	Efpeglenatide_low_dosage:Exenatide	0			-0.307113836	⊕⊕○○ Low	-0.307113836	⊕⊕○○ Low
147	Efpeglenatide_low_dosage:Inject_semaglutide_high_dosage	0			-0.022712561	⊕⊕○○ Low	-0.022712561	⊕⊕○○ Low
148	Efpeglenatide_low_dosage:Inject_semaglutide_low_dosage	0			-0.059114256	⊕⊕○○ Low	-0.059114256	⊕⊕○○ Low
149	Efpeglenatide_low_dosage:Inject_semaglutide_medium_dosage	0			0.183534988	⊕⊕○○ Low	0.183534988	⊕⊕○○ Low
150	Efpeglenatide_low_dosage:Liraglutide	0			-0.093447196	⊕⊕○○ Low	-0.093447196	⊕⊕○○ Low
151	Efpeglenatide_low_dosage:Lixisenatide	0			-0.175657437	⊕⊕○○ Low	-0.175657437	⊕⊕○○ Low

152	Efpeglenatide_low_dosage:Oral_semaglutide	0			-0.23278058	⊕⊕○○ Low	-0.23278058	⊕⊕○○ Low
153	Efpeglenatide_low_dosage:Placebo_or_Control	1	-0.159025994	⊕⊕⊕⊕ High	-0.267626007	⊕⊕⊕⊕ High	-0.188644632	⊕⊕⊕⊕ High
154	Efpeglenatide_low_dosage:Sotagliflozin	0			-0.014581974	⊕⊕○○ Low	-0.014581974	⊕⊕○○ Low
155	Efpeglenatide_low_dosage:Tirzepatide_high_dosage	0			0.287967889	⊕⊕○○ Low	0.287967889	⊕⊕○○ Low
156	Efpeglenatide_low_dosage:Tirzepatide_low_dosage	0			0.391980441	⊕⊕○○ Low	0.391980441	⊕⊕○○ Low
157	Efpeglenatide_medium_dosage:Empagliflozin_high_dosage	0			0.028304699	⊕⊕○○ Low	0.028304699	⊕⊕○○ Low
158	Efpeglenatide_medium_dosage:Empagliflozin_low_dosage	0			0.088306448	⊕⊕○○ Low	0.088306448	⊕⊕○○ Low
159	Efpeglenatide_medium_dosage:Ertugliflozin_high_dosage	0			-0.049725929	⊕⊕○○ Low	-0.049725929	⊕⊕○○ Low
160	Efpeglenatide_medium_dosage:Ertugliflozin_low_dosage	0			0.012146906	⊕⊕○○ Low	0.012146906	⊕⊕○○ Low
161	Efpeglenatide_medium_dosage:Exenatide	0			-0.17123522	⊕⊕○○ Low	-0.17123522	⊕⊕○○ Low
162	Efpeglenatide_medium_dosage:Inject_semaglutide_high_dosage	0			0.113166054	⊕⊕○○ Low	0.113166054	⊕⊕○○ Low
163	Efpeglenatide_medium_dosage:Inject_semaglutide_low_dosage	0			0.07676436	⊕⊕○○ Low	0.07676436	⊕⊕○○ Low
164	Efpeglenatide_medium_dosage:Inject_semaglutide_medium_dosage	0			0.319413604	⊕⊕○○ Low	0.319413604	⊕⊕○○ Low
165	Efpeglenatide_medium_dosage:Liraglutide	0			0.04243142	⊕⊕○○ Low	0.04243142	⊕⊕○○ Low
166	Efpeglenatide_medium_dosage:Lixisenatide	0			-0.039778821	⊕⊕○○ Low	-0.039778821	⊕⊕○○ Low
167	Efpeglenatide_medium_dosage:Oral_semaglutide	0			-0.096901964	⊕⊕○○ Low	-0.096901964	⊕⊕○○ Low
168	Efpeglenatide_medium_dosage:Placebo_or_Control	2	-0.002605732	⊕⊕⊕⊕ High	-0.205456605	⊕⊕⊕⊕ High	-0.052766016	⊕⊕⊕⊕ High
169	Efpeglenatide_medium_dosage:Sotagliflozin	0			0.121296642	⊕⊕○○ Low	0.121296642	⊕⊕○○ Low
170	Efpeglenatide_medium_dosage:Tirzepatide_high_dosage	0			0.423846505	⊕⊕○○ Low	0.423846505	⊕⊕○○ Low
171	Efpeglenatide_medium_dosage:Tirzepatide_low_dosage	0			0.527859057	⊕⊕○○ Low	0.527859057	⊕⊕○○ Low
172	Empagliflozin_high_dosage:Empagliflozin_low_dosage	3	0.108550922	⊕⊕⊕⊕ High	-0.012343759	⊕⊕⊕○ Medium	0.060001749	⊕⊕⊕⊕ High
173	Empagliflozin_high_dosage:Ertugliflozin_high_dosage	0			-0.078030628	⊕⊕○○ Low	-0.078030628	⊕⊕○○ Low

174	Empagliflozin_high_dosage:Ertugliflozin_low_dosage	0			-0.016157793	⊕⊕○○ Low	-0.016157793	⊕⊕○○ Low
175	Empagliflozin_high_dosage:Exenatide	0			-0.19953992	⊕⊕○○ Low	-0.19953992	⊕⊕○○ Low
176	Empagliflozin_high_dosage:Inject_semaglutide_high_dosage	0			0.084861355	⊕⊕○○ Low	0.084861355	⊕⊕○○ Low
177	Empagliflozin_high_dosage:Inject_semaglutide_low_dosage	0			0.048459661	⊕⊕○○ Low	0.048459661	⊕⊕○○ Low
178	Empagliflozin_high_dosage:Inject_semaglutide_medium_dosage	0			0.291108905	⊕⊕○○ Low	0.291108905	⊕⊕○○ Low
179	Empagliflozin_high_dosage:Liraglutide	0			0.014126721	⊕⊕○○ Low	0.014126721	⊕⊕○○ Low
180	Empagliflozin_high_dosage:Lixisenatide	0			-0.06808352	⊕⊕○○ Low	-0.06808352	⊕⊕○○ Low
181	Empagliflozin_high_dosage:Oral_semaglutide	1	0.770638797	⊕⊕⊕⊕ High	-0.552974263	⊕⊕⊕○ Medium	-0.125206663	⊕⊕⊕⊕ High
182	Empagliflozin_high_dosage:Placebo_or_Control	4	-0.18725899	⊕⊕⊕⊕ High	0.508671605	⊕⊕⊕○ Medium	-0.081070716	⊕⊕⊕⊕ High
183	Empagliflozin_high_dosage:Sotagliflozin	0			0.092991943	⊕⊕○○ Low	0.092991943	⊕⊕○○ Low
184	Empagliflozin_high_dosage:Tirzepatide_high_dosage	0			0.395541806	⊕⊕○○ Low	0.395541806	⊕⊕○○ Low
185	Empagliflozin_high_dosage:Tirzepatide_low_dosage	0			0.499554358	⊕⊕○○ Low	0.499554358	⊕⊕○○ Low
186	Empagliflozin_low_dosage:Ertugliflozin_high_dosage	0			-0.138032377	⊕⊕○○ Low	-0.138032377	⊕⊕○○ Low
187	Empagliflozin_low_dosage:Ertugliflozin_low_dosage	0			-0.076159542	⊕⊕○○ Low	-0.076159542	⊕⊕○○ Low
188	Empagliflozin_low_dosage:Exenatide	0			-0.259541669	⊕⊕○○ Low	-0.259541669	⊕⊕○○ Low
189	Empagliflozin_low_dosage:Inject_semaglutide_high_dosage	0			0.024859606	⊕⊕○○ Low	0.024859606	⊕⊕○○ Low
190	Empagliflozin_low_dosage:Inject_semaglutide_low_dosage	0			-0.011542088	⊕⊕○○ Low	-0.011542088	⊕⊕○○ Low
191	Empagliflozin_low_dosage:Inject_semaglutide_medium_dosage	0			0.231107156	⊕⊕○○ Low	0.231107156	⊕⊕○○ Low
192	Empagliflozin_low_dosage:Liraglutide	0			-0.045875028	⊕⊕○○ Low	-0.045875028	⊕⊕○○ Low
193	Empagliflozin_low_dosage:Lixisenatide	0			-0.128085269	⊕⊕○○ Low	-0.128085269	⊕⊕○○ Low
194	Empagliflozin_low_dosage:Oral_semaglutide	0			-0.185208412	⊕⊕○○ Low	-0.185208412	⊕⊕○○ Low
195	Empagliflozin_low_dosage:Placebo_or_Control	8	-0.14939361	⊕⊕⊕⊕ High	0.178465083	⊕⊕⊕○ Medium	-0.141072465	⊕⊕⊕⊕ High

196	Empagliflozin_low_dosage:Sotagliflozin	0			0.032990194	⊕⊕○○ Low	0.032990194	⊕⊕○○ Low
197	Empagliflozin_low_dosage:Tirzepatide_high_dosage	0			0.335540057	⊕⊕○○ Low	0.335540057	⊕⊕○○ Low
198	Empagliflozin_low_dosage:Tirzepatide_low_dosage	0			0.439552609	⊕⊕○○ Low	0.439552609	⊕⊕○○ Low
199	Ertugliflozin_high_dosage:Ertugliflozin_low_dosage	2	0.061406278	⊕⊕⊕⊕ High			0.061872835	⊕⊕⊕○ Medium
200	Ertugliflozin_high_dosage:Exenatide	0			-0.121509292	⊕⊕○○ Low	-0.121509292	⊕⊕○○ Low
201	Ertugliflozin_high_dosage:Inject_semaglutide_high_dosage	0			0.162891983	⊕⊕○○ Low	0.162891983	⊕⊕○○ Low
202	Ertugliflozin_high_dosage:Inject_semaglutide_low_dosage	0			0.126490289	⊕⊕○○ Low	0.126490289	⊕⊕○○ Low
203	Ertugliflozin_high_dosage:Inject_semaglutide_medium_dosage	0			0.369139533	⊕⊕○○ Low	0.369139533	⊕⊕○○ Low
204	Ertugliflozin_high_dosage:Liraglutide	0			0.092157349	⊕⊕○○ Low	0.092157349	⊕⊕○○ Low
205	Ertugliflozin_high_dosage:Lixisenatide	0			0.009947108	⊕⊕○○ Low	0.009947108	⊕⊕○○ Low
206	Ertugliflozin_high_dosage:Oral_semaglutide	0			-0.047176035	⊕⊕○○ Low	-0.047176035	⊕⊕○○ Low
207	Ertugliflozin_high_dosage:Placebo_or_Control	2	-0.00232661	⊕⊕⊕⊕ High			-0.003040088	⊕⊕⊕○ Medium
208	Ertugliflozin_high_dosage:Sotagliflozin	0			0.171022571	⊕⊕○○ Low	0.171022571	⊕⊕○○ Low
209	Ertugliflozin_high_dosage:Tirzepatide_high_dosage	0			0.473572434	⊕⊕○○ Low	0.473572434	⊕⊕○○ Low
210	Ertugliflozin_high_dosage:Tirzepatide_low_dosage	0			0.577584986	⊕⊕○○ Low	0.577584986	⊕⊕○○ Low
211	Ertugliflozin_low_dosage:Exenatide	0			-0.183382127	⊕⊕○○ Low	-0.183382127	⊕⊕○○ Low
212	Ertugliflozin_low_dosage:Inject_semaglutide_high_dosage	0			0.101019148	⊕⊕○○ Low	0.101019148	⊕⊕○○ Low
213	Ertugliflozin_low_dosage:Inject_semaglutide_low_dosage	0			0.064617454	⊕⊕○○ Low	0.064617454	⊕⊕○○ Low
214	Ertugliflozin_low_dosage:Inject_semaglutide_medium_dosage	0			0.307266698	⊕⊕○○ Low	0.307266698	⊕⊕○○ Low
215	Ertugliflozin_low_dosage:Liraglutide	0			0.030284514	⊕⊕○○ Low	0.030284514	⊕⊕○○ Low
216	Ertugliflozin_low_dosage:Lixisenatide	0			-0.051925727	⊕⊕○○ Low	-0.051925727	⊕⊕○○ Low
217	Ertugliflozin_low_dosage:Oral_semaglutide	0			-0.10904887	⊕⊕○○ Low	-0.10904887	⊕⊕○○ Low

218	Ertugliflozin_low_dosage:Placebo_or_Control	2	-0.063969597	⊕⊕⊕⊕ High			-0.064912923	⊕⊕⊕○ Medium
219	Ertugliflozin_low_dosage:Sotagliflozin	0			0.109149736	⊕⊕○○ Low	0.109149736	⊕⊕○○ Low
220	Ertugliflozin_low_dosage:Tirzepatide_high_dosage	0			0.411699599	⊕⊕○○ Low	0.411699599	⊕⊕○○ Low
221	Ertugliflozin_low_dosage:Tirzepatide_low_dosage	0			0.515712151	⊕⊕○○ Low	0.515712151	⊕⊕○○ Low
222	Exenatide:Inject_semaglutide_high_dosage	0			0.284401275	⊕⊕○○ Low	0.284401275	⊕⊕○○ Low
223	Exenatide:Inject_semaglutide_low_dosage	0			0.247999581	⊕⊕○○ Low	0.247999581	⊕⊕○○ Low
224	Exenatide:Inject_semaglutide_medium_dosage	0			0.490648825	⊕⊕○○ Low	0.490648825	⊕⊕○○ Low
225	Exenatide:Liraglutide	0			0.213666641	⊕⊕○○ Low	0.213666641	⊕⊕○○ Low
226	Exenatide:Lixisenatide	0			0.1314564	⊕⊕○○ Low	0.1314564	⊕⊕○○ Low
227	Exenatide:Oral_semaglutide	0			0.074333257	⊕⊕○○ Low	0.074333257	⊕⊕○○ Low
228	Exenatide:Placebo_or_Control	3	0.118469204	⊕⊕⊕⊕ High			0.118469204	⊕⊕⊕○ Medium
229	Exenatide:Sotagliflozin	0			0.292531862	⊕⊕○○ Low	0.292531862	⊕⊕○○ Low
230	Exenatide:Tirzepatide_high_dosage	0			0.595081726	⊕⊕○○ Low	0.595081726	⊕⊕○○ Low
231	Exenatide:Tirzepatide_low_dosage	0			0.699094277	⊕⊕○○ Low	0.699094277	⊕⊕○○ Low
232	Inject_semaglutide_high_dosage:Inject_semaglutide_low_dosage	0			-0.036401694	⊕⊕○○ Low	-0.036401694	⊕⊕○○ Low
233	Inject_semaglutide_high_dosage:Inject_semaglutide_medium_dosage	1	-0.002560821	⊕⊕⊕⊕ High	0.275191286	⊕⊕⊕○ Medium	0.20624755	⊕⊕⊕⊕ High
234	Inject_semaglutide_high_dosage:Liraglutide	1	-0.422272226	⊕⊕⊕⊕ High	-0.036631438	⊕⊕⊕⊕ High	-0.070734634	⊕⊕⊕⊕ High
235	Inject_semaglutide_high_dosage:Lixisenatide	0			-0.152944875	⊕⊕○○ Low	-0.152944875	⊕⊕○○ Low
236	Inject_semaglutide_high_dosage:Oral_semaglutide	0			-0.210068018	⊕⊕○○ Low	-0.210068018	⊕⊕○○ Low
237	Inject_semaglutide_high_dosage:Placebo_or_Control	4	-0.141664803	⊕⊕⊕⊕ High	-0.649214378	⊕⊕⊕⊕ High	-0.165932071	⊕⊕⊕⊕ High
238	Inject_semaglutide_high_dosage:Sotagliflozin	0			0.008130587	⊕⊕○○ Low	0.008130587	⊕⊕○○ Low
239	Inject_semaglutide_high_dosage:Tirzepatide_high_dosage	0			0.310680451	⊕⊕○○ Low	0.310680451	⊕⊕○○ Low

240	Inject_semaglutide_high_dosage:Tirzepatide_low_dosage	0			0.414693003	⊕⊕○○ Low	0.414693003	⊕⊕○○ Low
241	Inject_semaglutide_low_dosage:Inject_semaglutide_medium_dosage	2	0.319163512	⊕⊕⊕⊕ High	0.094449978	⊕⊕⊕⊕ High	0.242649244	⊕⊕⊕⊕ High
242	Inject_semaglutide_low_dosage:Liraglutide	1	-0.086644797	⊕⊕⊕⊕ High	-0.02071681	⊕⊕⊕⊕ High	-0.03433294	⊕⊕⊕⊕ High
243	Inject_semaglutide_low_dosage:Lixisenatide	0			-0.116543181	⊕⊕○○ Low	-0.116543181	⊕⊕○○ Low
244	Inject_semaglutide_low_dosage:Oral_semaglutide	0			-0.173666324	⊕⊕○○ Low	-0.173666324	⊕⊕○○ Low
245	Inject_semaglutide_low_dosage:Placebo_or_Control	3	-0.124015169	⊕⊕⊕⊕ High	-0.156432672	⊕⊕⊕⊕ High	-0.129530377	⊕⊕⊕⊕ High
246	Inject_semaglutide_low_dosage:Sotagliflozin	0			0.044532282	⊕⊕○○ Low	0.044532282	⊕⊕○○ Low
247	Inject_semaglutide_low_dosage:Tirzepatide_high_dosage	0			0.347082145	⊕⊕○○ Low	0.347082145	⊕⊕○○ Low
248	Inject_semaglutide_low_dosage:Tirzepatide_low_dosage	0			0.451094697	⊕⊕○○ Low	0.451094697	⊕⊕○○ Low
249	Inject_semaglutide_medium_dosage:Liraglutide	0			-0.276982184	⊕⊕○○ Low	-0.276982184	⊕⊕○○ Low
250	Inject_semaglutide_medium_dosage:Lixisenatide	0			-0.359192425	⊕⊕○○ Low	-0.359192425	⊕⊕○○ Low
251	Inject_semaglutide_medium_dosage:Oral_semaglutide	0			-0.416315568	⊕⊕○○ Low	-0.416315568	⊕⊕○○ Low
252	Inject_semaglutide_medium_dosage:Placebo_or_Control	3	-0.325313541	⊕⊕⊕⊕ High	-0.445060217	⊕⊕⊕⊕ High	-0.372179621	⊕⊕⊕⊕ High
253	Inject_semaglutide_medium_dosage:Sotagliflozin	0			-0.198116962	⊕⊕○○ Low	-0.198116962	⊕⊕○○ Low
254	Inject_semaglutide_medium_dosage:Tirzepatide_high_dosage	1	0.021672878	⊕⊕⊕⊕ High	0.164634375	⊕⊕⊕⊕ High	0.104432901	⊕⊕⊕⊕ High
255	Inject_semaglutide_medium_dosage:Tirzepatide_low_dosage	1	0.333769969	⊕⊕⊕⊕ High	0.095455873	⊕⊕⊕⊕ High	0.208445453	⊕⊕⊕⊕ High
256	Liraglutide:Lixisenatide	0			-0.082210241	⊕⊕○○ Low	-0.082210241	⊕⊕○○ Low
257	Liraglutide:Oral_semaglutide	0			-0.139333384	⊕⊕○○ Low	-0.139333384	⊕⊕○○ Low
258	Liraglutide:Placebo_or_Control	5	-0.14388472	⊕⊕⊕⊕ High	0.083740232	⊕⊕⊕○ Medium	-0.095197437	⊕⊕⊕⊕ High
259	Liraglutide:Sotagliflozin	0			0.078865221	⊕⊕○○ Low	0.078865221	⊕⊕○○ Low
260	Liraglutide:Tirzepatide_high_dosage	0			0.381415085	⊕⊕○○ Low	0.381415085	⊕⊕○○ Low
261	Liraglutide:Tirzepatide_low_dosage	0			0.485427637	⊕⊕○○ Low	0.485427637	⊕⊕○○ Low

262	Lixisenatide:Oral_semaglutide	0			-0.057123143	⊕⊕○○ Low	-0.057123143	⊕⊕○○ Low
263	Lixisenatide:Placebo_or_Control	1	-0.012987196	⊕⊕⊕⊕ High			-0.012987196	⊕⊕⊕○ Medium
264	Lixisenatide:Sotagliflozin	0			0.161075463	⊕⊕○○ Low	0.161075463	⊕⊕○○ Low
265	Lixisenatide:Tirzepatide_high_dosage	0			0.463625326	⊕⊕○○ Low	0.463625326	⊕⊕○○ Low
266	Lixisenatide:Tirzepatide_low_dosage	0			0.567637878	⊕⊕○○ Low	0.567637878	⊕⊕○○ Low
267	Oral_semaglutide:Placebo_or_Control	3	0.414196826	⊕⊕⊕⊕ High	-0.909416234	⊕⊕⊕○ Medium	0.044135947	⊕⊕⊕⊕ High
268	Oral_semaglutide:Sotagliflozin	0			0.218198605	⊕⊕○○ Low	0.218198605	⊕⊕○○ Low
269	Oral_semaglutide:Tirzepatide_high_dosage	0			0.520748469	⊕⊕○○ Low	0.520748469	⊕⊕○○ Low
270	Oral_semaglutide:Tirzepatide_low_dosage	0			0.624761021	⊕⊕○○ Low	0.624761021	⊕⊕○○ Low
271	Sotagliflozin:Placebo_or_Control	4	-0.174062658	⊕⊕⊕⊕ High			-0.174062658	⊕⊕⊕○ Medium
272	Tirzepatide_high_dosage:Placebo_or_Control	5	-0.520403781	⊕⊕⊕⊕ High	-0.265086067	⊕⊕⊕⊕ High	-0.476612522	⊕⊕⊕⊕ High
273	Tirzepatide_low_dosage:Placebo_or_Control	2	-0.582898769	⊕⊕⊕⊕ High	-0.578167011	⊕⊕⊕⊕ High	-0.580625073	⊕⊕⊕⊕ High
274	Sotagliflozin:Tirzepatide_high_dosage	0			0.302549863	⊕⊕○○ Low	0.302549863	⊕⊕○○ Low
275	Sotagliflozin:Tirzepatide_low_dosage	0			0.406562415	⊕⊕○○ Low	0.406562415	⊕⊕○○ Low
276	Tirzepatide_high_dosage:Tirzepatide_low_dosage	3	0.040574746	⊕⊕⊕⊕ High	0.575436073	⊕⊕⊕⊕ High	0.104012552	⊕⊕⊕⊕ High

Abbreviation: 95%CIs: 95% confidence intervals; GLP-1 agonist: glucagon-like peptide-1 agonist; NA: not applicable; NMA: network meta-analysis; OR: odds ratio; RCT: randomized controlled trial; SGLT2 inhibitor: sodium–glucose cotransporter 2 inhibitor

Table S9: Distribution of key clinical and design-related effect modifiers across treatment nodes to assess the plausibility of transitivity

Treatment node	No. of trials	No. of participants	Mean age (years)	Mean female proportion (%)	Mean study duration (weeks)	Trials including patients with renal impairment (%)	Trials including patients with diabetes mellitus (%)
Albiglutide	3	5625	62.8	33.2	79.1	0.0	100.0
Bexagliflozin	2	1289	65.0	31.3	48.6	50.0	100.0
Canagliflozin_high_dosage	4	4909	62.5	38.1	115.6	25.0	100.0
Canagliflozin_low_dosage	6	4586	61.9	36.4	110.4	50.0	83.3
Dapagliflozin	9	18591	63.5	36.6	144.6	11.1	44.4
Dulaglutide	5	5980	65.2	46.9	240.2	20.0	100.0
Efpeglenatide_high_dosage	3	1749	63.5	36.5	93.6	0.0	100.0
Efpeglenatide_low_dosage	1	102	58.5	46.1	62.0	0.0	100.0
Efpeglenatide_medium_dosage	3	1776	63.4	36.7	93.1	0.0	100.0
Empagliflozin_high_dosage	5	3992	61.0	35.2	109.4	60.0	100.0
Empagliflozin_low_dosage	8	11391	66.3	34.0	111.4	50.0	37.5
Ertugliflozin_high_dosage	2	2902	64.6	31.1	175.2	50.0	100.0
Ertugliflozin_low_dosage	2	2904	64.6	31.1	175.0	50.0	100.0
Exenatide	3	8102	61.4	38.7	157.8	0.0	100.0
Inject_semaglutide_high_dosage	4	9601	61.0	30.8	101.0	0.0	25.0
Inject_semaglutide_low_dosage	3	1906	28.3	50.3	72.5	0.0	66.7
Inject_semaglutide_medium_dosage	4	2053	33.6	46.1	71.4	0.0	100.0
Liraglutide	7	8811	57.2	50.4	126.9	0.0	57.1
Lixisenatide	1	176	74.2	48.0	26.0	0.0	100.0
Oral_semaglutide	4	3559	62.0	40.7	66.6	50.0	75.0
Placebo_or_Control	61	89121	59.1	35.7	126.9	26.2	67.2
Sotagliflozin	4	6948	66.8	44.3	97.4	50.0	100.0
Tirzepatide_high_dosage	6	2577	54.9	53.5	56.1	0.0	50.0
Tirzepatide_low_dosage	3	820	57.3	53.7	41.7	0.0	100.0

Reference list of supplement tables:

1. Page, M.J.; McKenzie, J.E.; Bossuyt, P.M.; Boutron, I.; Hoffmann, T.C.; Mulrow, C.D.; Shamseer, L.; Tetzlaff, J.M.; Akl, E.A.; Brennan, S.E.; et al. The PRISMA 2020 statement: an updated guideline for reporting systematic reviews. *Bmj* **2021**, *372*, n71, doi:10.1136/bmj.n71.
2. Jastreboff, A.M.; Aronne, L.J.; Ahmad, N.N.; Wharton, S.; Connery, L.; Alves, B.; Kiyosue, A.; Zhang, S.; Liu, B.; Bunck, M.C.; et al. Tirzepatide Once Weekly for the Treatment of Obesity. *N Engl J Med* **2022**, *387*, 205-216, doi:10.1056/NEJMoa2206038.
3. Del Prato, S.; Kahn, S.E.; Pavo, I.; Weerakkody, G.J.; Yang, Z.; Doupis, J.; Aizenberg, D.; Wynne, A.G.; Riesmeyer, J.S.; Heine, R.J.; et al. Tirzepatide versus insulin glargine in type 2 diabetes and increased cardiovascular risk (SURPASS-4): a randomised, open-label, parallel-group, multicentre, phase 3 trial. *Lancet* **2021**, *398*, 1811-1824, doi:10.1016/S0140-6736(21)02188-7.
4. Ludvik, B.; Giorgino, F.; Jodar, E.; Frias, J.P.; Fernandez Lando, L.; Brown, K.; Bray, R.; Rodriguez, A. Once-weekly tirzepatide versus once-daily insulin degludec as add-on to metformin with or without SGLT2 inhibitors in patients with type 2 diabetes (SURPASS-3): a randomised, open-label, parallel-group, phase 3 trial. *Lancet* **2021**, *398*, 583-598, doi:10.1016/S0140-6736(21)01443-4.
5. Hulst, A.H.; Ow, C.P.C.; May, C.N.; Hood, S.H.; Plummer, M.P.; Hermanides, J.; van Raalte, D.H.; Deane, A.M.; Bellomo, R.; Lankadeva, Y.R. Effects of sodium-glucose transporter-2 inhibition on systemic hemodynamics, renal function, and intra-renal oxygenation in sepsis-associated acute kidney injury. *Intensive Care Med Exp* **2024**, *12*, 64, doi:10.1186/s40635-024-00647-2.
6. Aleman Espino, A.; Aleman Espino, E.; Aleman Oliva, C.; Monteagudo, H.; Frontela, O. An Incidental Finding of a Glucagon-Like Peptide 1 (GLP-1)-Induced Acute Kidney Injury: A Case Report. *Cureus* **2023**, *15*, e45261, doi:10.7759/cureus.45261.
7. Ryan, R.; Choo, S.; Willows, J.; Walker, J.; Prasad, K.; Tez, D. Acute interstitial nephritis due to sodium-glucose co-transporter 2 inhibitor empagliflozin. *Clin Kidney J* **2021**, *14*, 1020-1022, doi:10.1093/ckj/sfaa033.
8. Farhat, F.; Gonzalez, P.; Ravin, A.B.; Foo, F.F.; Patel, S.; Cheng, J.; Holland, S.W.; Chinnici, A.; Abu Homoud, A. 7529 A Silent Menace Of Mounjaro: Acute Renal Failure Associated With Tirzepatide. *Journal of the Endocrine Society* **2024**, *8*, bvae163.1027, doi:10.1210/jendso/bvae163.1027.

9. Gomez, H.; Derde, L.P.G. Sodium-Glucose Cotransporter 2 Therapy for Acute Organ Dysfunction in Critically Ill Patients. *Jama* **2024**, *332*, 377-379, doi:10.1001/jama.2024.10171.
10. Zhang, Z.; Heerspink, H.J.L.; Chertow, G.M.; Correa-Rotter, R.; Gasparrini, A.; Jongs, N.; Langkilde, A.M.; McMurray, J.J.V.; Mistry, M.N.; Rossing, P.; et al. Ambient heat exposure and kidney function in patients with chronic kidney disease: a post-hoc analysis of the DAPA-CKD trial. *Lancet Planet Health* **2024**, *8*, e225-e233, doi:10.1016/S2542-5196(24)00026-3.
11. Jongs, N.; Chertow, G.M.; Greene, T.; McMurray, J.J.V.; Langkilde, A.M.; Correa-Rotter, R.; Kashihara, N.; Rossing, P.; Sjostrom, C.D.; Stefansson, B.V.; et al. Correlates and Consequences of an Acute Change in eGFR in Response to the SGLT2 Inhibitor Dapagliflozin in Patients with CKD. *J Am Soc Nephrol* **2022**, *33*, 2094-2107, doi:10.1681/ASN.2022030306.
12. Heerspink, H.J.L.; Furtado, R.H.M.; Berwanger, O.; Koch, G.G.; Martinez, F.; Mukhtar, O.; Verma, S.; Gasparyan, S.B.; Tang, F.; Windsor, S.L.; et al. Dapagliflozin and Kidney Outcomes in Hospitalized Patients with COVID-19 Infection: An Analysis of the DARE-19 Randomized Controlled Trial. *Clin J Am Soc Nephrol* **2022**, *17*, 643-654, doi:10.2215/CJN.14231021.
13. Heerspink, H.J.L.; Oshima, M.; Zhang, H.; Li, J.; Agarwal, R.; Capuano, G.; Charytan, D.M.; Craig, J.; de Zeeuw, D.; Di Tanna, G.L.; et al. Canagliflozin and Kidney-Related Adverse Events in Type 2 Diabetes and CKD: Findings From the Randomized CREDENCE Trial. *Am J Kidney Dis* **2022**, *79*, 244-256 e241, doi:10.1053/j.ajkd.2021.05.005.
14. Leiter, L.A.; Cefalu, W.T.; de Bruin, T.W.; Xu, J.; Parikh, S.; Johnsson, E.; Gause-Nilsson, I. Long-term maintenance of efficacy of dapagliflozin in patients with type 2 diabetes mellitus and cardiovascular disease. *Diabetes Obes Metab* **2016**, *18*, 766-774, doi:10.1111/dom.12666.
15. Hosseini, Z.S.; Jamili, M.J.; Ensan, B.; Donyadideh, G.; Shahri, B.; Eshraghi, H.; Darroudi, S.; Moohebati, M. Short-term effects of empagliflozin on preventing contrast induced acute kidney injury in patients undergoing percutaneous coronary intervention, a randomised trial. *Scientific reports* **2025**, *15*, 3940, doi:10.1038/s41598-024-82991-7.
16. Bai, Y.; Jin, J.; Zhou, W.; Zhang, S.; Xu, J. The safety outcomes of sodium-glucose cotransporter 2 inhibitors in patients with different renal function: A systematic review and meta-analysis. *Nutr Metab Cardiovasc Dis* **2021**, *31*, 1365-1374, doi:10.1016/j.numecd.2021.02.006.

17. Chewcharat, A.; Prasitlumkum, N.; Thongprayoon, C.; Bathini, T.; Medaura, J.; Vallabhajosyula, S.; Cheungpasitporn, W. Efficacy and Safety of SGLT-2 Inhibitors for Treatment of Diabetes Mellitus among Kidney Transplant Patients: A Systematic Review and Meta-Analysis. *Med Sci (Basel)* **2020**, *8*, doi:10.3390/medsci8040047.
18. Kumar, K.; Kheiri, B.; Simpson, T.F.; Osman, M.; Rahmouni, H. Sodium-Glucose Cotransporter-2 Inhibitors in Heart Failure: A Meta-Analysis of Randomized Clinical Trials. *The American journal of medicine* **2020**, *133*, e625-e630, doi:10.1016/j.amjmed.2020.04.006.
19. Zhang, X.; Zhong, Z.; Li, Y.; Li, W. Long-term renal outcomes associated with sodium glucose cotransporter 2 inhibitors in patients with type 2 diabetes mellitus: A systematic review and meta-analysis. *Diabetes/metabolism research and reviews* **2020**, *36*, e3303, doi:10.1002/dmrr.3303.
20. Menne, J.; Dumann, E.; Haller, H.; Schmidt, B.M.W. Acute kidney injury and adverse renal events in patients receiving SGLT2-inhibitors: A systematic review and meta-analysis. *PLoS medicine* **2019**, *16*, e1002983, doi:10.1371/journal.pmed.1002983.
21. Neuen, B.L.; Young, T.; Heerspink, H.J.L.; Neal, B.; Perkovic, V.; Billot, L.; Mahaffey, K.W.; Charytan, D.M.; Wheeler, D.C.; Arnott, C.; et al. SGLT2 inhibitors for the prevention of kidney failure in patients with type 2 diabetes: a systematic review and meta-analysis. *Lancet Diabetes Endocrinol* **2019**, *7*, 845-854, doi:10.1016/S2213-8587(19)30256-6.
22. Gilbert, R.E.; Thorpe, K.E. Acute kidney injury with sodium-glucose co-transporter-2 inhibitors: A meta-analysis of cardiovascular outcome trials. *Diabetes Obes Metab* **2019**, *21*, 1996-2000, doi:10.1111/dom.13754.
23. Donnan, J.R.; Grandy, C.A.; Chibrikov, E.; Marra, C.A.; Aubrey-Bassler, K.; Johnston, K.; Swab, M.; Hache, J.; Curnew, D.; Nguyen, H.; et al. Comparative safety of the sodium glucose co-transporter 2 (SGLT2) inhibitors: a systematic review and meta-analysis. *BMJ Open* **2019**, *9*, e022577, doi:10.1136/bmjopen-2018-022577.
24. Zhang, X.L.; Zhu, Q.Q.; Chen, Y.H.; Li, X.L.; Chen, F.; Huang, J.A.; Xu, B. Cardiovascular Safety, Long-Term Noncardiovascular Safety, and Efficacy of Sodium-Glucose Cotransporter 2 Inhibitors in Patients With Type 2 Diabetes Mellitus: A Systemic Review and Meta-Analysis With Trial Sequential Analysis. *J Am Heart Assoc* **2018**, *7*, doi:10.1161/JAHA.117.007165.
25. Huang, M.; Wei, R.; Wang, Y.; Su, T.; Li, Q.; Yang, X.; Chen, X. Protective effect of glucagon-like peptide-1 agents on reperfusion injury for acute myocardial infarction: a meta-analysis of randomized controlled trials. *Ann Med* **2017**, *49*, 552-561,

doi:10.1080/07853890.2017.1306653.
26. Cao, H.; Rao, X.; Jia, J.; Yan, T.; Li, D. Effects of sodium-glucose co-transporter-2 inhibitors on kidney, cardiovascular, and safety outcomes in patients with advanced chronic kidney disease: a systematic review and meta-analysis of randomized controlled trials. *Acta Diabetol* **2023**, *60*, 325-335, doi:10.1007/s00592-022-01989-7.
27. Gong, C.; Shen, S.C.; Zhang, K.; Zhou, L.; Shen, J.J.; Zhao, J.Y.; Ding, S.G.; Ma, L.K.; Gao, H. Association of sodium-glucose cotransporter 2 inhibitors with cardiovascular outcome and safety events: A meta-analysis of randomized controlled clinical trials. *Front Cardiovasc Med* **2022**, *9*, 926979, doi:10.3389/fcvm.2022.926979.
28. Kaze, A.D.; Zhuo, M.; Kim, S.C.; Patorno, E.; Paik, J.M. Association of SGLT2 inhibitors with cardiovascular, kidney, and safety outcomes among patients with diabetic kidney disease: a meta-analysis. *Cardiovasc Diabetol* **2022**, *21*, 47, doi:10.1186/s12933-022-01476-x.
29. Lin, D.S.; Lee, J.K.; Chen, W.J. Clinical Adverse Events Associated with Sodium-Glucose Cotransporter 2 Inhibitors: A Meta-Analysis Involving 10 Randomized Clinical Trials and 71 553 Individuals. *J Clin Endocrinol Metab* **2021**, *106*, 2133-2145, doi:10.1210/clinem/dgab274.
30. Nuffield Department of Population Health Renal Studies, G.; Consortium, S.i.M.-A.C.-R.T. Impact of diabetes on the effects of sodium glucose co-transporter-2 inhibitors on kidney outcomes: collaborative meta-analysis of large placebo-controlled trials. *Lancet* **2022**, *400*, 1788-1801, doi:10.1016/S0140-6736(22)02074-8.
31. Qiu, M.; Ding, L.L.; Zhang, M.; Zhou, H.R. Safety of four SGLT2 inhibitors in three chronic diseases: A meta-analysis of large randomized trials of SGLT2 inhibitors. *Diab Vasc Dis Res* **2021**, *18*, 14791641211011016, doi:10.1177/14791641211011016.
32. Salah, H.M.; Al'Aref, S.J.; Khan, M.S.; Al-Hawwas, M.; Vallurupalli, S.; Mehta, J.L.; Mounsey, J.P.; Greene, S.J.; McGuire, D.K.; Lopes, R.D.; et al. Efficacy and safety of sodium-glucose cotransporter 2 inhibitors initiation in patients with acute heart failure, with and without type 2 diabetes: a systematic review and meta-analysis. *Cardiovasc Diabetol* **2022**, *21*, 20, doi:10.1186/s12933-022-01455-2.
33. Tsai, W.C.; Hsu, S.P.; Chiu, Y.L.; Yang, J.Y.; Pai, M.F.; Ko, M.J.; Tu, Y.K.; Hung, K.Y.; Chien, K.L.; Peng, Y.S.; et al. Cardiovascular and renal efficacy and safety of sodium-glucose cotransporter-2 inhibitors in patients without diabetes: a systematic review and meta-analysis of randomised placebo-controlled trials. *BMJ Open* **2022**, *12*, e060655, doi:10.1136/bmjopen-2021-060655.

34. Vukadinovic, D.; Abdin, A.; Anker, S.D.; Rosano, G.M.C.; Mahfoud, F.; Packer, M.; Butler, J.; Bohm, M. Side effects and treatment initiation barriers of sodium-glucose cotransporter 2 inhibitors in heart failure: a systematic review and meta-analysis. *Eur J Heart Fail* **2022**, *24*, 1625-1632, doi:10.1002/ejhf.2584.
35. Zheng, C.; Lin, M.; Chen, Y.; Xu, H.; Yan, L.; Dai, H. Effects of sodium-glucose cotransporter type 2 inhibitors on cardiovascular, renal, and safety outcomes in patients with cardiovascular disease: a meta-analysis of randomized controlled trials. *Cardiovasc Diabetol* **2021**, *20*, 83, doi:10.1186/s12933-021-01272-z.
36. Taheri, S. Heterogeneity in cardiorenal protection by Sodium glucose cotransporter 2 inhibitors in heart failure across the ejection fraction strata: Systematic review and meta-analysis. *World J Nephrol* **2023**, *12*, 182-200, doi:10.5527/wjn.v12.i5.182.
37. Li, L.; Dong, Y.H.; Bai, Y.; Tang, Z.Y.; Deng, Y.M.; Wu, Z.; Li, W.Y. Association of SGLT2is with cardiovascular and reproductive diseases: a meta-analysis based on 14 large-scale randomized trials. *Endocrine* **2024**, *84*, 836-841, doi:10.1007/s12020-023-03618-x.
38. Pozzi, A.; Cirelli, C.; Merlo, A.; Rea, F.; Scangiuzzi, C.; Tavano, E.; Iorio, A.; Kristensen, S.L.; Wong, C.; Iacovoni, A.; et al. Adverse effects of sodium-glucose cotransporter-2 inhibitors in patients with heart failure: a systematic review and meta-analysis. *Heart Fail Rev* **2024**, *29*, 207-217, doi:10.1007/s10741-023-10363-w.
39. Zhou, T.; Yao, K.; Xie, Y.; Lin, Y.; Wang, J.; Chen, X. Renal Protection and Safety of Sodium-glucose Cotransporter-2 Inhibitors in Chronic Kidney Disease. *Curr Pharm Des* **2023**, *29*, 1659-1670, doi:10.2174/1381612829666230804103643.
40. Rigato, M.; Fadini, G.P.; Avogaro, A. Safety of sodium-glucose cotransporter 2 inhibitors in elderly patients with type 2 diabetes: A meta-analysis of randomized controlled trials. *Diabetes Obes Metab* **2023**, *25*, 2963-2969, doi:10.1111/dom.15193.
41. Bose, D.; Maurya, M.; Konwar, M. Impact of sodium-glucose co-transporter 2 inhibitors on renal outcomes in patients of diabetes mellitus: A meta-analysis of landmark renal and cardiovascular outcome trials. *Indian journal of pharmacology* **2023**, *55*, 119-127, doi:10.4103/ijp.ijp_342_21.
42. Liu, Y.; An, C.; Liu, P.; Yang, F.; Zhao, Q. Comparative safety of sodium-glucose co-transporter 2 inhibitors in elderly patients with type 2 diabetes mellitus and diabetic kidney disease: a systematic review and meta-analysis. *Renal failure* **2023**, *45*, 2217287, doi:10.1080/0886022X.2023.2217287.

43. Chen, H.B.; Meng, R.S.; Yang, Y.L.; Yu, T.H. The risk of all-cause death with dapagliflozin versus placebo: a systematic review and meta-analysis of phase III randomized controlled trials. *Expert opinion on drug safety* **2023**, *22*, 133-140, doi:10.1080/14740338.2023.2182290.
44. Carvalho, P.E.P.; Veiga, T.M.A.; Simoes, E.S.A.C.; Gewehr, D.M.; Dagostin, C.S.; Fernandes, A.; Nasi, G.; Cardoso, R. Cardiovascular and renal effects of SGLT2 inhibitor initiation in acute heart failure: a meta-analysis of randomized controlled trials. *Clin Res Cardiol* **2023**, *112*, 1044-1055, doi:10.1007/s00392-022-02148-2.
45. Jamil, S.; Zainab, A.; Arora, A.; Shaik, T.A.; Khemani, V.; Mekowulu, F.C.; Aschalew, Y.N.; Khan, S. Efficacy and Safety of Sodium Glucose Cotransporter-2 (SGLT2) Inhibitors in Patients With Diabetes and Chronic Kidney Disease (CKD): A Meta-analysis of Randomized Control Trials. *Cureus* **2022**, *14*, e31898, doi:10.7759/cureus.31898.
46. Aldafas, R.; Crabtree, T.; Alkharaiji, M.; Vinogradova, Y.; Idris, I. Sodium-glucose cotransporter-2 inhibitors (SGLT2) in frail or older people with type 2 diabetes and heart failure: a systematic review and meta-analysis. *Age and ageing* **2024**, *53*, doi:10.1093/ageing/afad254.
47. Dimitriadis, K.; Vakka, A.; Pyrpyris, N.; Apostolos, A.; Beneki, E.; Stathopoulou, E.; Giannou, P.; Tsioufis, P.; Iliakis, P.; Aznaouridis, K.; et al. Efficacy of Chronic Use of Sodium-Glucose Co-transporter 2 Inhibitors on the Prevention of Contrast-Induced Acute Kidney Injury in Patients with Type 2 Diabetes Mellitus Following Coronary Procedures: A Systematic Review and Meta-Analysis. *Am J Cardiovasc Drugs* **2025**, *25*, 57-69, doi:10.1007/s40256-024-00684-y.
48. Heerspink, H.J.L.; Eddington, D.; Chaudhari, J.; Estacio, R.; Imai, E.; Goicoechea, M.; Hannedouche, T.; Haynes, R.; Jafar, T.H.; Johnson, D.W.; et al. A meta-analysis of randomized controlled clinical trials for implications of acute treatment effects on glomerular filtration rate for long-term kidney protection. *Kidney Int* **2024**, *106*, 688-698, doi:10.1016/j.kint.2024.05.024.
49. Hou, J.; Ren, L.; Hou, Q.; Jia, X.; Mei, Z.; Xu, J.; Yang, Z.; Li, Y.; Yan, C. Efficacy and safety of sodium-glucose cotransporter 2 (SGLT2) inhibitors in patients with acute heart failure: a systematic review and meta-analysis. *Front Cardiovasc Med* **2024**, *11*, 1388337, doi:10.3389/fcvm.2024.1388337.
50. Patel, S.M.; Kang, Y.M.; Im, K.; Neuen, B.L.; Anker, S.D.; Bhatt, D.L.; Butler, J.; Cherney, D.Z.I.; Claggett, B.L.; Fletcher, R.A.; et al. Sodium-Glucose Cotransporter-2 Inhibitors and Major Adverse Cardiovascular Outcomes: A SMART-C Collaborative Meta-Analysis. *Circulation*

2024, *149*, 1789-1801, doi:10.1161/CIRCULATIONAHA.124.069568.

51. Usman, M.S.; Bhatt, D.L.; Hameed, I.; Anker, S.D.; Cheng, A.Y.Y.; Hernandez, A.F.; Jones, W.S.; Khan, M.S.; Petrie, M.C.; Udell, J.A.; et al. Effect of SGLT2 inhibitors on heart failure outcomes and cardiovascular death across the cardiometabolic disease spectrum: a systematic review and meta-analysis. *Lancet Diabetes Endocrinol* **2024**, *12*, 447-461, doi:10.1016/S2213-8587(24)00102-5.
52. Wang, Q.; Yu, J.; Deng, W.; Liu, C.; Yang, J.; Li, Y.; Cai, G.; Chen, X.; Dong, Z. Influence of sodium/glucose cotransporter-2 inhibitors on the incidence of acute kidney injury: a meta-analysis. *Front Pharmacol* **2024**, *15*, 1372421, doi:10.3389/fphar.2024.1372421.
53. Wang, X.; He, M.; Jin, D.; Sun, C.; Lu, H. Effect of SGLT-2 inhibitors on acute kidney injury in patients with heart failure: a systematic review and meta-analysis. *Diabetol Metab Syndr* **2024**, *16*, 207, doi:10.1186/s13098-024-01446-1.
54. Wanner, C.; Iliev, H.; Duarte, N.; Schueler, E.; Soares, A.R.; Thanam, V.; Pfarr, E. Safety of Empagliflozin: An Individual Participant-Level Data Meta-Analysis from Four Large Trials. *Adv Ther* **2024**, *41*, 2826-2844, doi:10.1007/s12325-024-02879-w.
55. Zhang, Y.; Luo, J.; Li, B.; Xu, J.; Yu, H.; Chen, N. Cardio-renal protective effect and safety of sodium-glucose cotransporter 2 inhibitors for chronic kidney disease patients with eGFR < 60 mL/min/1.73 m2: a systematic review and meta-analysis. *BMC nephrology* **2024**, *25*, 392, doi:10.1186/s12882-024-03833-2.
56. Ansari, H.U.H.; Samad, M.A.; Mahboob, E.; Zulfiqar, E.; Qazi, S.U.; Ahsan, A.; Ahmed, M.; Ahmed, F.; Ahmed, R.; Ali, S.; et al. Sodium-glucose cotransporter 2 inhibitors in patients with type 2 diabetes and myocardial infarction undergoing percutaneous coronary intervention: A systematic review and meta-analysis. *Am J Prev Cardiol* **2025**, *21*, 100927, doi:10.1016/j.ajpc.2024.100927.
57. Badve, S.V.; Bilal, A.; Lee, M.M.Y.; Sattar, N.; Gerstein, H.C.; Ruff, C.T.; McMurray, J.J.V.; Rossing, P.; Bakris, G.; Mahaffey, K.W.; et al. Effects of GLP-1 receptor agonists on kidney and cardiovascular disease outcomes: a meta-analysis of randomised controlled trials. *Lancet Diabetes Endocrinol* **2025**, *13*, 15-28, doi:10.1016/S2213-8587(24)00271-7.
58. Gao, F.M.; Ali, A.S.; Bellomo, R.; Gaca, M.; Lecamwasam, A.; Churilov, L.; Ekinci, E.I. A Systematic Review and Meta-analysis on the Safety and Efficacy of Sodium-Glucose Cotransporter 2 Inhibitor Use in Hospitalized Patients. *Diabetes Care* **2024**, *47*, 2275-2290, doi:10.2337/dc24-0946.
59. Chen, L.; Xue, Q.; Yan, C.; Tang, B.; Wang, L.; Zhang, B.; Zhao, Q. Comparative safety of different recommended doses of sodium-glucose

cotransporter 2 inhibitors in patients with type 2 diabetes mellitus: a systematic review and network meta-analysis of randomized clinical trials. *Front Endocrinol (Lausanne)* **2023**, *14*, 1256548, doi:10.3389/fendo.2023.1256548.
60. Kani, R.; Watanabe, A.; Miyamoto, Y.; Ejiri, K.; Iwagami, M.; Takagi, H.; Slipczuk, L.; Tsugawa, Y.; Aikawa, T.; Kuno, T. Comparison of Effectiveness Among Different Sodium-Glucose Cotransoporter-2 Inhibitors According to Underlying Conditions: A Network Meta-Analysis of Randomized Controlled Trials. *J Am Heart Assoc* **2024**, *13*, e031805, doi:10.1161/JAHA.123.031805.
61. Li, C.X.; Liu, L.Y.; Zhang, C.X.; Geng, X.H.; Gu, S.M.; Wang, Y.Q.; Liu, H.; Xie, Q.; Liang, S. Comparative safety of different sodium-glucose transporter 2 inhibitors in patients with type 2 diabetes: a systematic review and network meta-analysis of randomized controlled trials. *Front Endocrinol (Lausanne)* **2023**, *14*, 1238399, doi:10.3389/fendo.2023.1238399.
62. Sim, R.; Chong, C.W.; Loganadan, N.K.; Fong, A.Y.Y.; Navaravong, L.; Hussein, Z.; Khunti, K.; Lee, S.W.H. Comparative effectiveness of cardiovascular, renal and safety outcomes of second-line antidiabetic drugs use in people with type 2 diabetes: A systematic review and network meta-analysis of randomised controlled trials. *Diabet Med* **2022**, *39*, e14780, doi:10.1111/dme.14780.
63. Tang, H.; Li, D.; Zhang, J.; Li, Y.; Wang, T.; Zhai, S.; Song, Y. Sodium-glucose co-transporter-2 inhibitors and risk of adverse renal outcomes among patients with type 2 diabetes: A network and cumulative meta-analysis of randomized controlled trials. *Diabetes Obes Metab* **2017**, *19*, 1106-1115, doi:10.1111/dom.12917.
64. Yang, S.; He, W.; Zhao, L.; Mi, Y. Association between use of sodium-glucose cotransporter 2 inhibitors, glucagon-like peptide 1 agonists, and dipeptidyl peptidase 4 inhibitors with kidney outcomes in patients with type 2 diabetes: A systematic review and network meta-analysis. *PloS one* **2022**, *17*, e0267025, doi:10.1371/journal.pone.0267025.
65. Yang, S.; Zhao, L.; Mi, Y.; He, W. Effects of sodium-glucose cotransporter-2 inhibitors and aldosterone antagonists, in addition to renin-angiotensin system antagonists, on major adverse kidney outcomes in patients with type 2 diabetes and chronic kidney disease: A systematic review and network meta-analysis. *Diabetes Obes Metab* **2022**, *24*, 2159-2168, doi:10.1111/dom.14801.
66. Yang, S.Q.; Zhao, X.; Zhang, J.; Liu, H.; Wang, Y.H.; Wang, Y.G. Comparative efficacy and safety of SGLT2is and ns-MRAs in patients with diabetic kidney disease: a systematic review and network meta-analysis. *Front Endocrinol (Lausanne)* **2024**, *15*, 1429261, doi:10.3389/fendo.2024.1429261.

67. Bonnesen, K.; Heide-Jorgensen, U.; Christensen, D.H.; Christiansen, C.F.; Lash, T.L.; Hennessy, S.; Matthews, A.A.; Pedersen, L.; Thomsen, R.W.; Schmidt, M. Effectiveness of Empagliflozin vs Dapagliflozin for Kidney Outcomes in Type 2 Diabetes. *JAMA Intern Med* **2025**, doi:10.1001/jamainternmed.2024.7381.
68. Yang, H.; Yang, L.; Jardine, M.J.; Arnott, C.; Neuen, B.L.; Xu, K.; Zhao, X.; Qian, D.; Cui, B.; Qiu, Y.; et al. The association between sodium-glucose cotransporter 2 inhibitors and contrast-associated acute kidney injury in patients with type 2 diabetes undergoing angiography: a propensity-matched study. *Eur J Med Res* **2024**, *29*, 621, doi:10.1186/s40001-024-02214-7.
69. Pillai, A.; Riaz, S.; Arora, S.; Jaiswal, A. The Role of Sodium-Glucose Cotransporter-2 Inhibitors in Adults With Transthyretin Cardiac Amyloidosis: A Single-Center Retrospective Cohort Study. *Cureus* **2024**, *16*, e72725, doi:10.7759/cureus.72725.
70. Alkabbani, W.; Zongo, A.; Minhas-Sandhu, J.K.; Eurich, D.T.; Shah, B.R.; Alsabbagh, M.W.; Gamble, J.M. Renal effectiveness and safety of the sodium-glucose cotransporter-2 inhibitors: a population-based cohort study. *BMJ Open Diabetes Res Care* **2021**, *9*, doi:10.1136/bmjdrc-2021-002496.
71. Patorno, E.; Pawar, A.; Bessette, L.G.; Kim, D.H.; Dave, C.; Glynn, R.J.; Munshi, M.N.; Schneeweiss, S.; Wexler, D.J.; Kim, S.C. Comparative Effectiveness and Safety of Sodium-Glucose Cotransporter 2 Inhibitors Versus Glucagon-Like Peptide 1 Receptor Agonists in Older Adults. *Diabetes Care* **2021**, *44*, 826-835, doi:10.2337/dc20-1464.
72. Umpierrez, G.; Tofe Povedano, S.; Perez Manghi, F.; Shurzinske, L.; Pechtner, V. Efficacy and safety of dulaglutide monotherapy versus metformin in type 2 diabetes in a randomized controlled trial (AWARD-3). *Diabetes Care* **2014**, *37*, 2168-2176, doi:10.2337/dc13-2759.
73. Roden, M.; Weng, J.; Eilbracht, J.; Delafont, B.; Kim, G.; Woerle, H.J.; Broedl, U.C.; investigators, E.-R.M.t. Empagliflozin monotherapy with sitagliptin as an active comparator in patients with type 2 diabetes: a randomised, double-blind, placebo-controlled, phase 3 trial. *Lancet Diabetes Endocrinol* **2013**, *1*, 208-219, doi:10.1016/S2213-8587(13)70084-6.
74. Polidori, D.; Mari, A.; Ferrannini, E. Canagliflozin, a sodium glucose co-transporter 2 inhibitor, improves model-based indices of beta cell function in patients with type 2 diabetes. *Diabetologia* **2014**, *57*, 891-901, doi:10.1007/s00125-014-3196-x.
75. Ferrannini, E.; Berk, A.; Hantel, S.; Pinnetti, S.; Hach, T.; Woerle, H.J.; Broedl, U.C. Long-term safety and efficacy of empagliflozin, sitagliptin, and metformin: an active-controlled, parallel-group, randomized, 78-week open-label extension study in patients with type 2

diabetes. *Diabetes Care* **2013**, *36*, 4015-4021, doi:10.2337/dc13-0663.
76. Rosenstock, J.; Raccah, D.; Koranyi, L.; Maffei, L.; Boka, G.; Miossec, P.; Gerich, J.E. Efficacy and safety of lixisenatide once daily versus exenatide twice daily in type 2 diabetes inadequately controlled on metformin: a 24-week, randomized, open-label, active-controlled study (GetGoal-X). *Diabetes Care* **2013**, *36*, 2945-2951, doi:10.2337/dc12-2709.
77. Charbonnel, B.; Steinberg, H.; Eymard, E.; Xu, L.; Thakkar, P.; Prabhu, V.; Davies, M.J.; Engel, S.S. Efficacy and safety over 26 weeks of an oral treatment strategy including sitagliptin compared with an injectable treatment strategy with liraglutide in patients with type 2 diabetes mellitus inadequately controlled on metformin: a randomised clinical trial. *Diabetologia* **2013**, *56*, 1503-1511, doi:10.1007/s00125-013-2905-1.
78. Schernthaner, G.; Gross, J.L.; Rosenstock, J.; Guarisco, M.; Fu, M.; Yee, J.; Kawaguchi, M.; Canovatchel, W.; Meininger, G. Canagliflozin compared with sitagliptin for patients with type 2 diabetes who do not have adequate glycemic control with metformin plus sulfonylurea: a 52-week randomized trial. *Diabetes Care* **2013**, *36*, 2508-2515, doi:10.2337/dc12-2491.
79. Wilding, J.P.; Woo, V.; Soler, N.G.; Pahor, A.; Sugg, J.; Rohwedder, K.; Parikh, S.; Dapagliflozin 006 Study, G. Long-term efficacy of dapagliflozin in patients with type 2 diabetes mellitus receiving high doses of insulin: a randomized trial. *Ann Intern Med* **2012**, *156*, 405-415, doi:10.7326/0003-4819-156-6-201203200-00003.
80. Buse, J.B.; Rosenstock, J.; Sesti, G.; Schmidt, W.E.; Montanya, E.; Brett, J.H.; Zychma, M.; Blonde, L.; Group, L.-S. Liraglutide once a day versus exenatide twice a day for type 2 diabetes: a 26-week randomised, parallel-group, multinational, open-label trial (LEAD-6). *Lancet* **2009**, *374*, 39-47, doi:10.1016/S0140-6736(09)60659-0.
81. Garber, A.; Henry, R.; Ratner, R.; Garcia-Hernandez, P.A.; Rodriguez-Pattzi, H.; Olvera-Alvarez, I.; Hale, P.M.; Zdravkovic, M.; Bode, B.; Group, L.-S. Liraglutide versus glimepiride monotherapy for type 2 diabetes (LEAD-3 Mono): a randomised, 52-week, phase III, double-blind, parallel-treatment trial. *Lancet* **2009**, *373*, 473-481, doi:10.1016/S0140-6736(08)61246-5.
82. Pfeffer, M.A.; Claggett, B.; Diaz, R.; Dickstein, K.; Gerstein, H.C.; Kober, L.V.; Lawson, F.C.; Ping, L.; Wei, X.; Lewis, E.F.; et al. Lixisenatide in Patients with Type 2 Diabetes and Acute Coronary Syndrome. *N Engl J Med* **2015**, *373*, 2247-2257, doi:10.1056/NEJMoa1509225.
83. Nauck, M.A.; Stewart, M.W.; Perkins, C.; Jones-Leone, A.; Yang, F.; Perry, C.; Reinhardt, R.R.; Rendell, M. Efficacy and safety of once-

weekly GLP-1 receptor agonist albiglutide (HARMONY 2): 52 week primary endpoint results from a randomised, placebo-controlled trial in patients with type 2 diabetes mellitus inadequately controlled with diet and exercise. *Diabetologia* **2016**, *59*, 266-274, doi:10.1007/s00125-015-3795-1.
84. Davies, M.J.; Bergenstal, R.; Bode, B.; Kushner, R.F.; Lewin, A.; Skjoth, T.V.; Andreasen, A.H.; Jensen, C.B.; DeFronzo, R.A.; Group, N.N.S. Efficacy of Liraglutide for Weight Loss Among Patients With Type 2 Diabetes: The SCALE Diabetes Randomized Clinical Trial. *Jama* **2015**, *314*, 687-699, doi:10.1001/jama.2015.9676.
85. Mathieu, C.; Ranetti, A.E.; Li, D.; Ekholm, E.; Cook, W.; Hirshberg, B.; Chen, H.; Hansen, L.; Iqbal, N. Randomized, Double-Blind, Phase 3 Trial of Triple Therapy With Dapagliflozin Add-on to Saxagliptin Plus Metformin in Type 2 Diabetes. *Diabetes Care* **2015**, *38*, 2009-2017, doi:10.2337/dc15-0779.
86. Kovacs, C.S.; Seshiah, V.; Merker, L.; Christiansen, A.V.; Roux, F.; Salsali, A.; Kim, G.; Stella, P.; Woerle, H.J.; Broedl, U.C.; et al. Empagliflozin as Add-on Therapy to Pioglitazone With or Without Metformin in Patients With Type 2 Diabetes Mellitus. *Clin Ther* **2015**, *37*, 1773-1788 e1771, doi:10.1016/j.clinthera.2015.05.511.
87. Giorgino, F.; Benroubi, M.; Sun, J.H.; Zimmermann, A.G.; Pechtner, V. Efficacy and Safety of Once-Weekly Dulaglutide Versus Insulin Glargine in Patients With Type 2 Diabetes on Metformin and Glimepiride (AWARD-2). *Diabetes Care* **2015**, *38*, 2241-2249, doi:10.2337/dc14-1625.
88. Blonde, L.; Jendle, J.; Gross, J.; Woo, V.; Jiang, H.; Fahrbach, J.L.; Milicevic, Z. Once-weekly dulaglutide versus bedtime insulin glargine, both in combination with prandial insulin lispro, in patients with type 2 diabetes (AWARD-4): a randomised, open-label, phase 3, non-inferiority study. *Lancet* **2015**, *385*, 2057-2066, doi:10.1016/S0140-6736(15)60936-9.
89. Weinstock, R.S.; Guerci, B.; Umpierrez, G.; Nauck, M.A.; Skrivanek, Z.; Milicevic, Z. Safety and efficacy of once-weekly dulaglutide versus sitagliptin after 2 years in metformin-treated patients with type 2 diabetes (AWARD-5): a randomized, phase III study. *Diabetes Obes Metab* **2015**, *17*, 849-858, doi:10.1111/dom.12479.
90. Rosenstock, J.; Fonseca, V.A.; Gross, J.L.; Ratner, R.E.; Ahren, B.; Chow, F.C.; Yang, F.; Miller, D.; Johnson, S.L.; Stewart, M.W.; et al. Advancing basal insulin replacement in type 2 diabetes inadequately controlled with insulin glargine plus oral agents: a comparison of

adding albiglutide, a weekly GLP-1 receptor agonist, versus thrice-daily prandial insulin lispro. *Diabetes Care* **2014**, *37*, 2317-2325, doi:10.2337/dc14-0001.

91. Wysham, C.; Blevins, T.; Arakaki, R.; Colon, G.; Garcia, P.; Atisso, C.; Kuhstoss, D.; Lakshmanan, M. Efficacy and safety of dulaglutide added onto pioglitazone and metformin versus exenatide in type 2 diabetes in a randomized controlled trial (AWARD-1). *Diabetes Care* **2014**, *37*, 2159-2167, doi:10.2337/dc13-2760.
92. Yu, M.; Brunt, K.V.; Milicevic, Z.; Varnado, O.; Boye, K.S. Patient-reported Outcomes in Patients with Type 2 Diabetes Treated with Dulaglutide Added to Titrated Insulin Glargine (AWARD-9). *Clin Ther* **2017**, *39*, 2284-2295, doi:10.1016/j.clinthera.2017.10.002.
93. Home, P.D.; Ahren, B.; Reusch, J.E.B.; Rendell, M.; Weissman, P.N.; Cirkel, D.T.; Miller, D.; Ambery, P.; Carr, M.C.; Nauck, M.A. Three-year data from 5 HARMONY phase 3 clinical trials of albiglutide in type 2 diabetes mellitus: Long-term efficacy with or without rescue therapy. *Diabetes Res Clin Pract* **2017**, *131*, 49-60, doi:10.1016/j.diabres.2017.06.013.
94. Januzzi, J.L., Jr.; Butler, J.; Jarolim, P.; Sattar, N.; Vijapurkar, U.; Desai, M.; Davies, M.J. Effects of Canagliflozin on Cardiovascular Biomarkers in Older Adults With Type 2 Diabetes. *J Am Coll Cardiol* **2017**, *70*, 704-712, doi:10.1016/j.jacc.2017.06.016.
95. Ahren, B.; Masmiquel, L.; Kumar, H.; Sargin, M.; Karsbol, J.D.; Jacobsen, S.H.; Chow, F. Efficacy and safety of once-weekly semaglutide versus once-daily sitagliptin as an add-on to metformin, thiazolidinediones, or both, in patients with type 2 diabetes (SUSTAIN 2): a 56-week, double-blind, phase 3a, randomised trial. *Lancet Diabetes Endocrinol* **2017**, *5*, 341-354, doi:10.1016/S2213-8587(17)30092-X.
96. Mellander, A.; Billger, M.; Johnsson, E.; Traff, A.K.; Yoshida, S.; Johnsson, K. Hypersensitivity Events, Including Potentially Hypersensitivity-Related Skin Events, with Dapagliflozin in Patients with Type 2 Diabetes Mellitus: A Pooled Analysis. *Clinical drug investigation* **2016**, *36*, 925-933, doi:10.1007/s40261-016-0438-3.
97. Hadjadj, S.; Rosenstock, J.; Meinicke, T.; Woerle, H.J.; Broedl, U.C. Initial Combination of Empagliflozin and Metformin in Patients With Type 2 Diabetes. *Diabetes Care* **2016**, *39*, 1718-1728, doi:10.2337/dc16-0522.
98. Nauck, M.; Rizzo, M.; Johnson, A.; Bosch-Traberg, H.; Madsen, J.; Cariou, B. Once-Daily Liraglutide Versus Lixisenatide as Add-on to Metformin in Type 2 Diabetes: A 26-Week Randomized Controlled Clinical Trial. *Diabetes Care* **2016**, *39*, 1501-1509, doi:10.2337/dc15-2479.

99. Investigators, F.-S.T. Glucose Variability in a 26-Week Randomized Comparison of Mealtime Treatment With Rapid-Acting Insulin Versus GLP-1 Agonist in Participants With Type 2 Diabetes at High Cardiovascular Risk. *Diabetes Care* **2016**, *39*, 973-981, doi:10.2337/dc15-2782.
100. Dungan, K.M.; Weitgasser, R.; Perez Manghi, F.; Pintilei, E.; Fahrbach, J.L.; Jiang, H.H.; Shell, J.; Robertson, K.E. A 24-week study to evaluate the efficacy and safety of once-weekly dulaglutide added on to glimepiride in type 2 diabetes (AWARD-8). *Diabetes Obes Metab* **2016**, *18*, 475-482, doi:10.1111/dom.12634.
101. Davies, M.J.; Bain, S.C.; Atkin, S.L.; Rossing, P.; Scott, D.; Shamkhalova, M.S.; Bosch-Traberg, H.; Syren, A.; Umpierrez, G.E. Efficacy and Safety of Liraglutide Versus Placebo as Add-on to Glucose-Lowering Therapy in Patients With Type 2 Diabetes and Moderate Renal Impairment (LIRA-RENAL): A Randomized Clinical Trial. *Diabetes Care* **2016**, *39*, 222-230, doi:10.2337/dc14-2883.
102. Pratley, R.; Amod, A.; Hoff, S.T.; Kadowaki, T.; Lingvay, I.; Nauck, M.; Pedersen, K.B.; Saugstrup, T.; Meier, J.J.; investigators, P. Oral semaglutide versus subcutaneous liraglutide and placebo in type 2 diabetes (PIONEER 4): a randomised, double-blind, phase 3a trial. *Lancet* **2019**, *394*, 39-50, doi:10.1016/S0140-6736(19)31271-1.
103. Gallo, S.; Charbonnel, B.; Goldman, A.; Shi, H.; Huyck, S.; Darekar, A.; Lauring, B.; Terra, S.G. Long-term efficacy and safety of ertugliflozin in patients with type 2 diabetes mellitus inadequately controlled with metformin monotherapy: 104-week VERTIS MET trial. *Diabetes Obes Metab* **2019**, *21*, 1027-1036, doi:10.1111/dom.13631.
104. Coskun, T.; Sloop, K.W.; Loghin, C.; Alsina-Fernandez, J.; Urva, S.; Bokvist, K.B.; Cui, X.; Briere, D.A.; Cabrera, O.; Roell, W.C.; et al. LY3298176, a novel dual GIP and GLP-1 receptor agonist for the treatment of type 2 diabetes mellitus: From discovery to clinical proof of concept. *Mol Metab* **2018**, *18*, 3-14, doi:10.1016/j.molmet.2018.09.009.
105. Danne, T.; Cariou, B.; Banks, P.; Brandle, M.; Brath, H.; Franek, E.; Kushner, J.A.; Lapuerta, P.; McGuire, D.K.; Peters, A.L.; et al. HbA(1c) and Hypoglycemia Reductions at 24 and 52 Weeks With Sotagliflozin in Combination With Insulin in Adults With Type 1 Diabetes: The European inTandem2 Study. *Diabetes Care* **2018**, *41*, 1981-1990, doi:10.2337/dc18-0342.
106. Dekkers, C.C.J.; Petrykiv, S.; Laverman, G.D.; Cherney, D.Z.; Gansevoort, R.T.; Heerspink, H.J.L. Effects of the SGLT-2 inhibitor dapagliflozin on glomerular and tubular injury markers. *Diabetes Obes Metab* **2018**, *20*, 1988-1993, doi:10.1111/dom.13301.

107. Ludvik, B.; Frias, J.P.; Tinahones, F.J.; Wainstein, J.; Jiang, H.; Robertson, K.E.; Garcia-Perez, L.E.; Woodward, D.B.; Milicevic, Z. Dulaglutide as add-on therapy to SGLT2 inhibitors in patients with inadequately controlled type 2 diabetes (AWARD-10): a 24-week, randomised, double-blind, placebo-controlled trial. *Lancet Diabetes Endocrinol* **2018**, *6*, 370-381, doi:10.1016/S2213-8587(18)30023-8.

108. Aronson, R.; Frias, J.; Goldman, A.; Darekar, A.; Lauring, B.; Terra, S.G. Long-term efficacy and safety of ertugliflozin monotherapy in patients with inadequately controlled T2DM despite diet and exercise: VERTIS MONO extension study. *Diabetes Obes Metab* **2018**, *20*, 1453-1460, doi:10.1111/dom.13251.

109. Pratley, R.E.; Aroda, V.R.; Lingvay, I.; Ludemann, J.; Andreassen, C.; Navarria, A.; Viljoen, A.; investigators, S. Semaglutide versus dulaglutide once weekly in patients with type 2 diabetes (SUSTAIN 7): a randomised, open-label, phase 3b trial. *Lancet Diabetes Endocrinol* **2018**, *6*, 275-286, doi:10.1016/S2213-8587(18)30024-X.

110. Kaku, K.; Yamada, Y.; Watada, H.; Abiko, A.; Nishida, T.; Zacho, J.; Kiyosue, A. Safety and efficacy of once-weekly semaglutide vs additional oral antidiabetic drugs in Japanese people with inadequately controlled type 2 diabetes: A randomized trial. *Diabetes Obes Metab* **2018**, *20*, 1202-1212, doi:10.1111/dom.13218.

111. Ahmann, A.J.; Capehorn, M.; Charpentier, G.; Dotta, F.; Henkel, E.; Lingvay, I.; Holst, A.G.; Annett, M.P.; Aroda, V.R. Efficacy and Safety of Once-Weekly Semaglutide Versus Exenatide ER in Subjects With Type 2 Diabetes (SUSTAIN 3): A 56-Week, Open-Label, Randomized Clinical Trial. *Diabetes Care* **2018**, *41*, 258-266, doi:10.2337/dc17-0417.

112. Rosenstock, J.; Wysham, C.; Frias, J.P.; Kaneko, S.; Lee, C.J.; Fernandez Lando, L.; Mao, H.; Cui, X.; Karanikas, C.A.; Thieu, V.T. Efficacy and safety of a novel dual GIP and GLP-1 receptor agonist tirzepatide in patients with type 2 diabetes (SURPASS-1): a double-blind, randomised, phase 3 trial. *Lancet* **2021**, *398*, 143-155, doi:10.1016/S0140-6736(21)01324-6.

113. Rubino, D.; Abrahamsson, N.; Davies, M.; Hesse, D.; Greenway, F.L.; Jensen, C.; Lingvay, I.; Mosenzon, O.; Rosenstock, J.; Rubio, M.A.; et al. Effect of Continued Weekly Subcutaneous Semaglutide vs Placebo on Weight Loss Maintenance in Adults With Overweight or Obesity: The STEP 4 Randomized Clinical Trial. *Jama* **2021**, *325*, 1414-1425, doi:10.1001/jama.2021.3224.

114. Wadden, T.A.; Bailey, T.S.; Billings, L.K.; Davies, M.; Frias, J.P.; Koroleva, A.; Lingvay, I.; O'Neil, P.M.; Rubino, D.M.; Skovgaard, D.; et al. Effect of Subcutaneous Semaglutide vs Placebo as an Adjunct to Intensive Behavioral Therapy on Body Weight in Adults With

Overweight or Obesity: The STEP 3 Randomized Clinical Trial. *Jama* **2021**, *325*, 1403-1413, doi:10.1001/jama.2021.1831.
115. Wilding, J.P.H.; Batterham, R.L.; Calanna, S.; Davies, M.; Van Gaal, L.F.; Lingvay, I.; McGowan, B.M.; Rosenstock, J.; Tran, M.T.D.; Wadden, T.A.; et al. Once-Weekly Semaglutide in Adults with Overweight or Obesity. *N Engl J Med* **2021**, *384*, 989-1002, doi:10.1056/NEJMoa2032183.
116. Stack, A.G.; Han, D.; Goldwater, R.; Johansson, S.; Dronamraju, N.; Oscarsson, J.; Johnsson, E.; Parkinson, J.; Erlandsson, F. Dapagliflozin Added to Verinurad Plus Febuxostat Further Reduces Serum Uric Acid in Hyperuricemia: The QUARTZ Study. *J Clin Endocrinol Metab* **2021**, *106*, e2347-e2356, doi:10.1210/clinem/dgaa748.
117. Wang, J.; Li, H.Q.; Xu, X.H.; Kong, X.C.; Sun, R.; Jing, T.; Ye, L.; Su, X.F.; Ma, J.H. The Effects of Once-Weekly Dulaglutide and Insulin Glargine on Glucose Fluctuation in Poorly Oral-Antidiabetic Controlled Patients with Type 2 Diabetes Mellitus. *Biomed Res Int* **2019**, *2019*, 2682657, doi:10.1155/2019/2682657.
118. Lingvay, I.; Catarig, A.M.; Frias, J.P.; Kumar, H.; Lausvig, N.L.; le Roux, C.W.; Thielke, D.; Viljoen, A.; McCrimmon, R.J. Efficacy and safety of once-weekly semaglutide versus daily canagliflozin as add-on to metformin in patients with type 2 diabetes (SUSTAIN 8): a double-blind, phase 3b, randomised controlled trial. *Lancet Diabetes Endocrinol* **2019**, *7*, 834-844, doi:10.1016/S2213-8587(19)30311-0.
119. Mullins, R.J.; Mustapic, M.; Chia, C.W.; Carlson, O.; Gulyani, S.; Tran, J.; Li, Y.; Mattson, M.P.; Resnick, S.; Egan, J.M.; et al. A Pilot Study of Exenatide Actions in Alzheimer's Disease. *Curr Alzheimer Res* **2019**, *16*, 741-752, doi:10.2174/1567205016666190913155950.
120. Pieber, T.R.; Bode, B.; Mertens, A.; Cho, Y.M.; Christiansen, E.; Hertz, C.L.; Wallenstein, S.O.R.; Buse, J.B.; investigators, P. Efficacy and safety of oral semaglutide with flexible dose adjustment versus sitagliptin in type 2 diabetes (PIONEER 7): a multicentre, open-label, randomised, phase 3a trial. *Lancet Diabetes Endocrinol* **2019**, *7*, 528-539, doi:10.1016/S2213-8587(19)30194-9.
121. Aroda, V.R.; Rosenstock, J.; Terauchi, Y.; Altuntas, Y.; Lalic, N.M.; Morales Villegas, E.C.; Jeppesen, O.K.; Christiansen, E.; Hertz, C.L.; Haluzik, M.; et al. PIONEER 1: Randomized Clinical Trial of the Efficacy and Safety of Oral Semaglutide Monotherapy in Comparison With Placebo in Patients With Type 2 Diabetes. *Diabetes Care* **2019**, *42*, 1724-1732, doi:10.2337/dc19-0749.
122. Inagaki, N.; Takeuchi, M.; Oura, T.; Imaoka, T.; Seino, Y. Efficacy and safety of tirzepatide monotherapy compared with dulaglutide in Japanese patients with type 2 diabetes (SURPASS J-mono): a double-blind, multicentre, randomised, phase 3 trial. *Lancet Diabetes*

Endocrinol **2022**, *10*, 623-633, doi:10.1016/S2213-8587(22)00188-7.
123. Kadowaki, T.; Chin, R.; Ozeki, A.; Imaoka, T.; Ogawa, Y. Safety and efficacy of tirzepatide as an add-on to single oral antihyperglycaemic medication in patients with type 2 diabetes in Japan (SURPASS J-combo): a multicentre, randomised, open-label, parallel-group, phase 3 trial. *Lancet Diabetes Endocrinol* **2022**, *10*, 634-644, doi:10.1016/S2213-8587(22)00187-5.
124. Thiele, K.; Rau, M.; Hartmann, N.K.; Moller, M.; Mollmann, J.; Jankowski, J.; Keszei, A.P.; Bohm, M.; Floege, J.; Marx, N.; et al. Empagliflozin reduces markers of acute kidney injury in patients with acute decompensated heart failure. *ESC Heart Fail* **2022**, *9*, 2233-2238, doi:10.1002/ehf2.13955.
125. Kellerer, M.; Kaltoft, M.S.; Lawson, J.; Nielsen, L.L.; Strojek, K.; Tabak, O.; Jacob, S. Effect of once-weekly semaglutide versus thrice-daily insulin aspart, both as add-on to metformin and optimized insulin glargine treatment in participants with type 2 diabetes (SUSTAIN 11): A randomized, open-label, multinational, phase 3b trial. *Diabetes Obes Metab* **2022**, *24*, 1788-1799, doi:10.1111/dom.14765.
126. Tuttle, K.R.; Levin, A.; Nangaku, M.; Kadowaki, T.; Agarwal, R.; Hauske, S.J.; Elsasser, A.; Ritter, I.; Steubl, D.; Wanner, C.; et al. Safety of Empagliflozin in Patients With Type 2 Diabetes and Chronic Kidney Disease: Pooled Analysis of Placebo-Controlled Clinical Trials. *Diabetes Care* **2022**, *45*, 1445-1452, doi:10.2337/dc21-2034.
127. Heise, T.; Mari, A.; DeVries, J.H.; Urva, S.; Li, J.; Pratt, E.J.; Coskun, T.; Thomas, M.K.; Mather, K.J.; Haupt, A.; et al. Effects of subcutaneous tirzepatide versus placebo or semaglutide on pancreatic islet function and insulin sensitivity in adults with type 2 diabetes: a multicentre, randomised, double-blind, parallel-arm, phase 1 clinical trial. *Lancet Diabetes Endocrinol* **2022**, *10*, 418-429, doi:10.1016/S2213-8587(22)00085-7.
128. Fox, C.K.; Clark, J.M.; Rudser, K.D.; Ryder, J.R.; Gross, A.C.; Nathan, B.M.; Sunni, M.; Dengel, D.R.; Billington, C.J.; Bensignor, M.O.; et al. Exenatide for weight-loss maintenance in adolescents with severe obesity: A randomized, placebo-controlled trial. *Obesity (Silver Spring)* **2022**, *30*, 1105-1115, doi:10.1002/oby.23395.
129. Dahl, D.; Onishi, Y.; Norwood, P.; Huh, R.; Bray, R.; Patel, H.; Rodriguez, A. Effect of Subcutaneous Tirzepatide vs Placebo Added to Titrated Insulin Glargine on Glycemic Control in Patients With Type 2 Diabetes: The SURPASS-5 Randomized Clinical Trial. *Jama* **2022**, *327*, 534-545, doi:10.1001/jama.2022.0078.

130. Kadowaki, T.; Isendahl, J.; Khalid, U.; Lee, S.Y.; Nishida, T.; Ogawa, W.; Tobe, K.; Yamauchi, T.; Lim, S.; investigators, S. Semaglutide once a week in adults with overweight or obesity, with or without type 2 diabetes in an east Asian population (STEP 6): a randomised, double-blind, double-dummy, placebo-controlled, phase 3a trial. *Lancet Diabetes Endocrinol* **2022**, *10*, 193-206, doi:10.1016/S2213-8587(22)00008-0.
131. Rodgers, M.; Migdal, A.L.; Rodriguez, T.G.; Chen, Z.Z.; Nath, A.K.; Gerszten, R.E.; Kasid, N.; Toschi, E.; Tripaldi, J.; Heineman, B.; et al. Weight Loss Outcomes Among Early High Responders to Exenatide Treatment: A Randomized, Placebo Controlled Study in Overweight and Obese Women. *Front Endocrinol (Lausanne)* **2021**, *12*, 742873, doi:10.3389/fendo.2021.742873.
132. Mu, Y.; Bao, X.; Eliaschewitz, F.G.; Hansen, M.R.; Kim, B.T.; Koroleva, A.; Ma, R.C.W.; Yang, T.; Zu, N.; Liu, M.; et al. Efficacy and safety of once weekly semaglutide 2.4 mg for weight management in a predominantly east Asian population with overweight or obesity (STEP 7): a double-blind, multicentre, randomised controlled trial. *Lancet Diabetes Endocrinol* **2024**, *12*, 184-195, doi:10.1016/S2213-8587(23)00388-1.
133. Lee, B.W.; Cho, Y.M.; Kim, S.G.; Ko, S.H.; Lim, S.; Dahaoui, A.; Jeong, J.S.; Lim, H.J.; Yu, J.M. Efficacy and Safety of Once-Weekly Semaglutide Versus Once-Daily Sitagliptin as Metformin Add-on in a Korean Population with Type 2 Diabetes. *Diabetes Ther* **2024**, *15*, 547-563, doi:10.1007/s13300-023-01515-0.
134. Aronne, L.J.; Sattar, N.; Horn, D.B.; Bays, H.E.; Wharton, S.; Lin, W.Y.; Ahmad, N.N.; Zhang, S.; Liao, R.; Bunck, M.C.; et al. Continued Treatment With Tirzepatide for Maintenance of Weight Reduction in Adults With Obesity: The SURMOUNT-4 Randomized Clinical Trial. *Jama* **2024**, *331*, 38-48, doi:10.1001/jama.2023.24945.
135. Frias, J.P.; Hsia, S.; Eyde, S.; Liu, R.; Ma, X.; Konig, M.; Kazda, C.; Mather, K.J.; Haupt, A.; Pratt, E.; et al. Efficacy and safety of oral orforglipron in patients with type 2 diabetes: a multicentre, randomised, dose-response, phase 2 study. *Lancet* **2023**, *402*, 472-483, doi:10.1016/S0140-6736(23)01302-8.
136. Feitosa, M.P.M.; Lima, E.G.; Abizaid, A.A.C.; Mehran, R.; Lopes, N.H.M.; de Assis Fischer Ramos, T.; Hideo-Kajita, A.; Filho, R.K.; Junior, C.V.S. The safety of SGLT-2 inhibitors in diabetic patients submitted to elective percutaneous coronary intervention regarding kidney function: SAFE-PCI pilot study. *Diabetol Metab Syndr* **2023**, *15*, 138, doi:10.1186/s13098-023-01107-9.

137. Feng, P.; Sheng, X.; Ji, Y.; Urva, S.; Wang, F.; Miller, S.; Qian, C.; An, Z.; Cui, Y. A Phase 1 Multiple Dose Study of Tirzepatide in Chinese Patients with Type 2 Diabetes. *Adv Ther* **2023**, *40*, 3434-3445, doi:10.1007/s12325-023-02536-8.
138. Gao, L.; Lee, B.W.; Chawla, M.; Kim, J.; Huo, L.; Du, L.; Huang, Y.; Ji, L. Tirzepatide versus insulin glargine as second-line or third-line therapy in type 2 diabetes in the Asia-Pacific region: the SURPASS-AP-Combo trial. *Nat Med* **2023**, *29*, 1500-1510, doi:10.1038/s41591-023-02344-1.
139. Aroda, V.R.; Frias, J.P.; Ji, L.; Niemoeller, E.; Nguyen-Pascal, M.L.; Denkel, K.; Espinasse, M.; Guo, H.; Baek, S.; Choi, J.; et al. Efficacy and safety of once-weekly efpeglenatide in people with suboptimally controlled type 2 diabetes: The AMPLITUDE-D, AMPLITUDE-L and AMPLITUDE-S randomized controlled trials. *Diabetes Obes Metab* **2023**, *25*, 2084-2095, doi:10.1111/dom.15079.
140. Ji, L.; Lu, Y.; Li, Q.; Fu, L.; Luo, Y.; Lei, T.; Li, L.; Ye, S.; Shi, B.; Li, X.; et al. Efficacy and safety of empagliflozin in combination with insulin in Chinese patients with type 2 diabetes and insufficient glycaemic control: A phase III, randomized, double-blind, placebo-controlled, parallel study. *Diabetes Obes Metab* **2023**, *25*, 1839-1848, doi:10.1111/dom.15041.
141. Garvey, W.T.; Batterham, R.L.; Bhatta, M.; Buscemi, S.; Christensen, L.N.; Frias, J.P.; Jodar, E.; Kandler, K.; Rigas, G.; Wadden, T.A.; et al. Two-year effects of semaglutide in adults with overweight or obesity: the STEP 5 trial. *Nat Med* **2022**, *28*, 2083-2091, doi:10.1038/s41591-022-02026-4.
142. Tavares, C.A.M.; Azevedo, L.C.P.; Rea-Neto, A.; Campos, N.S.; Amendola, C.P.; Kozesinski-Nakatani, A.C.; David-Joao, P.G.; Lobo, S.M.; Filiponi, T.C.; Almeida, G.M.B.; et al. Dapagliflozin for Critically Ill Patients With Acute Organ Dysfunction: The DEFENDER Randomized Clinical Trial. *Jama* **2024**, *332*, 401-411, doi:10.1001/jama.2024.10510.
143. Zhao, L.; Cheng, Z.; Lu, Y.; Liu, M.; Chen, H.; Zhang, M.; Wang, R.; Yuan, Y.; Li, X. Tirzepatide for Weight Reduction in Chinese Adults With Obesity: The SURMOUNT-CN Randomized Clinical Trial. *Jama* **2024**, *332*, 551-560, doi:10.1001/jama.2024.9217.
144. Dei Cas, A.; Micheli, M.M.; Aldigeri, R.; Gardini, S.; Ferrari-Pellegrini, F.; Perini, M.; Messa, G.; Antonini, M.; Spigoni, V.; Cinquegrani, G.; et al. Long-acting exenatide does not prevent cognitive decline in mild cognitive impairment: a proof-of-concept clinical trial. *Journal of endocrinological investigation* **2024**, *47*, 2339-2349, doi:10.1007/s40618-024-02320-7.
145. Wason, S. Efficacy and Safety of Sotagliflozin Versus Placebo in Participants With Type 2 Diabetes Mellitus Who Have Inadequate

Glycemic Control While Taking Insulin Alone or With Other Oral Antidiabetic Agents (SOTA-INS). Available online: https://clinicaltrials.gov/study/NCT03285594?cond=NCT03285594&rank=1 (accessed on 2024/10/28).
146. Wason, S. Efficacy and Bone Safety of Sotagliflozin 400 and 200 mg Versus Placebo in Participants With Type 2 Diabetes Mellitus Who Have Inadequate Glycemic Control (SOTA-BONE). Available online: https://clinicaltrials.gov/study/NCT03386344?cond=NCT03386344&rank=1 (accessed on 2024/10/28).
147. Rehman, S.U.; Kolanu, N.D.; Mushtaq, M.M.; Ali, H.; Ahmed, Z.; Mushtaq, M.; Liaqat, M.; Sarwer, M.A.; Bokhari, S.F.H.; Ahmed, F.; et al. Assessing the Renal Outcomes of Semaglutide in Diabetic Kidney Disease: A Systematic Review. *Cureus* **2024**, *16*, e64038, doi:10.7759/cureus.64038.
148. Ziser, K.E.D.; Livori, A.C.; Morton, J.I.; Prosser, A.; Ilomaki, J.; Wood, S.J. Acute kidney injury, renal impairment and renal failure associated with sodium glucose co-transporter-2 inhibitors in at-risk groups: A systematic review. *Br J Clin Pharmacol* **2024**, *90*, 1541-1558, doi:10.1111/bcp.16088.
149. Rodriguez, R.; Kaluzna, S.D. Sodium-glucose cotransporter 2 inhibitors and cardiovascular clinical outcomes in acute heart failure: A narrative review. *Am J Health Syst Pharm* **2023**, *80*, 818-826, doi:10.1093/ajhp/zxad061.
150. Lin, Y.; Mok, M.; Harrison, J.; Battistella, M.; Farrell, A.; Leung, M.; Cheung, C. Use of sodium-glucose co-transporter 2 inhibitors in solid organ transplant recipients with pre-existing type 2 or post-transplantation diabetes mellitus: A systematic review. *Transplant Rev (Orlando)* **2023**, *37*, 100729, doi:10.1016/j.trre.2022.100729.
151. Copur, S.; Yildiz, A.; Basile, C.; Tuttle, K.R.; Kanbay, M. Is there any robust evidence showing that SGLT2 inhibitor use predisposes to acute kidney injury? *J Nephrol* **2023**, *36*, 31-43, doi:10.1007/s40620-022-01422-w.
152. Bailey, C.J.; Day, C.; Bellary, S. Renal Protection with SGLT2 Inhibitors: Effects in Acute and Chronic Kidney Disease. *Curr Diab Rep* **2022**, *22*, 39-52, doi:10.1007/s11892-021-01442-z.
153. Coca, A.; Bustamante-Munguira, E.; Fidalgo, V.; Fernandez, M.; Abad, C.; Franco, M.; Gonzalez-Pinto, A.; Pereda, D.; Canovas, S.; Bustamante-Munguira, J. EValuating the Effect of periopeRaTIve empaGliflOzin on cardiac surgery associated acute kidney injury: rationale and design of the VERTIGO study. *Clin Kidney J* **2024**, *17*, sfae229, doi:10.1093/ckj/sfae229.

154. Bliddal, H.; Bays, H.; Czernichow, S.; Udden Hemmingsson, J.; Hjelmesaeth, J.; Hoffmann Morville, T.; Koroleva, A.; Skov Neergaard, J.; Velez Sanchez, P.; Wharton, S.; et al. Once-Weekly Semaglutide in Persons with Obesity and Knee Osteoarthritis. *N Engl J Med* **2024**, *391*, 1573-1583, doi:10.1056/NEJMoa2403664.
155. Natale, P.; Tunnicliffe, D.J.; Toyama, T.; Palmer, S.C.; Saglimbene, V.M.; Ruospo, M.; Gargano, L.; Stallone, G.; Gesualdo, L.; Strippoli, G.F. Sodium-glucose co-transporter protein 2 (SGLT2) inhibitors for people with chronic kidney disease and diabetes. *The Cochrane database of systematic reviews* **2024**, *5*, CD015588, doi:10.1002/14651858.CD015588.pub2.
156. SURMOUNT-J. A Study of Tirzepatide (LY3298176) in Participants With Obesity Disease (SURMOUNT-J). Available online: https://clinicaltrials.gov/study/NCT04844918?cond=NCT04844918&rank=1 (accessed on 2024/10/28).
157. Tuttle, K.R.; Hauske, S.J.; Canziani, M.E.; Caramori, M.L.; Cherney, D.; Cronin, L.; Heerspink, H.J.L.; Hugo, C.; Nangaku, M.; Rotter, R.C.; et al. Efficacy and safety of aldosterone synthase inhibition with and without empagliflozin for chronic kidney disease: a randomised, controlled, phase 2 trial. *Lancet* **2024**, *403*, 379-390, doi:10.1016/S0140-6736(23)02408-X.
158. Buse, J.B.; Nordahl Christensen, H.; Harty, B.J.; Mitchell, J.; Soule, B.P.; Zacherle, E.; Cziraky, M.; Willey, V.J. Study design and baseline profile for adults with type 2 diabetes in the once-weekly subcutaneous SEmaglutide randomized PRAgmatic (SEPRA) trial. *BMJ Open Diabetes Res Care* **2023**, *11*, doi:10.1136/bmjdrc-2022-003206.
159. Cherney, D.Z.I.; Ferrannini, E.; Umpierrez, G.E.; Peters, A.L.; Rosenstock, J.; Powell, D.R.; Davies, M.J.; Banks, P.; Agarwal, R. Efficacy and safety of sotagliflozin in patients with type 2 diabetes and stage 3 chronic kidney disease. *Diabetes Obes Metab* **2023**, *25*, 1646-1657, doi:10.1111/dom.15019.
160. Garvey, W.T.; Frias, J.P.; Jastreboff, A.M.; le Roux, C.W.; Sattar, N.; Aizenberg, D.; Mao, H.; Zhang, S.; Ahmad, N.N.; Bunck, M.C.; et al. Tirzepatide once weekly for the treatment of obesity in people with type 2 diabetes (SURMOUNT-2): a double-blind, randomised, multicentre, placebo-controlled, phase 3 trial. *Lancet* **2023**, *402*, 613-626, doi:10.1016/S0140-6736(23)01200-X.
161. The, E.-K.C.G.; Herrington, W.G.; Staplin, N.; Wanner, C.; Green, J.B.; Hauske, S.J.; Emberson, J.R.; Preiss, D.; Judge, P.; Mayne, K.J.; et al. Empagliflozin in Patients with Chronic Kidney Disease. *N Engl J Med* **2023**, *388*, 117-127, doi:10.1056/NEJMoa2204233.
162. Lincoff, A.M.; Brown-Frandsen, K.; Colhoun, H.M.; Deanfield, J.; Emerson, S.S.; Esbjerg, S.; Hardt-Lindberg, S.; Hovingh, G.K.; Kahn, S.E.;

Kushner, R.F.; et al. Semaglutide and Cardiovascular Outcomes in Obesity without Diabetes. *N Engl J Med* **2023**, *389*, 2221-2232, doi:10.1056/NEJMoa2307563.

163. Rosenstock, J.; Frias, J.P.; Rodbard, H.W.; Tofe, S.; Sears, E.; Huh, R.; Fernandez Lando, L.; Patel, H. Tirzepatide vs Insulin Lispro Added to Basal Insulin in Type 2 Diabetes: The SURPASS-6 Randomized Clinical Trial. *Jama* **2023**, *330*, 1631-1640, doi:10.1001/jama.2023.20294.

164. Wadden, T.A.; Chao, A.M.; Machineni, S.; Kushner, R.; Ard, J.; Srivastava, G.; Halpern, B.; Zhang, S.; Chen, J.; Bunck, M.C.; et al. Tirzepatide after intensive lifestyle intervention in adults with overweight or obesity: the SURMOUNT-3 phase 3 trial. *Nat Med* **2023**, *29*, 2909-2918, doi:10.1038/s41591-023-02597-w.

165. Frias, J.P.; Choi, J.; Rosenstock, J.; Popescu, L.; Niemoeller, E.; Muehlen-Bartmer, I.; Baek, S. Efficacy and Safety of Once-Weekly Efpeglenatide Monotherapy Versus Placebo in Type 2 Diabetes: The AMPLITUDE-M Randomized Controlled Trial. *Diabetes Care* **2022**, *45*, 1592-1600, doi:10.2337/dc21-2656.

166. Rubino, D.M.; Greenway, F.L.; Khalid, U.; O'Neil, P.M.; Rosenstock, J.; Sorrig, R.; Wadden, T.A.; Wizert, A.; Garvey, W.T.; Investigators, S. Effect of Weekly Subcutaneous Semaglutide vs Daily Liraglutide on Body Weight in Adults With Overweight or Obesity Without Diabetes: The STEP 8 Randomized Clinical Trial. *Jama* **2022**, *327*, 138-150, doi:10.1001/jama.2021.23619.

167. Solomon, S.D.; McMurray, J.J.V.; Claggett, B.; de Boer, R.A.; DeMets, D.; Hernandez, A.F.; Inzucchi, S.E.; Kosiborod, M.N.; Lam, C.S.P.; Martinez, F.; et al. Dapagliflozin in Heart Failure with Mildly Reduced or Preserved Ejection Fraction. *N Engl J Med* **2022**, *387*, 1089-1098, doi:10.1056/NEJMoa2206286.

168. Spertus, J.A.; Birmingham, M.C.; Nassif, M.; Damaraju, C.V.; Abbate, A.; Butler, J.; Lanfear, D.E.; Lingvay, I.; Kosiborod, M.N.; Januzzi, J.L. The SGLT2 inhibitor canagliflozin in heart failure: the CHIEF-HF remote, patient-centered randomized trial. *Nat Med* **2022**, *28*, 809-813, doi:10.1038/s41591-022-01703-8.

169. Voors, A.A.; Angermann, C.E.; Teerlink, J.R.; Collins, S.P.; Kosiborod, M.; Biegus, J.; Ferreira, J.P.; Nassif, M.E.; Psotka, M.A.; Tromp, J.; et al. The SGLT2 inhibitor empagliflozin in patients hospitalized for acute heart failure: a multinational randomized trial. *Nat Med* **2022**, *28*, 568-574, doi:10.1038/s41591-021-01659-1.

170. Wada, T.; Mori-Anai, K.; Takahashi, A.; Matsui, T.; Inagaki, M.; Iida, M.; Maruyama, K.; Tsuda, H. Effect of canagliflozin on the decline of

estimated glomerular filtration rate in chronic kidney disease patients with type 2 diabetes mellitus: A multicenter, randomized, double-blind, placebo-controlled, parallel-group, phase III study in Japan. *J Diabetes Investig* **2022**, *13*, 1981-1989, doi:10.1111/jdi.13888.
171. Anker, S.D.; Butler, J.; Filippatos, G.; Ferreira, J.P.; Bocchi, E.; Bohm, M.; Brunner-La Rocca, H.P.; Choi, D.J.; Chopra, V.; Chuquiure-Valenzuela, E.; et al. Empagliflozin in Heart Failure with a Preserved Ejection Fraction. *N Engl J Med* **2021**, *385*, 1451-1461, doi:10.1056/NEJMoa2107038.
172. Bhatt, D.L.; Szarek, M.; Pitt, B.; Cannon, C.P.; Leiter, L.A.; McGuire, D.K.; Lewis, J.B.; Riddle, M.C.; Inzucchi, S.E.; Kosiborod, M.N.; et al. Sotagliflozin in Patients with Diabetes and Chronic Kidney Disease. *N Engl J Med* **2021**, *384*, 129-139, doi:10.1056/NEJMoa2030186.
173. Bhatt, D.L.; Szarek, M.; Steg, P.G.; Cannon, C.P.; Leiter, L.A.; McGuire, D.K.; Lewis, J.B.; Riddle, M.C.; Voors, A.A.; Metra, M.; et al. Sotagliflozin in Patients with Diabetes and Recent Worsening Heart Failure. *N Engl J Med* **2021**, *384*, 117-128, doi:10.1056/NEJMoa2030183.
174. Davies, M.; Faerch, L.; Jeppesen, O.K.; Pakseresht, A.; Pedersen, S.D.; Perreault, L.; Rosenstock, J.; Shimomura, I.; Viljoen, A.; Wadden, T.A.; et al. Semaglutide 2.4 mg once a week in adults with overweight or obesity, and type 2 diabetes (STEP 2): a randomised, double-blind, double-dummy, placebo-controlled, phase 3 trial. *Lancet* **2021**, *397*, 971-984, doi:10.1016/S0140-6736(21)00213-0.
175. Frias, J.P.; Davies, M.J.; Rosenstock, J.; Perez Manghi, F.C.; Fernandez Lando, L.; Bergman, B.K.; Liu, B.; Cui, X.; Brown, K.; Investigators, S.-. Tirzepatide versus Semaglutide Once Weekly in Patients with Type 2 Diabetes. *N Engl J Med* **2021**, *385*, 503-515, doi:10.1056/NEJMoa2107519.
176. Gerstein, H.C.; Sattar, N.; Rosenstock, J.; Ramasundarahettige, C.; Pratley, R.; Lopes, R.D.; Lam, C.S.P.; Khurmi, N.S.; Heenan, L.; Del Prato, S.; et al. Cardiovascular and Renal Outcomes with Efpeglenatide in Type 2 Diabetes. *N Engl J Med* **2021**, *385*, 896-907, doi:10.1056/NEJMoa2108269.
177. Kosiborod, M.N.; Esterline, R.; Furtado, R.H.M.; Oscarsson, J.; Gasparyan, S.B.; Koch, G.G.; Martinez, F.; Mukhtar, O.; Verma, S.; Chopra, V.; et al. Dapagliflozin in patients with cardiometabolic risk factors hospitalised with COVID-19 (DARE-19): a randomised, double-blind, placebo-controlled, phase 3 trial. *Lancet Diabetes Endocrinol* **2021**, *9*, 586-594, doi:10.1016/S2213-8587(21)00180-7.
178. Lock, J.P. Bexagliflozin Efficacy and Safety Trial (BEST). Available online:

https://clinicaltrials.gov/study/NCT02558296?cond=NCT02558296&rank=1 (accessed on 2024/10/28).
179. Cannon, C.P.; Pratley, R.; Dagogo-Jack, S.; Mancuso, J.; Huyck, S.; Masiukiewicz, U.; Charbonnel, B.; Frederich, R.; Gallo, S.; Cosentino, F.; et al. Cardiovascular Outcomes with Ertugliflozin in Type 2 Diabetes. *N Engl J Med* **2020**, *383*, 1425-1435, doi:10.1056/NEJMoa2004967.
180. Heerspink, H.J.L.; Stefansson, B.V.; Correa-Rotter, R.; Chertow, G.M.; Greene, T.; Hou, F.F.; Mann, J.F.E.; McMurray, J.J.V.; Lindberg, M.; Rossing, P.; et al. Dapagliflozin in Patients with Chronic Kidney Disease. *N Engl J Med* **2020**, *383*, 1436-1446, doi:10.1056/NEJMoa2024816.
181. Packer, M.; Anker, S.D.; Butler, J.; Filippatos, G.; Pocock, S.J.; Carson, P.; Januzzi, J.; Verma, S.; Tsutsui, H.; Brueckmann, M.; et al. Cardiovascular and Renal Outcomes with Empagliflozin in Heart Failure. *N Engl J Med* **2020**, *383*, 1413-1424, doi:10.1056/NEJMoa2022190.
182. Gerstein, H.C.; Colhoun, H.M.; Dagenais, G.R.; Diaz, R.; Lakshmanan, M.; Pais, P.; Probstfield, J.; Riesmeyer, J.S.; Riddle, M.C.; Ryden, L.; et al. Dulaglutide and cardiovascular outcomes in type 2 diabetes (REWIND): a double-blind, randomised placebo-controlled trial. *Lancet* **2019**, *394*, 121-130, doi:10.1016/S0140-6736(19)31149-3.
183. Husain, M.; Birkenfeld, A.L.; Donsmark, M.; Dungan, K.; Eliaschewitz, F.G.; Franco, D.R.; Jeppesen, O.K.; Lingvay, I.; Mosenzon, O.; Pedersen, S.D.; et al. Oral Semaglutide and Cardiovascular Outcomes in Patients with Type 2 Diabetes. *N Engl J Med* **2019**, *381*, 841-851, doi:10.1056/NEJMoa1901118.
184. McMurray, J.J.V.; Solomon, S.D.; Inzucchi, S.E.; Kober, L.; Kosiborod, M.N.; Martinez, F.A.; Ponikowski, P.; Sabatine, M.S.; Anand, I.S.; Belohlavek, J.; et al. Dapagliflozin in Patients with Heart Failure and Reduced Ejection Fraction. *N Engl J Med* **2019**, *381*, 1995-2008, doi:10.1056/NEJMoa1911303.
185. Mosenzon, O.; Blicher, T.M.; Rosenlund, S.; Eriksson, J.W.; Heller, S.; Hels, O.H.; Pratley, R.; Sathyapalan, T.; Desouza, C.; Investigators, P. Efficacy and safety of oral semaglutide in patients with type 2 diabetes and moderate renal impairment (PIONEER 5): a placebo-controlled, randomised, phase 3a trial. *Lancet Diabetes Endocrinol* **2019**, *7*, 515-527, doi:10.1016/S2213-8587(19)30192-5.
186. Perkovic, V.; Jardine, M.J.; Neal, B.; Bompoint, S.; Heerspink, H.J.L.; Charytan, D.M.; Edwards, R.; Agarwal, R.; Bakris, G.; Bull, S.; et al. Canagliflozin and Renal Outcomes in Type 2 Diabetes and Nephropathy. *N Engl J Med* **2019**, *380*, 2295-2306,

doi:10.1056/NEJMoa1811744.
187. Rodbard, H.W.; Rosenstock, J.; Canani, L.H.; Deerochanawong, C.; Gumprecht, J.; Lindberg, S.O.; Lingvay, I.; Sondergaard, A.L.; Treppendahl, M.B.; Montanya, E.; et al. Oral Semaglutide Versus Empagliflozin in Patients With Type 2 Diabetes Uncontrolled on Metformin: The PIONEER 2 Trial. *Diabetes Care* **2019**, *42*, 2272-2281, doi:10.2337/dc19-0883.
188. Rosenstock, J.; Allison, D.; Birkenfeld, A.L.; Blicher, T.M.; Deenadayalan, S.; Jacobsen, J.B.; Serusclat, P.; Violante, R.; Watada, H.; Davies, M.; et al. Effect of Additional Oral Semaglutide vs Sitagliptin on Glycated Hemoglobin in Adults With Type 2 Diabetes Uncontrolled With Metformin Alone or With Sulfonylurea: The PIONEER 3 Randomized Clinical Trial. *Jama* **2019**, *321*, 1466-1480, doi:10.1001/jama.2019.2942.
189. Wiviott, S.D.; Raz, I.; Bonaca, M.P.; Mosenzon, O.; Kato, E.T.; Cahn, A.; Silverman, M.G.; Zelniker, T.A.; Kuder, J.F.; Murphy, S.A.; et al. Dapagliflozin and Cardiovascular Outcomes in Type 2 Diabetes. *N Engl J Med* **2019**, *380*, 347-357, doi:10.1056/NEJMoa1812389.
190. Buse, J.B.; Garg, S.K.; Rosenstock, J.; Bailey, T.S.; Banks, P.; Bode, B.W.; Danne, T.; Kushner, J.A.; Lane, W.S.; Lapuerta, P.; et al. Sotagliflozin in Combination With Optimized Insulin Therapy in Adults With Type 1 Diabetes: The North American inTandem1 Study. *Diabetes Care* **2018**, *41*, 1970-1980, doi:10.2337/dc18-0343.
191. Frias, J.P.; Nauck, M.A.; Van, J.; Kutner, M.E.; Cui, X.; Benson, C.; Urva, S.; Gimeno, R.E.; Milicevic, Z.; Robins, D.; et al. Efficacy and safety of LY3298176, a novel dual GIP and GLP-1 receptor agonist, in patients with type 2 diabetes: a randomised, placebo-controlled and active comparator-controlled phase 2 trial. *Lancet* **2018**, *392*, 2180-2193, doi:10.1016/S0140-6736(18)32260-8.
192. Grunberger, G.; Camp, S.; Johnson, J.; Huyck, S.; Terra, S.G.; Mancuso, J.P.; Jiang, Z.W.; Golm, G.; Engel, S.S.; Lauring, B. Ertugliflozin in Patients with Stage 3 Chronic Kidney Disease and Type 2 Diabetes Mellitus: The VERTIS RENAL Randomized Study. *Diabetes Ther* **2018**, *9*, 49-66, doi:10.1007/s13300-017-0337-5.
193. Hernandez, A.F.; Green, J.B.; Janmohamed, S.; D'Agostino, R.B., Sr.; Granger, C.B.; Jones, N.P.; Leiter, L.A.; Rosenberg, A.E.; Sigmon, K.N.; Somerville, M.C.; et al. Albiglutide and cardiovascular outcomes in patients with type 2 diabetes and cardiovascular disease (Harmony Outcomes): a double-blind, randomised placebo-controlled trial. *Lancet* **2018**, *392*, 1519-1529, doi:10.1016/S0140-6736(18)32261-X.
194. O'Neil, P.M.; Birkenfeld, A.L.; McGowan, B.; Mosenzon, O.; Pedersen, S.D.; Wharton, S.; Carson, C.G.; Jepsen, C.H.; Kabisch, M.; Wilding,

J.P.H. Efficacy and safety of semaglutide compared with liraglutide and placebo for weight loss in patients with obesity: a randomised, double-blind, placebo and active controlled, dose-ranging, phase 2 trial. *Lancet* **2018**, *392*, 637-649, doi:10.1016/S0140-6736(18)31773-2.

195. Tuttle, K.R.; Lakshmanan, M.C.; Rayner, B.; Busch, R.S.; Zimmermann, A.G.; Woodward, D.B.; Botros, F.T. Dulaglutide versus insulin glargine in patients with type 2 diabetes and moderate-to-severe chronic kidney disease (AWARD-7): a multicentre, open-label, randomised trial. *Lancet Diabetes Endocrinol* **2018**, *6*, 605-617, doi:10.1016/S2213-8587(18)30104-9.

196. Aroda, V.R.; Bain, S.C.; Cariou, B.; Piletic, M.; Rose, L.; Axelsen, M.; Rowe, E.; DeVries, J.H. Efficacy and safety of once-weekly semaglutide versus once-daily insulin glargine as add-on to metformin (with or without sulfonylureas) in insulin-naive patients with type 2 diabetes (SUSTAIN 4): a randomised, open-label, parallel-group, multicentre, multinational, phase 3a trial. *Lancet Diabetes Endocrinol* **2017**, *5*, 355-366, doi:10.1016/S2213-8587(17)30085-2.

197. Holman, R.R.; Bethel, M.A.; Mentz, R.J.; Thompson, V.P.; Lokhnygina, Y.; Buse, J.B.; Chan, J.C.; Choi, J.; Gustavson, S.M.; Iqbal, N.; et al. Effects of Once-Weekly Exenatide on Cardiovascular Outcomes in Type 2 Diabetes. *N Engl J Med* **2017**, *377*, 1228-1239, doi:10.1056/NEJMoa1612917.

198. Meneilly, G.S.; Roy-Duval, C.; Alawi, H.; Dailey, G.; Bellido, D.; Trescoli, C.; Manrique Hurtado, H.; Guo, H.; Pilorget, V.; Perfetti, R.; et al. Lixisenatide Therapy in Older Patients With Type 2 Diabetes Inadequately Controlled on Their Current Antidiabetic Treatment: The GetGoal-O Randomized Trial. *Diabetes Care* **2017**, *40*, 485-493, doi:10.2337/dc16-2143.

199. Neal, B.; Perkovic, V.; Mahaffey, K.W.; de Zeeuw, D.; Fulcher, G.; Erondu, N.; Shaw, W.; Law, G.; Desai, M.; Matthews, D.R.; et al. Canagliflozin and Cardiovascular and Renal Events in Type 2 Diabetes. *N Engl J Med* **2017**, *377*, 644-657, doi:10.1056/NEJMoa1611925.

200. Marso, S.P.; Daniels, G.H.; Brown-Frandsen, K.; Kristensen, P.; Mann, J.F.; Nauck, M.A.; Nissen, S.E.; Pocock, S.; Poulter, N.R.; Ravn, L.S.; et al. Liraglutide and Cardiovascular Outcomes in Type 2 Diabetes. *N Engl J Med* **2016**, *375*, 311-322, doi:10.1056/NEJMoa1603827.

201. Marso, S.P.; Bain, S.C.; Consoli, A.; Eliaschewitz, F.G.; Jodar, E.; Leiter, L.A.; Lingvay, I.; Rosenstock, J.; Seufert, J.; Warren, M.L.; et al. Semaglutide and Cardiovascular Outcomes in Patients with Type 2 Diabetes. *N Engl J Med* **2016**, *375*, 1834-1844, doi:10.1056/NEJMoa1607141.

202. Cefalu, W.T.; Leiter, L.A.; de Bruin, T.W.; Gause-Nilsson, I.; Sugg, J.; Parikh, S.J. Dapagliflozin's Effects on Glycemia and Cardiovascular Risk Factors in High-Risk Patients With Type 2 Diabetes: A 24-Week, Multicenter, Randomized, Double-Blind, Placebo-Controlled Study With a 28-Week Extension. *Diabetes Care* **2015**, *38*, 1218-1227, doi:10.2337/dc14-0315.
203. Pi-Sunyer, X.; Astrup, A.; Fujioka, K.; Greenway, F.; Halpern, A.; Krempf, M.; Lau, D.C.; le Roux, C.W.; Violante Ortiz, R.; Jensen, C.B.; et al. A Randomized, Controlled Trial of 3.0 mg of Liraglutide in Weight Management. *N Engl J Med* **2015**, *373*, 11-22, doi:10.1056/NEJMoa1411892.
204. Zinman, B.; Wanner, C.; Lachin, J.M.; Fitchett, D.; Bluhmki, E.; Hantel, S.; Mattheus, M.; Devins, T.; Johansen, O.E.; Woerle, H.J.; et al. Empagliflozin, Cardiovascular Outcomes, and Mortality in Type 2 Diabetes. *N Engl J Med* **2015**, *373*, 2117-2128, doi:10.1056/NEJMoa1504720.
205. Barnett, A.H.; Mithal, A.; Manassie, J.; Jones, R.; Rattunde, H.; Woerle, H.J.; Broedl, U.C.; investigators, E.-R.R.t. Efficacy and safety of empagliflozin added to existing antidiabetes treatment in patients with type 2 diabetes and chronic kidney disease: a randomised, double-blind, placebo-controlled trial. *Lancet Diabetes Endocrinol* **2014**, *2*, 369-384, doi:10.1016/S2213-8587(13)70208-0.
206. Dungan, K.M.; Povedano, S.T.; Forst, T.; Gonzalez, J.G.; Atisso, C.; Sealls, W.; Fahrbach, J.L. Once-weekly dulaglutide versus once-daily liraglutide in metformin-treated patients with type 2 diabetes (AWARD-6): a randomised, open-label, phase 3, non-inferiority trial. *Lancet* **2014**, *384*, 1349-1357, doi:10.1016/S0140-6736(14)60976-4.
207. Pratley, R.E.; Nauck, M.A.; Barnett, A.H.; Feinglos, M.N.; Ovalle, F.; Harman-Boehm, I.; Ye, J.; Scott, R.; Johnson, S.; Stewart, M.; et al. Once-weekly albiglutide versus once-daily liraglutide in patients with type 2 diabetes inadequately controlled on oral drugs (HARMONY 7): a randomised, open-label, multicentre, non-inferiority phase 3 study. *Lancet Diabetes Endocrinol* **2014**, *2*, 289-297, doi:10.1016/S2213-8587(13)70214-6.
208. Ridderstrale, M.; Andersen, K.R.; Zeller, C.; Kim, G.; Woerle, H.J.; Broedl, U.C.; investigators, E.-R.H.H.S.t. Comparison of empagliflozin and glimepiride as add-on to metformin in patients with type 2 diabetes: a 104-week randomised, active-controlled, double-blind, phase 3 trial. *Lancet Diabetes Endocrinol* **2014**, *2*, 691-700, doi:10.1016/S2213-8587(14)70120-2.
209. Stenlof, K.; Cefalu, W.T.; Kim, K.A.; Jodar, E.; Alba, M.; Edwards, R.; Tong, C.; Canovatchel, W.; Meininger, G. Long-term efficacy and safety

of canagliflozin monotherapy in patients with type 2 diabetes inadequately controlled with diet and exercise: findings from the 52-week CANTATA-M study. *Curr Med Res Opin* **2014**, *30*, 163-175, doi:10.1185/03007995.2013.850066.
210. Weissman, P.N.; Carr, M.C.; Ye, J.; Cirkel, D.T.; Stewart, M.; Perry, C.; Pratley, R. HARMONY 4: randomised clinical trial comparing once-weekly albiglutide and insulin glargine in patients with type 2 diabetes inadequately controlled with metformin with or without sulfonylurea. *Diabetologia* **2014**, *57*, 2475-2484, doi:10.1007/s00125-014-3360-3.
211. Lavalle-Gonzalez, F.J.; Januszewicz, A.; Davidson, J.; Tong, C.; Qiu, R.; Canovatchel, W.; Meininger, G. Efficacy and safety of canagliflozin compared with placebo and sitagliptin in patients with type 2 diabetes on background metformin monotherapy: a randomised trial. *Diabetologia* **2013**, *56*, 2582-2592, doi:10.1007/s00125-013-3039-1.
212. Gallwitz, B.; Guzman, J.; Dotta, F.; Guerci, B.; Simo, R.; Basson, B.R.; Festa, A.; Kiljanski, J.; Sapin, H.; Trautmann, M.; et al. Exenatide twice daily versus glimepiride for prevention of glycaemic deterioration in patients with type 2 diabetes with metformin failure (EUREXA): an open-label, randomised controlled trial. *Lancet* **2012**, *379*, 2270-2278, doi:10.1016/S0140-6736(12)60479-6.
213. Gallwitz, B.; Bohmer, M.; Segiet, T.; Molle, A.; Milek, K.; Becker, B.; Helsberg, K.; Petto, H.; Peters, N.; Bachmann, O. Exenatide twice daily versus premixed insulin aspart 70/30 in metformin-treated patients with type 2 diabetes: a randomized 26-week study on glycemic control and hypoglycemia. *Diabetes Care* **2011**, *34*, 604-606, doi:10.2337/dc10-1900.
214. Bailey, C.J.; Gross, J.L.; Pieters, A.; Bastien, A.; List, J.F. Effect of dapagliflozin in patients with type 2 diabetes who have inadequate glycaemic control with metformin: a randomised, double-blind, placebo-controlled trial. *Lancet* **2010**, *375*, 2223-2233, doi:10.1016/S0140-6736(10)60407-2.
215. Nauck, M.; Frid, A.; Hermansen, K.; Shah, N.S.; Tankova, T.; Mitha, I.H.; Zdravkovic, M.; During, M.; Matthews, D.R.; Group, L.-S. Efficacy and safety comparison of liraglutide, glimepiride, and placebo, all in combination with metformin, in type 2 diabetes: the LEAD (liraglutide effect and action in diabetes)-2 study. *Diabetes Care* **2009**, *32*, 84-90, doi:10.2337/dc08-1355.
